# Building Programs to Eradicate Toxoplasmosis Part II: Education

**DOI:** 10.1007/s40124-022-00267-y

**Published:** 2022-08-01

**Authors:** Mariangela Soberón Felín, Kanix Wang, Aliya Moreira, Andrew Grose, Karen Leahy, Ying Zhou, Fatima Alibana Clouser, Maryam Siddiqui, Nicole Leong, Perpetua Goodall, Morgan Michalowski, Mahmoud Ismail, Monica Christmas, Stephen Schrantz, Zuleima Caballero, Ximena Norero, Dora Estripeaut, David Ellis, Catalina Raggi, Catherine Castro, Claudia Rengifo-Herrera, Davina Moossazadeh, Margarita Ramirez, Abhinav Pandey, Kevin Ashi, Samantha Dovgin, Ashtyn Dixon, Xuan Li, Ian Begeman, Sharon Heichman, Joseph Lykins, Delba Villalobos-Cerrud, Lorena Fabrega, José Luis Sanchez Montalvo, Connie Mendivil, Mario R. Quijada, Silvia Fernández-Pirla, Valli de La Guardia, Digna Wong, Mayrene Ladrón de Guevara, Carlos Flores, Jovanna Borace, Anabel García, Natividad Caballero, Maria Theresa Moreno de Saez, Michael Politis, Stephanie Ross, Mimansa Dogra, Vishan Dhamsania, Nicholas Graves, Marci Kirchberg, Kopal Mathur, Ashley Aue, Carlos M. Restrepo, Alejandro Llanes, German Guzman, Arturo Rebellon, Kenneth Boyer, Peter Heydemann, A. Gwendolyn Noble, Charles Swisher, Peter Rabiah, Shawn Withers, Teri Hull, David Frim, David McLone, Chunlei Su, Michael Blair, Paul Latkany, Ernest Mui, Daniel Vitor Vasconcelos-Santos, Alcibiades Villareal, Ambar Perez, Carlos Andrés Naranjo Galvis, Mónica Vargas Montes, Nestor Ivan Cardona Perez, Morgan Ramirez, Cy Chittenden, Edward Wang, Laura Lorena Garcia-López, Juliana Muñoz-Ortiz, Nicolás Rivera-Valdivia, María Cristina Bohorquez-Granados, Gabriela Castaño de-la-Torre, Guillermo Padrieu, Juan David Valencia Hernandez, Daniel Celis-Giraldo, John Alejandro Acosta Dávila, Elizabeth Torres, Manuela Mejia Oquendo, José Y. Arteaga-Rivera, Dan L. Nicolae, Andrey Rzhetsky, Nancy Roizen, Eileen Stillwaggon, Larry Sawers, Francois Peyron, Martine Wallon, Emanuelle Chapey, Pauline Levigne, Carmen Charter, Migdalia De Frias, Jose Montoya, Cindy Press, Raymund Ramirez, Despina Contopoulos-Ioannidis, Yvonne Maldonado, Oliver Liesenfeld, Carlos Gomez, Kelsey Wheeler, Samantha Zehar, James McAuley, Denis Limonne, Sandrine Houze, Sylvie Abraham, Raphael Piarroux, Vera Tesic, Kathleen Beavis, Ana Abeleda, Mari Sautter, Bouchra El Mansouri, Adlaoui El Bachir, Fatima Amarir, Kamal El Bissati, Ellen Holfels, Richard Penn, William Cohen, Alejandra de-la-Torre, Gabrielle Britton, Jorge Motta, Eduardo Ortega-Barria, Isabel Luz Romero, Paul Meier, Michael Grigg, Jorge Gómez-Marín, Jagannatha Rao Kosagisharaf, Xavier Sáez Llorens, Osvaldo Reyes, Rima McLeod

**Affiliations:** 1 Toxoplasmosis Programs and Initiatives in Panamá, Panama City, Panama; 2 Institute for Genomics and Systems Biology, The University of Chicago, Chicago, IL, USA; 3 Pritzker School of Medicine, The University of Chicago, Chicago, IL, USA; 4 Instituto de Investigaciones Científicas y Servicios de Alta Tecnología AIP (INDICASAT-AIP), Panama City, Panama; 5 Department of Pediatrics Infectious Diseases/Department of Neonatology, Hospital del Niño Doctor José Renán Esquivel, Panama City, Panama; 6 Department of Ophthalmology and Visual Sciences, The University of Chicago, Chicago, IL, USA; 7 The College, The University of Chicago, Chicago, IL, USA; 8 The Global Health Center, The University of Chicago, Chicago, IL, USA; 9 Universidad de Panamá, Panama City, Panama; 10 Department of Statistics, The University of Chicago, Chicago, IL, USA; 11 Rush University Medical School/Rush University Medical Center, Chicago, IL, USA; 12 Academia Interamericana de Panamá, Panama City, Panama; 13 Hospital Santo Tomás, Panama City, Panama; 14 Hospital San Miguel de Arcangel, Ciudad de Panama, Panama; 15 Capstone Program, Global Health Center, The University of Chicago, Chicago, IL, USA; 16 Harris School of Public Policy, The University of Chicago, Chicago, IL, USA; 17 Sanofi Aventis de Panamá S.A., University of South Florida, Panama City, Panama; 18 Northwestern University Feinberg School of Medicine, Chicago, IL, USA; 19 NorthShore Evanston Hospital, Evanston, IL, USA; 20 Department of Microbiology, The University of Tennessee, Knoxville, TN, USA; 21 Universidad de Federal de Minas Gerais, Belo Horizonte, Minas Gerais, Brazil; 22 Universidad Autónoma de Manizales, Manizales, Colombia; 23 Universidad del Quindío, Armenia, Colombia; 24 Grupo de Investigación en Neurociencias, Universidad del Rosario, Bogotá, Colombia; 25 The University of South Florida College of Public Health, Tampa, FL, USA; 26 Department of Economics, Gettysburg College, Gettysburg, PA, USA; 27 Department of Economics, American University, D, Washington .C, USA; 28 Institut des agents infectieux, Hôpital de la Croix-Rousse, Lyon, France; 29 Remington Specialty Laboratory, Palo Alto, CA, USA; 30 Department of Pediatrics, Division of Infectious Diseases, Stanford University College of Medicine, Stanford, CA, USA; 31 Roche Molecular Diagnostics, Pleasanton, CA, USA; 32 LDBioDiagnostics, Lyon, France; 33 Laboratory of Parasitologie, Bichat-Claude Bernard Hopital, Paris, France; 34 INH, Rabat, Morocco; 35 Faculty of Sciences Ain Chock, University Hassan II, Casablanca, Morocco; 36 Sistema Nacional de investigadores de Panamá (SNI), Panama City, Panama; 37 Secretaría Nacional de Ciencia, Tecnología e Innovación (SENACYT), Panama City, Panama; 38 GSK Vaccines, Panama City, Panama; 39 Molecular Parasitology Section, Laboratory of Parasitic Diseases, National Institutes of Health, NIAID, Bethesda, MD, USA; 40 Toxoplasmosis Center, The University of Chicago, and Toxoplasmosis Research Institute, Chicago, IL, USA; 41 Department of Pediatrics (Infectious Diseases), The University of Chicago, Chicago, IL, USA

**Keywords:** *Toxoplasma*, Toxoplasmosis, Education, Primary prevention

## Abstract

**Purpose of Review:**

Review work to create and evaluate educational materials that could serve as a primary prevention strategy to help both providers and patients in Panama, Colombia, and the USA reduce disease burden of *Toxoplasma* infections.

**Recent Findings:**

Educational programs had not been evaluated for efficacy in Panama, USA, or Colombia.

**Summary:**

Educational programs for high school students, pregnant women, medical students and professionals, scientists, and lay personnel were created. In most settings, short-term effects were evaluated. In Panama, Colombia, and USA, all materials showed short-term utility in transmitting information to learners. These educational materials can serve as a component of larger public health programs to lower disease burden from congenital toxoplasmosis. Future priorities include conducting robust longitudinal studies of whether education correlates with reduced adverse disease outcomes, modifying educational materials as new information regarding region-specific risk factors is discovered, and ensuring materials are widely accessible.

## Introduction

While nearly half of everyone on Earth is seropositive for *Toxoplasma gondii*, eliminating the congenital form of toxoplasmosis can ameliorate some of this parasite’s most severe effects [1]. As some of the worst consequences of untreated congenital toxoplasmosis (CT) include chorioretinitis, hydrocephalus, and blindness, the estimated 190,100 new infected infants each year are responsible for 1.20 million disability-adjusted life years worldwide [2••, 3•]. Prevention and prompt treatment of gestational *Toxoplasma* infection have already become priorities of global health research programs [4••].

Reducing CT rates requires early treatment of infected mothers, but within primary prevention strategies, prenatal education could help women avoid acquiring *Toxoplasma* during gestation [2••]. Recent attention to education comes from findings that general knowledge of toxoplasmosis among pregnant women is incomplete around the globe [5–11]. Studies on background knowledge comment on a need for systematic education of patients in the prenatal care setting. Meanwhile, a 2015 assessment suggests that readily accessible, high-quality, Internet-based sources of information are also a needed resource [12]. An education-based primary prevention strategy with easily accessible learning materials has been proposed as part of a comprehensive public health approach to CT [4••].

As part of the educational initiative, ensuring providers are also well informed about toxoplasmosis is also an issue that has gained recent attention. A 2010 US survey of obstetrician-gynecologists pointed to several gaps in providers’ approaches to toxoplasmosis prevention, such as counseling about all possible risk factors [13]. A later study in the USA corroborated these findings, noting that risk factor counseling and knowledge of screening approaches were inconsistent [14•]. Later surveys of providers’ knowledge in Poland and Mexico suggest that the need for improved education of prenatal care providers about CT, and about toxoplasmosis more generally, is a global problem [15, 16]. From our own early experiences in Panama, our discovery that providers’ compliance with a law that required reporting of toxoplasmosis was very low (see the “Results” section in this paper and Part IV) made us suspect that gaps in physicians’ knowledge were a barrier to reducing CT’s impact.

Taking into account the existing gaps in knowledge regarding toxoplasmosis, this segment of the broader public health initiative developed and tested a series of educational modules for patients, providers, and the public in Panama, Colombia, and the USA.

## Appproach

### Structure of Study and Overview

The Box shows the structure of the studies in consecutive summers or rotations of medical students to Panama and Colombia and then comparisons of teaching tools in the USA (Box). The same structure is shown in the “Approach” and “Updates” sections for corresponding time period subsequently.

### Initial Analysis of *Toxoplasma* Seroprevalence in Panama (2014)

Our initial study of *Toxoplasma* seroprevalence in 2014—conducted by researchers from Hospital del Niño (HdN) in Ciudad de Panamá—was a major starting point for studies that focused specifically on how to educate patients and providers regarding toxoplasmosis. This seroprevalence study was a cross-sectional analysis of pregnant women and their newborns at Hospital Santo Tomás (HST). With 1,320 births/month at HST’s maternity ward, one serum sample was collected every 21 births to reach a pre-determined sample size of 366. Twenty additional samples were collected to make up for women who refused to participate in the study. Inclusion criteria required participants to have given birth between June and November of 2014 and to have completed all screenings. Blood collected from participants and their newborns was analyzed via ELISA for anti-*Toxoplasma* IgM and IgG antibodies. Results were considered positive at 0.6 (>= 1 IU/Ml) (Supplement: Montenegro Vasquez et al.).

### High School–Based Educational Initiatives: Academia Interamericana de Panamá (2015)

**Fig. 1:**
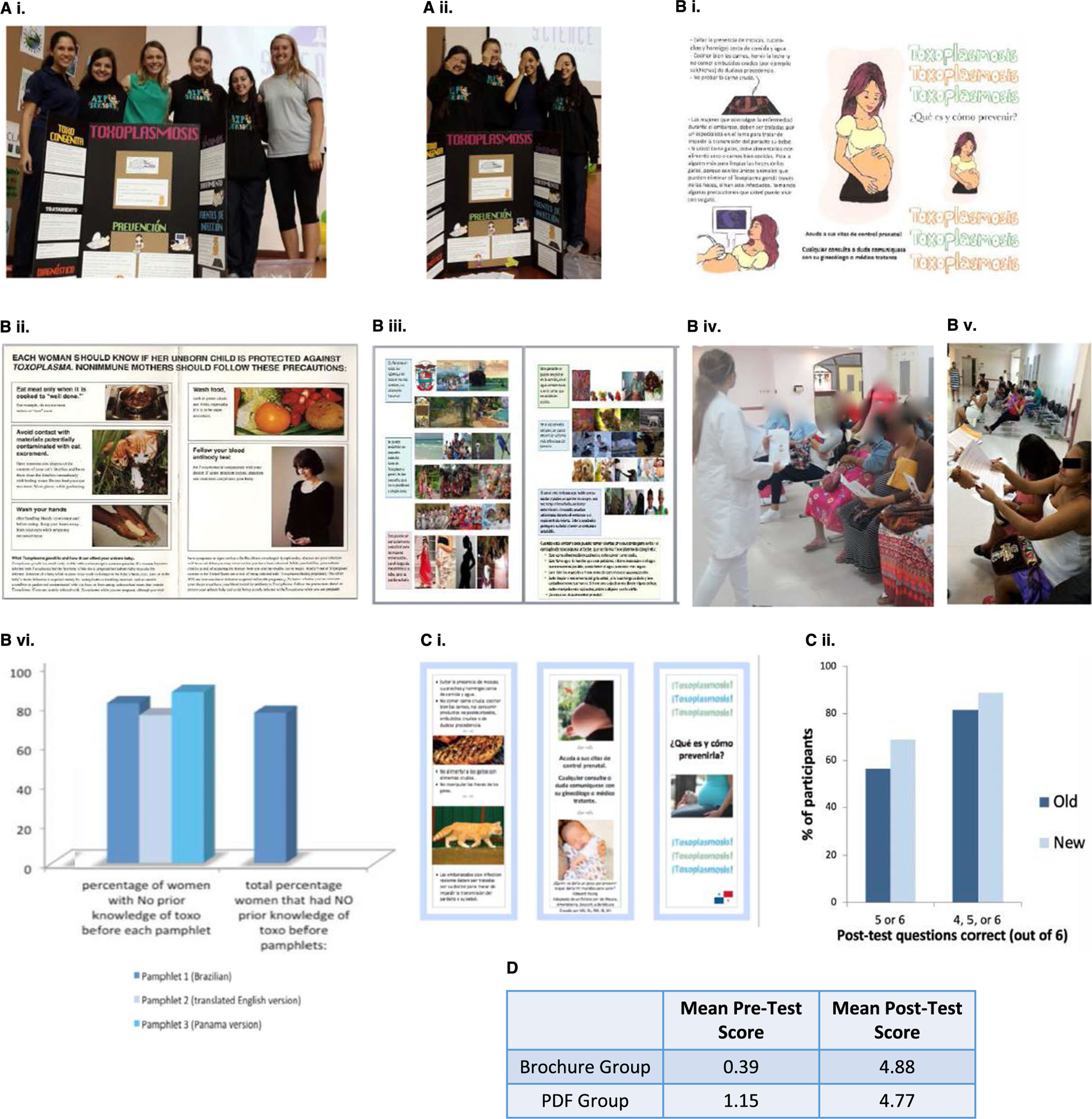
Beginning of toxoplasmosis education programs in Panama. Ai. Poster presentation by a group of AIP students. (Aii) “Why am I covering one eye?” campaign, part of the same group presentation that sought to educate both students and the public about *Toxoplasma*. (Bi) Educational Pamphlet 1 (Brazilian), developed by Fernanda Loureiro de Moura. (Bii) Educational Pamphlet 2 (translated English version). (Biii) Educational Pamphlet 3 (Panama-specific). (Biv) Participants reading pamphlets in the waiting room at Hospital Santo Tomás in Ciudad de Panamá. (Bv) Participants completing post-questionnaire in the waiting room at Hospital Santo Tomás. (Bvi) Comparison of effectiveness of pamphlets 1, 2, and 3. (Ci) Educational Pamphlet 4 (new and Panama-specific). (Cii) Comparison of New Panama-specific pamphlet (Educational Pamphlet 4) and old Brazilian Pamphlet (Educational Pamphlet 1). (D) Effectiveness of PDF vs printed Educational Pamphlet 4 Bi included with permission from de Moura(18) and Epidemiol Serv Saude.

While Montenegro Vasquez et al.’s work (see Results) led to passage of a law mandating screening of pregnant women for anti-*Toxoplasma* antibodies twice during gestation (see Results and Supplement: MINSA et al.), this study also inspired a weeklong educational campaign on toxoplasmosis for students at the Academia Interamericana de Panamá (AIP) in Ciudad de Panamá. This campaign adapted teaching materials that had been developed in the USA and previously used in settings such as grand rounds presentations for infectious disease fellows at the University of Chicago Medical Center (UCMC). Several AIP students also created posters and presentations that showed their understanding of what they had learned (Fig. 1 and 1).

### Evaluating Toxoplasmosis Learning Tools for Pregnant Women in Panama (2015–2016)

The Montenegro Vasquez et al. study also inspired a series of studies on education of pregnant women who would be screened for anti-*Toxoplasma* antibodies. The first study, conducted at HST in 2015, compared short-term teaching effectiveness of three pamphlets addressing toxoplasmosis: one from Brazil (Fig. 1), another from the UCMC (Fig. 1), and the third newly created for this research [7, 17, 18]. Eligible participants included pregnant women receiving routine care at HST’s maternity ward. Participants completed a six-question pre-test assessing preexisting knowledge about toxoplasmosis, were randomized to read one of the pamphlets, and completed the same questionnaire as a post-test. Pre- and post-intervention scores were compared (Supplement: Li et al.).

In light of this study’s results, the newest pamphlet was then updated (Fig. 1) and compared to the Brazilian pamphlet in a follow-up study. Pregnant women were recruited from the waiting room for high-risk obstetric clinic appointments at HST (Fig. 1 and 1), and methods outlined above were repeated (Supplement: Heichman et al.).

**Fig. 2:**
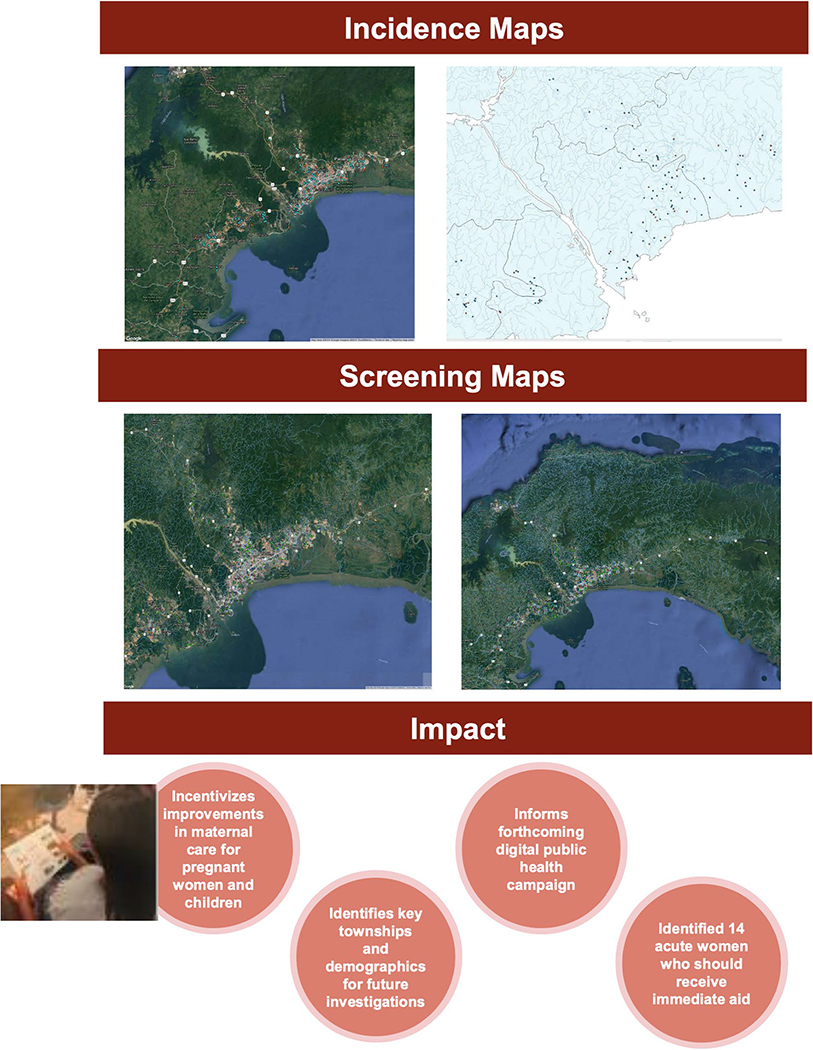
Maps and summary slides from presentation by Abhi Pandey and Aliya Moreira that details their studies in Panama. One component of their research was a study on the effectiveness of using digital media to teach pregnant women about congenital toxoplasmosis. Pandey and Moreira also created incidence and screening maps for toxoplasmosis in Panama; these maps were based on screening data, IgG/IgM test results, demographic data, and addresses from prenatal control charts. Also shown as Fig 1 in Part III

A third study compared the teaching effectiveness of the newest, Panama-specific pamphlet with that of a digital, iPhone-compatible PDF version of the same pamphlet (Fig. 1). Eligible participants included pregnant women who had been surveyed over eight weeks in HST. Li et al.’s methods were repeated (Fig. 2; Supplement: Moreira & Pandey et al.).

### Evaluating Toxoplasmosis Learning Tools for Pregnant Women in Colombia (2019)

**Fig. 3:**
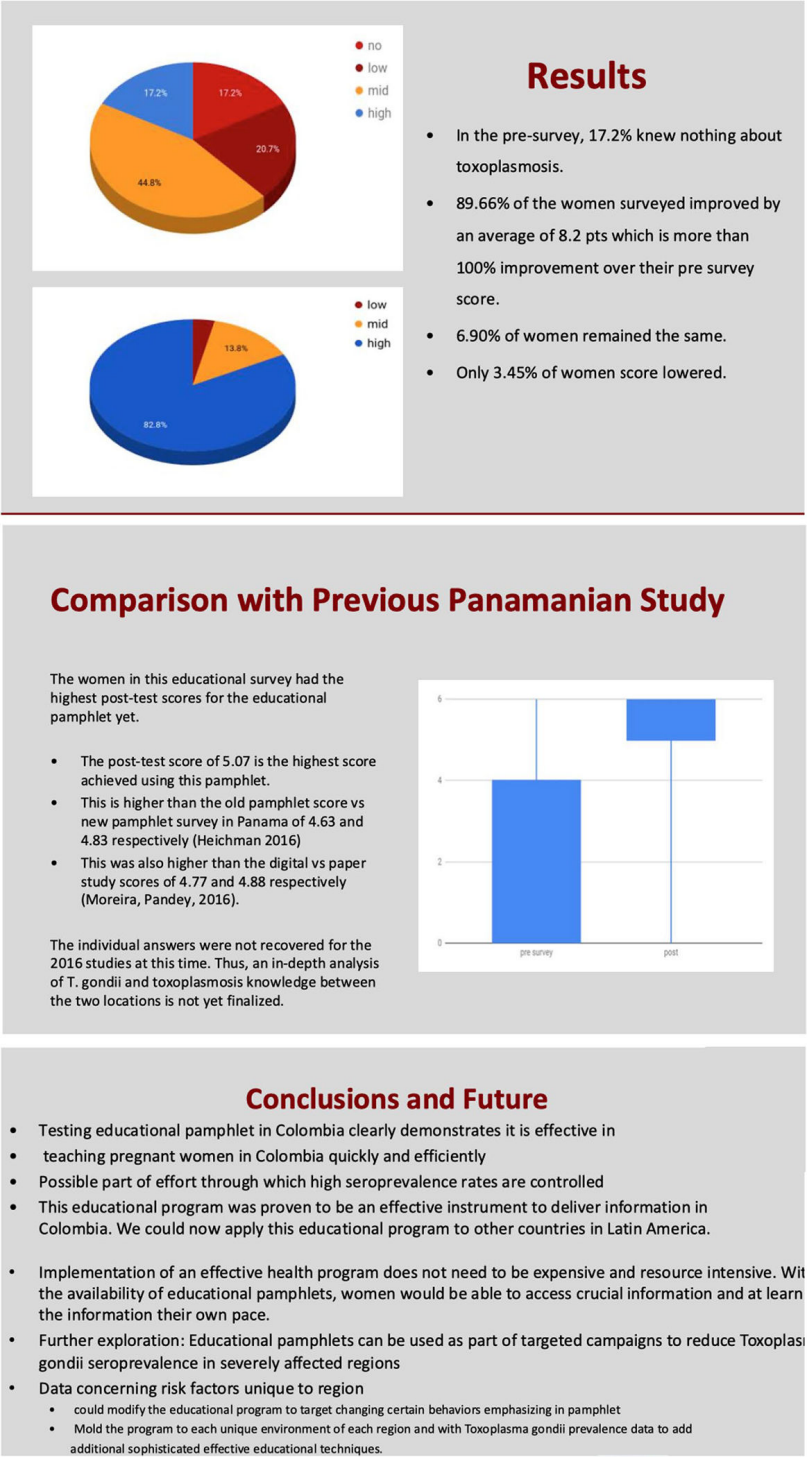
Data and conclusion slides from presentation by Jose Sanchez that outlines an educational study with pregnant women in Hospital del Sur and Hospital San Juan de Dios in Armenia, Colombia. An educational pamphlet developed for Panama was adapted to account for unique parasite transmission risk factors in Colombia. See Supplement for complete presentation

A 2019 study in Armenia, Colombia, measured the baseline level of knowledge on toxoplasmosis among Armenia’s pregnant population and determined the teaching effectiveness of the newest, Panama-specific pamphlet (Fig. 1)—which was edited to include a comment on infection risks of untreated water. Eligible participants included pregnant women in the waiting rooms of Hospital del Sur and Hospital San Juan de Dios. Li et al.’s methods were repeated (Fig. 3; Supplement: Sanchez et al.).

### Evaluating Toxoplasmosis Learning Tools for Medical Students and Professionals in Panama (2017)

A 2017 study measured short-term effectiveness of toxoplasmosis-related educational interventions for physicians and medical students. Medical students enrolled in accredited Panamanian medical schools and healthcare providers in Panamanian hospitals or clinics that provide maternal-fetal healthcare were eligible to participate. Participants completed a 25-question survey that tested knowledge on toxoplasmosis, watched a 25-min educational PowerPoint presentation, and then re-took the survey. Average scores (in aggregate and for each question) were compared in order to evaluate changes from baseline and intervention efficacy. Qualitative data from open-ended questions on screening were also collected and coded (Supplement: Castro et al.).

### Adapting Learning Tools to a Small Medical Student Cohort in the USA (2020)

A US study adapted Castro et al.’s methods to a convenience sample of 37 first-year medical students from the Pritzker School of Medicine at the UCMC. Quiz scores were compared both within the US sample pre- and post-intervention, and between the 2020 US and 2017 Panama student groups (Grose et al.).

## Updates

### Initial Analysis of *Toxoplasma* Seroprevalence in Panama (2014)

**Fig. 4:**
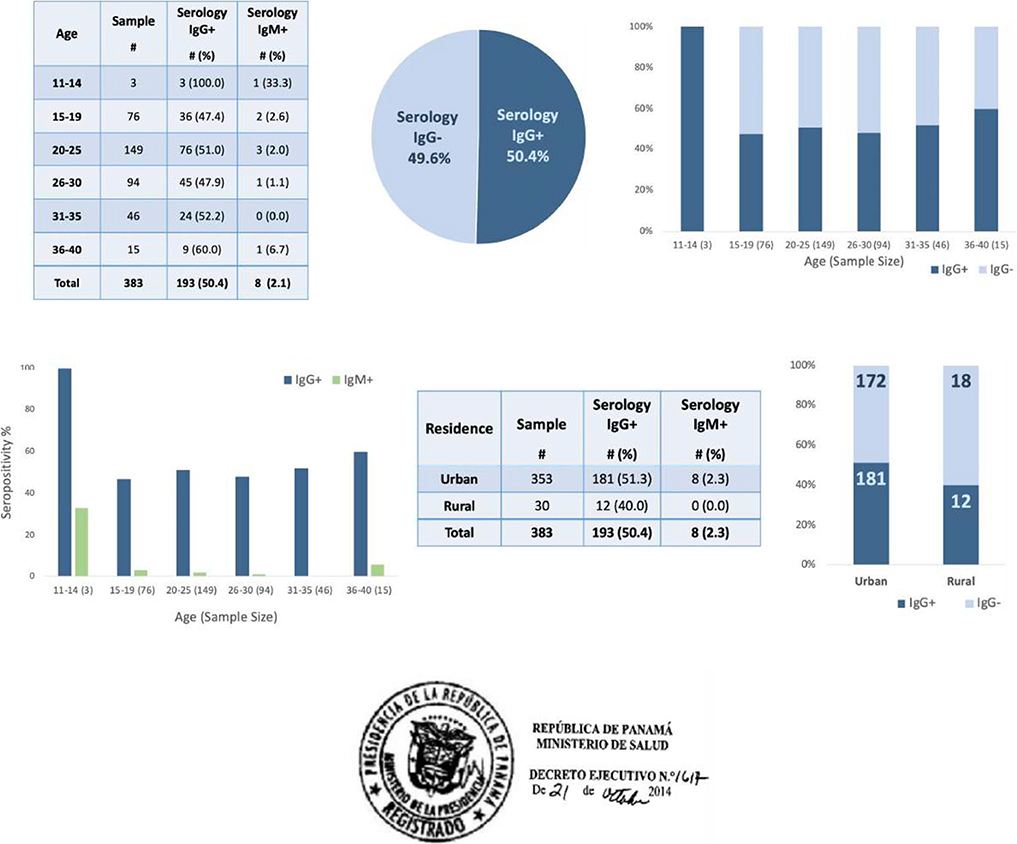
Prevalence of *Toxoplasma gondii* infection in pregnant women in 2014 at Hospital Santo Tomás in Ciudad de Panamá, Panama. (Ai) Prevalence of anti-*Toxoplasma* IgG+ and IgM+. (Aii) *Toxoplasma* IgG+ vs IgG−. (Aiii) *Toxoplasma* serology IgG+ and IgG− with age. (Aiv) *Toxoplasma* serology IgG+ and IgM+ with age. (Bi) IgG+ and IgM+ seroprevalence in urban and rural areas. (Bii) *Toxoplasma* IgG+ and IgG− in urban and rural areas. (Ci) Seal of executive action passed in 2014 by the Panama Ministry of Health (MINSA) following this study

Out of 383 pregnant women, prevalence was about 50% overall (Fig. 4 and 4). Age distributions were similar between seronegative women and seropositive women (Fig. 4). Of eight pregnant women acutely infected with *Toxoplasma*, age skewed toward younger patients, as 75% of the women who were IgM seropositive were 25 years old or younger (Fig. 4 and 4). Ninety-two percent of the women in this study lived in cities (Fig. 4). Of the 30 mothers who lived in rural areas, 40% were seropositive, compared to 51% seropositive urban mothers (Fig. 4).

High overall prevalence of toxoplasmosis indicated that *Toxoplasma* infection comprised a significant public health problem in Panama for pregnant women seeking care at HST. Following this study, a law was passed mandating screening for *Toxoplasma* twice during gestation (Fig. 4; Supplement: MINSA et al.).

### High School–Based Educational Initiatives: Academia Interamericana de Panamá (2015)

Although the teaching materials used with AIP students were not incorporated into formal research, these resources were meant to help increase students’ knowledge of toxoplasmosis. According to correspondence with several of the students who were part of the weeklong program, the early exposure to this information in 2015 has had an impact on participants’ career considerations. Several students have gone on to work with Institute of Scientific Research and High Technology Services of Panama (INDICASAT in Panama), while others have created their own learning resources on toxoplasmosis in the university setting (e.g., Supplement: Fernández Pirla et al.).

### Evaluating Toxoplasmosis Learning Tools for Pregnant Women in Panama (2015–2016)

Li et al. found that 76% of participants at HST (*N*=164) had no prior knowledge of toxoplasmosis (Fig. 1). The Brazilian pamphlet performed best in increasing knowledge: 89% of participants correctly answered at least five out of six questions on a post-intervention test, compared to 63% and 55% for the Panama-specific pamphlet and the UCMC pamphlet, respectively.

Heichman et al. found that 65% of the 156 participants had no relevant knowledge of toxoplasmosis, answering zero pre-test questions correctly. The new Panama-specific pamphlet was found to be significantly more effective in the short term than the Brazilian pamphlet (Fig. 3).

Moreira and Pandey et al. found that the group of women who used the hard-copy pamphlet group (*N*=105) had a net test score increase of 4.49 x ± S.D. 1.39 and in the I Phone group (*N*=109) had a net increase of 3.62 $\bar{x}$ ± SD 1.79 points. Based on a group-by-time interaction in a mixed-effects regression model, the brochure group performed significantly better than the group (*p*<0.001; Fig. 1), but both methods significantly increased scores (*p*<0.001).

### Effectiveness of Toxoplasmosis Learning Tools for Pregnant Women in Colombia (2019)

Sanchez et al. found that background knowledge among participants in Armenia was low, but 90% of women improved their scores by more than 100% post-intervention. Individual survey items were scored on a 0–3 scale with higher scores indicating greater understanding, and a total score was calculated based on the sum of the six items (possible range 0–18). Due to the paired design, Wilcoxon signed-rank tests were used to assess pre vs. post differences. Results for the individual items were also confirmed using a symmetry test. There was an overall increase in understanding from pre to post (*p*=0.004 for the total score); this was also true for individual items (*p*<0.05) except for the item about toxoplasmosis prevention (*p*=0.11).

**NB** In Panama, survey questions were graded pass/fail, and the total number of correct questions determined scores. Applying this same grading scale, the average pre-intervention score in Colombia was 2, and the average post-score is 5.07 (*p*<0.001). With average post-intervention score of the old and new pamphlet being 4.63 and 4.83, respectively, the average score of 5.07 achieved in Colombia was higher than the final average score in Panama (Fig. 3).

### Evaluating Toxoplasmosis Learning Tools for Medical Students and Professionals in Panama (2017)

**Fig. 5:**
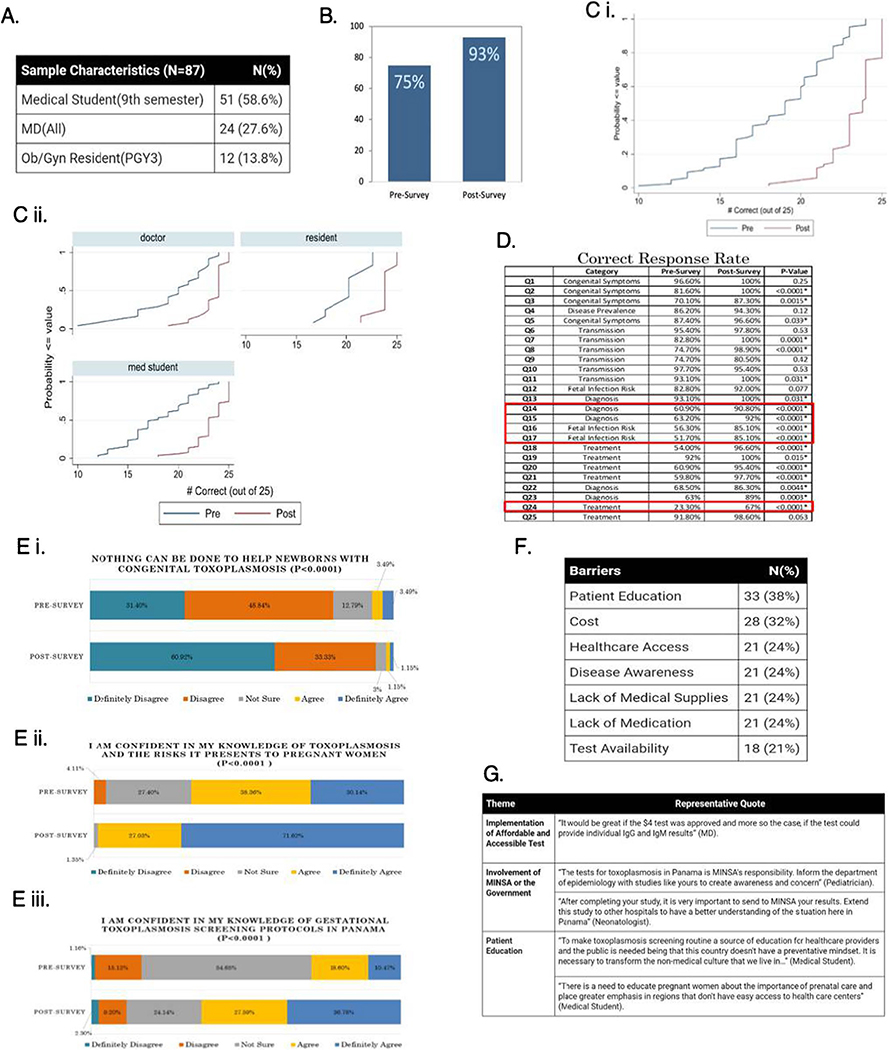
Results of Castro’s 2017 project on educational modules for medical students and medical professionals in Ciudad de Panamá. (A) Demographics included 87 participants from Universidad Latina, Policlínica Betania, Hospital Santo Tomás, and Hospital San Miguel Arcángel. (B) Correct response rates for pre and post surveys. There was an 18.32% increase from pre-survey mean score 74.96% when compared to the post-intervention mean score of 93.28% (*p*<0.001). (Ci) Mean correct response rates overall. (Cii) Mean correct response rates by demographic. (D). Highest score improvements were observed in questions about CT treatment. (Ei/Eii/Eiii) Following the intervention, participants reported greater confidence in their knowledge of CT treatment; of CT risks; and of CT diagnosis. (F) Respondents most commonly selected patient education (38%) and cost (32%) as barriers to improving toxoplasmosis care. (G) Example of qualitative analysis of participant feedback

Castro et al.’s study reached 119 participants (Fig. 5) and had a 73% response rate. Post-intervention scores increased by 18.32% (*p*<0.001; Fig. 5 and 5). Interventions effectively improved toxoplasmosis knowledge, knowledge confidence, and attitudes and beliefs (Fig. 5 and 5), with statistically significant increases in scores on questions about diagnosis, fetal infection risk, and treatment (*p*<0.0001; Fig. 5). A greater number of respondents felt confident in their knowledge of toxoplasmosis and the risks it presents to pregnant women post-intervention (*p* <0.0001; Fig. 5). Finally, there was a 35% increase in participants confident in their knowledge of toxoplasmosis screening protocols for pregnant women in Panama (*p* <0.0001; Fig. 5).

Qualitative data showed that participants tended to report patient education, healthcare access, cost, test availability, and disease awareness as issues for women trying to access gestational toxoplasmosis screening and treatment (Fig. 5); they frequently mentioned medical supplies, medication, cost, test availability, physician knowledge, and patient volume as barriers physicians face in treating toxoplasmosis (Fig. 5).

### Adapting Learning Tools to a Small Medical Student Cohort in the USA (2020)

On average, US students scored significantly higher than had the 2017 Panama sample on the pre-presentation quiz (20.32 vs 18, *p* = 0.000331). US students also showed a statistically significant score increase following the presentation (3.11 points, *p* < 0.00001), bringing them to a final score similar to that of the Panama group (23.43 vs 23.29, *p* = 0.69).

Initial qualitative analysis of free-response answers pointed to need for increased knowledge about topics such as cost-effectiveness of a universal screening program, patient and physician awareness of toxoplasmosis, an unequally distributed disease burden, restrictions on access to care, and obstetricians’ clinical protocols (Supplement: Grose et al.).

## Discussion

With the assumption that primary prevention through education can significantly reduce the burden of CT, the education component of this project has sought to improve awareness regarding toxoplasmosis in multiple populations and to make the resources widely available.

The initial focus on high school students came in part from the idea that comprehensive CT screening programs should begin before women reach childbearing age. If it is known who within a population is seropositive for anti-*Toxoplasma* antibodies (and, by definition, not at risk of conceiving a child susceptible to CT), a specific population of seronegative women can be isolated and monitored for newly acquired infection during pregnancy. As such, it was considered important to cultivate understanding regarding toxoplasmosis and the importance of screening among young women and younger people in general. The weeklong educational initiative served as a potential model for youth educational programs that can be used in countries besides Panama and applied longitudinally to high school cohorts.

Formal education studies began with a comparison of educational pamphlets for pregnant women. The first two patient education–focused studies compared the effectiveness of three different pamphlets, while the third adapted the best one to a cost-saving, Internet format and showed that patients could learn just as effectively online. A later study in Colombia adapted the materials we had developed in Panama to the department of Quindío, where water-borne *Toxoplasma* infection is a more serious issue and use of bottled drinking water is associated with lower rates of *Toxoplasma* infection (see Part III). This last study showed that these educational strategies can be fine-tuned to country or community-specific risk factors, along with the public health intervention strategies necessary to reduce disease acquisition in a specific population.

Despite the short-term effectiveness of our educational materials, it is largely unclear whether such materials could actually influence long-term patient behavior—and, ultimately, CT rates. There is promising observational evidence from a Belgian study that studied this question between 1979 and 2000, but epidemiologic studies since then have largely failed to find a correlation between educational intervention and reduced seroconversion rates during gestation [19•, 20•]. More recently, a review by Di Mario et al. found only two cluster-randomized controlled trials of preventive educational interventions since 1989, and it was concluded that both of these studies had severe methodological issues [21••]. While making learning materials available to patients is a priority, a next step of work in Panama might involve robust randomized controlled trials that evaluate whether a relationship exists between patient education and outcomes related to CT. In the future determining whether material learned is retained or knowledge can be reinforced will be another important aspect of evaluating the teaching materials.

In any case, it has been shown that these educational materials can improve people’s knowledge in the short term. Putting these modules on a website (http://www.toxoplasmosis.org) will make them available for use by providers, patients, and the general public. Additionally, as risk factors unique to new regions of the globe are defined, these educational modules can be modified to emphasize specific preventive practices, such as boiling or filtering potable water.

Patient education was a priority of this project’s efforts but making educational materials available to medical providers and to the general public is another important pillar of this work. Both Castro and Grose’s studies point to gaps in the knowledge of providers and providers-in-training, which could theoretically be addressed using educational modules similar to those employed in both studies. These informational aids were developed because at present there is little available outside France, Belgium, Austria, and Brazil.

**Fig. 6:**
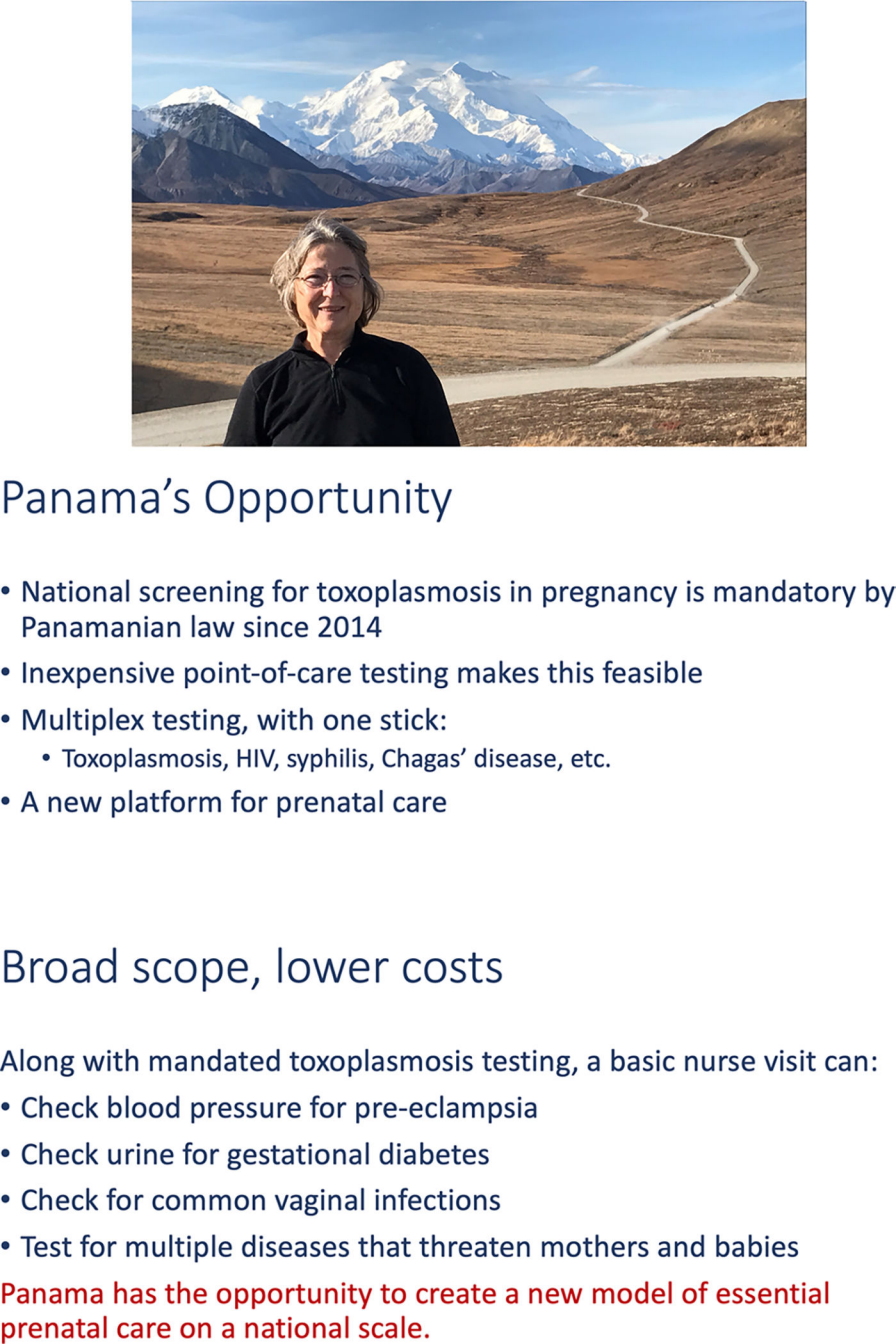
Impact slide from presentation by Eileen Stillwaggon and Rima McLeod at SENACYT symposium that details the economics of prenatal screening to prevent CT in Europe, particularly in Austria, a cost-benefit model for screening in the USA, and opportunities for a model of screening and prenatal care in Panama See Supplement for complete presentation

**Fig. 7:**
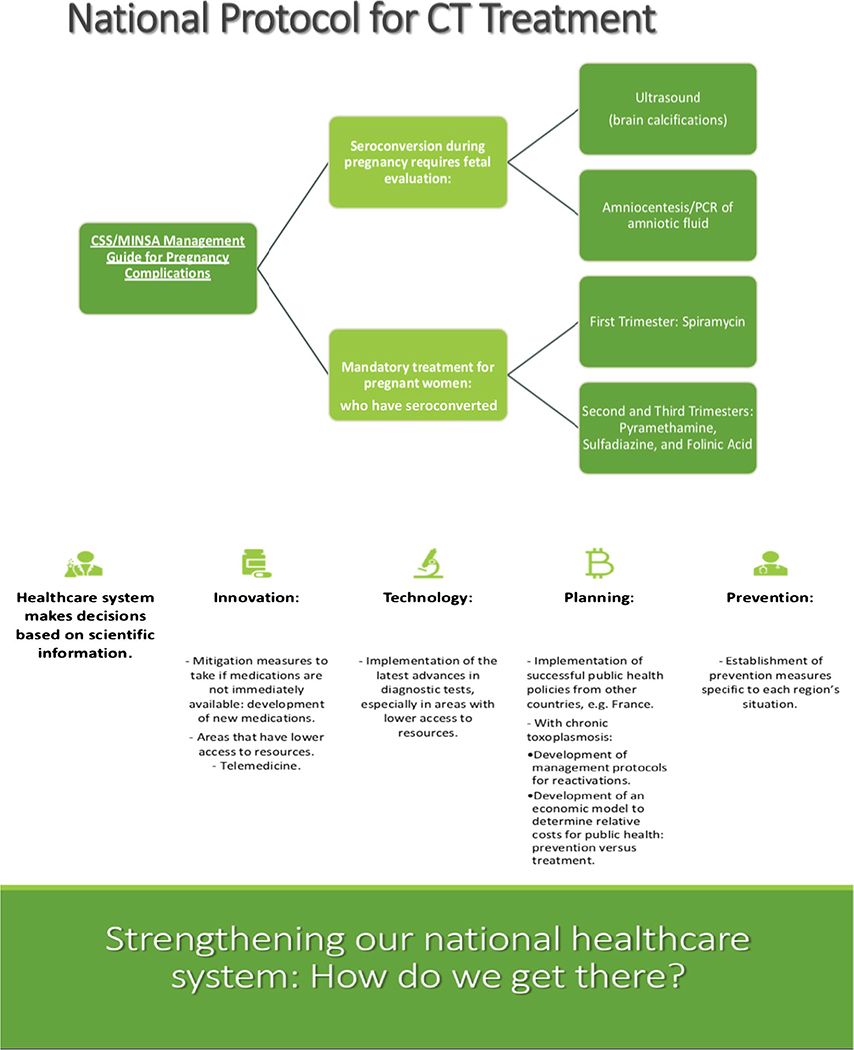
Conclusions slide from translation of a 2017 presentation by Mariángela Soberón Felín that examines compliance with mandatory reporting for congenital toxoplasmosis in Panama and makes suggestions for improving CT care. Full presentation is in the Supplemental

Outside of medicine, the importance of making the public more aware about toxoplasmosis has been acknowledged as well. Content from teaching materials has also been incorporated into a series of seminars, public presentations, and newspaper articles that addressed topics including routes of *Toxoplasma* infection, how to prevent acquisition of the parasite, and relative costs and benefits of CT screening programs in France and Austria. See Figures 6 and 7, along with Community Education (Miscellaneous) in the Supplement, for examples of these presentations.

## Conclusion

Altogether, the work described above to create better educational resources on toxoplasmosis has provided learning opportunities for patients, researchers, students, and medical professionals across Panama, Colombia, and the USA. A flexible and accessible set of learning materials regarding CT has set the stage for future studies that search for long-term relationships between educational initiatives on CT and disease outcomes.

## Supplementary Material

1832235_Sup_Material_1

1832235_Sup_Material_2

1832235_Sup_Material_3
