# Supplementary material for: Building Programs to Eradicate Toxoplasmosis Part II: Education
Source: Curr Pediatr Rep. Author manuscript; Available in PMC 2023 Mar 23. (PMC10035399; doi:10.1007/s40124-022-00267-y)
Supplement: 1832235_Sup_Material_1 [file NIHMS1832235-supplement-1832235_Sup_Material_1.pdf]

Supplemental Building Programs to Eradicate Toxoplasmosis Part II: Education

Mariangela Soberón Felín, JD[1*], Kanix Wang, PhD[2*], Catalina Raggi[3-7,9*], Aliya Moreira, MPH[3,7,9*] Abhinav Pandey[6,7,9*], Andrew Grose[3,6,9*], Zuleima Caballero, PhD[4*], Margarita Ramirez[6,7,9,] Davina Moossazadeh[6-9*], Catherine Castro, MD[3,6,9*], José Luis Sanchez Montalvo[6,7,9*], Karen Leahy, RN[3*], Ying Zhou, PhD[3,6*], Fatima Alibana Clouser[3,6*], Maryam Siddiqui, MD[3*], Nicole Leong, MD[3*], Perpetua Goodall, MD[3*], Morgan Michalowski, RN[3], Mahmoud Ismail, MD[3*], Monica Christmas, MD[3*], Stephen Schrantz, MD[3*], Ximena Norero, MD[5*], Dora Estripeaut, MD[5*], David Ellis, MD[5*], Kevin Ashi[3*], Samantha Dovgin[6,7*], Ashtyn Dixon, MD[6*], Xuan Li, MD[10*], Ian Begeman[3,6*], Sharon Heichman, MD[3,6*], Joseph Lykins, MD[3,6*], Delba Villalobos-Cerrud[4*], Lorena Fabrega[4*], Connie Mendivil[4*], Mario R. Quijada, BSc[4*], Silvia Fernández-Pirla[1,11*], Valli de La Guardia, MD[1,4,12], Digna Wong, MD[4*], Mayrene Ladrón de Guevara, MD[4,12*], Carlos Flores[12*], Jovanna Borace[12*], Anabel García, DVM[4*], Natividad Caballero, MD[13*], Claudia Rengifo-Herrera, DVM[4,14*], Maria Theresa Moreno de Saez, MD[5*], Michael Politis, MD[1*], Stephanie Ross, MD[10*], Mimansa Dogra,[6,7,9*] Vishan Dhamsania, MD,[3,15*] Nicholas Graves, MD,[3,15*] Marci Kirchberg, MS[15,16*], Kopal Mathur, MS[15,16*], Ashley Aue[9,16*], Carlos M. Restrepo, PhD[4*], Alejandro Llanes, PhD[4*], German Guzman[4*], Arturo Rebellon, MD[17*], Kenneth Boyer, MD[10*], Peter Heydemann, MD[10*], A. Gwendolyn Noble, MD, PhD[6,18*], Charles Swisher, MD[18*,**], Peter Rabiah, MD[19*], Shawn Withers, RN[6*], Teri Hull, PhD[3*], Chunlei Su, PhD[20*], Michael Blair MD[3,6], Paul Latkany MD[6*], Ernest Mui[6*], Daniel Vitor Vasconcelos-Santos, MD[21*], Alcibiades Villareal[4*], Ambar Perez, JD[4*], Carlos Andrés Naranjo Galvis, PhD[22*], Mónica Vargas Montes[23*], Nestor Ivan Cardona Perez, PhD[23*], Morgan Ramirez[6,7*], Cy Chittenden[6,7*], Edward Wang[6,7*], Laura Lorena Garcia-López,[23*], Guillermo Padrieu[24*], Juliana Muñoz-Ortiz[25*], Nicolás Rivera-Valdivia[25*], María Cristina Bohorquez-Granados[25*], Gabriela Castaño de-la-Torre[25*], Juan David Valencia Hernandez, BSc[23], Daniel Celis-Giraldo[23*], Juan Alejandro Acosta Dávila, PhD[25*], Elizabeth Torres, MSc[23*], Manuela Mejia Oquendo[23*], José Y. Arteaga-Rivera[25*], Dan L Nicolae, PhD[8*], Andrey Rzhetsky, PhD[2*], Nancy Roizen, MD[3*], Eileen Stillwaggon, PhD[26*,**], Larry Sawers, PhD[27*], Francois Peyron, MD, PhD[28*], Martine Wallon, MD[28*], Emanuelle Chapey[28*], Pauline Levigne, MD[6,28*], Carmen Charter, MD[12*], Migdalia De Frias, MD,[12*] Jose Montoya, MD[29*], Cindy Press[29*], Raymund Ramirez[29*], Despina Contopoulos-Ioannidis, MD[30*], Yvonne Maldonado, MD[30*], Oliver Liesenfeld, PhD, MD[31*], Carlos Gomez, MD[30*], Kelsey Wheeler, PhD[6,7*], Ellen Holfels[3,6], David Frim MD[3], David McLone MD PhD [18], Richard Penn MD [3], William Cohen[6,7], Samantha Zehar[18*], James McAuley MD, MPH[6,10*], Denis Limonne, Pharm D[32*], Sandrine Houze, MD[33], Sylvie Abraham[33], Raphael Piarroux, Pharm D, PhD[32*], Vera Tesic, MD, PhD[3*], Kathleen Beavis, MD, PhD[3*], Ana Abeleda[3*], Mari Sautter, MBA[3,6*], Bouchra El Mansouri[34*], Adlaoui El Bachir, DAPE[34*], Fatima Amarir, PhD[35*], Kamal El Bissati, PhD[3,34*], Alejandra de-la-Torre, MD, PhD[25*], Gabrielle Britton, PhD[4,36*], Jorge Motta, MD, MPH[37*], Eduardo Ortega-Barria, MD[36,37,38*], Isabel Luz Romero, MD[37*], Paul Meier, PhD[3**], Michael Grigg, PhD[39], Jorge Gómez-Marín, MD, PhD[23++], Jagannatha Rao Kosagisharaf, PhD[4*36++], Xavier Sáez Llorens, MD[5,36++], Osvaldo Reyes, MD[12,14,36++], Rima McLeod, MD, FACP, FIDSA, FAAAS[1-3,6,7,9,40,41++]

[1] Toxoplasmosis Programs and Initiatives in Panamá, Ciudad de Panamá, Panamá

[2] Institute for Genomics and Systems Biology, The University of Chicago, Chicago, Illinois

[3] Pritzker School of Medicine, The University of Chicago, Chicago, Illinois

[4] Instituto de Investigaciones Científicas y Servicios de Alta Tecnología AIP (INDICASAT-AIP), Ciudad de Panamá, Panamá

5. Department of Pediatrics Infectious Diseases/Department of Neonatology, Hospital del Niño doctor José Renán Esquivel, Ciudad de Panamá, Panamá

6. Department of Ophthalmology and Visual Sciences, The University of Chicago, Chicago, Illinois

[7] The College, The University of Chicago, Chicago, Illinois

[8] Department of Statistics, The University of Chicago, Chicago, Illinois
[9] The Global Health Center, The University of Chicago, Chicago, Illinois
[10] Rush University Medical School/Rush University Medical Center, Chicago, Illinois
[11] Academia Interamericana de Panamá, Ciudad de Panamá, Panamá
[12] Hospital Santo Tomás, Ciudad de Panamá, Panamá
[13] Hospital San Miguel Arcángel, Ciudad de Panamá, Panamá
[14] Universidad de Panamá, Ciudad de Panamá, Panamá
[15] Capstone Program, Global Health Center, The University of Chicago, Chicago, Illinois
[16] Harris School of Public Policy, The University of Chicago, Chicago, Illinois
[17] Sanofi Aventis de Panamá S.A., University of South Florida, Ciudad de Panamá, Panamá
[18] Northwestern University Feinberg School of Medicine, Chicago, Illinois
[19] NorthShore Evanston Hospital, Evanston, Illinois
[20] Department of Microbiology, The University of Tennessee, Knoxville, Tennessee
[21] Universidad de Federal de Minas Gerais, Minas Gerais, Brazil
[22] Universidad Autónoma de Manizales, Manizales, Colombia
[23] Universidad del Quindío, Armenia, Colombia
[24] The University of South Florida College of Public Health, Tampa, Florida
[25] Grupo de Investigación en Neurociencias, Universidad del Rosario, Bogotá, Colombia
[26] Department of Economics, Gettysburg College, Gettysburg, Pennsylvania
[27] Department of Economics, American University, Washington, D.C.
[28] Institut des agents infectieux, Hôpital de la Croix-Rousse, Lyon, France
[29] Remington Specialty Laboratory, Palo Alto, California
[30] Department of Pediatrics, Division of Infectious Diseases, Stanford University College of Medicine, Stanford, California
[31] Roche Molecular Diagnostics, Pleasanton California
[32] LDBioDiagnostics, Lyon, France
[33] Bichat-Claude Bernard Hopital,Laboratoire de Parasatologie,Paris ,France
[34] INH, Rabat, Morocco
[35] Faculty of Sciences Ain Chock, University Hassan II,Casablanca, Morocco
[36] Member of the Sistema Nacional de investigadores de Panamá (SNI)
[37] Secretaría Nacional de Ciencia, Tecnología e Innovación (SENACYT), Ciudad de Panamá, Panama
[38] GSK Vaccines, Panamá, Panamá
[39] Molecular Parasitology Programs, NIAID, NIH,Bethesda MD
[40] Toxoplasmosis Center, The University of Chicago, and Toxoplasmosis Research Institute, Chicago,Illinois
[41] Department of Pediatrics (Infectious Diseases), The University of Chicago, Chicago, Illinois

*Contributions to collection and/or analysis of primary data
**deceased

++To Whom Correspondence Should be Addressed

rmcleod@uchicago.edu

xsaezll@cwpanama.net

oreyespanama@yahoo.es

jrao@indicasat.org.pa

gepamol2@uniquindio.edu.co

Supplement Part II-Education: compilation of contributors' original research, writing, and presentations

Authors' note: Most of the materials in this supplement come from independent investigations conducted by students who were affiliated with global health research programs at the University of Chicago. As more student contributors were graciously invited to work with individuals and institutions in Panama and Colombia, the research that these students completed and presented became part of a truly international public health initiative, one that quickly involved more institutions and collaborators than many of us had originally conceived. None of these projects would have been possible without the collaboration of numerous U.S. and in-country partners. As such, each contributor's principal partners are highlighted in the title page of each section in this supplement.

Table of contents: Title page and Table of Contents Part II i-iv

Part A:

1. **MONTENEGRO VASQUEZ et al**…... ……………………………………………………1
Seroprevalence of *Toxoplasma gondii* infection in pregnant women (translated manuscript) 2

2.**MINISTERIO DE SALUD DE LA REPÚBLICA DE PANAMÁ (MINSA) et al**........ …………37
Decreto ejecutivo No. 1617(10/21/2014)...38

3.**LI, XUAN et al**.… ……………………………………………………………………52
Why Diagnose, Prevent and Treat Toxoplasmosis?(poster)..53
Toxoplasmosis Education for Pregnant Women in Panama (published abstract)...................... 54

4.**HEICHMAN, SHARON et al.**..56
. Effectiveness of Educational Materials about Toxoplasmosis (poster)………………………57
. Manuscript (same title)……….. ...58

5.**MOREIRA & PANDEY** et al.. 63
Investigating Social and Infrastructural Parameters (manuscript, excerpt)............................. 64
Excerpt of presentation (same title)... 75
Copy of educational pamphlet used in study...79

6.**SANCHEZ, JOSE et al**.. 81
Education as a Critical Tool to Aid in the Fight against Toxoplasmosis (poster).....................82
Manuscript (same title)..83
Presentation (same title) 105
Copy of educational pamphlet used in study..118

Part B

7 **CASTRO, CATHERINE** et al....1
Impact of Gestational and Congenital Toxoplasmosis (poster)....2
Manuscript (same title).... .3
Presentation (same title) *....18
Translation of educational presentation used in study.... .. 41

**Note: line drawing in presentation adapted with permission from Megli CJ and Coyne CB.Nature Rev Microbiol ,2022*

Part C

8.**GROSE, ANDREW** et al.... .1
Infectious Disease Education That Spans Borders (manuscript)....2
Presentation (same title) *.... 18

**Note: line drawing in presentation adapted with permission from Megli CJ and Coyne CB.Nature Rev Microbiol ,2022*

9.**FERNÁNDEZ PIRLA** et al....40
Toxoplasmosis (excerpt from presentation) 41

10. **COMMUNITY EDUCATION (MISCELLANEOUS)**
Congenital Toxoplasmosis in Panama (presentation by Mariángela Soberón Felín) ……...48
Community initiatives and presentations (list).... . 65
Cost Efficacy of Screening for congenital toxoplasmosis (by Eileen Stillwaggon) ………..68

Alba Nubia Montenegro Vásquez
with Mélida Escala, MD; Xavier Sáez Llorens, MD

HOSPITAL DEL NIÑO

REPÚBLICA DE PANAMA

TEACHING DEPARTMENT

THESIS FOR SUBSPECIALTY IN NEONATOLOGY

Topic:

Seroprevalence of *Toxoplasma gondii* infection in pregnant women as detected through anti-*T. gondii* IgG and IgM antibodies and its effect on newborns at the maternity wing of Hospital Santo Tomás between June and November 2014.

Researcher: Alba Nubia Montenegro Vásquez

Neonatology resident.

Clinical advisor: Dra. Mélida Escala

Pediatrician – Neonatology

Methods advisor: Dr. Xavier Sáez Llorens

Pediatrician – Infectologist

1 June 2014

INDEX	
	**Pg.**
ABSTRACT	1
INTRODUCTION	2
HISTORY	3
JUSTIFICATION FOR THIS STUDY	4
OBJECTIVES	5
BACKGROUND	6
METHODS	14
BUDGET	17
RESULTS	18
DISCUSSION	22
CONCLUSIONS	24
RECOMMENDATIONS	25
REFERENCES	26
APPENDICES	30

ABSTRACT

Congenital toxoplasmosis is responsible for more congenital birth defects than herpes, rubella, and syphilis combined, and it is more common and dangerous than what physicians and researchers have assumed up to now. Because of this, the few studies related to the disease in Central America have focused on weather, altitude, occupation, rural or urban living space, ethnicity, and individual habits.

Sixty percent of women tested had antibodies to *Toxoplasma*. Seventy-six of the 145 seroppositive women were of child bearing age, with 100% of 11-14 year olds and 60 % of 36-49 year olds seropositive.

Out of all of the women who were seropositive for IgG, 47 percent lived in urban areas, and 40 percent lived in rural areas.

With respect to the prevalence of IgM antibodies against *Toxoplasma gondii*, out of the 183 people in this study, eight women (2%) were positive for IgM, and 50% of the children of these mothers presented with congenital toxoplasmosis.

Introduction

The prevalence of congenital toxoplasmosis varies between and within countries. In general England had lower rates, Central and South America and Africa higher rates.(1,2, 4, 6, 11, 14-17). Transmission rates and percentages of patients with symptoms and times in life symptoms appear arepresented elsewhere in the papers in this series.

In Panama, there have been few studies on congenital toxoplasmosis. The latest study conducted was in 1988, which showed a prevalence of 8.6% (23). Currently, according to statistics from Hospital Santo Tomás, out of 16,381 births in 2013, only two pregnant women were screened for toxoplasmosis using IgM and IgG tests.

In Brazil, between September 1995 and December 1999, a country-wide screening study to detect congenital toxoplasmosis was conducted on 140,914 newborns who were between three and fifteen days old. This study used indirect immunofluorescence to detect IgG and IgM antibodies in mothers and children, and it found 47 cases of congenital toxoplasmosis, with only eight (17%) of these cases presenting clinical signs. This study was published in the Argentine Consensus of Congenital Toxoplasmosis in 2008 (21).

Spain has conducted a study with the goal of better understanding the serological diagnosis of congenital toxoplasmosis cases and antibody prevalence in pregnant women. Between 1992 and 2008, the study examined a total of 68,712 serum samples from 47,635 pregnant women within the catchment area of Hospital Miguel Servet (Zaragoza). The hospital's microbiology lab found IgA, IgM, and IgG antibodies against *Toxoplasma*; it determined that the cases of congenital toxoplasmosis were detected through maternal seroconversion during pregnancy, and that IgA was the most sensitive serologic marker for detecting congenital infection (9).

In Massachusetts and New Hampshire (United States), a study of 635,000 children used antibody tests for IgM antibodies against *T. gondii*; 100 tested positive, while 52 of these cases later had congenital infection confirmed (12).

In Colombia, in June of 2011 a study was done on 105 newborns; the main purpose of this research was to evaluate and determine the cutoff point for the detection of anti-*Toxoplasma* IgM. This study used the ELISA method on umbilical blood samples, tested two different comercial tests, and correlated the results obtained with the congenital toxoplasmosis diagnosis. A sensitivity of 100% and a specificity of 78% showed that this test could identify congenital toxoplasmosis using a sample of blood from the umbilical cord (1,19).

Epidemiological studies conducted in Cuba allow us to conservatively estimate that *T. gondii* seropositivity in pregnant women is between 51 and 75%, and that each year about 250 children are born with this parasite, even though only a small number of them present clinical evidence of infection upon birth. Seroconversion studies indicate rates of 0.2 to 2.0%, depending on the area studied (2).

In Guatemala, a study was done on toxoplasmosis prevalence in pregnant women who attended Roosevelt Maternity Hospital in March of 2006. It was found that out of 279 women studied, 195 (69.9%) presented with IgG antibodies against *Toxoplasma gondii*. In addition, two cases of IgM-positivity were found out of a random sample of 90 that had been chosen from the IgG-positive women (5).

In November 1995, Panama undertook a prospective cohort study of 500 children over 5 years; this research found a toxoplasmosis incidence rate of 12.6%. Another study, begun in March 1988 in Chorrera (a specific province of Panama), examined the prevalence of anti-*Toxoplasma* antibodies over 10 years in 326 children, from age two until age thirteen. This study found an average incidence rate of 8.6% per year; the antibody prevalence rate grew from 25% at five years old to 50% at ten years old (22, 23, 24).

Justification for this Study

Currently, the perinatal histories for mothers who go to the maternity wing at Hospital Santo Tomás lack data on consistent serological screening for toxoplasmosis. Out of 16,381 births in 2013, only two pregnant women were screened for toxoplasmosis using the recommended IgM and IgG tests. This happened despite the fact that toxoplasmosis is an infection that is responsible for more congenital defects than herpes, rubella, cytomegalovirus, and syphilis. This shows us that there is inadequate attention paid to this infection – more common and insidious than believed among physicians and researchers up to now – during pregnancy, which is itself a risk factor for congenital toxoplasmosis.

The risk and severity of fetal infection depend in great part on the moment at which the mother is infected. Because of this, it is of enormous importance that specialists who work in primary care – especially those who care for pregnant women – are familiar with the fundamental aspects of preventing and transmitting *T. gondii* infection, the clinical signs of toxoplasmosis, the immune response to the parasite, how to interpret diagnostic tests, and patient behavior.

In the Hospital del Niño, according to statistics taken over the last five years, eighteen children have left with a diagnosis of congenital toxoplasmosis. However, diagnosis is only made if the patient is born with clinical signs; we do not know how many patients who have not shown these signs at birth are infected. At birth, the majority of children with congenital toxoplasmosis are asymptomatic, but the consequences in the long term can be serious, including mental retardation, microcephaly, macrocephaly, microcephaly, cataracts, microphthalmia, glaucoma, blindness, and issues with psychomotor development. All of these symptoms negatively affect these children’s quality of life and their adult development. For this reason, it is of the utmost importance to understand the frequency of infection in pregnant women. Eventually, we hope that the results of this study will contribute to the creation of a universal screening program for pregnant women in primary care.

Objectives

General

Understand the frequency of *Toxoplasma gondii* in pregnant women who attend Hospital Santo Tomás between June and November 2014, as well as the impact of this parasite on newborns.

Specific

1. Idenfity the prevalence of *Toxoplasma gondii* in pregnant women.

2. Determine the prevalence of congenital toxoplasmosis in children who were born to *Toxoplasma*-positive women.

Background

Toxoplasmosis is a widely distributed zoonosis, caused by an organism from the Coccidia order, *Toxoplasma gondii*. This is an obligate intracellular parasite that was discovered in 1909 and named after the “gundii,” a north African rodent in which the microorganism was first detected (2,5,12,15).

“Congenital toxoplasmosis” typically comes about through a primary *T. gondii* infection during pregnancy that is later transmitted to a fetus through the placenta. However, this condition can also come about through reactivation of a previous *T. gondii* infection among pregnant women who are immunosuppressed (e.g. have AIDS, have lymphoma, are undergoing corticotherapy) (2).

Congenital toxoplasmosis has high morbidity and mortality; if not diagnosed in a timely manner, this disease can cause serious consequences for children. However, if women are diagnosed and treated during pregnancy, consequences for the fetus are diminished significantly, even allowing for normal development of the child (12, 29-32).

The infectious agent in question is capable of crossing the placental barrier and residing in embryonic or fetal tissue. This can cause various degrees of damage, leading to microscopic necrotic foci in the caruncular septa and allowing the parasite to spread to nearby tissues. Additionally, the organism leads to chorionic villi infection, which can stimulate hypertrophy and hyperplasia in affected trophoblast, desquamation of portions of the chorion, and coagulative necrosis. For this reason, some of the greatest and most destructive injuries related to toxoplasmosis appear in congenital infection (1,2,19).

Congenital toxoplasmosis has a wide variety of clinical presentations. However, we can put together four general categories: neonatal illness that shows up at birth, light or serious illness that shows in the first months of life, sequela or relapse of an undiagnosed infection during infancy or adolescence, and a subclinical infection with a serological test that confirms or shows infection (3).

In the first group, infection and its consequences are unmistakable, but the other groups show definitive sequelae. These sequelae can seriously affect the patient’s life, especially if the patient has not been diagnosed and treated on time. In this sense, it is possible that the majority of children with congenital toxoplasmosis will pass through asymptomatic and undiagnosed if they are only evaluated at birth (3).

Maternal-fetal transmission can present at any moment during pregnancy, although the rate of transmission rises with gestational age. If a mother acquires *T. gondii* during the first trimester, the risk of congenital infection is 15%; if during the second trimester, 30%; if during the third, 60%. However, the consequences for the fetus are more serious if the infection is earlier. In any case, the transmission rate can be

reduced to 5% or less if infection in mothers is detected and proper treatment is provided (2,8).

There are three main life forms in which *T. gondii* is transmitted to definitive and intermediate hosts; these are tachyzoites, tissue cysts, and oocysts. The life cycle of *T. gondii* has two other life stages, which are bradyzoites and merozoites (8).

The tachyzoite is the form of *T. gondii* that crosses the placenta and causes congenital infection; this is consistent with how tachyzoites of other parasitic infectious organisms are the active replicating life form, responsible for dissemination and tissue damage. *T. gondii* tachyzoites are also found in blood and tissue during acute infections (2).

During pregnancy, tachyzoites reach the placenta through hematogenous dissemination; they reproduce and accumulate in the chorion, decidua, and umbilical cord. If tachyzoites arrive at the fetus, they disseminate to all of its organs, including the central nervous system. Some cases of this congenital infection lead to abortion or stillbirth. Maternal-fetal transmission can occur at any moment during pregnancy, and the risk frequency tends to be greater for mothers who are infected later in pregnancy (2).

The organs of greatest clinical importance that *T. gondii* infects are the lymph nodes, skeletal muscle, myocardium, brain, retina, and placenta. In the infected tissues, tachyzoites penetrate various host cells (parenchymatous, endothelial, epithelial, and macrophage) by replicating in the cytoplasm, rupturing the cells, and continuing to infect nearby cells (2).

Only ten to fifteen percent of congenital *T. gondii* infections lead to symptoms at birth, while more than 80% of asymptomatic, infected children show serious sequelae in childhood, such as neurologic damage, toxoplasmic retinochoroiditis, and blindness (3,5,8,11).

The definitive host of *T. gondii* are felids, and intermediate hosts for this organism include all warm-blooded animals in nature, among them humans. When a cat ingests one of the forms of *T. gondii*, the parasite undergoes asexual reproduction in the host's epithelial intestinal cells, and up to millions of oocysts are later excreted. When these oocysts sporulate, they can then infect other organisms by being ingested. Sporulation is a process that requires various days and that depends on the existence of favorable – in this case, warm and humid – conditions (16).

In immunocompetent people, a *T. gondii* infection causes rapid cellular and humoral immune responses, which leads to detectable serum levels of IgM and IgG and a T cell response to *T. gondii* antigen *in vitro*. Both types of immune reaction seem to be crucial to initially controlling the proliferation of the microorganism – for this reason, nearly all people who are acutely infected with *T.*

gondii are seropositive. T cell activation takes place shortly after acute infection, in children infected ppostnatally and adults but not in all congenitally infected infants(10).

Toxoplasmosis during pregnancy

Primary infection with *Toxoplasma gondii* during pregnancy can lead to hematogenous dissemination to the placenta and transmission to the fetus through the placenta. Pregnant women who are at risk of acquiring the infection are those who are seronegative for antibodies against *T. gondii*, since this subset of women can be acutely infected during gestation – as such, their serological monitoring should be frequent. Pregnant women with serious cellular immunodepression also run the risk of acquiring this infection, regardless of their serology (16).

The consequences of maternal infection for a fetus are determined by the gestational age at which infection takes place. There are three general stages of gestational age and corresponding sequelae: irreversible sequelae, acute encephalitis, and generalized infection (3,6).

"Generalized infection" takes place when primary infection takes place during the third trimester of pregnancy, a gestational period in which fetal risk of acquiring infection is 80 to 90 percent (2-8,13). The child shows symptoms similar to acute sepsis, such as fever, jaundice, hepatosplenomegaly, and in several cases myocarditis or interstitial pneumonia. Exanthema is not present, and neurological complications are rarely seen (16,19).

"Acute encephalitis" presents when a primary infection takes place during the second trimester of pregnancy, a gestational period in which fetal risk of acquiring infection is 30%. In benign cases, the child can have a normal weight and present few clinical manifestations, but later on he becomes apathetic, refuses food, and can even have seizures. In serious cases the newborn shows hydrocephaly, retinochoroiditis, and cerebral calcifications (together known as Sabin's Triad), along with mental retardation (19,20).

Diagnosis

Methods used for diagnosis are different in distinct clinical situations: infection acquired in an immunocompetent host, in an immunodeficient host, or congenital infection. Diagnostic methods can be direct or indirect (9, 20).

Direct methods

Direct methods of diagnosing toxoplasmosis are based on detection of the parasite in blood, organic liquids, or tissues. However, detection is also possible using histological techniques, which involve isolation in cell cultures or inoculating mice (9,16,20).

The polymerase chain reaction method (PCR) can detect the deoxyribonucleic acid (DNA) of *T. gondii* in tissues and bodily fluids. When this method is applied to tissues (where cysts can reside), it is impossible to distinguish latent from active infections. However, PCR is valid for studying blood, amniotic fluid, or LCR, where there are no cysts (9,20).

Indirect methods

Indirect screening methods mainly depend on serological studies of patients. The interpretation of these tests should be done with caution, due to the very high prevalence of asymptomatic infection (28). Among these indirect methods are:

Sabin-Feldman: reference test that is currently conducted only in specialized labs due to its complexity and cost – in particular, the need for live parasites. This method detects anti-*Toxoplasma* antibodies in serum with methylene blue stain, which strongly stains *T. gondii* cells (28).

Indirect immunofluorescence (IIF): using antibodies marked with fluorescent agents, this detects specific antibodies present in serum that attach to the surface of the parasite. It can measure levels of IgM and IgG (27,28).

Enzyme-linked immunoabsorption assay (ELISA): A method that is more specific and sensitive than IIF or detecting IgM, since the former presents neither false positives for acquired and congenital toxoplasmosis nor false negatives for congenital toxoplasmosis. Given the complex structure of *Toxoplasma gondii*, it is better to use polyclonal antibodies. Finding IgM with the ELISA method can lead to false positives triggered by rheumatic factor. Acute toxoplasmosis is indicated by the presence of circulating antigens and antigens present as immune complexes in 100% of infected individuals. Acute infection is also indicated by elevated concentrations of IgM and IgG, which are only detectable at low concentrations in subacute toxoplasmosis (27,28).

Direct agglutination: this method measures total amounts of IgG and IgM against the parasite. It is easy to carry out and has good sensitivity, both for diagnosis and for follow-ups with pregnant women. If antibodies are not present, parasites form a button-shaped sediment; agglutination is positive if a lattice shape is formed (27).

Immunoglobulin-M immunosorbent agglutination (ISAGA): can detect IgM and IgA antibodies. Shows good sensitivity and specificity (27,28).

Indirect hemagglutination (IHA): detects IgG, present in sensitized red blood cells. Positive results occur later on. Not recommended for researching acute infection (28).

Diagnosing congenital infection

The definitive diagnosis of congenital infection *in utero* is made by isolating parasite from amniotic fluid,. Earlier study of fetal blood was used, and finding specific IgM in fetal blood, or by isolation of the pparasite but this is not used any longer. Obtaining a positive result for *T*.gondii DNA in a PCR test of amniotic fluid establishes the diagnosis(8,47). The PCR test is sensitive, specific, It is done after 15 weeks of ammennhorhea for acutely infected pregnant women (26).

Ultrasounds show whether embryofetopathies or placentomegaly exist; these should be conducted once a month after detection of acute maternal infection. After birth, the placenta of every woman who has received treatment for toxoplasmosis should be evaluated using microbiology and pathology studies (26).

Fetal infection should be sought every time maternal acute infection is detected during the course of pregnancy. Conventional methods include clinical evalution at birth with general examination, retina examination, brain ctomputed tomography scan or mri, lumbar puncture measuring cells, protein and glucose, total serum IgM in mgm/dl, seum evaluation in order to demonstrate the presence of *Toxoplasma gond*ii specific IgM and IgA,, decrease in platelet levels, rise in white cells, atypical lymphocytes or eosinophils, rise in direct bilirubin, transaminases, gamma-GT, and lactate dehydrogenase, and possibility of identifying the parasite or parasite DNA in whole blood,cerebrospinal fluid or placenta(27).

We can establish diagnosis in a child by confirming that detected antibodies belong to the child, and are not the product of transplacental movement of maternal antibodies. To establish this diagnosis it is necessary to:

a. Create an IgG antibody curve in the first months of the patient's life.

b. Demonstrate the presence of specific IgM or IgA antibodies in the first weeks or months of life (28).

Maternal IgG antibodies are transferred from mother to fetus, since they cross the blood-placental barrier. In non-infected newborns, these antibodies progressively go away and are fully gone by six to twelve months. In a newborn with congenital

toxoplasmosis, the titer of anti-*T. gondii* IgG can progressively grow and, in any case, these antibodies persist at a detectable level beyond 12 months of life (25).

Avidity of IgG Antibodies

Refers to the particular strength of the binding between antigen and antibody in acute and chronic infection, as described by Hedman et al. in 1989. Several studies show that for the first 20 weeks post-infection, low-avidity IgG antibodies predominate, which strongly suggests acute infection. Meanwhile, if high-avidity IgG antibodies dominate, this would be indicative of a past infection (25).

An IgM-positive result in a newborn is diagnostic of congenital toxoplasmosis, since this antibody's high molecular weight prevents it from crossing the placenta. It is recommended that ISAGA or double-capture ELISA be used to evaluate IgM levels (27).

If IgM turns out negative at birth, follow-up IgG testing should be done at three to six months, given that the absence of IgM does not rule out congenital infection. In fact, a significant number of infected infants do not develop detectable levels of IgM. If IgG is positive at six months, we are dealing with congenital toxoplasmosis, and if an infant is negative the case is dismissed (20. 27,28).

IgG	IgM	How to interpret serological results/Not applicable to infants
Negative	Negative	No serological evidence of toxoplasmosis infection
Negative	Unclear	Possible acute infection or false positive IgM. Need to take another sample for an IgG/IgM test. If results are the same, it is likely that the patient is not infected with *Toxoplasma*.
Negative	Positive	Possible acute infection or false positive IgM. Need to take another sample for an IgG/IgM test. If results are the same, it is likely that the IgM reaction was a false positive.
Unclear	Negative	Impossible to determine. Need to take a new sample or examine the current sample again for IgG/IgM.
Unclear	Unclear	Impossible to determine. Need to take a new sample for an IgG/IgM test.
Unclear	Positive	Possible acute *Toxoplasma* infection. Need to take a new sample for an IgG/IgM test. If results are the same, or if IgG appears positive, both samples should be sent to a reference laboratory with experience in diagnosing toxoplasmosis for additional testing.
Positive	Negative	*Toxoplasma* infection more than one year ago.
Positive	Unclear	Either *Toxoplasma* infection more than one year ago, or IgM false positive. Need to take a new sample for an IgM test. If results are the same, both samples should be sent to a reference laboratory with experience in diagnosing toxoplasmosis for additional testing.
Positive	Positive	Possible recent infection within the last twelve months, or IgM false positive. Need to send sample to a reference laboratory with experience in diagnosing toxoplasmosis for additional testing.

Uribarren T. Toxoplasmosis. Diagnostic findings. Modified from: Centers for Disease Control and Prevention. (27,28)

Interpretation scheme for various stages of infection

Infection	Serologic tests				
	SF	IIF	ELISA IgG	IIF/ELISA IgM, IgA (DS)	ISAGA IgM, IgA
Acute	+++	++	+++	+	+++
Congenital	++	+	++	+/-	++/-
Chronic	+	+	+	-	-

(+) Low titers (1:16-1:512 or 10-100 UI)
(++) Medium titers (1:1000-1:4000 or 100-300 UI)
(+++) High titers (greater than 1:4000 or greater than 300 UI)
(+/-) group of non-reactive patients
(-) non-reactive
SF: Sabin-Feldman
IIF: Indirect immunofluorescence
DS: Double sandwich
Tomado de Arias EA. “Toxoplasmosis en la embarazada”. 2002. 19 May 2003

Treatment

	Medication	Dose	Time
First choice	Pyrimethamine	2mg/kg/day first day; continue with 1mg/kg/day one or two daily doses by mouth	1 year
	Sulfadiazine	100 mg/kg/day two daily doses by mouth	1 year
Second choice	Pyramethamine	1mg/kg/day once a day by mouth	1 year
	Clindamycin	20mg/kg/day IM o IV three times a day in acute phase, later by mouth	1 year
Folinic acid		5-10 mg/ dose 3 times a year	1 year

MEDICINA (Buenos Aires) 2008; 68: 75-87, CONSENSO Internacional DE TOXOPLASMOSIS CONGENITA

Methods

Type of study: cross-sectional, descriptive epidemiological study.

Population: all children born between June and November 2014 in the Maternity Wing of Hospital Santo Tomás.

Sample: A random sample was taken out of an estimated population of 7920 children. Estimate was made based on an average of 1320 monthly births from January to December 2013. To pick the finite sample, the following formula was used: n=Z2 Npq/e2(N-1)+Z2 pq. Out of 366 children numbered 1 to 366, one sample was taken every 21.6 births. Twenty additional samples were taken, in case the mothers who were selected did not want to participate in the study.

Inclusion criteria:

a) Birth in the maternity wing at Hospital Santo Tomás.

b) Birth between June and November 2014.

c) Fulfills the menstrual cycle.

Exclusion criteria:

a) Birth that fulfills inclusion criteria and is not authorized by the mother.

b) Stillbirth.

Information collection:

Type of information: primary

The investigator filled a spreadsheet with each subject's data once informed consent was obtained. Tubes used to hold samples were labeled sequentially from 1 to 366; one sample was taken for every 21 births. When maternal IgM serologic tests were found positive, the child received external follow-ups from Dr. Mélida Escala Brito, neonatologist from the hospital unit.

Sample taking and detection of IgG/IgM antibodies against *T. gondii* through immunoassay. (ARCHITECT TOXO IgM and IgG)

- Puncture site was chosen between median cubital and middle cephalic veins.
- Tourniquet was tied on subject arm, and area for puncture was disinfected with cotton and alcohol.
- Three milliliters of blood were taken from each mother using extraction tubes previously labeled with the corresponding sample numbers.
- Samples were transported to the Hospital del Niño clinical laboratory and were analyzed using the ARCHITECT TOXO IgM/IgG assay, which is a two-step immunoassay for qualitative detection of both IgM and IgG antibodies for *Toxoplasma gondii* in human serum and plasma. The test uses flexible assay protocols known as Chimiflex.
- Serum extracted was put in previously labeled vials. When combined with monoclonal IgM antibodies, antigen in the samples caused complexes to form.
- Two conjugated complex washes were carried out. Mouse monoclonal anti-toxo p30 antibodies were marked with acridinium. Next, two native fragments and *Toxoplasma gondii* lysate, which contains p30 antigen, were added. The antigen in question can lead to a second step reaction involving formation of an antibody-antibody-conjugate complex.
- The second wash added pre-trigger solutions, which caused a chemoluminescent reaction (measured in relative light units, using the ARCHITECT System optical measurement).
- Presence or absence of anti-TOXO IgM and IgG in a sample was determined with the chemoluminescence signal relative to previous calibration.
- If the chemoluminescent signal in samples was greater or equal to the cutoff, the sample was considered “reactive” for anti-TOXO IgG/IgM antibodies.
- Samples were read every week by Dr. José Racine; on weekends they were processed and stored by serology personnel.

Reactants:

Bottle # 1: Macroparticles: Anti-human IgM and IgG anti-human (mouse, monoclonal) with protein stabilizer and detergent.

Bottle # 2: conjugate with acridinium-marked anti-*Toxoplasma* p30 antigen (monoclonal, mouse), antibodies, and native *Toxoplasma* lysate, on a phosphate pad with protein stabilizer and detergents.

Analysis:

The team's results are "negative" and "positive."

Value for a sample that is "positive" for IgM/IgG antibodies against *Toxoplasma gondii*: between 0.60 and 1.00 UI/mL

Value for a sample that is "negative" for IgM/IgG antibodies against *Toxoplasma gondii*: less than 0.50 UI/mL.

Value for an "indeterminate" result: between 0.50 and 0.60 UI/mL.

Quality control

- The control requirement for the ARCHITECT *Toxoplasma gondii* IgM/IgG assay was analysis of a single sample of each of the controls every 24 hours.
- Modular system was regularly reviewed.
- Samples had to be free of fibrin, red blood cells, or other particles. If any of this was present, samples were centrifuged for ten minutes at 10,000 rpm.
- Use of samples that were preheated, lipemic, or contaminated by bacteria was prohibited.

Analysis of results: a spreadsheet was used to collect data in Epi-Info. Results were analyzed in this program; variable crossing was done in accordance with the interests of the study.

Variables

Variable	Description	Range
Age	Time since live birth	11-14 15-19<br>20-25 26-30<br>31-35 36-49
Birthplace	Place of origin	Urban Rural
Province	Current providence of residence	Panamá Coclé<br>Veraguas Herrera<br>Los Santos Bocas del Toro<br>Colon Chiriquí<br>Darién<br>Comarca Guna Yala<br>Ngobe Bugle<br>Embera
Positive	Positive lab report	Low titer: 1:16 1:512 10-100UI<br>Medium titer: 1:1000 1:4000 o 100-300UI<br>High titer:>1:4000 o >300UI
Negative	Negative lab report	
Symptoms	Clinical signs that suggest *Toxoplasma gondii* infection	Hydrocephaly, microcephaly, chorioretinitis, cerebral calcifications, hepatosplenomegaly, jaundice, seizures.

Budget

Material	Number	Unit price	Total
Anti-*T. gondii* IgG reagent kits	8 bottles	300 dollars	2400 dollars
Anti-*T. gondii* IgM reagent kits	8 bottles	300 dollars	2400 dollars
Capillary tubes	732	5 dollars	3660 dollars
Multisample finger pricks (21 x 1.5)	800	0.1	80 dollars
Cotton	2 packages of 500 units each	5 dollars	10 dollars
Paid work (sampling)	Six months	30 dollars/week	720 dollars
Alcohol	2 boxes of 500 units each	5 dollars	10 dollars
Photocopies	3000 copies	Five cents	150 dollars
Reams of paper	5 of 500 sheets each	2 dollars	10 dollars
Total			9440 dollars

Results

This study was conducted between June and November 2014 on a sample of 383 pregnant women who were attended in the maternity wing of Hospital Santo Tomás in Ciudad de Panamá, Panama.

Out of the 383 pregnant women studied, 92% (353) were born in urban areas, while 8% (30) were born in rural areas. Out of the mothers sampled, 51.5% (193) showed IgG antibodies against *Toxoplasma gondii*, while 48.5% (190) did not present this. Out of the women who were positive for IgG, 94% (181) were urban and 6% (12) were rural; see tables 1 and 2.

Table 1. Birthplace for women who were attended at the maternity wing of Hospital Santo Tomás and who participated in this study. From June to November 2014.

Origin	Samples	IgG (+)
	Number	Percent
Urban	353	92
Rural	30	8
Total	383	100

Table 2. Anti-*Toxoplasma gondii* IgG serology relative to birthplace.

Origin	Samples		
	Total number	IgG +	Percent of total
Urban	353	181	51
Rural	30	12	40
Total	383	193	50

When it comes to presence of IgM antibodies against *Toxoplasma gondii*, 2% (8) of the pregnant women sampled were positive, all coming from urban areas.

Table 3. Anti-*Toxoplasma gondii* IgM serology relative to birthplace.

Origin	Samples		
	Total number	IgM +	Percent of total
Urban	353	8	2
Rural	30	0	0
Total	383	8	2

In pre-established age groups, the percent of mothers positive for IgG were: 100% of mothers 11-14 years old (3), 47% of mothers 15-19 years old (36), 51% of mothers 20-25 years old (76), 48% of mothers 26-30 years old (45), 52% of mothers 31-35 years old (24), and 60% of mothers 36-49 years old (9).

Table 4. Age distribution of IgG positive mothers.

Age	Samples		
	Total number	IgG +	Percent of total
11-14	3	3	100
15-19	76	36	47
20-25	149	76	51
26-30	94	45	48
31-35	46	24	52
36-49	15	9	60
Total	383	193	50

The distribution of IgM-positive mothers by age is below.

Cuadro No 5. Age distribution of IgM positive mothers.

Age	Samples		
	Total number	IgM+	Percent of total
11-14	3	1	33
15-19	76	2	3
20-25	149	3	2
26-30	94	1	1
31-35	46	0	0
36-49	15	1	7
Total	383	8	2

With respect to current place of residence, the majority of IgG-positive women came from Darién and Cuna Yala, Ciudad de Panamá, Veraguas, and Herrera.

Table 6. "Current place of residence" distribution of IgG-positive mothers.

Province	Samples		
	Total number	IgG+	Percent of total
Panamá	370	187	51
Veraguas	1	0	0
Colón	3	1	33
Coclé	2	1	50
Darién	2	2	100
Cuna Yala	1	1	100
Herrera	1	0	0
Los Santos	3	1	33
Total	383	193	50

The distribution of IgM-positive mothers by current place of residence is below.

Cuadro No 7. "Current place of residence" distribution of IgM-positive mothers.

Province	Samples		
	Total number	IgM+	Percent of total
Panamá	370	7	2
Veraguas	1	0	0
Colón	3	0	0

Coclé	2	0	0
Darién	2	1	50
Cuna Yala	1	0	0
Herrera	1	0	0
Los Santos	3	0	0
Total	383	8	2

Fifty percent of the children of IgM-positive mothers had congenital toxoplasmosis, but they were also asymptomatic at birth.

Table 8. Frequency of congenital toxoplasmosis in children of IgM-positive mothers.

Origin		IgM+ Total number	Percent of total
	Infant	Mother	%
Urban	4	8	50
Rural	0	0	0
Total	4	8	50

Discussion

Congenital toxoplasmosis is a disease with high worldwide morbidity and mortality, and it has serious consequences for children who are not diagnosed in a timely manner.

Our study determined the frequency of *Toxoplasma gondii* in a sample of 383 pregnant women who were attended in the maternity wing of Hospital Santo Tomás between June and November 2014. Immunoassay (ARCHITECT Toxo IgG/IgM) was used to find antibodies against *Toxoplasma gondii*.

Out of the 383 pregnant women studies, 92% (353) were born in urban areas and 8% in rural areas (8%). This high urban proportion is significant, given that the Santo Tomás is responsible for all institutional hospital births in Ciudad de Panamá; it tends to only see rural pregnant women who have serious complications. It was found that 51% (193) of women tested showed anti-*Toxoplasma gondii* IgG antibodies, 94% of whom were born in urban areas and 6% of whom were born in rural areas.

The high frequency of IgG-positive results found in this study helps us argue that toxoplasmosis is endemic in Panama, likely because the country is tropical and has inadequate hygienic and sanitary conditions. This study illustrates the importance of screening for antibodies in pregnant women and of putting together a screening routine. In order to promptly diagnose to treat to prevent congenital toxoplasmosis and for education of pregnant women, it is necessary to identify seronegative pregnant women, the subset of patients at risk of passing the parasite on to their child if infected during gestation. It is important to regularly screen these women for acute infection and to start early treatment in order to avoid serious consequences for newborns.

Panama has a greater frequency of toxoplasmosis than do other countries that have done studies like this one. These other countries include Denmark (27.4% seroprevalence), Finland (20.3%), and the United Kingdom (7.7%).

Countries such as Cuba and Guatemala report a prevalence between 51 and 69 percent, which is similar to the level found in our study.

The province with highest IgG frequency was Ciudad de Panamá; this may be due to the location of the study.

Importantly, 40% (12) of the 30 pregnant women from rural areas were seropositive for IgG. This number is consistent with medical literature that

describes toxoplasmosis as an illness more frequent in rural areas, where soil is warmer and women more frequently get in contact with cat feces through activities such as farming and gardening.

The age group of 11-14 year olds was very small but does highlight likely demographics of young teenagers who are giving birth in Panama'. Although only 3 such teenagers were identified, this was an age group with highest infection prevalence. 100% of mothers 11-14 years old (3 people total). In the whole group 51% of mothers 20-25 years old were seropositive (76). This second age group is most commonly pregnant in Panama'. There was an increase in seropositivity in the 36-49 years old age group, to 60% which reflects the increasing seroprevalence with age in other studies as well (9). A similar result was reported in a Guatemalan study on seroprevalence of *Toxoplasma gondii* in pregnant women, a study that showed a seropositivity maximum in women between 31 and 35 years old. It has been shown that as women get older, the likely time of exposure to the parasite and rate of seroconversion also increase. (2,16).

The 3(100%) of 3 teenagers between 11 and 14 years old who were IgG-positive; with one positive for IgM antibodies might suggest that special consideration of who these young pregnant women are and how they might become infected could be meaningful in evaluation of exposures and risk factors. There are no studies with similar findings, nor any existing literature, on this subject.

With respect to *T. gondii*'s impact on newborns whose mothers were IgM-positive (8), 50% of these children (4) were IgM-positive, but did not present clinical signs of toxoplasmosis at birth. This is consistent with most of the literature, which states that only ten to fifteen percent of congenitally infected children present symptoms at birth. Most congenitally infected children show symptoms later, either in the first months of life or even during infancy or adolescence.

Compared to what other epidemiological studies say on *Toxoplasma gondii* seroprevalence in pregnant women, Guatemala, Cuba, Denmark, and Colombia report lower rates than our study does. Our seroprevalence rate was exceeded only by that of a study done on 635,000 children in Massachusetts and New Hampshire (United States), all of whom underwent a test for anti-*T. gondii* IgM antibodies. Out of these children, 100 were positive, and congenital infection was later confirmed in 52 of them.

Conclusions

1. Frequency of IgG antibodies against *Toxoplasma gondii* in pregnant women who were attended in the maternity wing of Hospital Santo Tomás was 51.5%.

2. The majority of women in the study and of those with IgG/IgM antibodies against *Toxoplasma gondii* were originally from an urban area.

3. In terms of age, there is a general linear increase in seropositive rates as age increases; that 3of 3 young teenagers were seropositive and one had IgM suggests that further study of this demographic in Panama might lead to identificationof risk factors or exposures,but the numbers are too small herein to draw conclusions, and would require further study.

4. From the 383 pregnant women studied, 2% (8) were positive for IgM antibodies. Out of the children of these women, 50% (4) had congenital toxoplasmosis, but all were asymptomatic at birth.

Recommendations

For the Ministry of Health

1. Establish routine screening for *Toxoplasma* in pregnant women, starting at the beginning of pregnancy. This allows for timely and specific treatment in case of a seroconversion and can protect against serious effects for the children of infected mothers.

For the Information, Education, and Communication promotion department

2. Promoting sanitary and hygienic habits with respect to activities such as handling cat feces and gardening.

For the maternity wing at Hospital Santo Tomás

3. ,All age groups of pregnant women should be screened for acquisition of *T.gondii* during gestation. In this high prevalence country screening of pre-pregnant women could be beneficial. Such screening could help to identify those infected pre-conception, and those who are seronegative and thus at risk of sero-conversion during gestation. This could allow identification of seroconverting women by monthly screening for acquisition of antibodies to *T.gondii.*

Bibliography

1. Torres, Elizabeth; Osorio, Elkin; Núñez, Lilian; Chacón, Leonor; Castaño, María Eugenia; Rivera, Raúl; Validación de pruebas ELISA IgM anti-Toxoplasma para uso en programas de tamización en recién nacidos / June 2011.

2. Martín Hernández, I. “Toxoplasmosis congénita: una mirada al problema” Rev. Biomed June-September 2004; 15:181-190.

3. VHI Sida en Chile. “Infecciones oportunistas” 2003

4. González González NL, *et al*. Programas de prevención de la toxoplasmosis congénita. 2003

5. Pamela Johana Sambrano Bonilla, Prevalencia de Toxoplasmosis en mujeres embarazadas que asisten a la maternidad del Hospital Roosevelt Guatemala Marzo 2006.

6. Ivonne Martin Hernández Toxoplasmosis congénita: Una mirada al problema, Rev Biomed 2004; 15: 181- 190.

7. Jiménez Monroy P “Guías de Manejo de la Toxoplasmosis en el embarazo” 19 May 2006.

8. Desmonts G, et al Colombia Toxoplasmosis 2003.

9. Braselli A, “Toxoplasmosis”19 May 2004.

10. Gangneux FR, et al. “Value of Prenatal Diagnosis and Early Postnatal Diagnosis of congenital toxoplasmosis. Retrospective study of 110 cases. J clin Microb, 24 July 2003.

11. Suzuki L “ Evaluation of serological markers for the inmunodiagnosis of acute adquired Toxoplasmosis” J Med Microb 2005, 50; 62-70

12. Guerina N, Et al " Neonatal Serologic Screening and Early Tratment for congenital Toxoplasma gondii infection" New Eng J Med 23 July 2003.

13. Pinon JM at al," Early neonatal Diagnosis of congenital Toxoplasmosis: value of comparative Enzyme-Linked Inmunofiltration Assay Inmunologicl Profiles and Anti-*Toxoplasma gondii* Inmunoglobulin M (IgM) or IgA Inmunocapture and Implications for Postnatal Therapeutic Strategies" 24 July 2005.

14. Sánchez A, Martin I, García SM Estudio de reactividad a *Toxoplasma gondii* en embarazadas de las provincias Ciudad de la Habana y pinar del Rio Cuba. Bioquímica 2003.

15. Siicsalud. "Epidemiología de la Toxoplasmosis de transmisión materno fetal" julio 2006.

16. Pizzi HL. Toxoplasmosis. Argentina: Rhone Toulé Royer, 80 p. July 2007.

17. Controlled trials in Cochrane Pregnancy and Childbirth (November 2007, CENTRAL (*The Cochrane Library* 2007, number 3), MEDLINE (1966 to November 2007), EMBASE (1980 to November 2007), CINAHL (1982 to November 2007), LILACS (1982 to November 2007) IMEMR (1984 to November 2007).

18. Bulletin from the Pan-American Sanitary Bureau, Toxoplasmosis como causa de enfermedad humana: OMS. 2009.

19. Fernando Roso, Alejandro Agudelo, Angela Izasa,Jose Gilberto Montoya, Toxoplasmosis congénita Aspectos clínicos y epidemiológicos de la infección durante el embarazo, Colombia medica, Vol 38 No 3, July-September 2007.

20. María Lourdes Roc, María Pilar Palacián, Elena Lomba, María Luisa Monforte, Victor Rebaje, María José Revillo, Serologic diagnosis of congenital toxoplasmosis Servicio de Microbiología, Hospital Universitario Miguel Servet, Zaragoza, España Servicio de Pediatría, October 2010.

21. Ricardo Durlach, Federico Kaufer, Liliana Carral, Cristina Freuler, Mariana Ceriotto, Marcelo Rodríguez, Héctor Freilij, Jaime Altcheh, Liliana Vázquez,

Rosana Corazza, Edgardo Sturba, consenso Argentino de toxoplasmosis congénita January-February 2008.

22. Etheredge GD, Frenkel JK. Human Toxoplasma infection in Kuna and Embera children in the Bayano and San Blas, eastern Panamá 1995; Nov; 53(5):448-57.

23. Sousa OE, Saenz RE, Frenkel JR. Toxoplasmosis in Panamá: a 10-year stady. 1988Mar; 38(2):315-22.

24. Frankel JK, Hassenein KM, Hassenein RS, Brown E, Thulliez P, Quintero-Nuñez R; Transmission of Toxoplasma gondii in Panama city, Panama: a five-year prospective cohort study of children, cats, rodents, birds, and soil, 1995 Nov; 53(5): 458-68.

25. FACHADO, Alberto, FONTE, Luis, ALBERTI, Esteban et al. Usefulness of the detection of Toxoplasma gondii antigens in AIDS patients. October 2007.

26. Holec-Gasior L. Toxoplasma gondii recombinant antigens as tools for serodiagnosis of human toxoplasmosis - the current status of studies Clin Vaccine Immunol. June 2013.

27. Ferreira AI, De Mattos CC, Frederico FB, Meira CS, Almeida GC, Nakashima F, Bernardo CR, Pereira-Chioccola VL, De Mattos LC. Risk factors for ocular toxoplasmosis in Brazil. March 2013, latest edition 27 August 2013.

28. Alvarado-Esquivel C, Sánchez-Okrucky R, Dubey JP. Serological evidence of Toxoplasma gondii infection in captive marine mammals in Mexico. Vet Parasitol 23 March 2012. Latest edition 27 August 2013.

29. McAuley J, Boyer KM, Patel D, Mets M, Swisher C, Roizen N, Wolters C, Stein L, Stein M, Schey W, Remington J, Meier P, Johnson D,

Heydemann P, Holfels E, Withers S, Mack D, Brown C, Patton D, McLeod R. Early and longitudinal evaluations of treated infants and children and untreated historical patients with congenital toxoplasmosis: the Chicago Collaborative Treatment Trial. Clin Infect Dis. 1994;18(1):38-72. PMID 8054436.

30. McLeod R, Boyer K, Karrison T, Kasza K, Swisher C, Roizen N, Jalbrzikowski J, Remington J, Heydemann P, Noble AG, Mets M, Holfels E, Withers S, Latkany P, Meier P. Outcome of treatment for congenital toxoplasmosis, 1981-2004: the National Collaborative Chicago-Based, Congenital Toxoplasmosis Study. *Clin Infect Dis.* 2006;42:1383-94. PMID 16619149.

31. McLeod R, Boyer K, Lee D. Mui E, Wroblewski K, Karrison T, Noble AG, Withers S, Swisher CN, Heydemann PT, Sautter M, Babiarz J, Rabiah P, Meier P, Grigg M. Prematurity & Severity Associate with *T. gondii* Alleles (NCCCTS, 1981-2009). *Clin Infect Dis.* 2012;54:1595-1605. PMID 22499837.

32. Remington JS, McLeod R, Thulliez, P, Desmonts G. Toxoplasmosis. In *Infectious Diseases of the Fetus and Newborn Infant*, 7th Ed. J Remington, J Klein (Eds). Philadelphia, WB Saunders, 2011.

Finite Sample Calculations

Formula	n= Z2 Npq/e2(N - 1) +Z2 pq				
Sample	n	?	Results		
Population	N	7920	Z2Npq	7606.368	
Reliability	Z	1,96	e2(N - 1)	19.7975	
Sample error	e	0.05	e2(N - 1)+Z2pq	20.7579	
			Total number of births to sample		
Success probability	p	0.5			
Failure probability	q	0.5	n =	366	

Formula

pq	0.25
Z2	3.8416
Npq	1980
e2	0.0025
N -1	7919
Z2pq	0.9604

I

Muestra	Nacimiento	Muestra	Nacimiento	Muestra	Nacimiento	Muestra	Nacimiento	Muestra	Nacimiento	Muestra	Nacimiento
1	1	17	347	33	693	49	1038	65	1384	81	1730
2	23	18	368	34	714	50	1060	66	1406	82	1752
3	44	19	390	35	736	51	1082	67	1428	83	1773
4	66	20	412	36	757	52	1103	68	1449	84	1795
5	87	21	433	37	779	53	1125	69	1471	85	1817
6	109	22	455	38	801	54	1147	70	1492	86	1838
7	131	23	477	39	822	55	1168	71	1514	87	1860
8	152	24	498	40	844	56	1190	72	1536	88	1881
9	174	25	520	41	866	57	1211	73	1557	89	1903
10	196	26	541	42	887	58	1233	74	1579	90	1925
11	217	27	563	43	909	59	1255	75	1600	91	1946
12	239	28	585	44	930	60	1276	76	1622	92	1968
13	260	29	606	45	952	61	1298	77	1644	93	1989
14	282	30	628	46	974	62	1319	78	1665	94	2011
15	304	31	649	47	995	63	1341	79	1687	95	2033
16	325	32	671	48	1017	64	1363	80	1708	96	2054

Muestra	Nacimiento	Muestra	Nacimiento	Muestra	Nacimiento	Muestra	Nacimiento	Muestra	Nacimiento	Muestra	Nacimiento
97	2076	113	2422	129	2768	145	3113	161	3459	177	3805
98	2098	114	2443	130	2789	146	3135	162	3481	178	3827
99	2119	115	2465	131	2811	147	3157	163	3502	179	3848
100	2141	116	2487	132	2832	148	3178	164	3524	180	3870
101	2162	117	2508	133	2854	149	3200	165	3546	181	3891
102	2184	118	2530	134	2876	150	3221	166	3567	182	3913
103	2206	119	2551	135	2897	151	3243	167	3589	183	3935
104	2227	120	2573	136	2919	152	3265	168	3611	184	3956
105	2249	121	2595	137	2940	153	3286	169	3632	185	3978
106	2270	122	2616	138	2962	154	3308	170	3654	186	4000
107	2292	123	2638	139	2984	155	3330	171	3675	187	4021
108	2314	124	2659	140	3005	156	3351	172	3697	188	4043
109	2335	125	2681	141	3027	157	3373	173	3719	189	4064
110	2357	126	2703	142	3049	158	3394	174	3740	190	4086
111	2379	127	2724	143	3070	159	3416	175	3762	191	4108
112	2400	128	2746	144	3092	160	3438	176	3783	192	4129

Muestra	Nacimiento	Muestra	Nacimiento	Muestra	Nacimiento	Muestra	Nacimiento	Muestra	Nacimiento
193	4151	209	4497	225	4842	241	5188	257	5534
194	4172	210	4518	226	4864	242	5210	258	5556
195	4194	211	4540	227	4886	243	5232	259	5577
196	4216	212	4562	228	4907	244	5253	260	5599
197	4237	213	4583	229	4929	245	5275	261	5621
198	4259	214	4605	230	4951	246	5296	262	5642
199	4281	215	4626	231	4972	247	5318	263	5664
200	4302	216	4648	232	4994	248	5340	264	5685
201	4324	217	4670	233	5015	249	5361	265	5707
202	4345	218	4691	234	5037	250	5383	266	5729
203	4367	219	4713	235	5059	251	5404	267	5750
204	4389	220	4734	236	5080	252	5426	268	5772
205	4410	221	4756	237	5102	253	5448	269	5794
206	4432	222	4778	238	5123	254	5469	270	5815
207	4453	223	4799	239	5145	255	5491	271	5837
208	4475	224	4821	240	5167	256	5513	272	5858

Muestra	Nacimiento	Muestra	Nacimiento	Muestra	acimiento	Muestra	acimiento	Muestra	acimient	Muestra	acimient
273	5880	289	6226	305	6572	321	6917	337	7263	353	7609
274	5902	290	6247	306	6593	322	6939	338	7285	354	7631
275	5923	291	6269	307	6615	323	6961	339	7306	355	7652
276	5945	292	6291	308	6636	324	6982	340	7328	356	7674
277	5966	293	6312	309	6658	325	7004	341	7350	357	7696
278	5988	294	6334	310	6680	326	7025	342	7371	358	7717
279	6010	295	6355	311	6701	327	7047	343	7393	359	7739
280	6031	296	6377	312	6723	328	7069	344	7415	360	7760
281	6053	297	6399	313	6745	329	7090	345	7436	361	7782
282	6074	298	6420	314	6766	330	7112	346	7458	362	7804
283	6096	299	6442	315	6788	331	7134	347	7479	363	7825
284	6118	300	6464	316	6809	332	7155	348	7501	364	7847
285	6139	301	6485	317	6831	333	7177	349	7523	365	7868
286	6161	302	6507	318	6853	334	7198	350	7544	366	7890
287	6183	303	6528	319	6874	335	7220	351	7566		
288	6204	304	6550	320	6896	336	7242	352	7587		

HOSPITAL DEL NIÑO

REPÚBLICA DE PANAMA

DEPARTAMENTO DE DOCENCIA

Formulario de Consentimiento Informado

Estudio de investigación descriptivo corte transversal sobre Seroprevalencia de infección de Toxoplasma gondii en mujeres embarazadas por medio de detección de anticuerpos IgG e IgM anti T. gondii y su impacto en el recién nacido, que asisten a la Maternidad del Hospital Santo Tomás en el periodo de Junio – Noviembre 2014.

Investigadora principal:

Alba Nubia Montenegro Vásquez

Residente de Neonatología.

Teléfono: 62694394

Información específica del centro:

Hospital del Niño

Departamento de Neonatología

Avenida Balboa, Panamá

Teléfono: 225-1546 Ext. 230/219

¿Qué es el estudio de Seroprevalencia de la Toxoplasmosis congénita?

Este estudio de investigación está siendo conducido por un grupo de asesores el cual será realizado en el Hospital Santo Tomás ya que las muestras de sangre serán extraídas a usted en la sala donde este ingresada.

Usted está invitada a participar de manera voluntaria en el estudio el cual trata sobre la prueba de sangre que se le extraerá 3 ml (media cucharadita), la cual será examinada para identificar infección del agente Toxoplasma gondii. (Parasito que es el responsable de la toxoplasmosis congénita).

Este agente es capaz de atravesar la barrera placentaria (membrana que es fuente de alimentación y protección al bebe) y alojarse en los tejidos embrionarios o fetales causando daños de variada magnitud como son: hidrocefalia(aumento del tamaño de la cabeza), retinocoroiditis (Inflamación de la retina) calcificaciones cerebrales, y retraso mental a largo plazo y si se presenta en forma aguda fiebre, ictericia, hepatoesplenomegalia(aumento de tamaño del bazo y del Hígado) y en algunos casos miocarditis y neumonía intersticial(Infamación y daño del tejido cardiaco y pulmonar respectivamente)

¿Cuáles son los beneficios de mi niño al ser yo el sujeto de investigación?

Este formulario de consentimiento informado le suministrara toda la información que necesite acerca del estudio, puede solicitar cualquier información adicional si usted está de acuerdo en participar. A usted se le solicitara que firme el formulario de consentimiento informado, con esta decisión no afectara los cuidados médicos de su hijo.

Si usted resultara positivo para la infección de Toxoplasma gondii deberá asistir a cita entre la 2 o 4 semana posterior a su egreso con su hijo al cual se le realizara hasta ese momento prueba serología y se le dará seguimiento en la consulta externa la cual será brindada por Doctora Mélida Escala funcionaria del servicio de Neonatología. Recibirá información sobre el tema así como la condición de su bebe si el resultado fuera positivo.

Este estudio fue aprobado por el Comité de Bioética de Investigación del Hospital del Niño ya que a usted se le extraerá muestra sanguínea necesaria para el estudio, no habrá ningún gasto financiero y no se le dará compensación directa por participar en este estudio de investigación.

¿Cuáles son los propósitos de este estudio?

Conocer la frecuencia con que se presenta la Toxoplasmosis congénita ya que se puede prevenir la transmisión al niño realizando buenos controles prenatales y enviando en tiempo y forma las pruebas serológicas a la embarazada ya que las mayoría de los niños que contraen la enfermedad por transmisión materna no presentan síntomas al nacimiento si no que se manifiesta la patología cuando ya el niño presenta retraso mental, corioretinitis (inflamación de la retina) y ceguera influyendo negativamente sobre la calidad de vida de estos niños.

¿Que se requiere de usted durante el estudio?

Que nos permita extraerle 3 ml de sangre a nivel de la vena cefálica y mediana cubital del antebrazo derecho o izquierdo.

¿Se requerirá mas procedimiento después que mi niño complete el estudio?

Solo si usted resultara positiva se tomara a su bebe una muestra sanguínea 3ml (media cucharadita) de la vena cefálica y mediana cubital antebrazo derecho o izquierdo

¿Cuáles son los riesgos de participar en este estudio?

No existe ningún riesgo para su bebe, pero si usted sentirá un pinchazo o sensación de picadura en el sitio de punción al igual que su bebe si usted resultara con serología positiva para Toxoplasmosis

¿Cuales son los beneficios de participar en este estudio?

Recibirá información sobre el tema, así como condición de su niño y posterior seguimiento por consulta externa de tener un resultado positivo.

¿Se me recompensara por participar en este estudio?

El total de niños que serán seleccionados son 366 y la duración del estudio son 6 meses, su participación es voluntaria. Se guardara la confidencialidad, y el estudio fue aprobado por el Comité de Bioética de Investigación del Hospital del Niño ya que a usted se le extraerá muestra sanguínea necesaria para el estudio, no habrá ningún gasto financiero y no se le dará compensación directa por participar en este estudio de investigación.

Consentimiento informado sobre Seroprevalencia de infección de Toxoplasma gondii en mujeres embarazadas por medio de detección de anticuerpos IgG e IgM anti T. gondii y su impacto en el recién nacido, que asisten a la maternidad del Hospital Santo Tomás en el periodo de Junio – Noviembre 2014.

1. Conozco que la participación en este estudio es voluntaria
2. Doy permiso a los investigadores de este estudio que me realicen a mí y a mi hijo(a) las pruebas correspondientes así como que el momento que deseen revisar mi historial clínico y la información recolectada.

Nombre y apellido de la madre: ________________________________

Fecha: _____________________

Firma: ________________________________

Firma del que obtuvo el consentimiento_______________ Fecha________
Nombre y apellido_________________________________

Teléfono del presidente de comité de Bioética en Investigación del Hospital del Niño
Dr. Luis Coronado: 66127318

Ficha de recolección de Datos

Estudio de Investigación sobre Seroprevalencia de infección de Toxoplasma gondii en mujeres embarazadas por medio de detección de anticuerpos IgG e IgM anti T. gondii y su impacto en el recién nacido, que asisten a la Maternidad del Hospital Santo Tomás en el periodo de Junio – Noviembre 2014.

Registro_________________

Edad: 11-14 años_______15-19______ 20-25_______26-30_______31-35______36-49_______

Procedencia: Urbana_______ Rural_________

Provincia y comarca: Panamá.______ Coclé______ Veraguas______ Herrera _______

Los Santos ______ Bocas del Toro _____ Colon _____ Chiriquí ____ Darién _______

Comarca Cuna Yala________ Ngobe Bugle _________ Embera _______

Resultados Actales Titulos de anticuerpos

Toxoplasmosis: IgG Madre _______ IgM Madre______

Toxoplasmosis IgG Niño _______ IgM Niño ______

Hallazgos físicos de toxoplasmosis congénita:

Hidrocefalia: Si _____ No _____ Microcefalia: Si_____ No _____

Corioretinitis: Si _____ No _____ Calcificaciones cerebrales: Si____ No___

Hepatoesplenomegalia: Si _____ No _____ ictericia: Si _____ No _____

Convulsiones: Si ______ No ____

Hospitalización: Si _____ No_____

Tratamiento: Si_____ No_____

Fecha de entrega de Resultado ______________

Ministerio de Salud de la República de Panamá (MINSA)

with Mariángela Soberón Felín, JD

GACETA OFICIAL DIGITAL

GOBIERNO DE LA REPÚBLICA DE PANAMÁ

Año CX | Panamá, R. de Panamá miércoles 22 de octubre de 2014 | N° 27648-A

CONTENIDO

MINISTERIO DE LA PRESIDENCIA
Decreto N° 160
(De miércoles 22 de octubre de 2014)

QUE DESIGNA A LA MINISTRA Y VICEMINISTRA DE GOBIERNO, ENCARGADAS.

MINISTERIO DE LA PRESIDENCIA
Decreto N° 161
(De miércoles 22 de octubre de 20:4)

QUE DESIGNA AL MINISTRO DE COMERCIO E INDUSTRIAS, ENCARGADO.

MINISTERIO DE SALUD
Decreto Ejecutivo N° 1617
(De martes 21 de octubre de 2014)

QUE DETERMINA Y CATEGORIZA LOS EVENTOS DE SALUD PÚBLICA DE NOTIFICACIÓN E INVESTIGACIÓN OBLIGATORIA, DEFINE LOS TIPOS DE VIGILANCIA EPIDEMIOLÓGICA, LA VIGILANCIA LABORATORIAL Y SE SEÑALAN LOS PROCEDIMIENTOS PARA SU REALIZACIÓN.

MINISTERIO DE SALUD
Decreto Ejecutivo N° 1626
(De martes 21 de octubre de 2014)

QUE ADOPTA EL REGLAMENTO TÉCNICO CENTROAMERICANO RTCA 67.01.15:07 HARINAS. HARINAS DE TRIGO FORTIFICADA. ESPECIFICACIONES, ANEXO DE LA RESOLUCIÓN NO. 201 - 2007 DEL CONSEJO DE MINISTROS DE INTEGRACIÓN ECONÓMICA (COMIECO - XLV).

MINISTERIO DE ECONOMÍA Y FINANZAS
Resolución Ministerial N° 033-2014-DdCP
(De jueves 16 de octubre de 2014)

POR LA CUAL SE ESTABLECEN LAS CONDICIONES DEL DÉCIMO NOVENO TRAMO DE NOTAS DEL TESORO CON VENCIMIENTO EN FEBRERO 2021.

MINISTERIO DE TRABAJO Y DESARROLLO LABORAL
Resolución N° DM-482-14
(De miércoles 15 de octubre de 2014)

POR LA CUAL SE CONFORMA UNA COMISIÓN TÉCNICA POR PARTE DEL MINISTERIO DE TRABAJO Y DESARROLLO LABORAL, PARA ATENDER LOS TEMAS RELATIVOS A LAS NEGOCIACIONES ENTRE EL ESTADO PANAMEÑO, LA EMPRESA MIBUS Y LOS TRABAJADORES DE ESTA ÚLTIMA.

SUPERINTENDENCIA DE BANCOS
Resolución N° SBP-101-2014
(De jueves 24 de julio de 2014)

REPÚBLICA DE PANAMÁ
MINISTERIO DE SALUD

DECRETO EJECUTIVO N.° 1617
De 21 de octubre 2014

Que determina y categoriza los eventos de salud pública de notificación e investigación obligatoria, define los tipos de vigilancia epidemiológica, la vigilancia laboratorial y se señalan los procedimientos para su realización

EL PRESIDENTE DE LA REPÚBLICA
en uso de sus facultades constitucionales y legales,

CONSIDERANDO:

Que de conformidad con la Constitución Política de la República de Panamá, es función esencial del Estado velar por la salud de la población;

Que de conformidad con la Ley 66 de 10 de noviembre de 1947, que aprueba el Código Sanitario de la República de Panamá, el Órgano Ejecutivo, a proposición del Ministerio de Salud, dictará el reglamento que determinará las enfermedades de notificación obligatoria, las normas para efectuar las denuncias, los estudios epidemiológicos correspondientes, y los medios y procedimientos de control; por ende, para cumplir con esta responsabilidad se necesita realizar una efectiva vigilancia del riesgo sanitario;

Que el Decreto de Gabinete N.°1 de 15 de enero de 1969, crea al Ministerio de Salud y como ente rector del sector salud le corresponde la determinación y conducción de la política de salud del gobierno en el país;

Que para poder alcanzar los objetivos antes citados, el Ministerio de Salud necesita disponer de un sistema de vigilancia epidemiológica, que cuente con los instrumentos legales necesarios, a fin de obtener información completa, veraz y oportuna de todas las personas e instituciones públicas y privadas que, por la naturaleza de sus funciones o responsabilidad, puedan conocer la existencia de riesgos sanitarios;

Que la República de Panamá, como miembro de las Naciones Unidas, adoptó en la 58ª Asamblea Mundial de la Salud el Reglamento Sanitario Internacional de 2005, cuya finalidad es prevenir la propagación internacional de enfermedades, proteger su propagación, controlarla, y darle una respuesta de salud pública proporcionada y restringida a los riesgos para la salud pública, evitando al mismo tiempo las interferencias innecesarias con el tráfico y comercio internacional;

Que mediante la Ley 38 de 5 de abril de 2011, se adoptó el Reglamento Sanitario Internacional, conocidos por sus siglas (RSI -2005) y sus anexos,

DECRETA:

Artículo 1. Establecer la existencia de una categoría de riesgos a la salud individual y colectiva, denominados eventos de salud pública de notificación e investigación obligatoria y se definen los tipos de vigilancia epidemiológica y laboratorial.

Artículo 2. Categorizar los eventos de notificación obligatoria,para uso del sistema de vigilancia epidemiológica, según tipo de agrupamiento de la notificación como:

1. Eventos de notificación individual.
2. Eventos de notificación colectiva.

Artículo 3. Categorizar los eventos de notificación obligatoria para uso del sistema de vigilancia epidemiológica, según periodicidad de la notificación, en eventos de:

1. Notificación individual inmediata y semanal.
2. Notificación semanal.

Artículo 4. Categorizar los eventos de investigación obligatoria como:

1. Eventos de investigación inmediata o mediata.
2. Eventos de investigación ante la presencia casos o de brotes.

Artículo 5. Categorizar los diferentes tipos de vigilancia epidemiológica como:

1. Vigilancia universal.
2. Vigilancia centinela u otra modalidad.
3. Vigilancia de febriles.
4. Vigilancia laboratorial.
5. Vigilancia de factores de riesgo y de factores protectores a la salud.

Artículo 6. Vigilar universalmente todos los eventos contenidos en el artículo 19 del presente Decreto Ejecutivo, según las normas y procedimientos de vigilancia e investigación del Ministerio de Salud.

Artículo 7. El Ministerio de Salud, a través de la Dirección General de Salud Pública, definirá qué eventos de notificación obligatoria serán objeto de vigilancia centinela, según normas y procedimientos técnicos establecidos.

Artículo 8. El Ministerio de Salud, a través de la Dirección General de Salud Pública, incluirá o eliminará sistemas de vigilancia centinela, según la realidad epidemiológica nacional o internacional.

Artículo 9. El Ministerio de Salud, a través de la Dirección General de Salud Pública, autorizará qué agentes causales de enfermedad serán objetos de vigilancia laboratorial, en coordinación con los diferentes laboratorios públicos y privados, según normas y procedimientos técnicos establecidos.

Artículo 10. El Ministerio de Salud, a través de la Dirección General de Salud Pública, autorizará cuales agentes causales de enfermedad serán objetos de vigilancia laboratorial para la resistencia de los antimicrobianos o antivirales, en coordinación con los diferentes laboratorios públicos y privados, según normas y procedimientos técnicos establecidos.

Artículo 11. La vigilancia laboratorial deberá ser realizada en todos los establecimientos hospitalarios, públicos y privados, que efectúen cultivos o pruebas moleculares para microorganismos, tales como bacterias, virus, hongos u otros, por sus propios medios o con el apoyo del Laboratorio Central de Referencia del Instituto Conmemorativo Gorgas de Estudios de la Salud, de acuerdo a como lo dispone la norma y procedimiento técnico correspondiente.

Artículo 12. Los laboratorios de los hospitales deberán remitir al Laboratorio Central de Referencia del Instituto Conmemorativo Gorgas de Estudios de la Salud, la información de los resultados de la vigilancia laboratorial; a su vez, dichos laboratorios, informarán los resultados de esta vigilancia al Ministerio de Salud, de acuerdo a como lo dispone la norma y procedimiento técnico correspondiente.

Artículo 13. Los laboratorios clínicos, veterinarios, laboratorios de agua para consumo humano y los bancos de sangre, públicos y privados, en que se identifiquen los agentes causales de las enfermedades mencionados en el artículo 19, estarán obligados a notificarlos semanalmente al Instituto Conmemorativo Gorgas de Estudios de la Salud, y éste a su vez, al Departamento Nacional de Epidemiología, mediante formularios provistos para este fin, en los que se deben registrar los siguientes antecedentes:

1. Identificación del paciente.

PRESIDENCIA DE LA REPÚBLICA DE PANAMÁ
MINISTERIO DE LA PRESIDENCIA
REGISTRADO

2. Diagnóstico.
3. Naturaleza de la(s) muestra(s); tipo de muestra, tales como orina, sangre y otros.
4. Institución solicitante.

Artículo 14. Los establecimientos mencionados en el artículo 12, deberán enviar las muestras o cepas correspondientes, al Laboratorio Central de Referencia del Instituto Conmemorativo Gorgas de Estudios de la Salud, el cual realizará el estudio del agente y notificará los resultados al Departamento Nacional de Epidemiología del Ministerio de Salud.

Artículo 15. Se considerarán eventos de salud pública, sujetos a vigilancia epidemiológica, los factores de riesgo y factores protectores asociados a las enfermedades más prevalentes del país, a través de formas o establecimientos definidos por la Dirección General de Salud Pública, según las normas y procedimientos de vigilancia e investigación del Ministerio de Salud.

Artículo 16. La vigilancia universal y la centinela incluyen a los laboratorios y otras instalaciones de atención designadas, según las normas y procedimientos de vigilancia e investigación del Ministerio de Salud.

Artículo 17. Para los efectos del presente Decreto Ejecutivo, se tendrán las siguientes definiciones:

1. **Autoridad Competente en eventos radiológicos:** Autoridad nombrada o reconocida de otra forma por el Gobierno, con fines de reglamentación en materia de protección y seguridad radiológica.
2. **Brote:** Es la aparición de dos o más casos o eventos de salud pública nuevos asociados en tiempo, lugar y persona; cambio de la frecuencia de hospitalizaciones o muertes, o cambio en la afectación de los grupos de poblaciones humanas que supera los valores esperados. En caso de enfermedades nuevas o eliminadas, un caso es un brote.
3. **Caso sospechoso:** Es toda persona viva o fallecida que por los antecedentes, la evidencia clínica o ambos, se puede presumir la existencia de un evento de salud de notificación obligatoria.
4. **Caso confirmado:** Es todo caso sospechoso con evidencia laboratorial, imagenológica u otra evidencia definitiva de un evento de salud de notificación obligatoria.
5. **Caso confirmado por nexo epidemiológico:** Es todo caso sospechoso y con vínculo a un caso confirmado.
6. **Evento imprevisto:** Evento causado por una enfermedad o un agente ya eliminado o erradicado del Estado parte o no notificado anteriormente.
7. **Evento inusitado:** Es la presencia de un evento causado por un agente desconocido, o bien la fuente, el vehículo o la vía de transmisión son inusitados o desconocidos, o la evolución de los casos es más grave de lo previsto o presenta síntomas no habituales o la manifestación del evento resulta inusual para la zona, la estación o la población.
8. **Emergencia de salud pública de importancia internacional (ESPII):** Es un evento extraordinario que, de conformidad con el Reglamento Sanitario Internacional de 2005 se ha determinado que constituye un riesgo para la salud pública de otros Estados, a causa de la propagación internacional de una enfermedad y podría exigir una respuesta internacional coordinada.
9. **Enfermedad emergente:** Son aquellas enfermedades infecciosas nuevas o infecciones multiresistentes, cuya incidencia se ha incrementado en los últimos años.
10. **Enfermedad reemergente:** Es una enfermedad transmisible, previamente conocida, que reaparece como problema de salud pública, tras una etapa de significativo descenso de su incidencia y aparente control.
11. **Eventos de notificación obligatoria (ENO):** Son los rumores, sospechas, casos o defunciones por enfermedades, riesgos u otros eventos de salud, que por su transmisibilidad, peligrosidad o potencialidad de daño, sean definidos como tales, por el presente Decreto Ejecutivo.
12. **Eventos de salud de notificación obligatoria inmediata:** Son los rumores, sospechas, casos o defunciones de aquellas enfermedades, riesgos o eventos de salud, que por su transmisibilidad, peligrosidad o potencialidad de daño a la salud, deben ser

comunicados de forma inmediata, definidos como tales por el presente Decreto Ejecutivo.

13. **Eventos de salud de notificación obligatoria semanal**: Son los rumores, sospechas, casos o defunciones por aquellas enfermedades, riesgos o eventos de salud, que por su transmisibilidad, peligrosidad o potencialidad de daño a la salud, deben ser comunicados de forma semanal, definidos como tales por el presente Decreto Ejecutivo.
14. **Evento de salud pública de importancia internacional:** Es un evento de salud extraordinario que, de conformidad con el Reglamento Sanitario Internacional, Anexo II, constituye un riesgo para la salud pública de otros Estados y podría exigir una respuesta internacional coordinada y que debe ser notificada por el Estado parte.
15. **Evento de Salud Pública de Importancia Nacional (ESPIN):** Son los eventos de salud que ocurren en el territorio nacional y, a juicio del Ministerio de Salud son o pueden convertirse en un riesgo para la salud pública nacional.
16. **Emergencias Radiológicas:**Todo suceso involuntario, incluido un error de operación, fallo de equipo u otro contratiempo, cuyas consecuencias reales o potenciales no puedan desconocerse desde el punto de vista de la protección o seguridad, y que pudiera conducir a una exposición potencial o a condiciones de exposición anormales.
17. **Investigación inmediata:** Es la búsqueda de información necesaria y complementaria de todo rumor, sospecha, caso o defunción, de un evento de salud y de sus contactos, en menos de cuarenta y ocho (48) horas, contados a partir del momento en que se tuvo conocimiento de su existencia, de acuerdo a lo estipulado en las normas y procedimientos de vigilancia epidemiológica y otras leyes especiales que regulan el tema tales como bioterrorismo, violencia intrafamiliar y otras.
18. **Investigación mediata:** Es la búsqueda de información necesaria y complementaria, en menos de quince (15) días calendario, contados a partir del momento en que se tuvo conocimiento de su existencia, de todo rumor, sospecha, caso o defunción, de un problema o evento de salud, de acuerdo a lo estipulado en las normas de vigilancia epidemiológica y otras leyes especiales que regulan el tema.
19. **Investigación de brotes:** Es la búsqueda de información necesaria y complementaria en menos de cuarenta y ocho (48) horas, según las normas y procedimientos de vigilancia epidemiológica y otras leyes especiales que regulan el tema.
20. **Notificación obligatoria:** Es la comunicación de los eventos de salud de notificación obligatoria a las autoridades sanitarias establecidas en el presente decreto, de acuerdo a las normas y procedimientos de vigilancia epidemiológica.
21. **Notificación inmediata:** Es la comunicación por vía telefónica, fax, por escrito o en medio electrónico de todo evento de salud, al coordinador local, regional o central de epidemiología del Ministerio de Salud y, en su ausencia, al encargado de la entidad de salud pública más cercana, en menos de dos (2) horas, contadas a partir del momento en que se tuvo conocimiento de su existencia, y de acuerdo a lo señalado en las normas y procedimientos de vigilancia epidemiológica.
22. **Notificación semanal:** Es la comunicación por escrito, vía fax o medio electrónico de todo evento de salud al coordinador local, regional o central de epidemiología del Ministerio de Salud en la semana epidemiológica siguiente, de acuerdo a lo señalado en las normas y procedimientos de vigilancia epidemiológica. Este reporte debe ser realizado a más tardar a la 1:00 p.m. del segundo día laboral de la semana epidemiológica siguiente al nivel central de epidemiología.
23. **Riesgo para la salud pública:** Significa la probabilidad de que se produzca un evento que puede afectar adversamente a la salud de las poblaciones humanas, considerando en particular la probabilidad de que se propague internacionalmente o pueda suponer un peligro grave y directo.
24. **Rumor:** Son opiniones espontáneas y no confirmadas, originadas en la comunidad o en medios de comunicación de masas, asociadas a la presencia o incremento de problemas o eventos de salud pública, de notificación obligatoria o que implican algún riesgo a la salud individual o colectiva.
25. **Semana epidemiológica:** Es la unidad básica temporal para la notificación de casos y su agregación y análisis en los niveles locales, regionales y nacionales. Se cuenta a partir del domingo de cada semana y finaliza a las veinticuatro (24) horas del sábado

PRESIDENCIA DE LA REPÚBLICA DE PANAMÁ
MINISTERIO DE LA PRESIDENCIA
REGISTRADO

siguiente. El Ministerio de Salud oficializará, cada año, el calendario con las semanas epidemiológicas a utilizar, por el sistema de vigilancia epidemiológico.

26. **Zoonosis:** Es una infección o enfermedad infecciosa transmisible, que en condiciones naturales, ocurre entre los animales vertebrados y el hombre.

Artículo 18. Para los efectos del presente Decreto Ejecutivo, se utilizarán además las definiciones de los eventos, los que se encuentren en la correspondiente norma técnica de cada uno de estos eventos a vigilar.

Artículo 19. Se declaran de notificación obligatoria en la República de Panamá, los rumores, sospechas, casos confirmados o defunciones al Departamento de Epidemiología de la Dirección General de Salud Pública, de los eventos de salud contenidos en el siguiente listado:

1. Amebiasis intestinal;
2. Botulismo;
3. Bronconeumonía;
4. Bronquiolitis;
5. Brucelosis;
6. Carbunco;
7. Chancro blando;
8. Cólera;
9. Dengue sin signos de alarma (DSSA), Dengue con signos de alarma (DCSA) y Dengue grave (DG);
10. Diarreas por rotavirus;
11. Difteria;
12. Efecto tóxico del contacto con escorpión;
13. Efecto tóxico del contacto con serpiente;
14. Efectos tóxicos agudos por plaguicidas;
15. Efectos tóxicos por metales;
16. Encefalitis virales: Equina del Este, San Luis, Equina del Oeste, Equina Venezolana y otras sin especificar;
17. Enfermedad de Creutzfeld-Jakob y su variante, Encefalopatía espongiforme;
18. Enfermedad de Chagas: Tripanosomiasis americana;
19. Enfermedad diarreica y gastroenteritis de origen infeccioso;
20. Enfermedad meningocóccica;
21. Hantavirus;
22. Eventos supúestamente atribuidos a la vacunación o inmunización (ESAVI);
23. Eventos de bioterrorismo;
24. Eventos de emergencia química o ambiental;
25. Eventos de emergencia radiológica o radio nucleares;
26. Fiebre amarilla;
27. Fiebre del Valle del Rift;
28. Fiebre equina venezolana;
29. Fiebre hemorrágica virales: Ébola, Lassa, Marburgo, Chicungunya y otras;
30. Fiebre del Nilo Occidental;
31. Fiebre recurrente por piojos;
32. Fiebre tifoidea y paratifoidea;
33. Filariasis;
34. Geohelmintiasis;
35. Granuloma inguinal;
36. Hepatitis: A, B, C, D, E, y otras no especificadas;
37. Herpes genital;
38. Infección gonocócica;
39. Infección por VIH/Sida;
40. Infección por virus papiloma humano;
41. Infecciones asociadas a la atención de salud (IAAS);
42. Infección por virus linfotrópico humano HTLVI-II;
43. Influenza o gripe;
44. Influenza por un nuevo subtipo de virus;

45. Intoxicación alimentaria;
46. Invaginación o intususcepción intestinal en niños menores de un(1) año de edad;
47. Leishmaniasis;
48. Lepra;
49. Leptospirosis;
50. Linfogranuloma venéreo;
51. Meningitis bacterianas: Meningitis por Haemophilus influenzae, Meningitis por Streptococus pneumoniaey sin especificar;
52. Meningitis virales;
53. Mordedura de murciélago;
54. Mordedura de perro;
55. Muertes maternas, perinatal e infantil;
56. Neumonia;
57. Oncocercosis;
58. Otras afecciones inflamatorias de la vagina y de la vulva;
59. Paludismo;
60. Parálisis flácida aguda en menores de quince (15) años;
61. Parotiditis;
62. Peste;
63. Poliomielitis;
64. Rabia en humanos;
65. Rickettsiosis;
66. Rubéola;
67. Salmonelosis;
68. Sarampión;
69. Shiguelosis;
70. Sífilis y otras no especificadas;
71. Sífilis congénita;
72. Sífilis en embarazadas;
73. Sífilis primaria;
74. Sindrome Coqueluchoide;
75. Síndrome febril agudo hemorrágico;
76. Síndrome febril agudo ictérico;
77. Síndrome febril agudo neurológico;
78. Síndrome febril con distress respiratorio;
79. Síndrome respiratorio agudo severo (SARS);
80. Síndrome rubéola congénita;
81. Tétanos y tétanos neonatal;
82. Tifus exantemático por piojos;
83. Tos ferina;
84. Toxoplasmosis congénita;
85. Tuberculosis;
86. Ulceración e inflamación vulvovaginal en enfermedades clasificadas en otra parte;
87. Uretritis no especificadas;
88. Varicela;
89. Violencia intrafamiliar;
90. Viruela.
91. Todo agente nuevo o resistencia a las terapias utilizadas, identificado a través de laboratorios públicos y privados;
92. Todo brote de enfermedad o evento nuevo, de importancia para la salud pública nacional o internacional, incluso los de origen desconocido, no incluido en este listado;
93. Todos los hallazgos de los sistemas de vigilancia epidemiológicas especiales, los hallazgos positivos de los sistemas de vigilancia clínica laboratorial, los hallazgos de investigaciones en humanos a animales y de otros sistemas de monitoreos incluidos los de alimentos y vigilancia del agua para consumo humano.

Artículo 20. Clasificar los eventos de salud de notificación obligatoria, según tipo y periodicidad de la notificación, y tipo de investigación epidemiológica y periodicidad, de acuerdo al siguiente cuadro:

	Eventos de Salud Pública de Notificación Obligatoria	Tipo de notificación	Periodicidad de la notificación	Tipo de investigación epidemiológica y periodicidad	
		Individual Colectiva	Semanal – 1 Inmediata y semanal =2	Caso	Brotes
1	Amebiasis intestinal	Colectiva	1		Inmediata
2	Botulismo	Individual	2	Inmediata	Inmediata
3	Bronconeumonía	Colectiva	1		Inmediata
4	Bronquiolitis	Colectiva	1		Inmediata
5	Brucelosis	Individual	1	Mediata	Inmediata
6	Carbunco	Individual	2	Inmediata	Inmediata
7	Chancro blando	Colectiva	1		Inmediata
8	Cólera	Individual	2	Inmediata	Inmediata
9	Dengue sin signos de alarma (DSSA), Dengue con signos de alarma (DCSA) y Dengue grave (DG)	Individual	2	Inmediata	Inmediata
10	Diarreas por rotavirus	Colectiva	1		Inmediata
11	Difteria	Individual	2	Inmediata	Inmediata
12	Efecto tóxico del contacto con escorpión	Colectiva	1		Inmediata
13	Efecto tóxico del contacto con serpiente	Colectiva	1		Inmediata
14	Efectos tóxicos agudos por plaguicidas	Individual	1	Mediata	Inmediata
15	Efectos tóxicos por metales	Individual	1	Mediata	Mediata
16	Encefalitis virales: Equina del Este, San Luis, Equina del Oeste, Equina Venezolana y otras sin especificar	Individual	2	Inmediata	Inmediata
17	Enfermedad de Creutzfeld-JaKob y su variante, Encefalopatía espongiforme	Individual	1	Mediata	Mediata
18	Enfermedad de Chagas: Tripanosomiasis americana	Individual	1	Mediata	Inmediata
19	Enfermedad diarreica y gastroenteritis de origen infeccioso	Colectiva	1		Inmediata
20	Enfermedad meningocóccica	Individual	2	Inmediata	Inmediata
21	Hantavirus	Individual	2	Inmediata	Inmediata
22	Eventos supuestamente atribuidos a la vacunación o inmunización (ESAVI)	Individual	2	Inmediata	Inmediata
23	Eventos de bioterrorismo	Individual	2	Inmediata	Inmediata
24	Eventos de emergencia química o ambiental	Individual	2	Inmediata	Inmediata
25	Eventos de emergencia radiológica o radionucleares	Individual	2	Inmediata	Inmediata
26	Fiebre amarilla	Individual	2	Inmediata	Inmediata
27	Fiebre del Valle del Rift	Individual	2	Inmediata	Inmediata
28	Fiebre equina venezolana	Individual	2	Inmediata	Inmediata
29	Fiebre hemorrágica virales: Ébola, Lassa, Marburgo, Chicungunya y otros	Individual	2	Inmediata	Inmediata
30	Fiebre del Nilo Occidental	Individual	2	Inmediata	Inmediata
31	Fiebre recurrente por piojos	Individual	2	Inmediata	Inmediata
32	Fiebre tifoidea y paratifoidea	Individual	2	Inmediata	Inmediata
33	Filariasis	Individual	1	Mediata	Mediata
34	Geohelmintiasis	Colectiva	1		
35	Granuloma inguinal	Colectiva	1		[illegible]

PRESIDENCIA DE LA REPÚBLICA DE PANAMÁ
MINISTERIO DE LA PRESIDENCIA
REGISTRADO

36	Hepatitis: A, B, C, D, E, y otras no especificadas	Individual	1	Mediata	Mediata
37	Herpes genital	Colectiva	1		
38	Infección gonocócica	Colectiva	1		Mediata
39	Infección por VIH/Sida	Individual	1	Mediata	Inmediata
40	Infección por virus papiloma humano	Colectiva	1		
41	Infecciones asociadas a la atención de la salud (IAAS)	Colectiva	1		Inmediata
42	Infección por virus linfotrópico humano HTLVI-II	Individual	1	Mediata	
43	Influenza o gripe	Colectiva	1		Inmediata
44	Influenza por un nuevo subtipo de virus	Individual	2	Inmediata	Inmediata
45	Intoxicación alimentaria	Individual	1		Inmediata
46	Invaginación o intususcepción intestinal en niños menores de un(1) año de edad	Individual	1	Mediata	
47	Leishmaniasis	Individual	1		Mediata
48	Lepra	Individual	1	Mediata	Mediata
49	Leptospirosis	Individual	2	Inmediata	Inmediata
50	Linfogranuloma venéreo	Colectiva	1		
51	Meningitis bacterianas: Meningitis por Haemophilus influenzae, Meningitis por Streptococus pneumoniae y sin especificar	Individual	2	Inmediata	Inmediata
52	Meningitis virales	Individual	2	Inmediata	Inmediata
53	Mordedura de murciélago	Individual	2	Inmediata	Inmediata
54	Mordedura de perro	Colectiva	1		
55	Muertes maternas, perinatal e infantil	Individual	1	Mediata	
56	Neumonia	Colectiva	1		Inmediata
57	Oncocercosis	Individual	1	Mediata	Mediata
58	Otras afecciones inflamatorias de la vagina y de la vulva	Colectiva	1		
59	Paludismo	Individual	2	Inmediata	Inmediata
60	Parálisis flácida aguda en menores de quince (15) años	Individual	2	Inmediata	Inmediata
61	Parotiditis	Individual	1	Mediata	Inmediata
62	Peste	Individual	2	Inmediata	Inmediata
63	Poliomielitis	Individual	2	Inmediata	Inmediata
64	Rabia en humanos	Individual	2	Inmediata	Inmediata
65	Rickettsiosis	Individual	2	Inmediata	Inmediata
66	Rubéola	Individual	2	Inmediata	Inmediata
67	Salmonelosis	Individual	1	Mediata	Inmediata
68	Sarampión	Individual	2	Inmediata	Inmediata
69	Shiguelosis	Individual	1	Mediata	Inmediata
70	Sífilis y otras no especificadas	Individual	1	Mediata	
71	Sífilis congénita	Individual	1	Mediata	
72	Sífilis en embarazadas	Individual	1	Mediata	
73	Sífilis primaria	Individual	1	Mediata	
74	Síndrome Coqueluchoide	Individual	2	Inmediata	Inmediata
75	Síndrome febril agudo hemorrágico	Individual	2	Inmediata	Inmediata
76	Síndrome febril agudo ictérico	Individual	2	Inmediata	Inmediata
77	Síndrome febril agudo neurológico	Individual	2	Inmediata	Inmediata
78	Síndrome febril con distress respiratorio	Individual	2	Inmediata	Inmedi[illegible]

PRESIDENCIA DE LA REPÚBLICA DE PANAMÁ
MINISTERIO DE LA PRESIDENCIA
REGISTRADO

79	Síndrome respiratorio agudo severo (SARS)	Individual	2	Inmediata	Inmediata
80	Síndrome rubéola congénita	Individual	2	Inmediata	
81	Tétanos y tétanos neonatal	Individual	1	Mediata	
82	Tifus exantemático por piojos	Individual	2	Inmediata	Inmediata
83	Tos ferina	Individual	2	Inmediata	Inmediata
84	Toxoplasmosis congénita	Individual	1	Mediata	
85	Tuberculosis	Individual	1	Mediata	Inmediata
86	Ulceración e inflamación vulvovaginal en enfermedades clasificadas en otra parte	Colectiva	1		
87	Uretritis no especificadas	Colectiva	1		
88	Varicela	Colectiva	1		
89	Violencia intrafamiliar	Individual	1		
90	Viruela	Individual	2	Inmediata	Inmediata
91	Todo agente nuevo o resistente a las terapias utilizadas, identificado a través de laboratorios público o privados	Individual	2	Inmediata	Inmediata
92	Todo evento nuevo, de importancia para la salud pública nacional o internacional, incluso los de origen desconocido, no incluido en este listado	Individual	2	Inmediata	Inmediata
93	Todos los hallazgos de los sistemas de vigilancia epidemiológicas especiales como centinelas y otros, y de los sistemas de vigilancia clínica laboratorial que identifiquen resultados positivos	Individual	1	Inmediata	Inmediata

Artículo 21. La Dirección General de Salud Pública, vigilará que se elaboren y actualicen periódicamente las normas y procedimientos de vigilancia e investigación epidemiológica.

Artículo 22. El Ministerio de Salud pondrá a disposición un sistema de información que permita al sector público, privado, patronatos, organizaciones no gubernamentales y otras instituciones que presten servicios de salud, el reporte oportuno de los eventos de salud de notificación e investigación obligatoria.

Artículo 23. Para facilitar la notificación de los eventos, contemplados en el presente Decreto Ejecutivo, todas las instalaciones en donde existan responsables de registros médicos o personal encargado y de epidemiología en el Ministerio de Salud, patronatos y el personal de la Caja de Seguro Social, deberán de contar con equipamiento informático y acceso a internet en un plazo no mayor de dos (2) años, a partir de la promulgación del presente Decreto Ejecutivo. Solo se eximirá del acceso a internet, a las áreas donde no se pueda captar la señal.

Artículo 24. La autoridad sanitaria, en los diferentes niveles de la organización de salud, podrá designar comisiones para investigar eventos de salud o brotes contemplados en el presente Decreto Ejecutivo y los directores de instituciones de salud pública o privadas u otras, deberán de inmediato dar toda la cooperación para el cumplimiento de este mandato.

Artículo 25. Para la realización de las investigaciones epidemiológicas, todas las regiones e instalaciones de salud deberán de tener disponibles y debidamente entrenados, al menos a un (1) equipo de respuesta rápida (ERR), para realizar las investigaciones correspondientes, según lo define el artículo 20 del presente Decreto Ejecutivo.

PRESIDENCIA DE LA REPÚBLICA DE PANAMÁ
MINISTERIO DE LA PRESIDENCIA
REGISTRADO

Artículo 26. Los directores regionales, directores de hospitales y administradores, son responsables de brindar todo el apoyo logístico a los equipos de respuesta rápida (ERR), para que estos puedan realizar las investigaciones epidemiológicas contempladas en el presente Decreto Ejecutivo, aún en horarios no regulares.

Artículo 27. Los hospitales públicos y privados, deberán de tener constituido formalmente unidades de epidemiologia y equipo de respuesta rápida, que podrán ampliar o modificar, de acuerdo al tipo de evento que se investiga, los cuales están obligados a cumplir las normas de vigilancia epidemiológica.

Artículo 28. Los hospitales públicos y privados o cualquier otra instalación, están obligados en forma expedita permitir al Ministerio de Salud realizar la investigación, monitoreo y evaluación de los eventos contemplados en este Decreto Ejecutivo dentro de sus instalaciones y proporcionar información inmediata o según lo requiera el (la) Director (a) General de Salud Pública o sus designados.

Artículo 29. Los responsables de las instituciones públicas, privadas, organizaciones y otras, que realicen investigaciones o que brinden servicios de salud humana, salud animal o realicen monitoreo ambiental, y detecten fauna o flora nociva o cualquier riesgo a la salud humana, incluyendo los eventos de salud, contemplados en el presente Decreto Ejecutivo, están obligados a notificar de forma oportuna y completa, a epidemiología del Ministerio de Salud del nivel correspondiente, por la vía más rápida disponible.

Artículo 30. Todas las empresas de servicios aeroportuarios, puertos, puestos fronterizos, aerolíneas, embarcaciones, aeronaves, u otro transporte público o privado, organizaciones no gubernamentales (ONG), los servicios públicos, tales como aduana, migración, seguridad y otros, están obligados para efectos de investigación epidemiológica, a proporcionar de inmediato, el nombre de los enfermos, expuestos y contactos de cualquier evento de salud de notificación obligatoria incluida en el presente Decreto Ejecutivo y la siguiente información adicional: número de cédula o pasaporte, nombre y teléfono de algún familiar o contacto.

Artículo 31. Los médicos, odontólogos, enfermeras, veterinarios, laboratoristas, ayudantes de salud y otros que identifiquen los eventos de salud, contenidos en el presente Decreto Ejecutivo, de las instalaciones del Ministerio de Salud, unidades ejecutoras de la Caja del Seguro Social (CSS), sector privado y otras instituciones, lo comunicarán al responsable de epidemiologia de su institución y, en su ausencia, al director de la instalación de salud y estos al nivel regional de epidemiología, quiénes lo notificarán a nivel central de epidemiología del Ministerio de Salud, de acuerdo a las normas y procedimientos de vigilancia epidemiológica.

Artículo 32. Los eventos de salud señalados en el artículo 19 del presente Decreto Ejecutivo, como de notificación obligatoria, en las instituciones del sistema público o privado de salud, deben ser notificados en forma electrónica o escrita; en caso de no disponer de sistema vía web, con la periodicidad establecida de acuerdo a las normas y procedimientos para la vigilancia e investigación epidemiológica elaborado por el Ministerio de Salud. Esto no invalida la notificación inmediata por el medio accesible en los casos que se indica.

Artículo 33. Todas las instalaciones de salud deberán clasificar los casos confirmados de eventos, según la Clasificación Estadística Internacional de Enfermedades y Problemas relacionados con la salud, cuando así aplique.

Artículo 34. Todas las instalaciones públicas y privadas que brinden servicios de salud o las que realicen monitoreo ambiental, están obligadas a comunicar los problemas o eventos señalados en el artículo 19 del presente Decreto Ejecutivo, según las normas y procedimientos para la vigilancia e investigación epidemiológica, elaborado por el Ministerio de Salud, por la vía más expedita con que se cuente, ya sea teléfono, fax, correo electrónico, por el sistema electrónico vigente o personalmente y escrito, al encargado de la entidad de salud.

Artículo 35. Cuando la notificación realizada fuese telefónica, el informe escrito debe [illegible] enviado en un término no mayor de cuarenta y ocho (48) horas, de acuerdo a las [illegible]

PRESIDENCIA DE LA REPÚBLICA DE PANAMÁ
MINISTERIO DE LA PRESIDENCIA
REGISTRADO

procedimientos para la vigilancia e investigación epidemiológica, elaborado por el Ministerio de Salud.

Artículo 36. La notificación individual, colectiva y la investigación de casos y brotes de los eventos de salud, contenidos en los artículos 19 y 20 del presente Decreto Ejecutivo, deben realizarse en los formularios establecidos, de acuerdo a las normas y procedimientos para la vigilancia e investigación epidemiológica, elaborados por el Ministerio de Salud, considerándose imprescindible para los eventos de notificación individual, los siguientes datos:

1. Nombres y apellidos de la persona sospechosa o enferma. Si es menor de edad o discapacitado, además se debe notificar el nombre de la persona responsable.
2. Número de la cédula de identidad personal, en su ausencia, número de expediente clínico, seguro social o pasaporte.
3. Edad.
4. Sexo.
5. Lugar de residencia: provincia, región, distrito, corregimiento, localidad, sector, zona o barriada, lugares de referencia de ser necesario y teléfono, si tiene.
6. Diagnóstico probable o sospechoso o confirmado por clínica, laboratorio o nexo epidemiológico.
7. Fechas de inicio de síntomas y hospitalización, condición del paciente y fecha defunción, si fuere el caso.
8. Lugar donde se presume que se produjo el contagio o la exposición, si se conociera;
9. Fecha de toma de muestra para estudio de laboratorio si fuere el caso y resultados de estar disponibles.
10. Identificar la región de salud e instalación donde se detectó el caso, incluyendo el número de teléfono, si lo hubiere.
11. Observaciones de ser necesario.
12. Fecha de notificación.
13. Nombre, apellido y cargo de la persona que notifica el caso.

En caso de que por la naturaleza del problema se requieran datos complementarios a los señalados, la autoridad sanitaria podrá solicitarlos y deberán ser proporcionados.

Artículo 37. Todas las instalaciones públicas, privadas y otras que detecten riesgos a la salud individual y colectiva, ya sean productos de sistemas de vigilancia, investigaciones, monitoreo o análisis de productos de consumo u otro uso humano, deberán de notificarlo inmediatamente al Departamento de Epidemiología del Ministerio de Salud, según las normas y procedimientos de vigilancia e investigación epidemiológica del Ministerio de Salud

Artículo 38. Las investigaciones epidemiológicas de los eventos de salud, contenidos en el artículo 20 del presente Decreto Ejecutivo, se realizará en el tiempo y términos contenidos en las normas y procedimientos de vigilancia e investigación epidemiológica del Ministerio de Salud, coordinadas por el responsable de epidemiología o por el director de la instalación de salud, o por quien ellos designen a excepción de aquellas investigaciones de eventos de notificación obligatoria que estén regulados por otras normas o procedimientos o legislaciones: violencia intrafamiliar, muertes maternas, muertes perinatales, muertes infantiles y muertes de menores de cinco (5) años, eventos químicos, eventos radiológicos, bioterrorismo, reacciones adversas a medicamentos, farmacovigilancia y otras, en donde epidemiología apoyará las acciones de investigación y seguimiento.

Artículo 39. Quedan obligados a notificar los eventos de salud, según las normas y procedimientos de vigilancia e investigación del Ministerio de Salud, las siguientes personas:

1. El médico, el odontólogo y la enfermera que asistan pacientes con enfermedad, riesgo o evento de salud de notificación obligatoria.
2. El médico que asista o realice una autopsia/necropsia, cuya causa se presuma constituya una enfermedad, riesgo o evento de salud que se encuentre entre las de notificación obligatoria.
3. Los médicos que laboran en el Instituto de Medicina Legal y Ciencias Forenses.

PRESIDENCIA DE LA REPÚBLICA DE PANAMÁ
MINISTERIO DE LA PRESIDENCIA
REGISTRADO

4. El responsable de la instalación pública o privada donde se presente un caso o sospecha, incluyendo los puntos de entrada designados independiente de su título profesional.
5. El dueño o encargado del laboratorio o banco de sangre, institutos de investigaciones y laboratorista que identifiquen resultados positivos.
6. Los veterinarios, en caso de zoonosis de importancia en salud pública.
7. La persona responsable del monitoreo ambiental que detecte una situación de riesgo o problema sanitario.
8. Los registradores auxiliares del Tribunal Electoral.
9. Los funcionarios del Instituto Nacional de Estadística y Censo de la Contraloría General de la República.
10. La persona responsable del enfermo o de la situación de riesgo o problema sanitario.
11. Cualquier persona que tuviera conocimiento o sospecha de la existencia de uno de estos casos.

Artículo 40. Cuando la denuncia sea hecha ante otra autoridad administrativa, como en caso de corregidores, otras autoridades civiles y policías entre otros, ésta la transmitirá inmediatamente a la autoridad de salud correspondiente.

Artículo 41. El Ministerio de Salud estará obligado a incluir en sus normas y procedimientos de vigilancia e investigación, los mecanismos para preservar la confidencialidad de las personas que han padecido los eventos de salud incluidos en este Decreto Ejecutivo.

Artículo 42. Es responsabilidad de los Departamentos de Registros Médicos y Estadísticas consolidar y enviar, los informes de los eventos de salud contenidos en el presente Decreto Ejecutivo, que se capten en la red de servicios de salud, según lo establece las normas y procedimientos de vigilancia e investigación del Ministerio de Salud y de las bases de datos de los sistemas de vigilancia epidemiológicas especiales.

Artículo 43. Los responsables de laboratorios y bancos de sangre notificarán los resultados de las muestras humanas, animales, ambientales, agua y alimentos, al responsable de epidemiología del nivel correspondiente y a los responsables de la toma y envío de muestras, según las normas y procedimientos de vigilancia e investigación del Ministerio de Salud.

Artículo 44. Los laboratorios clínicos públicos y privados, en que se efectúen exámenes que confirmen algunas de las enfermedades establecidas en el artículo 19 del presente Decreto Ejecutivo, deberán notificarlas a la autoridad sanitaria correspondiente, con los siguientes datos: nombre, apellidos, edad, sexo y domicilio de la persona a quién se le practicó el examen; tipo de examen, sin perjuicio de que su resultado sea enviado al profesional o institución que lo solicitó.

Artículo 45. La Dirección General de Salud Pública podrá agregar o eliminar eventos de salud pública, sujetos a vigilancia epidemiológica o laboratorial y modificar el tipo, periodicidad de la notificación y las formas de investigación de los casos, según necesidades, así como establecer otras estrategias de vigilancia que complementen las de notificación obligatoria.

Artículo 46. Los responsables de epidemiología de los hospitales o quienes la administración deleguen, están obligados a notificar todas las defunciones debidas a las enfermedades de notificación obligatorias (ENO), según las normas y procedimientos de vigilancia e investigación del Ministerio de Salud.

Artículo 47. Cada instalación de salud, tanto del sector público como privado, deberá asignar un responsable para la vigilancia epidemiológica de estos eventos y designarle el tiempo y apoyo logístico para que realice sus funciones.

Artículo 48. Ninguna entidad pública ni privada podrá publicar o hacer uso de la información emanada de la vigilancia de los eventos incluidos en el presente Decreto Ejecutivo, sin hacer referencia a su origen.

Artículo 49. La información resultante de la vigilancia de los eventos de salud de notificación obligatoria podrá ser compartida en los diferentes niveles según las normas y procedimientos de vigilancia e investigación del Ministerio de Salud.

Artículo 50. El Ministerio de Salud, la Caja de Seguro Social y demás instituciones obligadas a notificar los eventos de salud incluidos en el presente Decreto Ejecutivo, tomarán las previsiones presupuestarias, a fin de que en el nivel respectivo, se cuente con las facilidades logísticas y los fondos suficientes que permitan asegurar la vigilancia, investigación y confirmación laboratorial, imagenológica y las acciones de prevención y control permanente.

Artículo 51. El tratamiento de los datos obtenidos como resultado de las notificaciones y comunicaciones a que alude el presente Decreto Ejecutivo, se regirán por lo establecido en la Ley 68 del 20 de noviembre de 2003, que regula los derechos y obligaciones de los pacientes, en materia de información y de decisión libre e informada en su capítulo III, Derecho a la Intimidad.

Artículo 52. El Ministerio de Salud, a través de la Dirección General de Salud Pública, deberá de publicar los datos obtenidos de la vigilancia epidemiológica de los eventos de notificación obligatoria en su página web y publicar boletines epidemiológicos en forma periódica.

Artículo 53. Se autoriza a los médicos tratantes, directores de hospitales, responsables de epidemiologia o al (la) Director (a) General de Salud Pública a solicitar, con carácter de obligatoriedad, y en forma oportuna, es decir, en menos de cuarenta y ocho (48) horas, la autopsia en los casos de que no se haya dilucidado el diagnóstico final de cualquier evento de salud contemplados en el presente Decreto Ejecutivo.

Artículo 54. El responsable de la entidad, pública o privada, los funcionarios o profesionales implicados o la persona natural, que infrinjan el presente Decreto Ejecutivo, serán sancionados de conformidad con el procedimiento y las sanciones contempladas en la Ley 66 de 1947, modificada por la Ley 40 de 2006.

Artículo 55. El presente Decreto Ejecutivo deroga el Decreto Ejecutivo N.°268 de 17 de agosto de 2001, así como cualquier disposición que le sea contraria.

Artículo 56. El presente Decreto Ejecutivo empezará a regir a partir de su promulgación.

FUNDAMENTO DE DERECHO: Ley 66 de 10 de noviembre de 1947, Decreto de Gabinete N.°1 de 15 de enero de 1969.

COMUNÍQUESE Y CÚMPLASE.

Dado en la ciudad de Panamá a los 21 ( ) días del mes de oct. del año dos mil catorce (2014).

JUAN CARLOS VARELA RODRÍGUEZ.
Presidente de la República

PRESIDENCIA DE LA REPÚBLICA DE PANAMÁ
MINISTERIO DE LA PRESIDENCIA
REGISTRADO

FRANCISCO JAVIER TERRIENTES
Ministro de Salud

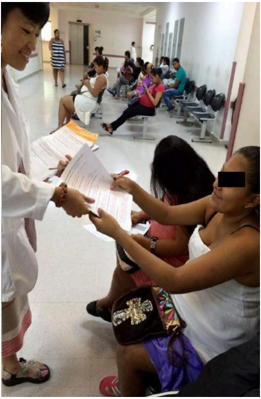

Xuan Li

with Oswaldo Reyes, MD; Mariángela Soberón Felín, JD; Lillian Bodden, MD; Fatima Clouser; Rima McLeod, MD; and Hospital Santo Tomás – Panama; Hospital del Niño – Panama; Instituto de Investigaciones Científicas (INDICASAT) – Panama

Why Diagnose, Prevent and Treat Toxoplasmosis? A study and program to benefit maternal and child health in Panama

Xuan Li[1], Oswaldo Reyes, MD[2], Mariangela Soberon Felin, JD[3], Lillian Bodden, MD[2], Fatima A Clouser[4], Ashtyn Dixon[5], Kenneth Boyer, MD[1], Peter Heydemann, MD[1], Xavier Saez-Llorens, MD[6], Maria Teresa Moreno, MD[6], Sylvia Fernandez Pirla[7], Zuleima Caballero, PhD[8], Claudia Rengifo DVM, PhD[8], Digna Wong, MD[8], J. Rao, PhD[8], J. Motta, MD[8], Eduardo Ortega, MD, PhD[9], Rima McLeod, MD[4]

[1]Rush Medical College, Chicago, IL [2]Hospital Santo Tomas, Panama City, Panama, [3]Toxoplasmosis Program in Panama, Panama City, Panama, [4]The University of Chicago, Chicago IL, [5]Loyola University Medical School, Maywood, IL, [6]Hospital del Nino, Panama City, Panama, [7]Institute of Scientific Investigations and High Technology Services (INDICASAT), Panama City, Panama, [9] Glaxo-Smith Kline, South America .

Introduction

Toxoplasmosis is caused by the parasite *Toxoplasma gondii*. It can be passed to a fetus from the mother infected for the first time during gestation. Transmission of the parasite occurs from consumption of undercooked meat with cysts and ingestion of oocysts shed by cats into the environment[1]. Serious problems can occur in fetuses including neurologic and ophthalmologic damage. While there is limited detailed information about toxoplasmosis in Panama, factors such as tropical weather, high prevalence of feral cats and soil exposure make hospitable conditions for *T. gondii* to remain in environments for prolonged times.

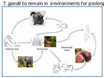

Figure 1. Transmission and Life Cycle

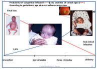

Figure 2. Congenital infection is most severe but least frequently transmitted early in gestation. It is most frequently transmitted late in gestation and is then usually less severe but almost always has sequellae *from Peyron F, 2015*

The 2010 population census revealed that blindness is the second most frequent cause of disability in Panama and toxoplasmosis is a frequent cause of loss of sight in Panama.

In French studies, serologic screening, diagnosis, and treatment during gestation markedly improves outcomes for infants . Efficacy of gestational screening for *Toxoplasma* infection monthly in seronegative patients has been shown resulting in healthy babies with good quality lives for the children and their families.

In the US, the same good outcomes are found when the French algorhithym is applied. Treatment postnatally also improves outcomes but residual damage that occurs from ongoing infection prenatally may be irreversible.

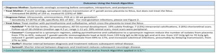
Outcomes Using the French Algorithm

- Diagnose Mother: Systematic serologic screening before conception, intrapartum, and postpartum.
- Treat Mother: If acute serology, spiramycin reduces transmission of *T. gondii* across placenta to fetus, but does not treat the fetus.
  - Untreated 94 (60%) of 154 vs. treated 91 (23%) of 388.
- Diagnose Fetus: Ultrasounds, amniocentesis, PCR at ≥ 18 wk gestation.
  - Sensitivity 37 (97%) of 38; specificity 301 of 301. For mid-gestation infections, please see Figure 2.
- Treat Fetus: Treat mother with pyrimethamine and sulfadiazine, which cross the placenta to treat the fetus.
  - Hohlfeld: N=54 live births; 34 terminations; All 54 normal development. 19% subtle findings: 7 (13%) intracranial calcifications, 3 (6%) chorioretinal scars. Follow-up of 18 children (median age 4.5 yr; range 1-11 yr): 39% retinal scars, most scars were peripheral.
  - Couvreur: Compared to a spiramycin regimen, adding pyrimethamine and sulfadiazine to a spiramycin regimen reduce the number of isolates from placenta from 77% to 42%, reduced *T. gondii*-specific immunoglobulin load at birth from 139 IU/g IgG to 86 IU/g IgG and at 6 mo. from 137 IU/g IgG to 70 IU/g IgG, reduced *T. gondii*-specific IgM prevalence in the neonate from 69% to 17%, and increased subclinical infections, presumably by delaying transmission, from 17% to 30%.
  - Kieffer: Shorter interval between diagnosis and treatment reduces subsequent retinal disease.
  - Syrocot: Shorter interval between diagnosis and treatment reduces subsequent neurologic disease.
- Conclusion: Favorable outcomes with treatment in utero in France and as French Algorithm applied in U.S.

Figure 3 Outcome using French Algorithm

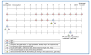

Figure 4. Serologic screening monthly during pregnancy results in prompt diagnosis of this infection and facilitates prompt treatment which improves outcomes Short intervals between diagnosis and treatment are critical

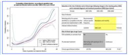

Figure 5. Probability of fetal infection according to gestation age at maternal infection before and after 1992 in the Lyon Cohort . Amniocentesis and gestational treatment of the mother to treat the infected fetus markedly improved outcomes with this change implemented in 1992.

Our hypothesis was that because Panama is the most developed country in Central America, specifically in terms of robust economy, judicial stability, connectivity and medical services, a regional center in Panama will satisfy the needs of region and can provide services for screening and guidelines for treatment of congenital toxoplasmosis to doctors and patients in central America, Caribbean and northern South America.

Methods

Because of proven benefit of gestational screening in prevention of congenital toxoplasmosis in France & as occurs in some best US obstetrical practices, we took following steps to build a program in Panama:

- A law mandating reporting and gestational screening was conceptualized
- Development of Education programs for high school students obstetricians, MFMs, medical students, residents, fellows prepared & presented
- Age specific seroprevalence among 383 pregnant women
- 3 pamphlets created and compared for efficacy.

Results

Define problem: Incidence Study In Panama the incidence of toxoplasmosis is very high. For 383 pregnant women at the Maternity Hospital Santo Tomas in months of June to November 2014, serum antibodies against *Toxoplasma gondii* were determined using an immunoassay (ARCHITECT Toxo IgG and IgM).

Results (continued)

- Toxoplasma IGG positive in serologic screening in ~50 % of pregnant women.
- Age specific seroprevalence determined
- Present in urban and rural areas

Age	Sample	Serology IGG +	Serology IGG %
	No	IGG +	%
11 a 14	3	3	100
15 a 19	76	36	47
20 a 25	149	76	51
26 a 30	94	45	48
31 a 35	46	24	52
36 a 49	15	9	60
Total	383	193	50

Table 1. Age specific seroprevalence
Note: small # of 10-14 years old 100% ;X= 50%.

Residence	Sample	Serology IGG %
	No	%
Urban	353	92
Rural	30	8
Total	383	100

Table 2. Frequency of toxoplasmosis at Hospital Santo Tomas by residence in June-Nov 2014

In Panama incidence of toxoplasmosis is high. Al study at Hospital del Niño (One of the country´s most important pediatric hospitals that serves the maternity of Hospital Santo Tomas, which is the biggest public hospital in the country, and that delivers ~ 16,000 live births every year) shows that approximately 50% of the population has been exposed to *Toxoplasma*, with IGG positive results in serology screening. *Alba Nubia Montenegro Vásquez;. Xavier Sáez Llorens*

Diagnose and Report because it's the law:

- Panama has started initiatives to improve clinical care for pregnant woman, prevent, & treat congenital toxoplasmosis. These initiatives focus on education, research, and recommendations for improved care.
- This provides a foundation for developing in Panama a regional center for screening and treating congenital toxoplasmosis that will serve Panama, the Central America and Caribbean region.

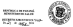

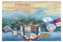

Figure 6: Law from the Ministry of Health with Soberon- Felin et al, 2014

One of the main achievements of these initiatives was the inclusion of congenital toxoplasmosis in October of 2014, in the new legislation for public health events of compulsory notification and investigation, after a specific request presented to the Ministry of Health authorities. Since 2014, all suspected cases, confirmed individual cases and deaths related to congenital toxoplasmosis should be weekly reported to the Ministry of Health, this legislation is mandatory for public and private health institutions, laboratories and all medical personnel in general terms

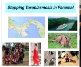

Figure 7. Educational programs in highschools For medical students, residents and practicing Physicians

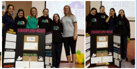

Figure 8. High school students at Science week

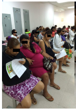

Figure 9. Participants at Maternity Clinic

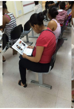

Figure 10. Participants at Maternity Clinic

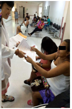

Figure 11. Post Questionnaire

Results(continued)

In our study in September of 2015, in assessing knowledge of toxoplasmosis before & after pamphlet exposure, **A total of 164 pregnant women participated.**

- First response was binary yes/no.
- For initial questionnaire, **76%** had no relevant knowledge.
- A narrative response was scored as understanding 0 correctly, 1 (1-2 questions correct), 2 (3-4 correct), or 3(5-6 question correct).
- For pamphlets 1,2 and 3, **89%,63%,55%** of participants scored 3 respectively, after reading the pamphlet.

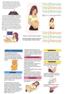

Figure 12. Pamphlet #1- Best Success

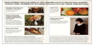

Figure 13. Pamphlet #2, Adapted from USA, in Spanish

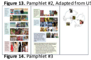

Figure 14. Pamphlet #3

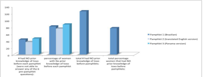

Figure 15. frequency of toxoplasmosis at Hospital Santo Tomas by age in June-Nov 2014

Conclusion

- There was limited knowledge about toxoplasmosis amongst pregnant women in Panama at baseline.
- After educational pamphlets exposure, there was a marked increased understanding of the disease in pregnant women, especially with pamphlet #1.
- After basic educational programs and guidelines for physicians are in place for screening in pregnant women, an inexpensive and rapid point of service test will be the next goal to screen for active infections for all pregnant women in Panama.
- Future goals: France has done this. Panama is moving ahead in leaps and bounds. What are we doing in the United States to prevent this disease?

Future

In the future we will create a Toxoplasmosis center in Panama. We will implement monthly point of service tests to decrease transmission rate by detecting seroconversion, treatment and thereby improve clinical outcomes. We will:

1) Understand demographics and epidemiology of infection, e.g., where the 14 year olds with 100% seropositivity and why.
2) Identify and train prenatal health providers about toxoplasmosis, screening, treatment and prevention
3) Provide point-of-care rapid screening for toxoplasmosis diagnosis
4) Provide treatment with Pyrimethamine, Sulfadiazine, and Folinic acid with instructions, and information on side-effects.

The main outcomes we would like to obtain are: all pregnant women will have prenatal care. They will be screened monthly for toxoplasma antibodies when seronegative originally, and educated about toxoplasmosis. All identified seroconverting pregnant women will be treated properly with medication during their pregnancy. All infected infants will be treated and followed.

References & Acknowledgment

1. Elmore SA, et al. Toxoplasma gondii: Epidemiology, Feline Clinical Aspects, and Prevention. Trends Parasitol. 2010 Apr; 26(4):190-6. PMID:20202907
2. Calero-Bernal R, et al. Congenital toxoplasmosis in wild boar (Sus scrofa) and identification of the Toxoplasma gondii types involved. J Wildl Dis. 2013 Oct; 49(4): 1019-23. PMID: 24502733
3. Vidal P, et al. Prenatal toxoplasmosis diagnosis from amniotic fluid by PCR. Revista da Sociedade Brasileira de Medicina Tropical. Jan-Feb, 2002. 35(1)
4. McLeod R, et al. Management of Congenital Toxoplasmosis. Curr Pediatr Rep (2014) 2:166-194.
5. McLeod R, et al. (2015). Toxoplasmosis in the Fetus and Newborn Infant. In Stevenson D and Sunshine P, Neonatology: Clinical Practice and Procedures. 1st ed. New York: McGraw Hill, 821-7
6. McLeod R, et al. (2015). Diagnosis of Congenital Toxoplasmosis. In Stevenson D and Sunshine P, Neonatology Clinical Practice and Procedures. 1st ed. New York: McGraw Hill, 1297-310.

We thank the University of Chicago for the Global health seed grant, NIH NIAAID DMID grant, and the ACOG Gibbons Medical Student Award

78%. Neonatal resuscitation occurred in 12.7% of fetuses, 17% required noninvasive ventilation and 10.6% intubation. Mild to moderate neonatal hypoxia occurred in 16.9% of the neonates, fetal death in 9.8%, admission to neonatal ICU in 25.5% and neonatal death in 7.8%.

CONCLUSION: APE occurred more frequently antepartum and hypertension was the most common etiology. This severe disease associated with high morbidity and mortality for mother and fetus. Fluid overload seems to have importance in triggering the APE.

Financial Disclosure: *The authors did not report any potential conflicts of interest.*

Downloaded from https://journals-lww-com.proxy.uchicago.edu/greenjournal by [illegible] on 01/25/2020

An Obstetrical Triage Acuity Scale Does Not Predict Hospital Admission [12A]

Casey M. Cosgrove, MD
Department of Obstetrics and Gynecology, Ohio State College of Medicine, Columbus, OH
Tara L. Usakoski, MD, Heather Frey, MD, and Cynthia Shellhaas, MD

INTRODUCTION: To evaluate the application of a five-tiered Obstetrical Triage Acuity Scale (OTAS) in a tertiary care center by assessing the relationship between the triage score and admission rates.

METHODS: A retrospective chart review over a 2 month period in 2014 was conducted. The previously validated OTAS score for obstetrics was assigned to each patient. Seven scoring parameters were used to assign a final score, stratified into 5 categories: 1) resuscitative, 2) emergent, 3) urgent, 4) less urgent, and 5) non-urgent. Characteristics of patients assigned to each scoring group were compared using the Chi-square test. Rates of admission amongst the groups were also compared.

RESULTS: Eight hundred and thirty five patients were included. The mean gestational age of patients was 35.9 weeks. The median number of triage visits was 2 (1–11) with 112 (13.4%) patients classified as triage super users (above 5 visits). The most frequently assigned scores were level 4 (34.7%) and level 2 (32.1%). Only 4 patients were assigned a level 1. Hospital admission rates were significantly associated with OTAS score. Seventy-five percent of level 1, 56.9% of level 4 and 53.8% of level 3 women were admitted. Most level 5 (87.1%) and level 2 (65.9%) visits were discharged to home.

CONCLUSION: The OTAS in our population did not predict hospital admission, because level 2 patients were most likely to be discharged home. Level 3 and level 4 encounters were also likely to be admitted and delivered during the same visit; most term labor admissions will occur in these two levels.

Financial Disclosure: *The authors did not report any potential conflicts of interest.*

Editor's Note: *Poster no. 13A was withdrawn during production.*

Stillbirth: Evaluating Risk Factors and Antepartum Care at Two Midwestern Obstetrical Clinics [14A]

Annabel Mancillas, MD
University of Kansas School of Medicine–Wichita, Wichita, KS
Frank Dong, PhD, Ashley S. Robbins, MD, Jennifer Duong, MPH, Sapphire Garcia, and Darren Farley, MD

INTRODUCTION: Known causes of stillbirth include premature rupture of membranes, congenital anomalies, preeclampsia, and intrauterine growth restriction. Risk factors for stillbirth include age, black or Hispanic ethnicity, history of smoking, history of stillbirth, diabetes, and hypertension. The objective of this study was to determine the stillbirth rate and descriptive factors for clinics serving the a Midwestern, metropolitan community.

METHODS: After receiving IRB approval, a retrospective chart review was conducted at two local, private practice obstetrical clinics. Records were reviewed for patients that experienced a stillbirth between January 1, 2007 to December 31, 2013 using ICD-9 codes: V27.1, 656.4, 674.9, 656.43, 779.9, and 651.6. Cases with a demise before 14 weeks gestation were excluded. Demographics, maternal history, antepartum care, antepartum education, and delivery information were collected.

RESULTS: The stillbirth rate was 4.1 per 1,000 deliveries. Among the 78 patients included in the final analysis, average maternal age was 28.2 years (SD=7.1, range=17 to 45). Most were white (n=48, 64%), never smoked (n=50, 64.9%), and had private medical insurance (n=51, 68%). Average gestational age at delivery was 27.9 weeks (SD=7.9, range=14 to 41). Among the 45 women who experienced stillbirth after 24 weeks gestation, 12 (27.9%) reported decreased fetal movement, and most did not receive education on fetal movement (n=44, 97.8%), safe sleep (n=45, 100%), or breastfeeding (n=43, 93.3%).

CONCLUSION: Prenatal education in regards to fetal movement, safe sleep, and breastfeeding was lacking. A locally designed intervention to detect fetal movement should be developed to reduce stillbirth.

Financial Disclosure: *The authors did not report any potential conflicts of interest.*

Systematic Review of Umbilical Cord Clamping Practices Worldwide [15A]

Kathy C. Matthews, MD
Mt. Sinai SOM, New York, NY
Mayri S. Leslie, EdD, MSN, CNM

INTRODUCTION: Although there is no standard definition of early versus delayed umbilical cord clamping, recent randomized controlled trials demonstrate benefits for both term and preterm infants from the latter. Even a brief delay in cord clamping results in placental transfusion, giving the term neonate approximately 30% more blood volume and the preterm as much as 50%, thereby substantially increasing iron stores within the first few months of life.

METHODS: A search was conducted to identify studies that included information on umbilical cord clamping practices among maternity care providers worldwide. Databases were searched for English publications from 1990 to the present. Articles were included if they contained information on timing of umbilical cord clamping. Surveys and observational studies were acceptable; topical articles and professional guidelines were excluded.

RESULTS: Five of 13 articles were focused on umbilical cord clamping whereas the remaining 8 investigated the management of the third stage of labor. The demographics of the studies varied greatly depending on location and research design. Collectively, our review reports on the practices of 1457 obstetricians, 2124 midwives and 181 other maternity care providers. One study reports on 1175 maternity units in 14 distinct countries. Some studies also used direct observation in reporting their results from 884 births.

CONCLUSION: Delayed cord clamping is rarely practiced despite substantial current evidence in its favor. Many professional organizations even recommend delaying cord clamping as part of their guidelines for the active management of the third stage of labor. Consequently, a change in practice is called for.

Financial Disclosure: *The authors did not report any potential conflicts of interest.*

Toxoplasmosis Education for Pregnant Women in Panama [16A]

Xuan Li
The Toxoplasma Center, Chicago, IL
Mariangela Soberon Felin, JD, Lillian Bodden, MD, Kenneth M. Boyer, MD, Rima McLeod, MD, and Osvaldo Reyes, MD

INTRODUCTION: In 2014, Panama passed a law requiring toxoplasmosis screening for pregnant women and reporting of positive cases. Therefore, it is important to educate pregnant women about toxoplasmosis. The objective of this study is to use educational pamphlets to provide essential information to pregnant women in Panama.

METHODS: In September 2015, we created three different educational pamphlets on toxoplasmosis for pregnant women in Hospital Santo Tomas. We also created a questionnaire to assess knowledge of toxoplasmosis before and after exposure to the pamphlets. The endpoint of this study was the level of knowledge in answering the questionnaire after exposure to the pamphlets.

Copyright © by The American College of Obstetricians and Gynecologists. Published by Wolters Kluwer Health, Inc. Unauthorized reproduction of this article is prohibited.

RESULTS: A total of 164 pregnant women participated. For the initial questionnaire, 76% of the women had no knowledge of toxoplasmosis. We created an understanding scale for those who were able to answer the questionnaire after reading the pamphlets. 0 is answering zero question correctly, 1 is answering one to two questions correctly, 2 is answering three to four questions correctly, and 3 is answering five to six questions correctly. For pamphlet 1, 89% of the participants had an understanding scale of 3 after reading the pamphlet. For pamphlet 2 and 3, 63% and 55% of participants had an understanding scale of 3, respectively.

CONCLUSION: After educational pamphlets exposure, there was an increase in understanding of toxoplasmosis in pregnant women. Pamphlet 1 had the best educational results. With education in place, a rapid point of service test will be the next goal to screen for active infections for pregnant women in Panama.

Financial Disclosure: *The authors did not report any potential conflicts of interest.*

Editor's Note: *Poster no. 17A was withdrawn during production.*

Complications of Rapid Preterm Cesarean Delivery [18A]

Tetsuya Kawakita, MD
Washington Hospital Center, Washington, DC
Sameer Desale, MS, and Uma M. Reddy, MD

INTRODUCTION: There are limited data regarding the impact of rapid cesarean delivery (CD) (incision-delivery time [I-D] less than 2 minutes) on the risk of maternal operative complications.

METHODS: This was a multicenter retrospective cohort study of women undergoing CD between 23 and 31 weeks gestation between 2005 and 2014. Women with multiple gestation, maternal severe conditions (renal failure, cardiopulmonary collapse, acute respiratory distress syndrome, major trauma, and active seizure), and placenta previa/accreta were excluded. Women were categorized based on I-D (rapid group: 2 minute or less or control: greater than 2 minutes). Maternal characteristics and complications were ascertained. Multivariable regression model was created to calculate adjusted *P* value, controlling for maternal characteristics and pregnancy complications.

RESULTS: Of 886 women, 132 women (17.5%) had rapid CD. Rapid group was more likely to be not obese, without previous uterine scar, and to undergo general anesthesia ($P<.05$). Rapid group was less likely to be complicated by hypertensive disease ($P<.05$). In primary CD, rapid CD was associated with increased risk of endometritis (4.8% vs 11.1%; $P<.05$) and intensive care unit (ICU) admission (1.7% vs 6.0%; $P<.05$). Postpartum hemorrhage (5.3% vs 9.5%), transfusion (7.2% vs 12.0%), wound infection (2.4% vs 5.1%) appeared to be increased in rapid CD although they were not significant ($P>.05$). In repeat CD, rapid CD appeared to be associated with increased risk of postpartum hemorrhage (11.5% vs 28.6%) and transfusion (10.1% vs 21.4%) although they were not significant ($P>.05$).

CONCLUSION: Among women who underwent preterm CD, rapid CD was associated with increased risk of maternal complications.

Financial Disclosure: *The authors did not report any potential conflicts of interest.*

"Recent Vaginal Exam" No Longer a Contraindication to Fetal Fibronectin Sampling: A Prospective Study [19A]

Steven Swancoat
Wyckoff Heights Medical Center, New York, NY

INTRODUCTION: Threatened Preterm labor (TPL) accounts for 1,000's of unnecessary yearly admissions with an average cost of $14,000 per patient. With a 98% specificity, Fetal Fibronectin (FFN) is a very powerful test that can safely rule out threatened preterm labor with nearly 99% accuracy for up to two weeks. However the company states that a "recent vaginal examination" is a contraindication as it may lead to a false positive. Yet they submit no clinical research to support this statement.

METHODS: Two FFN sampling were collected from 18 patients whom met the indicated criteria for TPL. Criteria included single gestation between 24–33 6/7 weeks, no sexual intercourse or vaginal exam within past 48 hours, or showing signs or symptoms of preterm labor. Patients were initially sampled for FFN, vaginal exam performed, following a second FFN sampling was collected. The two samples were then compared against each other and patients were followed until they went into labor.

RESULTS: Of the 18 patients, (14) patients tested FFN neg/neg. (2) positive/neg, (1) neg/positive and (1) positive/positive, Of 15 initial neg results, only one tested positive after vaginal exam, yielding a 6% false positive. Of three who tested initially positive, two retested neg, Neither delivered before 37 weeks.

CONCLUSION: Though a small sample size, study shows "recent vaginal exam" prior to FFN sampling yields a very low rate for false positives and should no longer be considered a contraindication to testing. Furthermore with an initial positive test result, a repeat test should be performed for true negative.

Financial Disclosure: *The author did not report any potential conflicts of interest.*

Does Sickle Cell Trait Affect Adverse Pregnancy Outcomes? [20A]

Uzma F. Naeem, MD
University of Tennessee OBGYN Residency, Memphis, TN
Luis M. Gomez, MD, Logan K. Williams, Danielle L. Tate, MD, and Rebecca A. Uhlmann, PhD

INTRODUCTION: While data and pregnancy outcomes are well established, previous data published on sickle cell trait and pregnancy outcomes have been controversial with some reports showing a significant association with preeclampsia. Our study aims to evaluate the risk of adverse pregnancy outcomes in pregnant patients with sickle cell trait who underwent prenatal care at our tertiary-care institution.

METHODS: Database obtained from LabCorp of all patients from the Regional One Health Outpatient Clinic who underwent hemoglobin electrophoresis obtained from July 2012 to April 2013. Chart review was performed to collect medical history, laboratory data, delivery information and pregnancy outcomes. Data were analyzed with SPSS Version 22 software. ANOVA, chi-square test and Bonferroni correction test were used where applicable.

RESULTS: Of 350 patients, 102 were excluded for lack of delivery information, multiple gestation, and delivery less than 20 weeks. A comparison of 223 patients with normal hemoglobin and 16 patients with HbAS showed no statistically significant difference in pregnancy outcomes including Preeclampsia or HELLP Syndrome, gestational hypertension, fetal growth restriction, preterm delivery, gestational diabetes, premature rupture of membranes or cesarean delivery. Furthermore, in our cohort, there was no increased risk of bacteriuria diagnosed in the outpatient setting. A subanalysis of patients with HbAS who tested positive for bacteriuria failed to show increased risk for fetal growth restriction.

CONCLUSION: In our cohort, carriers of HbAS are not at increased risk for adverse pregnancy outcomes or bacteriuria. Fetal growth restriction was not more prevalent in our cohort even in women with bacteriuria when compared to controls.

Financial Disclosure: *The authors did not report any potential conflicts of interest.*

Early Postpartum Sleep and Substantial Postpartum Weight Retention in Women with Gestational Diabetes Mellitus [21A]

Lisette Davidson, MD, MPH
Kaiser Permanente Oakland, Oakland, CA
Yvonne Crites, MD, Juan Carlos LaGuardia, and Erica P. Gunderson, PhD, MS, MPH

INTRODUCTION: In adults, sleep duration has been linked to obesity, coronary artery disease, and type 2 diabetes. Less sleep at 6 months postpartum has been linked with higher weight retention. This study evaluates postpartum sleep and 1-yr weight retention among women with gestational diabetes mellitus (GDM).

METHODS: Prospective cohort study of 1,035 women with GDM who delivered a singleton, live birth in northern California and enrolled in the Study of Women, Infant Feeding and Type 2 Diabetes

Copyright © by The American College of Obstetricians and Gynecologists. Published by Wolters Kluwer Health, Inc. Unauthorized reproduction of this article is prohibited.

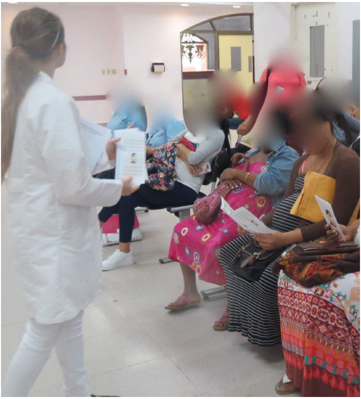

Sharon Heichman

with Mariángela Soberón Felín, JD; Oswaldo Reyes, MD; Rima McLeod, MD; and Hospital Santo Tomás – Panama

THE EFFECTIVENESS OF EDUCATIONAL MATERIALS ABOUT TOXOPLASMOSIS TO BENEFIT MATERNAL AND CHILD HEALTH IN PANAMA

Sharon Heichman[1], Mariangela Soberon Felin, JD[2], Xuan Li[3], Rima McLeod, MD[4]
[1]Pritzker School of Medicine, Chicago, IL, [2]Tozoplasmosis Program, Panama City, Panama, [3]Rush Medical College, Chicago, IL, [4]Toxoplasmosis Center, University of Chicago, Chicago, IL

THE UNIVERSITY OF CHICAGO Center for Global Health

Introduction

➢ Toxoplasmosis is a disease caused by the parasite *Toxoplasma gondii*.

➢ Humans can be infected through consumption of undercooked meats, inadvertent contact with the feces of domestic cats, or ingestion of contaminated water that contains oocysts.

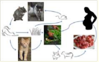

Figure 1. Toxoplasma life cycle and transmission

➢ *T. gondii* can cause congenital infection if a mother is infected for the first time during pregnancy. Typically, maternal infection occurs without symptoms; however, serious problems can manifest in the fetus from untreated congenital infection including neurologic and ophthalmic presentations.

➢ In Panama, factors such as tropical weather and a high prevalence of feral cats results in hospitable conditions for *T. gondii* to remain in the environment.

➢ The burden of disease in Panama is significant. The seroprevalence of *T.gondii* in women of childbearing age is estimated to be 50%.

➢ Health education has been shown to make a measurable difference in toxoplasmosis transmission rates. In France, following implementation of education about toxoplasma infection, transmission rates were reduced by approximately 50%.

Objectives

➢ To develop a highly informative and visually appealing educational pamphlet about toxoplasmosis targeted toward pregnant women in Panama containing information about what toxoplasmosis is, the fetal sequelae of infection, how to prevent *T. gondii* infection, and the importance of being screened for infection during pregnancy

➢ To compare the effectiveness of this new pamphlet with an older version that was shown to be effective in educating pregnant women in a previous study

Methods

➢Pregnant women recruited from the waiting room for obstetric clinic appointments at Hospital Santo Tomás, the largest public hospital in Panama

➢Women first completed a pre-test questionnaire to assess their preexisting knowledge about toxoplasmosis

➢Participants were randomized to receive the old version of the toxoplasmosis educational pamphlet (developed in Brazil) or the new version of the pamphlet that we developed specifically for use in Panama

➢Participants then completed the same questionnaire again as a post-test to assess what they had learned from the educational pamphlet

➢Participant ages were recorded

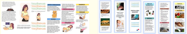

Figure 2 and 3. Old (left) and new (right) version of pamphlet

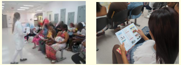

Figure 4 and 5. Participants reading pamphlets and completing questionnaires in the waiting room at Hospital Santo Tomás

Results

➢A **total of 156** sets of pre- and post- test questionnaires completed by pregnant women were analyzed. More women participated but were not included because they did not complete the second questionnaire, their handwriting was illegible, etc.

➢In both the old and new pamphlet groups, **67% of women had no relevant preexisting knowledge about toxoplasmosis**, answering 0 pre-test questions correctly.

Results (continued)

➢The most often missed questions were about what *T. gondii* is and the symptoms of infection. A vast majority of participants answered questions about how to prevent infection and how toxoplasmosis is diagnosed (the most important questions for primary prevention and treatment) correctly.

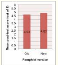

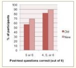

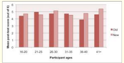

Figure 6-7 (top). Mean post-test scores; percentage of participants scoring 5 or 6 or 4, 5, or 6 (out of 6) on post-test

Figure 8. Post-test scores by age cohort

Conclusions

➢Participants' knowledge about toxoplasmosis improved considerably after reading either pamphlet

➢Both groups went from a mean score of between 0 and 1 to between 4 and 5 post-test questions correct out of 6

➢At least 4 of 6 post-test questions were answered correctly by 82% of the old pamphlet group and 89% of the new pamphlet group.

➢While no clear trend emerges for participants 35 years old or under, it appears that the new version of the pamphlet is more effective for pregnant women over 36.

Acknowledgements

Fatima, Ian (authors?)
Dr. Oswaldo Reyes, Dr. Carmen Charter
Funding: Margaret P. Thorp Scholarship Fund

Methods:
Pregnant women were recruited from the waiting room for high-risk obstetric clinic appointments at Hospital Santo Tomás, the largest public hospital in Panama. Women first completed a pre-test questionnaire to assess their preexisting knowledge about toxoplasmosis. Participants were then randomized to receive and read the old version of the toxoplasmosis educational pamphlet (developed in Brazil) or the new version of the pamphlet that we developed specifically for use in Panama. Both pamphlets were written in Spanish and participant ages were recorded. The pamphlet was then taken back while participants completed the same questionnaire again as a post-test to assess what they had learned from the educational pamphlet.

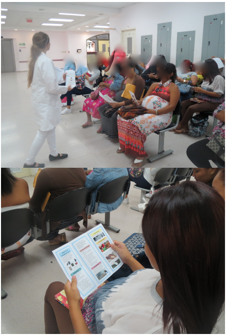

Figure 1. Participants reading pamphlets and completing questionnaires in the waiting room at Hospital Santo Tomás

- Evitar la presencia de moscas, cucarachas y hormigas cerca de comida y agua.
- Cocinar bien las carnes, hervir la leche y no comer embutidos crudos (por ejemplo salchichas) de dudosa procedencia.
- No probar la carne cruda.

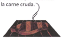

- Las mujeres que contraigan la enfermedad durante el embarazo, deben ser tratadas por un especialista en el tema para tratar de impedir la transmisión del parásito su bebé.
- Si usted tiene gatos, debe alimentarlos con alimento seco o carnes bien cocidas. Pida a alguien más para limpiar las heces de los gatos, porque son los únicos animales que pueden eliminar el Toxoplasma gondii través de las heces, si han sido infectados. Tomando algunas precauciones que usted puede vivir con su gato.

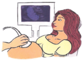

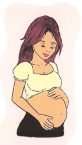

Acuda a sus citas de control prenatal!

Cualquier consulta o duda comuniquese con su ginecólogo o médico tratante

Toxoplasmosis
Toxoplasmosis
Toxoplasmosis

¿Qué es y cómo prevenir?

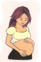

Toxoplasmosis
Toxoplasmosis
Toxoplasmosis

¿QUÉ ES LA TOXOPLASMOSIS?

Es una enfermedad zoonótica (enfermedad que afecta a los seres humanos y a los animales). Es causada por un protozoario llamado Toxoplasma gondii. Es conocida también "enfermedad de gato".

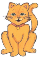

SÍNTOMAS

En la mayoría de los casos se presentan síntomas leves o los pacientes la pueden cursar de manera desapercibida, aunque también pueden ocurrir complicaciones graves en algunos casos.

En la fase aguda de la enfermedad puede ser confundida con la gripe,púes pueden aparecer síntomas como: dolor de cabeza, malestar general, fiebre, dolores musculares, dolor en las articulaciones, fatiga, irritación e inflamación de ganglios linfáticos en la región de la ingle, el cuello y la axila.

En algunos casos, puede ocurrir una reducción o incluso la pérdida total de la visión. Si una mujer se infecta durante el embarazo puede pasar al bebé y causar lesiones graves.

FUENTES DE INFECCIÓN

- Consumo de carne cruda o poco cocida.
- Beber leche cruda y consumir huevos crudos o poco cocidos.
- Beber agua potable contaminada con formas resistentes del parásito.
- Llevarse a la boca las manos sucias de tierra o arena.
- Consumo de vegetales, frutas y verduras crudas sin lavar.
- Transmisión de la madre con infección aguda para el feto durante el embarazo.
- En raras ocasiones a través de transfusiones de sangre y trasplantes de órganos.

TRATAMIENTO

Sólo el médico puede evaluar si tratar o no la infección.

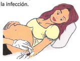

DIAGNÓSTICO

El diagnóstico de la enfermedad se realiza mediante análisis de sangre. Es muy importante que las mujeres embarazadas no carezcan de consultas-durante el período prenataly deben realizarse exámenes con regularidad.

PREVENCIÓN

- Lavar con agua limpia las frutas y verduras crudas antes de comerlas.

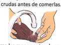

- Lavarse las manos antes de comer y después de tocar tierra, verduras, animales y cajas de basura.

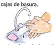

- Usar guantes al manipular cajas de suelo y arena.

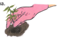

Figure 2. Old version of educational pamphlet (developed in Brazil)

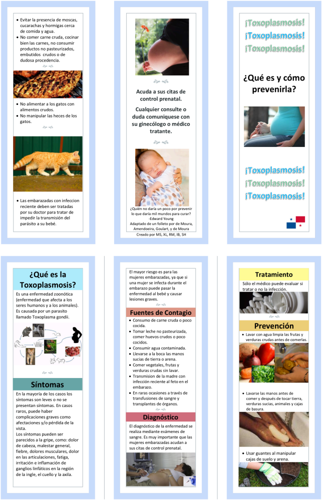

Figure 3. New version of educational pamphlet (developed for Panamanian audience)

Results:
A total of 156 sets of pre- and post- test questionnaires completed by pregnant women were analyzed. Approximately 50 other women participated but were not included because they did not complete the post-test (~42). they were referring to a pamphlet during the post-test (6), or their handwriting was illegible (2). In both the old and new pamphlet groups, 67% of women had no relevant preexisting knowledge about toxoplasmosis, answering 0 pre-test questions correctly. The most often missed post-test questions were about what *T. gondii* is and the symptoms of infection. A vast majority of participants answered questions about how to prevent infection and how toxoplasmosis is diagnosed (the most important questions for primary prevention and treatment) correctly.

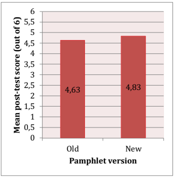

Figure 3. Mean post-test scores by group (two sample t-test, p=0.26)

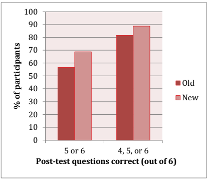

Figure 4. Percentage of participants scoring 5 or 6 or 4, 5, or 6 (out of 6) on post-test.

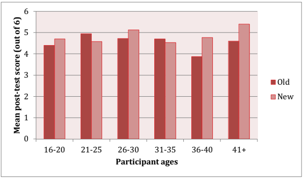

Figure 5. Mean post-test scores by age cohort.

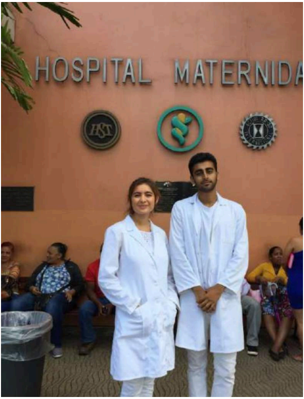

Aliya Moreira and Abhinav Pandey
with Rima McLeod, MD; Mariángela Soberón Felín, JD; Kanix Wang, PhD; Oswaldo Reyes, MD; and Hospital Santo Tomás – Panama; Hospital San Miguel – Panama

University of Chicago Center for Global Health
Summer Research Fellowship Written Report
September 25th 2016

Investigating Social and Infrastructural Parameters Concerning Toxoplasmosis in Panama: Part 1 (the second part of this paper is in Manuscript III concerning Spatial epidemiology and risk factors)

ALIYA F. MOREIRA and ABHI PANDEY
University of Chicago

(Know what you chose is a student study of educational pamphlets for pregnant women as hard copy **pamphlet or digital information)**

Overview

This report summarizes a two-project research study that took place in July and August of 2016. Researchers Aliya Moreira and Abhi Pandey traveled on a University of Chicago Center for Global Health Summer Research Fellowship to Panama. In Panama City, they worked at the maternity ward of the largest public hospital, Hospital Santo Tomas, and in Hospital San Miguel, to administer surveys to and collect data from pregnant patients. In order to target the high rates of toxoplasmosis incidence in Panama, their projects seek to:

1) Determine cost and impact-effective educational strategies to inform Panamanians about the risks of toxoplasmosis and the methods of transmission.

2) Examine the rates of screening that take place in the country's public health centers in order to facilitate the enforcement of universal screening; additionally to examine areas of high incidence in order to determine possible methods of transmission of the disease.

In this supplement we present Part o f the student study)knowing what you choose. Part 2 is presented in the supplement to part III of the series of manuscripts about spatial epidemiology and risk factors.

Abstract 1

The objective of this study is to determine if an online educational toxoplasmosis campaign would be effective in Panama. To do so, we used an educational pamphlet tested for effectiveness previously by Li et al (2016) and a PDF version of the same pamphlet that was displayed on an iPhone. We also used Li et al (2016)'s pre-test and post-test questionnaire to assess subject knowledge of toxoplasmosis before and after reading the pamphlet. A total of 214 pregnant women participated in the study. Of the 6 questions of the questionnaire, the overall average pre-test score was 0.78 questions correct, with an average overall post-test score of 4.05. For both groups, there was a statistically significant increase in knowledge of toxoplasmosis from reading the pamphlet. However, the change over time was statistically different between the two groups, with the Brochure group having a significantly greater increase than the PDF group. While these results suggest a pamphlet is more effective in a hospital setting, it also provides evidence that a phone campaign would be a cheaper and still effective method to increase toxoplasmosis awareness in the country.

Introduction-1

Panama has one of the highest rates of toxoplasmosa infection in Latin America with a seroprevalence of 50% in 10-year-olds and 90% in 60 year-olds (Sousa et al, 1988). Therefore, it is important to educate pregnant mothers about the causes and risks of the disease in order to prevent transmission. In a previous study, Li et al (2016) compared the educational effectiveness of three different versions of a paper toxoplasmosis educational pamphlet by measuring the increase in knowledge caused by exposure to the pamphlet to pregnant women patients in Panama's Hospital Santo Tomas. However, while Li et al (2016) determined the most effective version of the pamphlet of the three to be one designed by Sharon Heichman and Ian Begeman, we found that printing costs for the color pamphlets were very high—approximately $3 per pamphlet. Therefore, in this study we hoped to investigate the potential that an online awareness campaign has in educating pregnant women about toxoplasmosis. In order to accomplish this, we conducted an educational comparison using the most successful version of the pamphlet that Li et al determined in her study, and compared the effectiveness of a paper version of the pamphlet with a phone PDF version of the same pamphlet. Our hypothesis is that the paper pamphlet and PDF versions will be equally effective in educating Panama's pregnant women, which would then provide empirical evidence to support an initiative for a more cost-effective online toxoplasmosis awareness campaign.

Methods 1

Participants

Our participant sample was a convenience sample consisting of 214 pregnant women in

attendance at the Hospital Santo Tomas in Panama City, Panama. Patients were surveyed from the External Consult room on the first floor, where patients come for check-ups and emergency visits, as well as from the In-Patient Ward on the seventh floor, where patients stay during their pregnancy for maintenance of longer-term conditions like hypertension. Demographic information was not obtained from subjects. Subjects were randomized into the PDF (P) and Brochure group (B) through researcher-blind administration of one of four pre-test clipboards that either contained a paper marked B or P for condition. Due to the fact that participants were randomly assigned to condition, the number of participants in each condition varied slightly. The Brochure group contained 105 participants, and the PDF group contained 109 participants.

Stimuli

The procedure involved the use of two documents:

1) The Pre-Test/Post-Test survey was a replica of the same survey used by Li et al (2016) in her comparison of the effectiveness of different formats of the pamphlet. We used the same questions so that success of the use of the pamphlet could be compared with the results of her study as well. It was administered as a single double-sided survey with the same questions typed onto both the front and back. Each side contained six open-ended response questions.
2) The Pamphlet (Figure 1) used was the same pamphlet designed by Sharon Heichman and Ian Begeman that was determined by Li et al (2016) to be the most effective in educating Panamanian pregnant mothers about toxoplasmosis out of the three versions that she compared in her study. The Pamphlet contained sections on defining toxoplasmosis, symptoms, sources of disease contraction, diagnosis, treatment, and prevention. The

Pamphlet was used in two formats:

A) Brochure: A printed version of Li et al (2016)'s pamphlet.

B) PDF: Li et al (2016)'s pamphlet converted to a PDF form and uploaded onto an iPhone 6 or 6S. This version was shown to participants through the iBooks app to allow for easy enlargement of text or images and turning of pamphlet pages.

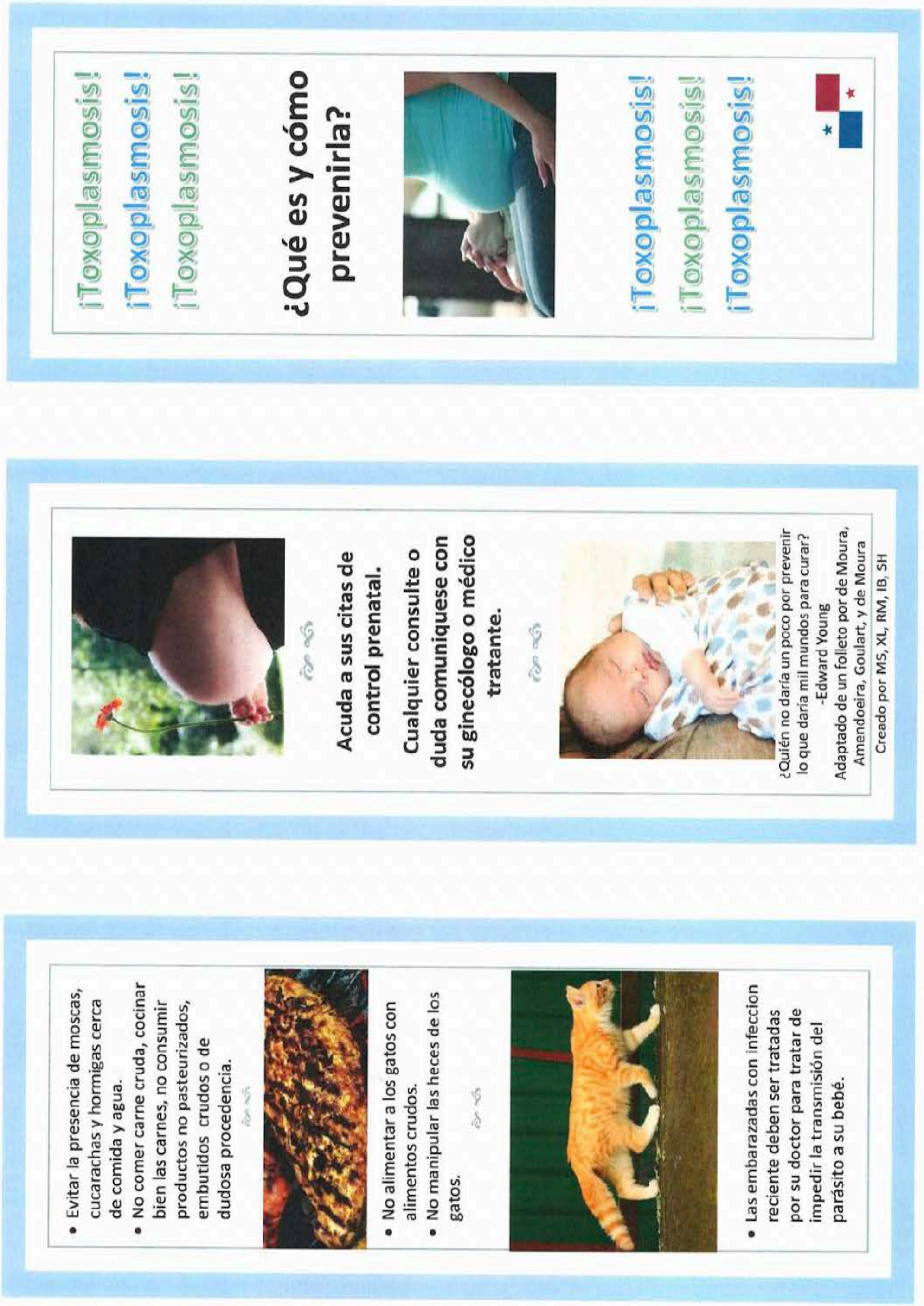

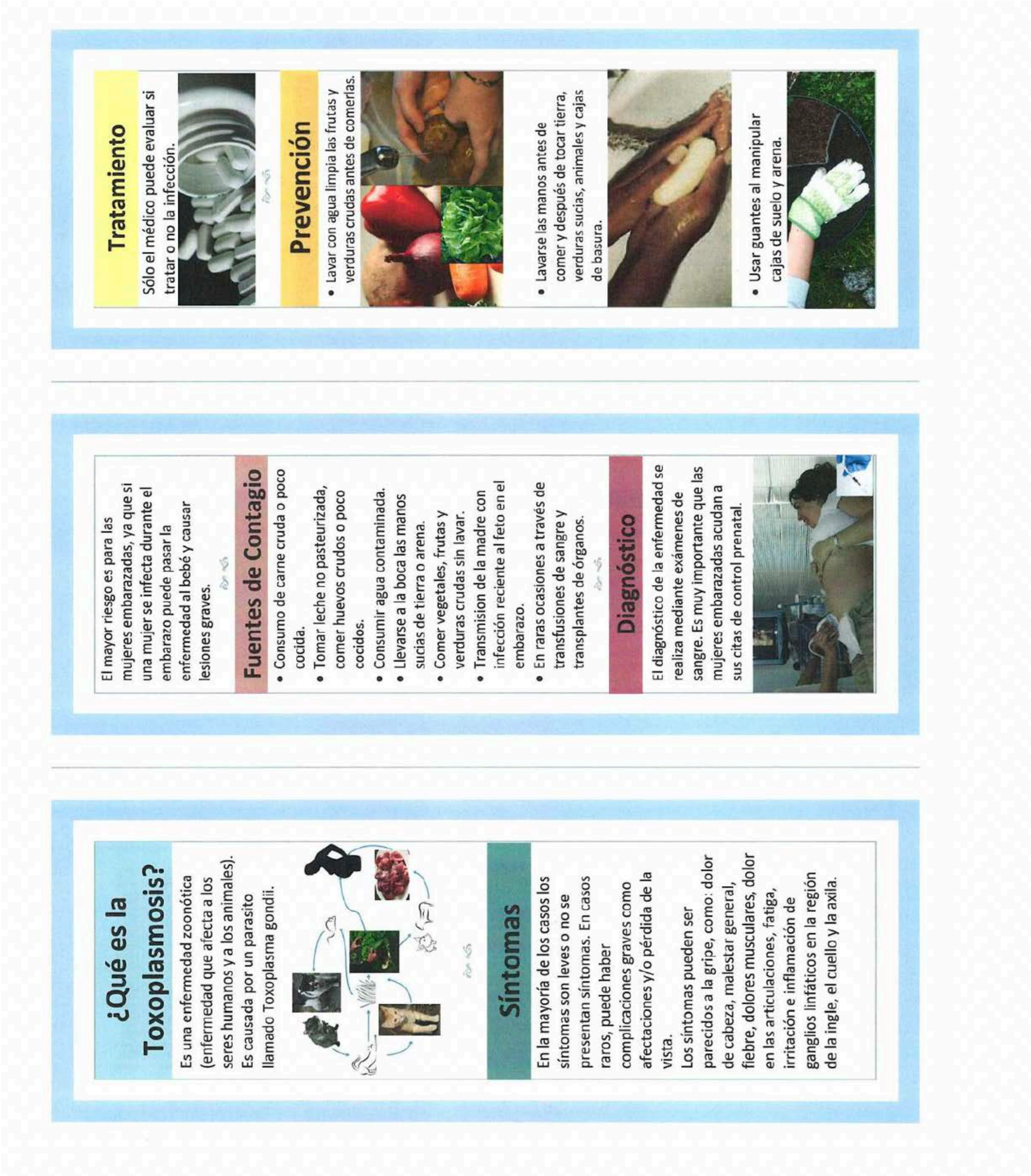

Figure 1. Pamphlet that was displayed to participants in both physical Brochure and PDF form.

Procedure

Research investigators invited women in the Maternity Ward to participate in a short, ten minute study about toxoplasmosis that included a pre-test, a short reading, and a post-test survey. Participants who agreed were then randomly handed one of four clipboards and asked to fill out the pre-test survey to measure their initial knowledge. The clipboards contained double-sided sheets of paper with the pre-test survey on the front, and the post-test survey on the back. The back of the sheet, with the post-test survey, was labeled with the format of the pamphlet that the participant would then receive. Upon receiving the pre-test clipboard back, the researcher would then look to determine which condition was labeled and administer the appropriate version of the pamphlet. The participant were handed either the phone PDF or the paper pamphlet, and be asked to read through the six panels. Upon completion of reading, they were handed back their initial clipboard, flipped to show the post-test survey, and asked to complete the six questions. Following completion of the survey, participants were debriefed that the results of the investigation were used to compare the effectiveness of different educational materials in the country. They were then invited to take home a copy of the paper Brochure regardless of which intervention they had received.

Results 1

In order to interpret our results, we first summed the totals of answers correct for each pre-test and each post-test for each group using the answers sheet utilized by Li et al (2016). We averaged the totals correct in the pre-test for the Brochure group and then for the PDF group, and

subsequently found the average number correct for the post-tests in each group. The results are summarized in the table below.

	Mean Pre-Test Score	Mean Post-Test Score
**Brochure Group**	0.39	4.88
**PDF Group**	1.15	4.77

Table 1. Mean Pre and Post-Test # of questions correct for both Brochure and PDF groups.

Due to the fact that the average pre-test score was very different between the Brochure group and the PDF group, we ran analysis and found that this difference was statistically significant. After comparing the groups, we additionally found that both educational intervention formats resulted in a statistically significant increase in knowledge between pre-test and post-test ($p<0.001$ for both). However, based on a group by time interaction in a mixed-effects regression model, we found that the change over time was statistically different between the two groups ($p<0.001$) with the Brochure group having a significantly greater increase than the PDF group.

Discussion 1

The data from this experiment indicates that both a physical Brochure and online PDF are effective in informing Panamanian pregnant women about toxoplasmosis. However, it also suggests that a physical Brochure pamphlet is statistically significantly more effective.

Overall, these results do not support our hypothesis that a paper Brochure and phone PDF will be equally effective in educating Panamanian pregnant women about toxoplasmosis. However, the results do support the objective of the experiment, which was to examine the effectiveness of the more cost-effective PDF version. Our results indicate that the target

demographic of our educational intervention is able to navigate the iPhone PDF successfully. The format of the information did not result in the knowledge being inaccessible to the Panamanian women who are most impacted by the disease.

Additionally, the information from the physical Brochure was more effective in educating participants. However, Li et al (2016) specifically tested different formats of physical paper brochures in order to determine a design that was most effective in educating the target demographic. What this suggests is that it is possible that the design of the pamphlet, while ideal for a Brochure, may not be most effective for a phone PDF. This is a key limitation of our study due to the fact that the PDF used was simply an uploaded version of the phone Brochure. A more effective comparison could be performed in future research with a phone PDF that is formatted to be more appropriate for phone use. In such a study, it is quite possible that a differently formatted PDF version of the Brochure would be equally or more impactful in increasing participant knowledge.

Another potential area to investigate concerning the potential phone educational interventions possess is an interactive display. Brochures are physically larger, with clearer text and images. In order to balance that out, one potential is for phone information to be presented in a more interactive way in order to best utilize the format. Including features with in-program questions and demonstrations would invoke the testing effect and increase retention of information learned through this format. Additionally, participants would be able to keep the phone version on their phones for a future reference source that is much less easily misplaced. Future research might attempt this same experiment with an interactive phone informational source in order to check for this discrepancy.

Conclusion

In conclusion, we observed that both PDF and physical Brochures are effective methods of education for the target demographic of Panamanian pregnant women. While the physical Brochure method proved better overall, there are a few reasons suggested above that qualify this result. The specific PDF version that we experimented with was not specifically designed to be used on a phone, and a version developed specifically with phone use, possibly with interactive educational features to take advantage of the format, could potentially be even more effective. Overall, our study provides support for the effectiveness of an online educational toxoplasmosis awareness campaign in Panama. Given the cost differential between the one-time cost of developing a phone PDF, email, or text message blast compared to the $3/person cost of printing, it is definitely worth exploring different formats of online educational campaigns to find more effective formats and make use of the potential this study has proven for education that online formats have.

References

1. Li, Xuan, Mariangela Felin Soberon, Lillian Bodden MD, Kenneth Boyer M., Rima McLeod, and Osvaldo Reyes. "Toxoplasmosis Education for Pregnant Women in Panama." *Obstetrics & Gynecology* 127.5 (2016): 15S-6S. Web.
2. McLeod, Rima. "An Overview of Toxoplasmosis: Cause, Prevalence, and Consequences." *TRI: Information about Toxoplasmosis*. Toxoplasmosis Research Institute and Center, n.d. Web. 15 Sept. 2016.
3. Sousa, O.E., R.E. Saenz, J.K. Frenkel. “Toxoplasmosis in Panama: A 10-year study.” *American J Trop Med Hygiene 38 (1988), pp. 315-322.*

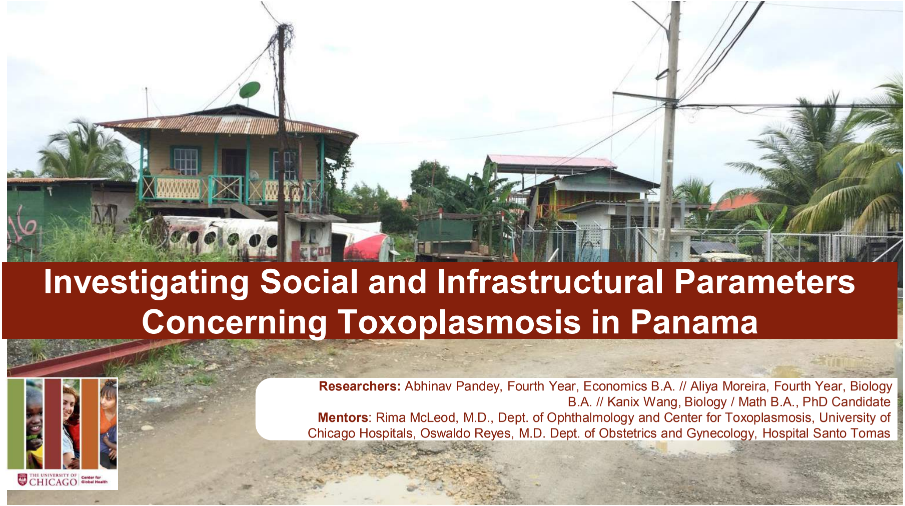

Investigating Social and Infrastructural Parameters Concerning Toxoplasmosis in Panama

Researchers: Abhinav Pandey, Fourth Year, Economics B.A. // Aliya Moreira, Fourth Year, Biology B.A. // Kanix Wang, Biology / Math B.A., PhD Candidate

Mentors: Rima McLeod, M.D., Dept. of Ophthalmology and Center for Toxoplasmosis, University of Chicago Hospitals, Oswaldo Reyes, M.D. Dept. of Obstetrics and Gynecology, Hospital Santo Tomas

THE UNIVERSITY OF CHICAGO Center for Global Health

Toxoplasmosis Overview

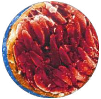

Spread by the protozoan *Toxoplasma gondii*

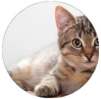

Cat oocysts: major source of transmission

33% Most prevalent parasite in the world, lying dormant in over 2,000,000,000 people.

50% Prevalence rate in Panama

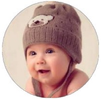

Congenital transmission can cause brain and eye damage

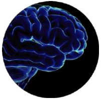

Seroprevalence associated with neurologic disease / seizures

Investigating Effectiveness of Digital Information Distribution

Objective: Inform Potential Digital Public Health Campaign

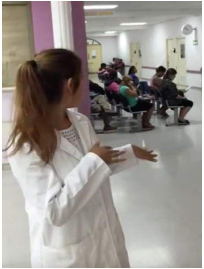

Many Panamanians now own smartphones; digital media costs much less than physical brochures

Pregnant women were first administered a pretest with questions on toxoplasmosis. Afterwards, women were randomly handed either a paper brochure or a phone with pdf

After women finished reviewing information, the information medium was removed and the posttest was administered

Survey Results

Final Avg Score (Brochure): 4.88
Final Avg Score (PDF) 4.77

Government should feel confident launching digital campaign

Formats do not significantly differ in their informative power (p > __)

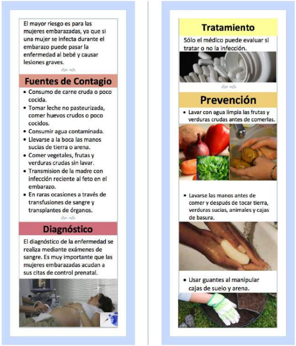

El mayor riesgo es para las mujeres embarazadas, ya que si una mujer se infecta durante el embarazo puede pasar la enfermedad al bebé y causar lesiones graves.

Fuentes de Contagio

- Consumo de carne cruda o poco cocida.
- Tomar leche no pasteurizada, comer huevos crudos o poco cocidos.
- Consumir agua contaminada.
- Llevarse a la boca las manos sucias de tierra o arena.
- Comer vegetales, frutas y verduras crudas sin lavar.
- Transmision de la madre con infección reciente al feto en el embarazo.
- En raras ocasiones a través de transfusiones de sangre y transplantes de órganos.

Diagnóstico

El diagnóstico de la enfermedad se realiza mediante exámenes de sangre. Es muy importante que las mujeres embarazadas acudan a sus citas de control prenatal.

Tratamiento

Sólo el médico puede evaluar si tratar o no la infección.

Prevención

- Lavar con agua limpia las frutas y verduras crudas antes de comerlas.
- Lavarse las manos antes de comer y después de tocar tierra, verduras sucias, animales y cajas de basura.
- Usar guantes al manipular cajas de suelo y arena.

Impact

- Incentivizes improvements in maternal care for pregnant women and children
- Identifies key townships and demographics for future investigations
- Informs forthcoming digital public health campaign
- Identified 14 acute women who should receive immediate aid

- Evitar la presencia de moscas, cucarachas y hormigas cerca de comida y agua.
- No comer carne cruda, cocinar bien las carnes, no consumir productos no pasteurizados, embutidos crudos o de dudosa procedencia.

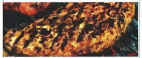

- No alimentar a los gatos con alimentos crudos.
- No manipular las heces de los gatos.

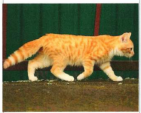

- Las embarazadas con infeccion reciente deben ser tratadas por su doctor para tratar de impedir la transmisión del parásito a su bebé.

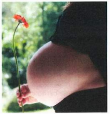

Acuda a sus citas de control prenatal.

Cualquier consulte o duda comuniquese con su ginecólogo o médico tratante.

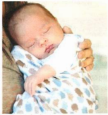

¿Quién no daría un poco por prevenir lo que daría mil mundos para curar?
-Edward Young
Adaptado de un folleto por de Moura, Amendoeira, Goulart, y de Moura
Creado por MS, XL, RM, IB, SH

¡Toxoplasmosis!
¡Toxoplasmosis!
¡Toxoplasmosis!

¿Qué es y cómo prevenirla?

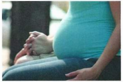

¡Toxoplasmosis!
¡Toxoplasmosis!
¡Toxoplasmosis!

¿Qué es la Toxoplasmosis?

Es una enfermedad zoonótica (enfermedad que afecta a los seres humanos y a los animales). Es causada por un parasito llamado Toxoplasma gondii.

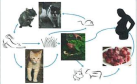

Síntomas

En la mayoría de los casos los síntomas son leves o no se presentan síntomas. En casos raros, puede haber complicaciones graves como afectaciones y/o pérdida de la vista.
Los síntomas pueden ser parecidos a la gripe, como: dolor de cabeza, malestar general, fiebre, dolores musculares, dolor en las articulaciones, fatiga, irritación e inflamación de ganglios linfáticos en la región de la ingle, el cuello y la axila.

El mayor riesgo es para las mujeres embarazadas, ya que si una mujer se infecta durante el embarazo puede pasar la enfermedad al bebé y causar lesiones graves.

Fuentes de Contagio

- Consumo de carne cruda o poco cocida.
- Tomar leche no pasteurizada, comer huevos crudos o poco cocidos.
- Consumir agua contaminada.
- Llevarse a la boca las manos sucias de tierra o arena.
- Comer vegetales, frutas y verduras crudas sin lavar.
- Transmision de la madre con infección reciente al feto en el embarazo.
- En raras ocasiones a través de transfusiones de sangre y transplantes de órganos.

Diagnóstico

El diagnóstico de la enfermedad se realiza mediante exámenes de sangre. Es muy importante que las mujeres embarazadas acudan a sus citas de control prenatal.

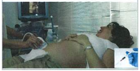

Tratamiento

Sólo el médico puede evaluar si tratar o no la infección.

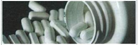

Prevención

- Lavar con agua limpia las frutas y verduras crudas antes de comerlas.

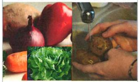

- Lavarse las manos antes de comer y después de tocar tierra, verduras sucias, animales y cajas de basura.

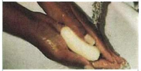

- Usar guantes al manipular cajas de suelo y arena.

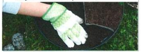

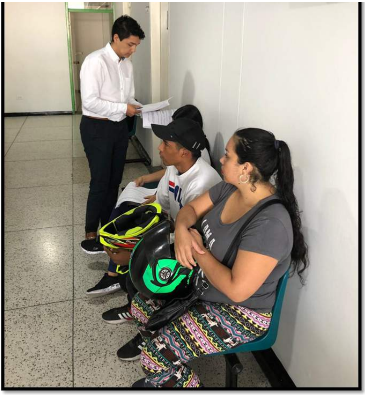

José Sánchez

with Catalina Raggi; Laura Lorena López; Richard James Orozco González, MD; Jorge Gómez Marín, MD; Rima McLeod, MD; and Hospital del Sur – Colombia; Hospital San Juan de Dios – Colombia; Universidad del Quindío – Colombia

Education as a Critical Tool to Aid in the Fight against Toxoplasmosis

Jose Sanchez, Catalina Raggi, Laura Lorena Lopez, Richard James Orozco Gonzalez MD, Jorge Gomez Marin MD, Rima McLeod MD
The College at the University of Chicago

Introduction

Background

- Toxoplasmosis is a disease caused by the protozoan *Toxoplasma gondii*. It is the most prevalent parasite in the world, affecting more than 1/3 of the world's population.
- Cat oocysts are the major source of transmission, but humans can come into contact with them inadvertently through ingestion of contaminated water, dirt floors,, and improperly washed produce.
- Cysts in meat that is undercooked is another means of transmission. These cysts are part of the dormant chronic infection.
- Congenital infection can occur if a mother is infected for the first time during her pregnancy. This can cause serious problems for the fetus including brain and eye damage, and sometimes death.
- In Colombia, factors like the tropical weather, water contamination, and high prevalence of feral cats contribute to the high incidence of *Toxoplasma gondii* infection and toxoplasmosis in the country
- El Quindio Colombia toxoplasmosis seroprevalence rates are over 50%, one of the highest rates in the country.

Objectives

- To gain insight about what pregnant women know about toxoplasmosis and how to prevent transmission.
- To determine effective educational methods to educate pregnant women

Methods

- A total of 37 pregnant women were surveyed in two hospitals, Hospital del Sur and Hospital San Juan de Dios in the capital of the department of Quindio, Colombia.
- The women were given a pre-survey with no aids and given as much time as they needed.
- They were then given the educational pamphlet and were allowed as much time as needed.
- Then they were given the post-survey which is the same exact survey as the pre-survey.

- The surveys were graded on a scale of 0-3
  - 0: no answer
  - 1: response but no understanding
  - 2: little understanding or too general
  - 3: good understanding for each question

- each person's total was added resulting in a cumulative score to the following classifications:
  - 0: no answer
  - 1-6: low comprehension
  - 7-12: medium comprehension
  - 13-18: high comprehension

Methods(continued)

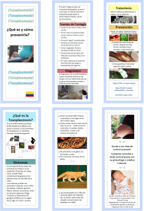

Figure 1. Pamphlet used in survey

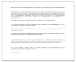

Figure 2. Sample Surveys

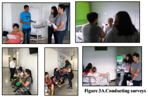

Figure 3A.Conducting surveys

Results

Figure 3B. Pre (top panel) and post (bottom panel)t-test score distribution in pie diagram.

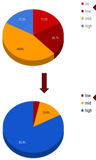

- *In the pre-survey, 17.24% had no previous knowledge on toxoplasmosis.*
  - *82.7% of women surveyed had no, low, and mid comprehension combined*

- *89.66% of the women surveyed improved by an average of 8.2 pts which is more than 100% improvement over their pre survey score.*
- *6.90% of women remained the same. Only 3.45% of women score lowered.*

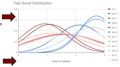

Figure 3C. Pre and post-test score distribution.

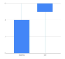

Figure 4: Box plot showing pre and post survey scores using 2016 scoring method

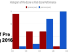

Figure 5: Histogram of Pre and post scores using 2016 scoring method

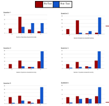

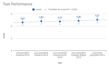

Figure 6A: Histogram of number of people and their score on each question. B. Test performance in differing settings in Panama and Colombia

Figure 7: summary table of all survey performances and survey performances plotted

Post-Test	Score (of 6 possible)
old pamphlet Panama 2016	4.63
new pamphlet Panama 2016	4.83
new pamphlet Panama print 2016	4.77
new pamphlet Panama digital 2016	4.88
Colombian pamphlet 2019	5.07

Conclusions

- Pamphlet clearly demonstrates it is effective in teaching pregnant women quickly and efficiently
- Possible route through which high seroprevalence rates are tackled
- Once this educational program is proven to be an effective instrument to deliver information in a new country or region as it was here, we can then apply this educational program to other countries in Latin America.
- Furthermore, using other data of risk factors unique to each region we can modify the educational program to focus on changing certain behaviors a population may have that makes the population much more likely toacure this infection and toxoplasmosis. We would be able to mold the program to each unique environment of each region and with *Toxoplasma* infection prevalence data to bring sophisticated effective educational techniques that target the unique key factors of the region to lower seroprevalence rates in the area with a rapid rate of efficiency given the results of these educational surveys.

Acknowledgements

- Rima McLeod, M.D., Dept. of Ophthalmology and Center for Toxoplasmosis, University of Chicago Hospitals
- Jorge Enrique Gomez Marin M.D. PhD, MSc
- Richard James Orozco Gonzalez MD
- Hospital del Sur and Hospital San Juan de Dios
- Catalina Raggi
- University of Quindio
- Funding: CGH

University of Chicago Center for Global Health

Summer Research Fellowship Report

August 23rd, 2019

Education as a Critical Tool to Aid in the Fight Against Toxoplasmosis

Jose Sanchez, Catalina Raggi, Laura Lorena Lopez, Richard James Orozco Gonzalez MD, Jorge Gomez Marin MD, Rima McLeod MD

University of Chicago

Abstract

Background

The objective of this study was to assess the level of understanding pregnant women have about *Toxoplasma gondii* and toxoplasmosis in Quindio Colombia. Rules and legislations address the management of the disease; however, the rate of seropositivity still remains high in this region of Colombia, signaling a lack of efficacy in delivery of information about toxoplasmosis to the general population. Therefore, we surveyed a group of women and were able to quantify what these women know about the disease compared to what these women should know.

Methods

We surveyed a total of 37 pregnant women of all ages hailing primarily of poor socioeconomic backgrounds in the waiting area of the maternity ward of Hospital San Juan and Hospital del Sur in the capital of Quindio, Armenia. They were given a survey with 6 questions relating to toxoplasmosis and allowed time, without time limit, to answer the survey. Once they indicated they were ready to proceed, an educational pamphlet was provided. This pamphlet discussed *T. gondii* infection, how it is acquired, how to prevent it, and the disease it causes, toxoplasmosis. The women were allowed to read the pamphlet for as long as they wished. When the women indicated they were ready to proceed, the same set of six questions was provided once more to identify whether the pamphlet led to learning key information along with short term knowledge retention.

Results

Analyzing the women's performance on both surveys we learned that the level of understanding was low initially. The mean correct response and range of responses on the six questions was 7.97 out of 18 possible. After reading the pamphlet, scores improved significantly with a mean score of 15.24 and a range of 6-18 overall. Using a chi- squared analysis we obtained p values of $p<0.0001$ between responses to the six questions before and after reading the pamphlet.

Conclusion

We determined that the use of the educational pamphlet improved knowledge in a statistically significant manner for each of the six questions, increasing knowledge about the infection, how it is acquired, and how to prevent it and the disease of toxoplasmosis.

Source of Funding: CUGH

Introduction

Toxoplasmosis is a disease caused by the protozoan parasite *Toxoplasma gondii*, *T. gondii* is one of the most prevalent parasites in the world (McLeod 2017). Because of its prevalence, there are many sources of transmission to humans with cats being the definitive hosts for the parasite. Acutely infected cats shed up to 5 million oocysts in two weeks (Dubey and Beattie, 1998; Aguirre *et al*., 2019, Triviño-Valencia *et al*., 2016). Millions of oocysts are deposited in nature through feline feces and can survive for years in warm moist soil or water, giving enough time for their wide spread, contaminating water, soil and food. Even one sporulated oocyst is infectious Sources of infection include ingestion of contaminated water, shellfish, and improperly washed produce, and bradyzoites in cysts can contaminate and be transmitted via undercooked meats (Massie et al., 2010, Staggs et al., 2015). Because of the parasite's ability to survive in temperate climates, infection occurs easily in Colombia (Rudzinski, Meyer, Khoury, & Couto, 2013). Infection is generally asymptomatic and may go unrecognized or its symptoms may not be attributed to toxoplasmosis. This becomes dangerous when a previously seronegative woman acquires the infection for the first-time during gestation and vertical transmission of the parasite to the fetus occurs. Clinical manifestations of congenital toxoplasmosis vary depending on the time during gestation when the infection is acquired and are usually more severe if transmitted during early gestation. In these cases, severe damage to the retina and central nervous system may occur, including blindness, encephalitis, and microcephaly. Moreover, even if a child is initially asymptomatic, almost all untreated congenital infections are likely to result in chorioretinal lesions and visual impairment by adulthood. (McLeod 2017).

Over the last decade, several important advances have taken place in the fight against toxoplasmosis in Colombia. To combat the high seroprevalence, the nation developed a protocol

delineating the course of action a physician should take for cases of toxoplasmosis (Cortes 2012). Toxoplasmosis is now among the “mandatory to report diseases” in monthly prenatal screenings along with other potential diseases such as HIV. The establishment of an educational program against toxoplasmosis in Colombia is an important step in the fight against congenital toxoplasmosis and could lower toxoplasma seroprevalence. Although Colombia’s current legislation aims to diminish seroprevalence in the country, seroprevalence still remains well over 50% in some parts of Colombia (Gomez-Marin 2017). There have also been several cases where *T. gondii* outbreaks have occurred in both water and soil in the United States and Brazil among other countries. In the American dust epidemic in Atlanta, Georgia, 95% of the 37 infected people reported symptoms including headache, myalgias and fever, but only 7% were correctly diagnosed by their primary physician (Teutsch *et al.*, 1979; Dubey *et al.*, 1981). Symptoms of headache and myalgia in patients with lymphadenopathy were emphasized in the USA (Wong *et al.*, 2012) and Brazilian cases (reviewed in Dubey *et al.*, 2012). In the Amazon region, acute infection associated with interstitial pneumonia and death have been reported in epidemics in the and along the Maroni River in Guyana (Carme *et al.*, 2002, 2009; Demar *et al.*, 2007, 2012). A waterborne epidemic occurred in Panama between January 27 and February 18, 1979, 600 soldiers of the 1st Battalion of the 508th Infantry attended a three-week training course in the jungle in Panama (Benneson et al., 1982). During the two weeks after their return to Fort Bragg, North Carolina, 39 of the 98 soldiers in one company (the Combat Support Company) came down with a febrile illness caused by acute Toxoplasmosis due to the men drinking water contaminated with t. gondii oocysts in the jungles of Panama (Benneson et al., 1982). Likewise, in March 1995 in Victoria, Canada; 100 individuals met the criteria to be diagnosed with acute toxoplasmosis. The cause of this outbreak was traced back to a water reservoir that was affected

by increased rainfall and turbidity (Bowie et al., 1997) demonstrating even treated drinking water can still pose a risk factor. The numerous epidemics demonstrate that the responsibility to know about the parasite and the disease should not only fall on the physician as the parasite is widespread and difficult to eradicate. The general public lacks the necessary knowledge about the parasite *T. gondii* but should be aware of the risk the disease poses in order to aid in the prevention of transmission of the disease from expected sources. Thus, it is crucial that the general public is properly educated about the dangers of this disease, especially pregnant women, to prevent congenital toxoplasmosis.

Educational pamphlets may be part of effective educational instruments to deliver this to pregnant women. The use of these pamphlets would inherently diminish the burden on social workers and doctors which are already tasked with providing pregnant women with a plethora of important information relating to their pregnancy.

To determine whether the pamphlet could play a role in their ability to acquire knowledge, we surveyed pregnant women before and after the administration of the educational pamphlet on toxoplasmosis. Thereby we were able to gain insight into what is common knowledge among the women and whether they are capable of quickly learning crucial information relating to toxoplasmosis from this educational pamphlet. Additionally, we determined effective methods to educate pregnant women about toxoplasmosis, its effects on the child, and how to prevent transmission.

The educational pamphlet used in this study was created by the University of Chicago Center for Toxoplasmosis and was the result of several years of research in three phases. In the first, the pamphlet was compared with two other existing toxoplasmosis pamphlets from Brazil and the Toxoplasma Center made pamphlet for the March of Dimes (Li, 2015). The original

Brazilian pamphlet performed the best. Our pamphlet was revamped, improved, and then put to the test against the old Brazilian pamphlet in the second phase. The new University of Chicago pamphlet resulted in higher comprehension; however, the cost of printing was excessive in Panama ($3 per copy) and not cost effective (Heichman, 2016). Thus, in the third and final phase, the physical pamphlet was compared to a digital iPhone pdf. In this final phase, `Pregnant women (N=214) were surveyed over eight weeks in Hospital Santo Tomas in Panama City where women were administered a pretest composed of six questions. After completion of the pretest, women were randomly given informational material either in the form of a brochure or PDF on an iPhone. Once the women finished reviewing information, a post-test with the same six questions was administered. The brochure group (N=105) had a net increase of 4.49±1.39 and the PDF group (N=109) had a net increase of 3.62±1.79 in their test scores. Based on a group by time interaction in a mixed-effects regression model, the change over time was significantly different between the two groups ($p<0.001$) with the brochure group having a significantly greater increase than the pdf group. However, both groups showed a significant increase from pre- to post-test ($p<0.001$ for both). Also of note is that the two groups differed significantly in their pretest scores (brochure: 0.39±0.66, PDF group: 1.15±1.45, $p<0.001$) in Panama" (Moreira, Pandey, 2016).

Thus, we had established the effectiveness of this pamphlet in providing knowledge which was ready to be implemented. Yet, due to data that demonstrated *T. gondii* oocysts in the water systems of Quindio we modified the pamphlet (Triviño-Valencia et al., 2016). The modification to the pamphlet was simple and indicated that in Colombia, all water used for consumption should be clean water; either boiled or bottled to prevent ingestion of *T. gondii* oocysts which are present in the local system's water.

Methods

In late June of 2019 an educational survey was taken to determine what a subset of the pregnant population of Armenia, Colombia knew about t. gondii and toxoplasmosis using the pamphlet developed by the University of Chicago Toxoplasmosis Center for its Panamanian Toxoplasmosis Center. The pamphlet was adapted for Colombia where exposure to water which is not boiled or bottled is known to be a risk factor. We asked 6 questions about toxoplasmosis to pregnant women in waiting rooms of Hospital del Sur y Hospital San Juan de Dios.

The questions were:

1. What is *Toxoplasma gondii* and what is toxoplasmosis?

2. What are the symptoms of toxoplasmosis?

3. What are the sources of toxoplasmosis?

4. How do we diagnose Toxoplasma infection and
toxoplasmosis?

5. Original: What are the treatments for toxoplasmosis?

Modified: What do you do in case you do not follow the preventative measures. In case you drink unboiled water, poorly cooked meat, or handled soil without the proper safety measures?

6. How do you prevent toxoplasmosis?

Translation used in Colombia was as follows:

1. ¿Sabe usted qué es Toxoplasma gondii y que es el toxoplasmosis?

2. ¿Conoce cuales son los síntomas que produce la toxoplasmosis? Si su respuesta es positiva, indique cuales.

3. Nombre las fuentes de infección, en la cual se puede adquirir el parásito Toxoplasma gondii:

4. ¿Sabe usted cómo se diagnostica una infección por Toxoplasma gondii? Si su respuesta es positiva, explique por favor cómo.

5. ¿Que debo hacer en caso de que no cumpla las condiciones de prevención, es decir, en caso de que haya consumido agua sin hervir, alimentos mal cocidos o haya manipulado tierra o arena sin las condiciones de higiene adecuadas?

6. Y por último, ¿sabe cómo se puede prevenir la toxoplasmosis? Si su respuesta es positiva explique cómo.

First, the women were asked if they would like to be surveyed. If they agreed, as only approximately 1/3 of the patient population did, they were given a survey and allowed to take as much time as they wanted to fill it out. Once done, they were given the educational pamphlet on toxoplasmosis and allowed to read over the pamphlet for as long as they needed. When ready, they were then given the same survey they had completed to see how much the women had learned in a relatively short amount of time.

If they stated that they did not want to participate they were given the educational pamphlet without a survey.

Statistics: All statistics were calculated using Google Sheets. Chi Square regression was used to calculate the significance of the pamphlet with comprehension.

Results

A total of 37 pregnant women of ages 16-55 participated in the study hailing primarily of poor socioeconomic backgrounds. Of the 37 women, 8 could not complete the post-test due to external factors relating to their visit to the obstetrician.

A scale of 0-3 was used to determine the level of comprehension for each question. 0 = no answer, 1= response but no understanding, 2= little understanding or too general, 3 = good understanding for each question. Then each person's total was added resulting in a cumulative score to the following classifications:

0-6 (low) 7-12 (medium) 13-18 (high) comprehension

In the pre-survey, 17.24% had no previous knowledge about the disease. Questions number three and four had the highest number of people with the highest comprehension (3) with ten and eleven people respectively (Figure D). Question three asked the women to name sources of toxoplasmosis, with a large majority of the population stating water and cats as sources of the disease. Question four asked the women "how does one diagnose toxoplasmosis." 37.93% of the women responded with an answer relating to blood tests (Fig. D). However, the questions that received the lowest amount of high comprehension provide more insight. 89.66% of people could not name the symptoms for toxoplasmosis and 79.31% had no previous knowledge about toxoplasmosis as a disease nor where it came from (Fig. D). Many pregnant women wrote that the disease was contracted through the fur of cats suggesting that the women's perception of the disease to be incorrect or lacking complete understanding. When the women were surveyed once again, their answers were improved dramatically. The average score doubled demonstrating the efficacy of educational pamphlets.

Chi-Squared analysis for the difference in scores for each question between pre and post-survey was found to be statistically important for all questions as well as a t-test value of <0.001 (Table 1). 89.66% of the women surveyed improved by an average of 8.2 pts which is more than 100% improvement over their pre-survey score. The minimum improvement was only 2 points however maximum improvement was of 17 points. 6.90% of women remained the same and only 3.45% of women worsened.

Survey Results Compared with Previous Studies in Panama

As previously mentioned, the pamphlet used in this study was based on a pamphlet developed for Panama over the course of several years. An important distinction between this study and the previous is the grading scale used. In Panama, the surveys were graded pass/fail for each question, and the total number of correct questions determined their score. Applying this same grading scale, the average pre-score in Colombia is 2 and the average post-score is 5.07 ($p<0.001$) resulting in higher scores for the pregnant women in Colombia compared to those in Panama of 2016 (Fig. H). The average score of 5.07 achieved in Colombia is higher than those achieved in the study of 2016 comparing the old University of Chicago Center for Toxoplasmosis pamphlet and the new pamphlet. With average post-score of the old and new pamphlet being 4.63 and 4.83 respectively (Heichman 2016) (Fig. H). The score achieved in Colombia is also higher than the digital vs paper study of 2016 where scores of 4.77 and 4.88 respectively (Moreira, Pandey, 2016) (Fig. H). The individual answers were not able to be recovered for the 2016 studies and as a result, an in-depth analysis of toxoplasmosis knowledge between the two locations is yet to be finalized but these preliminary results show the efficacy of the pamphlet is improving.

Discussion

The purpose of surveying this group of women was to determine what preexisting knowledge the women had of t *T. gondii* infections and toxoplasmosis in Quindio, Colombia, to understand the gap in communication between the government and health professionals and the common person. The results of the survey show that in this population there is a general lack of knowledge among all women about the correct process for acquiring the parasitic disease. Although the women washed their produce before consumption and washed their hands, a large portion of the women used tap water. The problem with the use of tap water in Colombia is that it does not lower the women's risk to of contracting the parasite as the Quindio water is known to contain *T. gondii* DNA (Fabiana et al. 2002) and tap water is now a risk factor. Nevertheless, the modified pamphlet stressed the importance of using clean water, that is water that has been boiled or bottled water and the women's post-tests answers included distinctions between tap water, boiled, and bottled water in their answer signaling an important understanding of the risk factor tap water poses to the women. This knowledge is important for the pamphlet to focus on addressing these key areas for they are most likely the most overlooked in the Colombian anti-toxoplasmosis campaign. These important modifications may be the key difference in combating toxoplasmosis in Colombia.

The pamphlet was demonstrated to be extremely efficient in providing pregnant women with important information about toxoplasmosis (Li, 2015). Having this type of educational pamphlet is crucial in the fight to combat toxoplasmosis given Colombia's perfect combination of conditions for *T. gondii* to flourish given the humidity, rainfall, and proximity to bodies of water (Bates 2012). The efficacy of the pamphlet could be improved by simplifying the syntax. It was found to be higher than necessary for the level of education for women in Armenia, Quindío

by local experts. Implementation of the pamphlet in each hospital or clinic was reviewed by the chief Obstetrician before their use and a common suggestion was to simplify the delivery of the information further. The simplification of the pamphlet could be improved to contain only crucial information for pregnant women about what they should do to lessen risk factors while pregnant as the women surveyed did not attain a satisfactory level of knowledge in the pre-survey and had misconceptions about the sources of the disease. A common misconception was the disease was transmitted through the fur of cats. While cats are the definitive source for oocysts, the oocysts are found primarily in feces, not fur, of cats. Especially in the feces of younger cats. This is problematic for the women do not truly understand the mechanism of the disease and are susceptible for they may be taking inadequate precautions against the disease. Further analysis would have to be performed to determine if these women followed the necessary precautions for the disease. Although seeming counterintuitive, pregnant women are already under tremendous stress due to all the appointments and blood tests they must be aware of, as well as receiving an excess of information concerning numerous congenital diseases they should also be wary of. The high seroprevalence demonstrates that this wealth of information is imperfect, for the target population remains heavily affected. Therefore, further tailoring of the pamphlet must be helpful before implementation in a new setting. Approval of the pamphlet by a local doctor who has experience working with pregnant women in the region might improve the long-term retention of the information as well as lessening the burden of important things to keep in mind for pregnant women which may lead to a better pregnancy.

When graded by the same method as in Panama, the pre and post survey scores were higher than those in Panama in 2016. However, this could be attributed to different graders being used to grade the exams; yet, the statistical significance still demonstrates the crucial role of the

pamphlet in decreasing knowledge gap and increasing knowledge of *T. gondii.* The lack of information at the onset and significant improvement in knowledge demonstrates the need for this educational pamphlet in both countries.

Conclusion and Further Development

Once this educational program is proven to be an effective instrument to deliver information via, we can then apply this educational program to other countries in Latin America. Furthermore, using other data of risk factors unique to each region we can modify the educational program to focus on changing certain behaviors a population may have that makes the population much more susceptible to toxoplasmosis. We would be able to mold the program to each unique environment of each region and with toxoplasmosis prevalence data to bring sophisticated effective educational techniques that target the unique key factors of the region to lower seroprevalence rates in the area with a rapid rate of efficiency given the results of these educational surveys.

References

Aguirre AA, Longcore T, Barbieri M, Dabritz H, Hill D, Klein PN, Lepczyk C, Lilly EL, McLeod R, Milcarsky J, Murphy CE. 2019. The One Health approach to toxoplasmosis: epidemiology, control, and prevention strategies. EcoHealth. Apr 3:1-3. https://doi.org/10.1007/s10393-019-01405-7.

Bates, S. T., Clemente, J. C., Flores, G. E., Walters, W. A., Parfrey, L. W., Knight, R., & Fierer, N. (2012, December 13). Global biogeography of highly diverse protistan communities in soil. *The ISME Journal* **volume 7**, pages652–659 (2013) https://www.nature.com/articles/ismej2012147

Beneson, M. W., Takafuji, E. T., Lemon, S. M., & Greenup, R. L. (1982, September 9). Oocyst-Transmitted Toxoplasmosis Associated with Ingestion of Contaminated Water. *The New England Journal of Medicine*, (307), 666–669. doi: 10.1056/NEJM198209093071107

Bowie, W. R., King, A. S., Werker, D. H., Issac-Renton, J. L., Bell, A., Eng, S. B., & Marion, S. A. (1997, July 19). Outbreak of toxoplasmosis associated with municipal drinking water. *The Lancet*, *350*(9072), 173–177. doi: https://doi.org/10.1016/S0140-6736(96)11105-3

Cañón-Franco1, W. A., López-Orozco1, N., Enrique, J., & Dubey4, J. P. (2014, September 4). *An overview of seventy years of research (1944 – 2014) on toxoplasmosis in Colombia, South America*. *Parasites & Vectors* **volume 7**, : 427 (2014) https://parasitesandvectors.biomedcentral.com/articles/10.1186/1756-3305-7-427

Carme, B., Bissuel, F., Ajzenberg, D., Bouyne, R., Aznar, C., Demar, M., Bichat, S., Louvel, D., Bourbigot, A.M., Peneau, C., Neron, P., Dardé, M.L., 2002. Severe acquired toxoplasmosis in immunocompetent adult patients in French Guiana. J. Clin. Microbiol. 2002 (40), 4037–4044.

Carme, B., Demar, M., Ajzenberg, D., Dardé, M.L., 2009. Severe acquired toxoplasmosis caused by wild cycle of *Toxoplasma gondii*, French Guiana. Emerg. Infect. Dis. 15, 656–658.

Cortés, A., Jorge, Gómez, E., Jorge, Silva, I., Pedro, … Pio. (2012, January 1). *Guía de atención integral para la prevención, detección temprana y tratamiento de las complicaciones del embarazo, parto y puerperio: sección toxoplasmosis en el embarazo.* Retrieved from https://www.elsevier.es/es-revista-infectio-351-articulo-guia-atencion-integral-prevencion-deteccion-S0123939212700188

Demar, M., Ajzenberg, D., Maubon, D., Djossou, F., Panchoe, D., Punwasi, W., Valery, N., Peneau, C., Daigre, J.L., Aznar, C., Cottrelle, B., Terzan, L., Dardé, M.L., Carme, B., 2007. Fatal outbreak of human toxoplasmosis along the Maroni River: epidemiological, clinical, and parasitological aspects. Clin. Infect. Dis. 45, e88–e95.

Demar, M., Hommel, D., Djossou, F., Peneau, C., Boukhari, R., Louvel, D., Bourbigot, A.M., Nasser, V., Ajzenberg, D., Darde, M.L., Carme, B., 2012. Acute toxoplasmoses in immunocompetent patients hospitalized in an intensive care unit in French Guiana. Clin. Microbiol. Infect. 18, E221–E231.

Dubey, J.P., Beattie, C.P., 1998. Toxoplasmosis of animals and man. CRC Press, Boca Raton, FL.

Dubey, J.P., Lago, E.G., Gennari, S.M., Su, C., Jones, J.L., 2012. Toxoplasmosis in humans and animals in Brazil: high prevalence, high burden of disease, and epidemiology. Parasitology 139 (11), 1375–1424.

Dubey, J.P., Sharma, S.P., Juranek, D.D., Sulzer, A.J., Teutsch, S.M., 1981. Characterization *of Toxoplasma gondii* isolates from an outbreak of toxoplasmosis in Atlanta, Georgia. Am. J. Vet. Res. 42 (6), 1007–1010.

Fabiana, Pérez, Enrique, J., Arias, Eduardo, S., Gómez, … Lora. (2002.). Toxoplasma gondii infection of meat for human consumption detected by PCR assay in three cities from the coffee region of Colombia. Retrieved from http://www.scielo.org.co/scielo.php?pid=S0123-93922007000300004&script=sci_arttext&tlng=pt

Heichman, Sharon (2015) *Educational Pamphlet Study in this series of papers Part II*

Kean, B. H., Kimball, A. C., & Chistenson, W. N. (1969, May 12). An Epidemic of Acute Toxoplasmosis. *JAMA*, *208*(6), 1002–1004. doi: 10.1001/jama.1969.03160060072008

Li Xuan (2014). *Why Diagnose, Prevent, and Treat Toxoplasmosis? A Study and Program to Benefit Maternal and Child Health in Panama. in this series of papers Part II*

Marín, G.omez Jorge, E. (n.d.). *Toxoplasmosis: Un problema de Salud Pública en Colombia*. Retrieved from http://www.scielo.org.co/scielo.php?script=sci_arttext&pid=S0124-00642002000400003

Massie, G. N., Ware, M. W., Villegas, E. N., & Black, M. W. (2010, May). Uptake and transmission of Toxoplasma gondii oocysts by migratory, filter-feeding fish. *Veterinary Parasitology*, *169*(3-4), 296–303. doi: https://doi.org/10.1016/j.vetpar.2010.01.002

McLeod, Rima. *"An Overview of Toxoplasmosis: Cause, Prevalence, and Consequences."* TRI: Information about Toxoplasmosis. Toxoplasmosis Research Institute and Center, n.d. Web. 15 Sept. 2016. Website at Toxopplasmosis.org

Moreira, Aliya F., Pandey, Abhi (2016) *Investigating Social and Infrastructural Parameters Concerning Toxoplasmosis in Panama. in this series of papers Part II*

Rudzinski, M., Meyer, A., Khoury, M., & Couto, C. (2013, November 14). Is reactivation of toxoplasmic retinochoroiditis associated to increased annual rainfall? Retrieved from Parasite. 2013; 20: 44. Published online 2013 Nov 14. doi: 10.1051/parasite/2013044

PMCID: PMC3826532 PMID: 24225023

https://www.ncbi.nlm.nih.gov/pmc/articles/PMC3826532/

Staggs, S. E., Keely, S. P., Ware, M. W, Schable, Nancy, See, Mary Jean, Gregorio, Dominic, & Zou, Xuan. (2015, December). The development and implementation of a method using blue mussels (Mytilus spp.) as biosentinels of Cryptosporidium spp. and Toxoplasma gondii contamination in marine aquatic environments. *Parasitology Research*, *114*(12), 4655–4667.

Teutsch, S.M., Juranek, D.D., Sulzer, A., Dubey, J.P., Sikes, R.K., 1979. Epidemic toxoplasmosis associated with infected cats. N. Engl. J. Med. 300, 695–699.

Triviño-Valencia, J., Lora, F., Zuluaga, J. D., & Gomez-Marin, J. E. (2016, January 16). Detection by PCR of pathogenic protozoa in raw and drinkable water samples in Colombia. *Parasitology Research* **volume 115**, pages1789–1797 (2016) https://link.springer.com/article/10.1007/s00436-016-4917-5

Weiss, L. M., & Kim, K. (Eds.). (2014). *Toxoplasma Gondii: The Model Apicomplexan: Perspectives and Methods* (2nd ed.). S.l.: ELSEVIER ACADEMIC PRESS.

Wong, W.K., Upton, A., Thomas, M.G., 2012. Neuropsychiatric symptoms are common in immunocompetent adult patients with *Toxoplasma gondii* acute lymphadenitis. Scand. J. Infect. Dis. Scand J Infect Dis 2013 May;45(5):357-61. doi: 10.3109/00365548.2012.737017.Epub 2012 Dec 4.

Figure Legends:

Figure A. Pamphlet used for educational survey.

Figure B. Pre-test comprehension.

Figure C. Post-test comprehension.

Figure D. Histogram of number of people and their respective score on each question.

Figure E. Distribution Curve of Women's Performance on each Question (pre and post).

Figure F. Box plot Scores of Pre and Post-test performance using Panamanian grading scale.

Figure G. Histogram of pre-test vs post-test performance.

Tables

Table 1.

**q1**	**pre**	**post**		**pre**	**post**
**# people 0**	5	0	mean	1.10	2.14
**# people 1**	18	7	median	1	2
**# people 2**	4	11	mode	1	2
**# people 3**	2	11	std	**0.78**	**0.79**

**q2**	**pre**	**post**		**pre**	**post**
**# people 0**	7	0	mean	0.93	2.72
**# people 1**	19	2	median	1	3
**# people 2**	1	4	mode	1	3
**# people 3**	2	23	std	**0.75**	**0.59**

**q3**	**pre**	**post**		**pre**	**post**
**# people 0**	6	0	mean	1.55	2.72
**# people 1**	11	3	median	1	3
**# people 2**	2	2	mode	1	3
**# people 3**	10	24	std	**1.18**	**0.65**

**q4**	**pre**	**post**		**pre**	**post**
**# people 0**	6	1	mean	1.66	2.13
**# people 1**	9	2	median	1	3
**# people 2**	3	2	mode	3	3
**# people 3**	11	24	std	**1.20**	**0.76**

**q5**	**pre**	**post**		**pre**	**post**
**# people 0**	9	1	mean	1.21	2.59
**# people 1**	11	4	median	1	3
**# people 2**	3	1	mode	1	3
**# people 3**	6	23	std	**1.11**	**0.87**

**q6**	**pre**	**post**		**pre**	**post**

**# people 0**	7	1	mean	1.52	2.38
**# people 1**	8	5	median	1	3
**# people 2**	6	5	mode	1	3
**# people 3**	8	18	std	**1.16**	**0.90**

Summary Table	
Question Number	Chi-squared value
1	< 0.001
2	< 0.001
3	< 0.001
4	< 0.001
5	< 0.001
6	< 0.001

1. Mean Pre and Post-Test # of questions correct for both Brochure and PDF groups.

2.

Post-Test	Score (of 6 possible)
old pamphlet Panama 2016	4.63
new pamphlet Panama 2016	4.83
new pamphlet Panama print 2016	4.77
new pamphlet Panama digital 2016	4.88
Colombian pamphlet 2019	5.07

2. Summary Table of Post-Test scores of all surveys done using the University of Chicago Center forToxoplasmosis pamphlet

A.

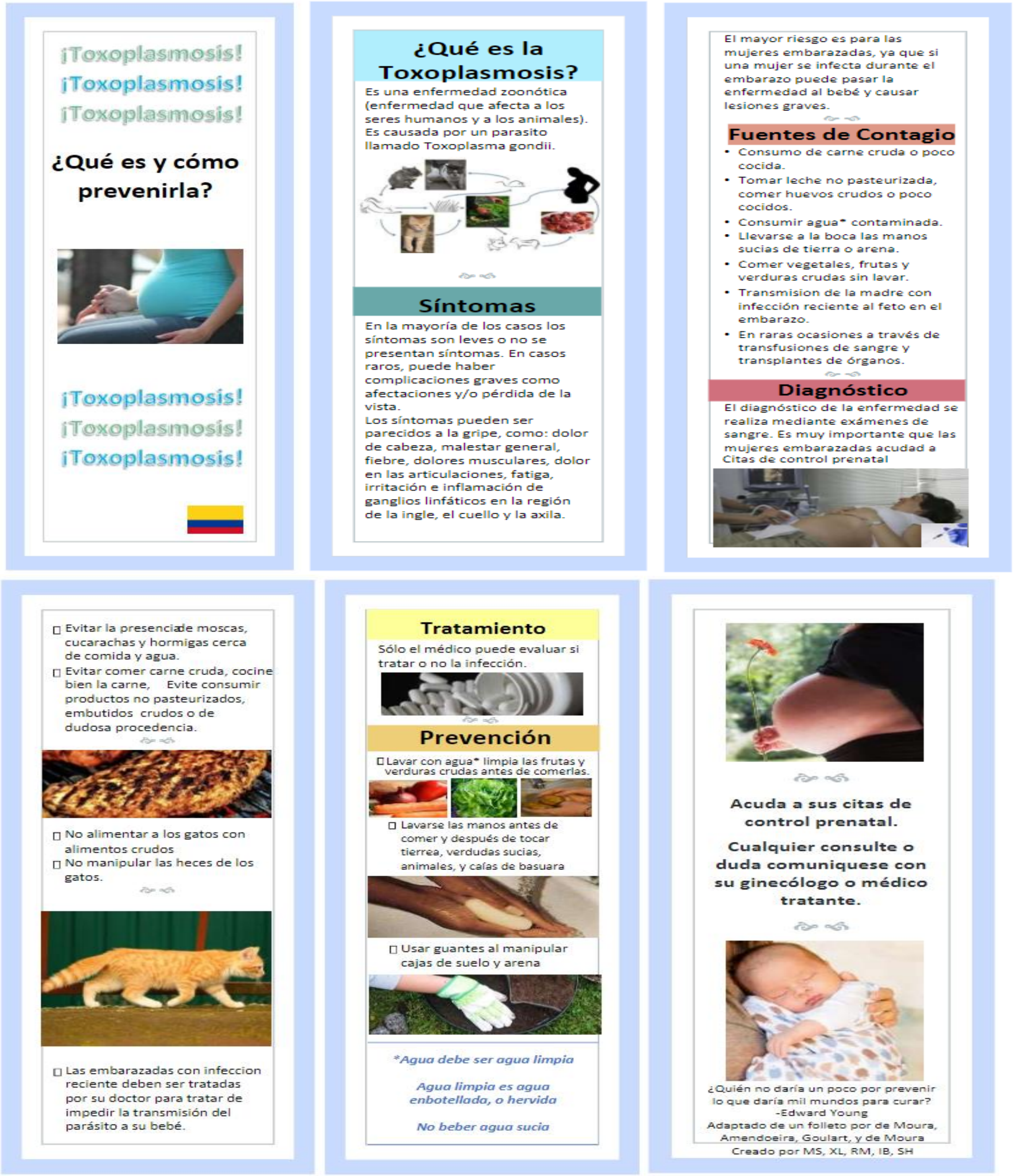

¡Toxoplasmosis!
¡Toxoplasmosis!
¡Toxoplasmosis!

¿Qué es y cómo prevenirla?

¡Toxoplasmosis!
¡Toxoplasmosis!
¡Toxoplasmosis!

¿Qué es la Toxoplasmosis?

Es una enfermedad zoonótica (enfermedad que afecta a los seres humanos y a los animales). Es causada por un parasito llamado Toxoplasma gondii.

Síntomas

En la mayoría de los casos los síntomas son leves o no se presentan síntomas. En casos raros, puede haber complicaciones graves como afectaciones y/o pérdida de la vista.
Los síntomas pueden ser parecidos a la gripe, como: dolor de cabeza, malestar general, fiebre, dolores musculares, dolor en las articulaciones, fatiga, irritación e inflamación de ganglios linfáticos en la región de la ingle, el cuello y la axila.

El mayor riesgo es para las mujeres embarazadas, ya que si una mujer se infecta durante el embarazo puede pasar la enfermedad al bebé y causar lesiones graves.

Fuentes de Contagio

- Consumo de carne cruda o poco cocida.
- Tomar leche no pasteurizada, comer huevos crudos o poco cocidos.
- Consumir agua* contaminada.
- Llevarse a la boca las manos sucias de tierra o arena.
- Comer vegetales, frutas y verduras crudas sin lavar.
- Transmision de la madre con infección reciente al feto en el embarazo.
- En raras ocasiones a través de transfusiones de sangre y transplantes de órganos.

Diagnóstico

El diagnóstico de la enfermedad se realiza mediante exámenes de sangre. Es muy importante que las mujeres embarazadas acudad a Citas de control prenatal

- Evitar la presenciade moscas, cucarachas y hormigas cerca de comida y agua.
- Evitar comer carne cruda, cocine bien la carne, Evite consumir productos no pasteurizados, embutidos crudos o de dudosa procedencia.
- No alimentar a los gatos con alimentos crudos
- No manipular las heces de los gatos.
- Las embarazadas con infeccion reciente deben ser tratadas por su doctor para tratar de impedir la transmisión del parásito a su bebé.

Tratamiento

Sólo el médico puede evaluar si tratar o no la infección.

Prevención

- Lavar con agua* limpia las frutas y verduras crudas antes de comerlas.
- Lavarse las manos antes de comer y después de tocar tierrea, verdudas sucias, animales, y cajas de basuara
- Usar guantes al manipular cajas de suelo y arena

**Agua debe ser agua limpia*

Agua limpia es agua enbotellada, o hervida

No beber agua sucia

Acuda a sus citas de control prenatal.

Cualquier consulte o duda comuniquese con su ginecólogo o médico tratante.

¿Quién no daría un poco por prevenir lo que daría mil mundos para curar?
-Edward Young
Adaptado de un folleto por de Moura, Amendoeira, Goulart, y de Moura
Creado por MS, XL, RM, IB, SH

104b

B.

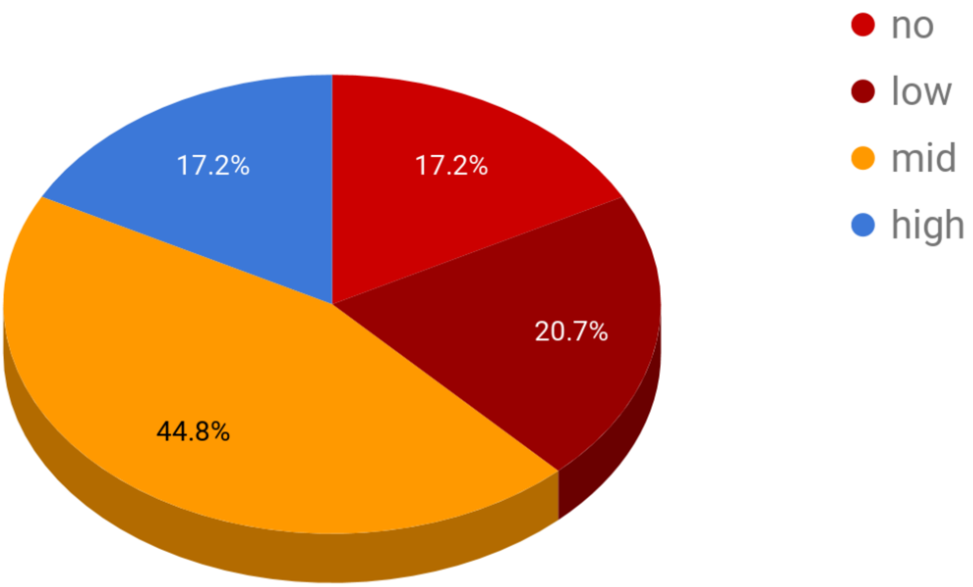

C.

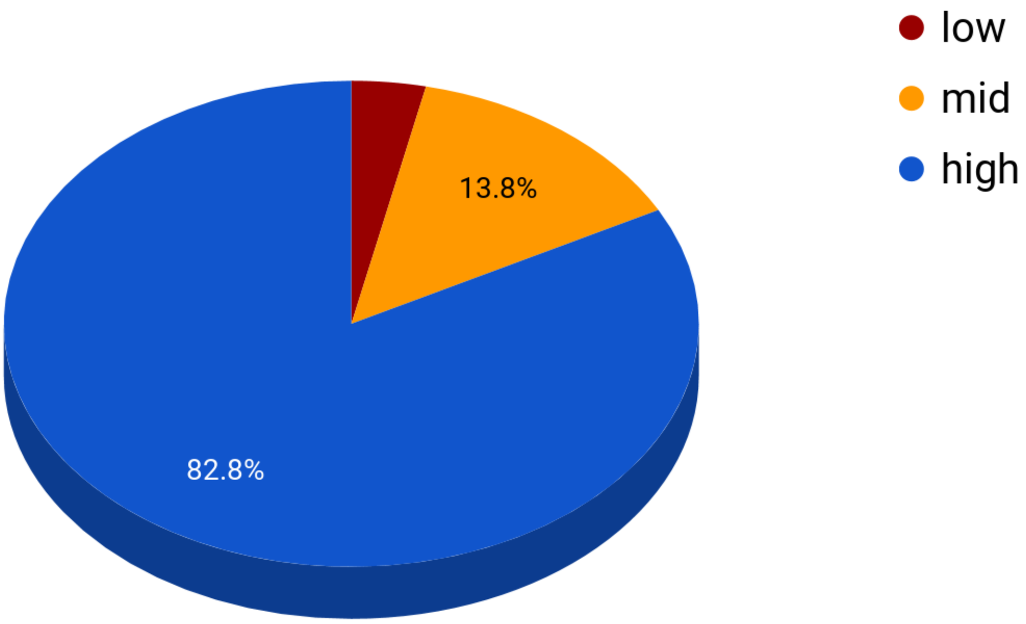

D.

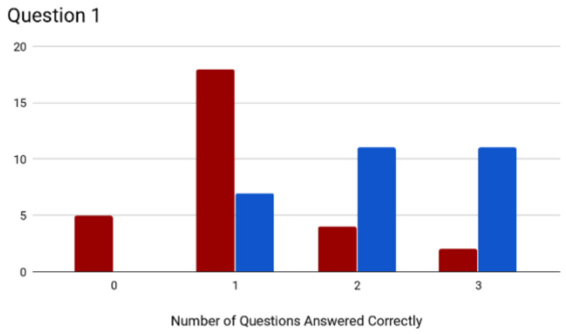

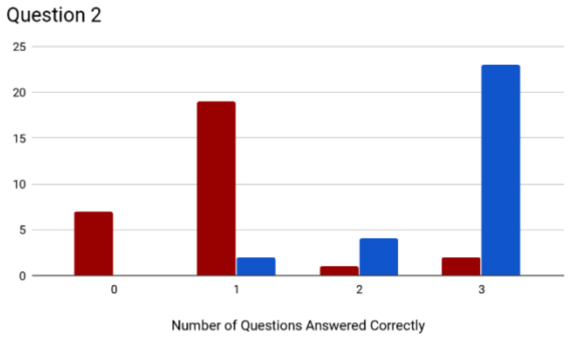

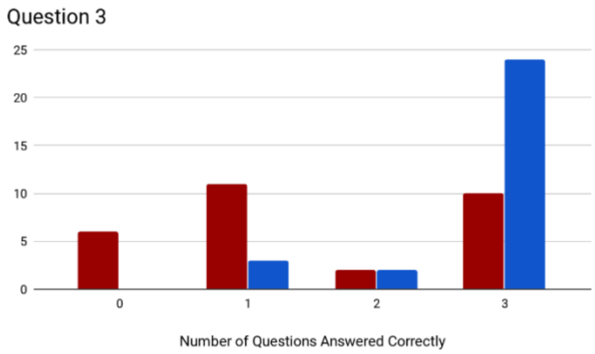

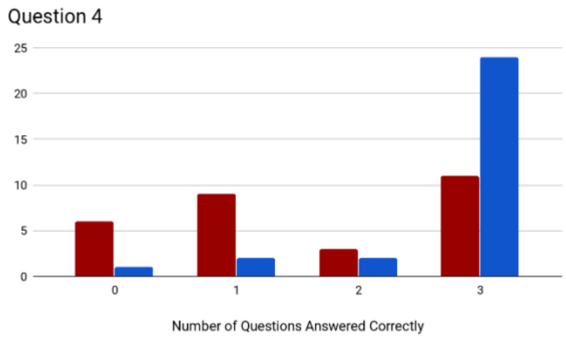

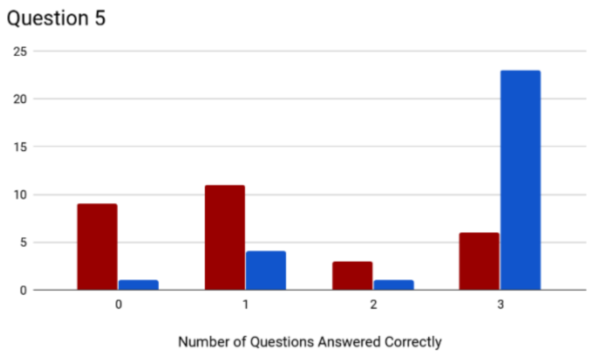

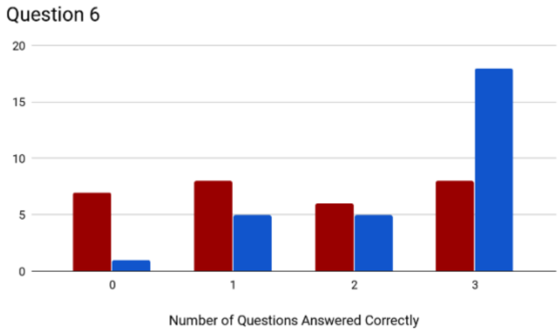

Pre-Test

Post- Test

E.

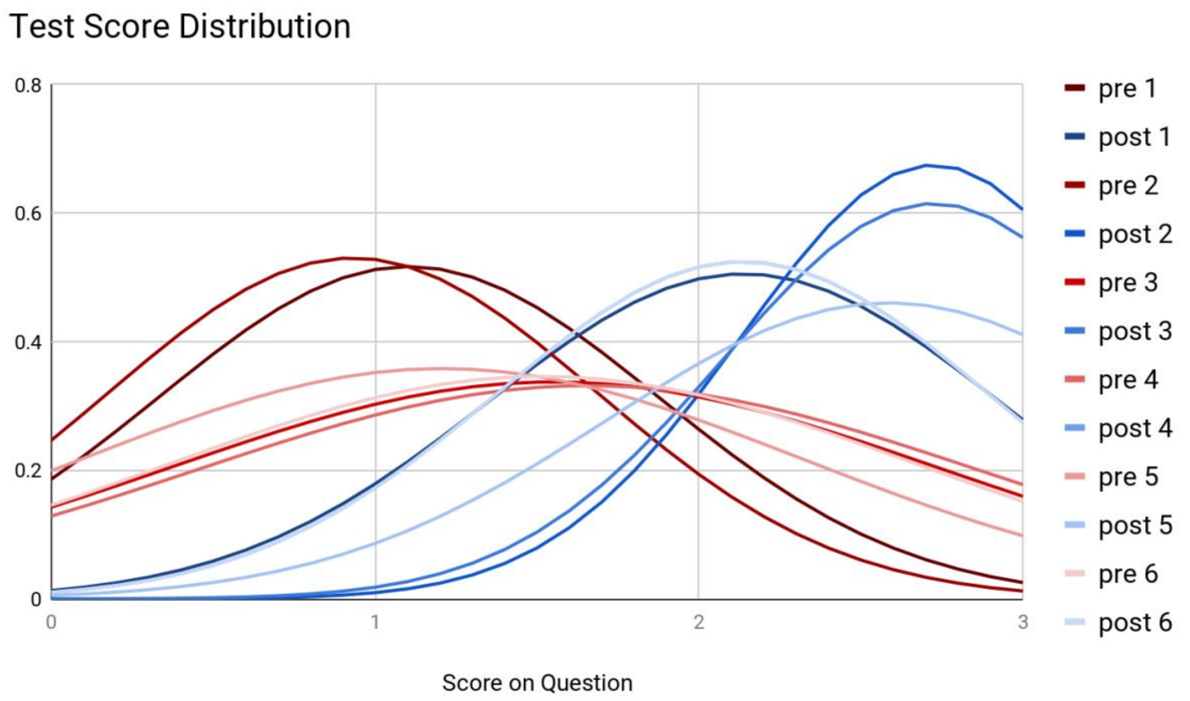
Test Score Distribution
0.8
0.6
0.4
0.2
0
0
1
2
3
Score on Question
pre 1
post 1
pre 2
post 2
pre 3
post 3
pre 4
post 4
pre 5
post 5
pre 6
post 6

F.

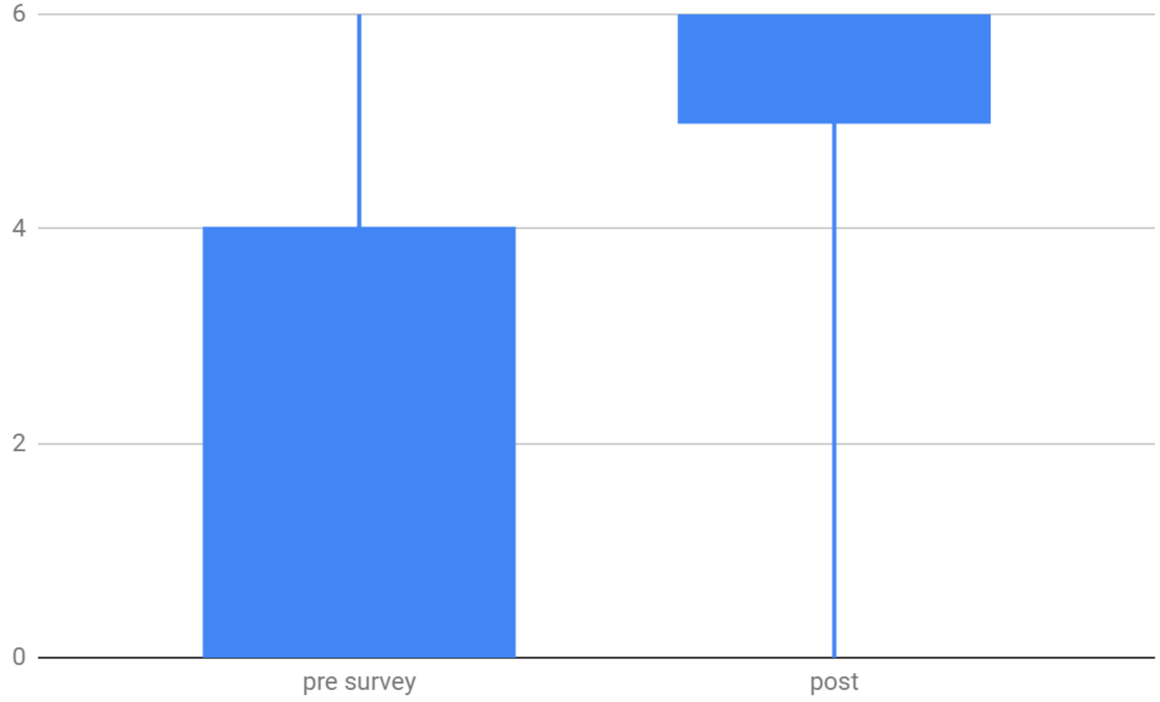

G.

H.

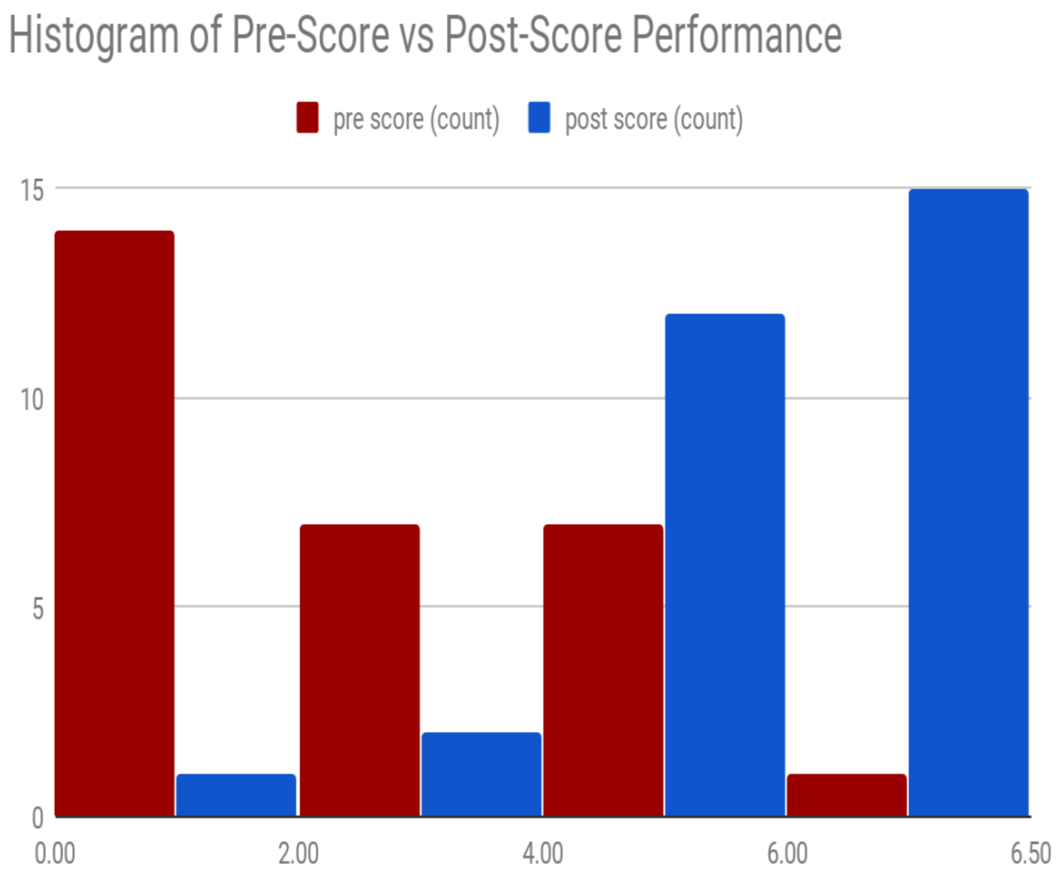
Histogram of Pre-Score vs Post-Score Performance
pre score (count)
post score (count)
15
10
5
0
0.00
2.00
4.00
6.00
6.50

H.

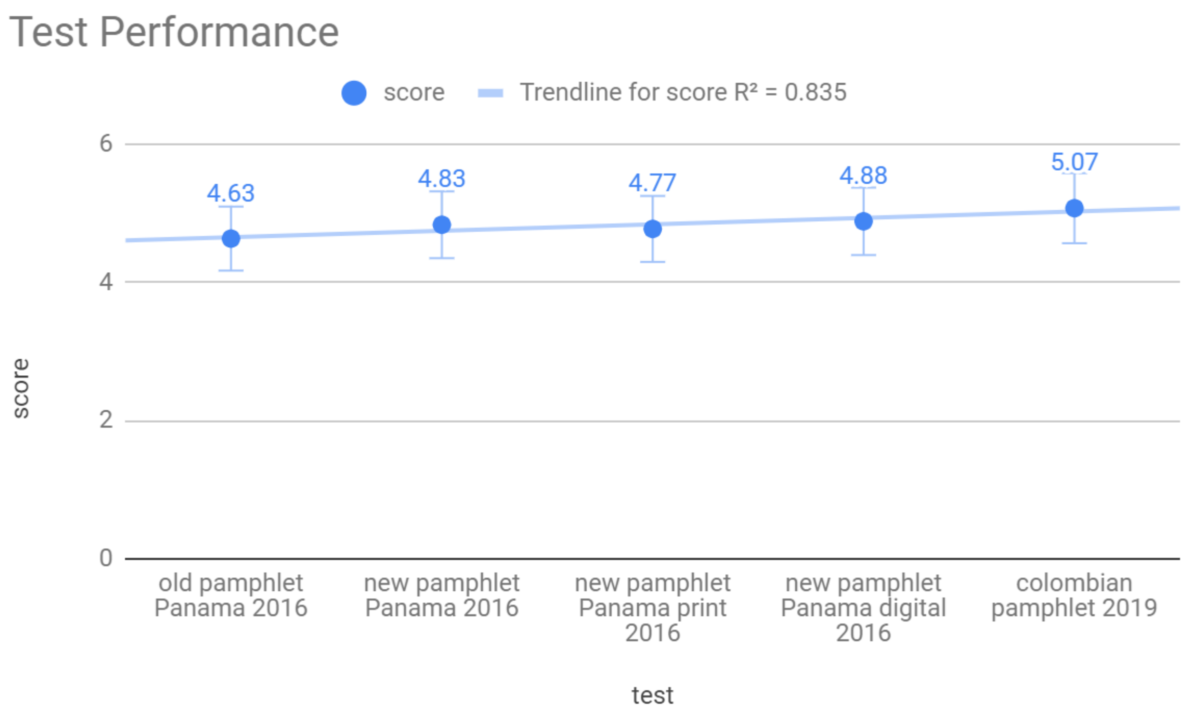
Test Performance
score
Trendline for score R² = 0.835
6
4
2
0
score
4.63
4.83
4.77
4.88
5.07
old pamphlet Panama 2016
new pamphlet Panama 2016
new pamphlet Panama print 2016
new pamphlet Panama digital 2016
colombian pamphlet 2019
test

THE UNIVERSITY OF CHICAGO | Center for Global Health

Education as a Critical Tool to Aid in the Fight against Toxoplasmosis

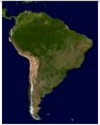

Presented by
Jose Sanchez*

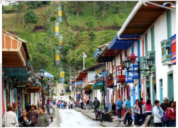

*In collaboration with Cat Reggaez, JJorge Enrique Gómez Marín, Laura Lorena López, Elizabeth Torres, Juan David Valencia, Manuela Mejia Oquendo,Daniel Celis, Mónica Vargas,, Mariangela Soberon Felin, Abhi Pandya, Aliya Moreira,, Stefany Velasco, Daniel Celis Giraldo, Catherine Castro, Margarita Ramirez, Davina Moosazadeh, Osvaldo Reyes, a, Guillermo Pradieu, Dora Estripeaut,, Sharon Heichman, Rima McLeod

Backoground

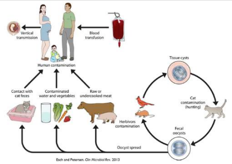

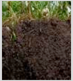

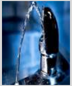

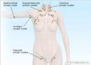

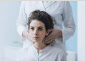

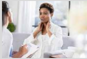

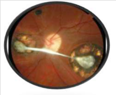

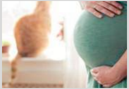

***Toxoplasma gondii* and Toxoplasmosis**

- World's most common parasite of humans;disease.
- Acutely infected **ca**ts excrete 500 million oocysts in 2 weeks, Even 1 is infectious,
- Persist in water & warm moist soil >1yr.
- Can coontaminate **environment** in large numbers
- **Livestock** eat oocysts,so in meat
- Also on fruits and vegetables
- Epidemics and endemic
- Hypervirulent parasites along Amazon and it tributaries

- **Primary initial infection**
- May be asymptomatic, or cause fever, myalgias
- May cause systemic illness
- May cause swolen lymph nodes
- Abnormalities of brain
- Eye Disease-most common
- More in Central/ South America

- **Congenital infection;Immne compromise**

For infants born to mothers with primary infection in gestation and for people with weakened immune systems, toxoplasmosis may cause loss of life, Cognition, motor functiion and sight.

THE UNIVERSITY OF CHICAGO | Center for Global Health

Background (cont)

Quindio Colombia

- A department of Colombia nested in the Andes mountain range in the heart of the country.
- The department is the center for Colombia's coffee production
- Also is one of Colombia's departments with the highest antibody seroprevalence indicating T. gondii infection in the country
- More than 50% of the population is seropositive

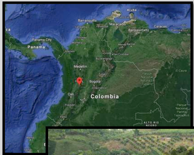

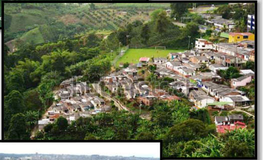

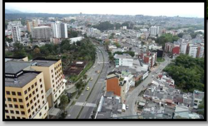

THE UNIVERSITY OF CHICAGO | Center for Global Health

Hypothesis: Education Contributes to Building an Effective Public Health System

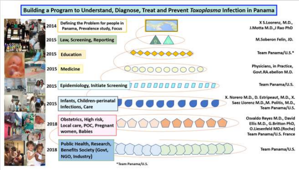

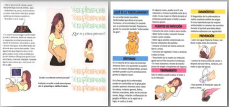

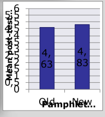

- We believe that providing an educational pamphlet could help reduce *T. gondii* seroprevalence in Colombia.
- The pamphlet we used was developed over several years to be useful for pregnant women in Panama.
- For the Panamanian Toxoplasmosis Center and major obstetrical Hospital Santo Tomas, it was proven to be effective in conveying relevant information.

THE UNIVERSITY OF CHICAGO | Center for Global Health

Purpose

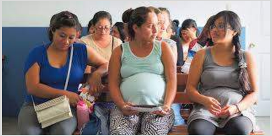

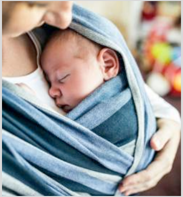

Toxoplasma in Colombia is addressed in legislation, however, toxoplasmosis seroprevalence remains high.

- We wanted to gain insight on what Pregnant women Knew about toxoplasmosis and how to prevent transmission.
- Determine effective educational methods to educate pregnant women to prevent vertical transmission and congenital toxoplasmosis.

THE UNIVERSITY OF CHICAGO | Center for Global Health

Methods

Top: Hospital San Juan de Dios Bottom: Hospital del Sur

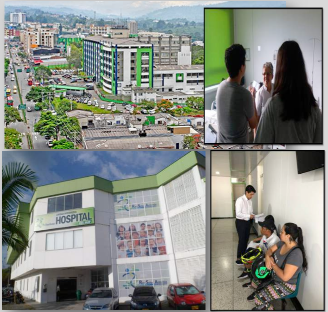

- 37 pregnant women were surveyed in two hospitals: Hospital del Sur and Hospital San Juan de Dios
- In the capital of Quindío Colombia.
- Women were given a survey with no aids and allowed as much time as needed.
- Then given educational pamphlet
- Allowed to read over pamphlet for as long needed.
- The same survey was administered once more.
- Analyzed what pregnant women know, did not know, and what information delivered was delivered via the pamphlet.

THE UNIVERSITY OF CHICAGO | Center for Global Health

Methods(cont)

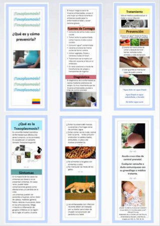

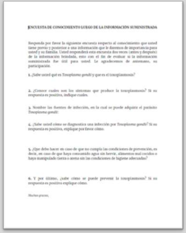

ENCUESTA DE CONOCIMIENTO LUEGO DE LA INFORMACIÓN SUMINISTRADA

Responda por favor la siguiente encuesta respecto al conocimiento que usted tiene previo y posterior a una información que le daremos de importancia para usted y su familia. Usted responderá esta encuesta dos veces (antes y después) de la información brindada, esto con el fin de evaluar si la información suministrada fue útil para usted. Le agradecemos de antemano, su participación.

1. ¿Sabe usted qué es *Toxoplasma gondii* y que es el toxoplasmosis?

2. ¿Conoce cuales son los síntomas que produce la toxoplasmosis? Si su respuesta es positiva, indique cuales.

3. Nombre las fuentes de infección, en la cual se puede adquirir el parásito *Toxoplasma gondii*:

4. ¿Sabe usted cómo se diagnostica una infección por *Toxoplasma gondii*? Si su respuesta es positiva, explique por favor cómo.

5. ¿Que debo hacer en caso de que no cumpla las condiciones de prevención, es decir, en caso de que haya consumido agua sin hervir, alimentos mal cocidos o haya manipulado tierra o arena sin las condiciones de higiene adecuadas?

6. Y por último, ¿sabe cómo se puede prevenir la toxoplasmosis? Si su respuesta es positiva explique cómo.

Muchas gracias,

- Pamphlet adapted to Colombia-esp.water
- Each survey question was graded on a scale of 0-3
- 0: no answer
- 1: response but no understanding
  - Response was off topic
- 2: little understanding or too general
  - Response contained no specific information relating to Toxoplasmosis, e.g.: getting a test instead of specifying type
- 3: good understanding for each question
  - Question contained key words like boiled water,

  Cat feces, etc.

each person's total was added resulting in a cumulative score to the following classifications:

- 0: no answer
- 1-6: low comprehension
- 7-12: medium comprehension
- 13-18: high comprehension

THE UNIVERSITY OF CHICAGO | Center for Global Health

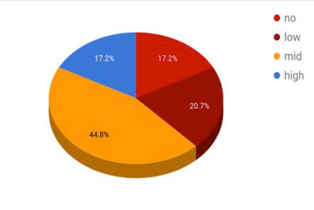

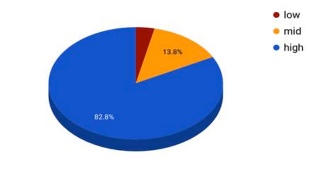

Results

- In the pre-survey, 17.2% knew nothing about toxoplasmosis.
- 89.66% of the women surveyed improved by an average of 8.2 pts which is more than 100% improvement over their pre survey score.
- 6.90% of women remained the same.
- Only 3.45% of women score lowered.

Histogram for Each Question

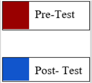

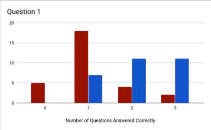

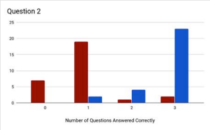

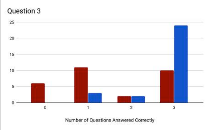

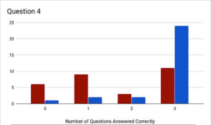

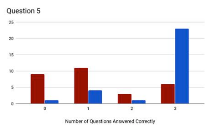

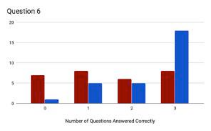

Summary Table

Question #	Chi-squared p value
1	0
2	0
3	0.000000694025598
4	0.00001331166034
5	0
6	0.0002820550319

THE UNIVERSITY OF CHICAGO | Center for Global Health

Comparison with Previous Panamanian Study

The women in this educational survey had the highest post-test scores for the educational pamphlet yet.

- The post-test score of 5.07 is the highest score achieved using this pamphlet.
- This is higher than the old pamphlet score vs new pamphlet survey in Panama of 4.63 and 4.83 respectively (Heichman 2016)
- This was also higher than the digital vs paper study scores of 4.77 and 4.88 respectively (Moreira, Pandey, 2016).

The individual answers were not recovered for the 2016 studies at this time. Thus, an in-depth analysis of T. gondii and toxoplasmosis knowledge between the two locations is not yet finalized.

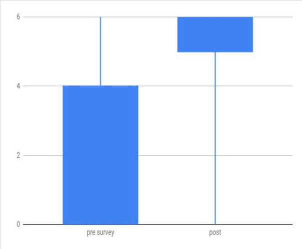

THE UNIVERSITY OF CHICAGO | Center for Global Health

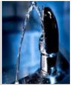

Conclusions and Future

- Testing educational pamphlet in Colombia clearly demonstrates it is effective in
- teaching pregnant women in Colombia quickly and efficiently
- Possible part of effort through which high seroprevalence rates are controlled
- This educational program was proven to be an effective instrument to deliver information in Colombia. We could now apply this educational program to other countries in Latin America.

- Implementation of an effective health program does not need to be expensive and resource intensive. Wit the availability of educational pamphlets, women would be able to access crucial information and at learn the information their own pace.
- Further exploration: Educational pamphlets can be used as part of targeted campaigns to reduce Toxoplas gondii seroprevalence in severely affected regions
- Data concerning risk factors unique to region
  - could modify the educational program to target changing certain behaviors emphasizing in pamphlet
  - Mold the program to each unique environment of each region and with Toxoplasma gondii prevalence data to add additional sophisticated effective educational techniques.

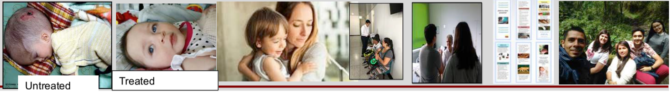

THE UNIVERSITY OF CHICAGO | Center for Global Health

Thank you!

Thank you to the Center for Global Health for funding this project and allowing me to travel to Colombia to perform this project.

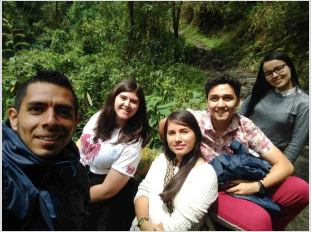

THE UNIVERSITY OF CHICAGO | Center for Global Health

¡Toxoplasmosis!
¡Toxoplasmosis!
¡Toxoplasmosis!

¿Qué es y cómo prevenirla?

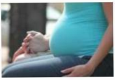

¡Toxoplasmosis!
¡Toxoplasmosis!
¡Toxoplasmosis!

El mayor riesgo es para las mujeres embarazadas, ya que si una mujer se infecta durante el embarazo puede pasar la enfermedad al bebé y causar lesiones graves.

Fuentes de Contagio

- Consumo de carne cruda o poco cocida.
- Tomar leche no pasteurizada, comer huevos crudos o poco cocidos.
- Consumir agua* contaminada.
- Llevarse a la boca las manos sucias de tierra o arena.
- Comer vegetales, frutas y verduras crudas sin lavar.
- Transmision de la madre con infección reciente al feto en el embarazo.
- En raras ocasiones a través de transfusiones de sangre y transplantes de órganos.

Diagnóstico

El diagnóstico de la enfermedad se realiza mediante exámenes de sangre. Es muy importante que las mujeres embarazadas acudad a Citas de control prenatal

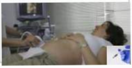

Tratamiento

Sólo el médico puede evaluar si tratar o no la infección.

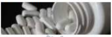

Prevención

- Lavar con agua* limpia las frutas y verduras crudas antes de comerlas.

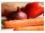

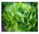

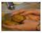

- Lavarse las manos antes de comer y después de tocar tierrea, verdudas sucias, animales, y caías de basuara

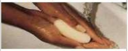

- Usar guantes al manipular cajas de suelo y arena

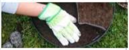

**Agua debe ser agua limpia*

Agua limpia es agua enbotellada, o hervida

No beber agua sucia

¿Qué es la Toxoplasmosis?

Es una enfermedad zoonótica (enfermedad que afecta a los seres humanos y a los animales). Es causada por un parasito llamado Toxoplasma gondii.

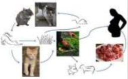

Síntomas

En la mayoría de los casos los síntomas son leves o no se presentan síntomas. En casos raros, puede haber complicaciones graves como afectaciones y/o pérdida de la vista.

Los síntomas pueden ser parecidos a la gripe, como: dolor de cabeza, malestar general, fiebre, dolores musculares, dolor en las articulaciones, fatiga, irritación e inflamación de ganglios linfáticos en la región de la ingle, el cuello y la axila.

- Evitar la presenciade moscas, cucarachas y hormigas cerca de comida y agua.
- Evitar comer carne cruda, cocine bien la carne, Evite consumir productos no pasteurizados, embutidos crudos o de dudosa procedencia.

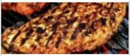

- No alimentar a los gatos con alimentos crudos
- No manipular las heces de los gatos.

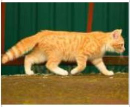

- Las embarazadas con infeccion reciente deben ser tratadas por su doctor para tratar de impedir la transmisión del parásito a su bebé.

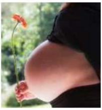

Acuda a sus citas de control prenatal.

Cualquier consulte o duda comuniquese con su ginecólogo o médico tratante.

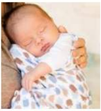

¿Quién no daría un poco por prevenir lo que daría mil mundos para curar?
-Edward Young

Adaptado de un folleto por de Moura, Amendoeira, Goulart, y de Moura
Creado por MS, XL, RM, IB, SH
