# Supplementary material for: Building Programs to Eradicate Toxoplasmosis Part II: Education
Source: Curr Pediatr Rep. Author manuscript; Available in PMC 2023 Mar 23. (PMC10035399; doi:10.1007/s40124-022-00267-y)
Supplement: 1832235_Sup_Material_2 [file NIHMS1832235-supplement-1832235_Sup_Material_2.pdf]

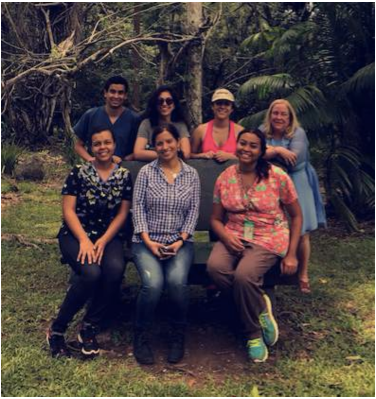

Catherine Castro

with Mariángela Soberón Felín, JD; Joseph Lykins, MD; Zuleima Caballero, PhD; Digna Wong, PhD; Oswaldo Reyes, MD; Rima McLeod, MD; and; Instituto de Investigaciones Científicas (INDICASAT) – Panama; Hospital Santo Tomás – Panama
Hospital San Miguel Arcángel – Panama

Impact of Gestational and Congenital Toxoplasmosis Medical Education: A Pre- and Post- Intervention Study in Panama City

Catherine Castro[1], Mariangela Soberón Felin[2], Joseph Lykins[1], Kristen Wroblewski[3], Rima McLeod MD [4,5]
[1] Pritzker School of Medicine, [2] INDICASAT AIP, [3] Department of Public Health Sciences, [4] Department of Ophthalmology and Visual Sciences, [5] Section of Infectious Diseases, Department of Pediatrics, Institute of Genomics, Genetics, and Systems Biology, Global Health Center, Toxoplasmosis Center, the Center for Health and the Social Sciences, CHeSS, the College, University of Chicago, Chicago, Illinois

Background

- Toxoplasmosis is a common parasitic infection and significant cause of morbidity and mortality worldwide, especially in Latin America
- Primary acquisition and vertical transmission of the causative agent, *Toxoplasma gondii*, can cause severe fetal brain and eye damage and fatal congenital disease
- Early diagnosis and treatment of acutely infected mothers markedly improves fetal health outcomes
- It is, therefore, important medical students and healthcare providers are knowledgeable about toxoplasmosis and the importance of gestational screening
- Studies that aim to assess the impact of toxoplasmosis education on patients, medical students, and healthcare providers are limited

Specific Aims

- Deliver a toxoplasmosis medical education intervention in Panama
- Assess impact of intervention on toxoplasmosis knowledge, knowledge confidence, and attitudes and beliefs
- Characterize routine gestational toxoplasmosis screening barriers
- Assess study participants' recommendations on how to successfully implement routine screening in Panama

Methods

Figure 1: Methods

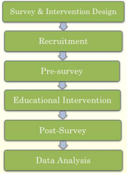

- PowerPoint presentation addressed various aspects of toxoplasmosis
- Pre- and post-surveys included a combination of true or false questions and attitudinal statements eliciting responses on a 5-point Likert scale
- Surveys asked participants to identify screening barriers and provide written feedback and comment on how they believe routine screening can be implemented in Panama
- Various statistical methods were used to asses demographics and changes from baseline

Results

Table 1: Demographics

Sample Characteristics (N=87)	N (%)
Medical Student (9th Semester)	51 (58.6%)
MD (All)	24 (27.6%)
Ob/Gyn Resident (PGY3)	12 (13.8%)

Figure 2: Mean knowledge score

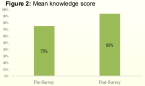

Figure 3: Knowledge confidence

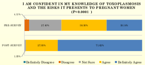

Table 2: Reported screening barriers

Barriers	(N, %)
Patient Education	33 (38%)
Cost	28 (32%)
Healthcare Access	21 (24%)
Disease Awareness	21 (24%)
Lack of Medical Supplies	21 (24%)
Lack of Medication	21 (24%)
Test Availability	18 (21%)

Figure 4: Proportion of respondents with a given score or lower out of 25

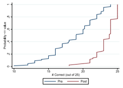

Table 3: Participant recommendations

Theme	Representative Quote
Implementation of affordable and accessible test	"It would be great if the $4 test was approved and more so the case, if the test could provide individual IgG and IgM results" (MD).
Involvement of MINSA or the government	"The tests for toxoplasmosis in Panama is MINSA's responsibility. Inform the department of epidemiology with studies like yours to create awareness and concern" (Pediatrician).
	"After completing your study, it is very important to send to MINSA your results. Extend this study to other hospitals to have a better understanding of the situation here in Panama" (Neonatologist).
Patient education	"To make toxoplasmosis screening routine a source of education for healthcare providers and the public is needed being that this country doesn't have a preventative mindset. It is necessary to transform the non-medical culture that we live in…" (Medical Student).
	"There is a need to educate pregnant women about the importance of pre-natal care and place greater emphasis in regions that don't have easy access to health care centers" (Medical Student).

Conclusions

- The intervention effectively improved toxoplasmosis knowledge, knowledge confidence, and attitudes and beliefs in a cohort of medical students, physicians, and OB/GYN residents
- Numerous screening barriers and recommendations were identified , which remain good targets for public health interventions
- More studies evaluating toxoplasmosis educational interventions can contribute to the development of more robust screening programs and help reduce the incidence and burden of this devastating congenital disease.

Limitations

- Sample size could be expanded
- Surveys can be further validated
- Intervention was carried out in a an urban setting and could be expanded to Panama's rural interior
- Future study directions can address study limitations.

Acknowledgements

- Zuleima Caballero PhD, Digna Wong PhD, Osvaldo Reyes MD, Natividad, Anabel Garcia DVM, Mario Quijada MPH, Guillermo Pradieu BA, Connie, Jeanne Farnan MD, Brian Callender MD, John Schneider MD
- Pritzker School of Medicine SRP
- (NIDDK) Grant #T35DK062719-30
- ASTMH Benjamin H. Kean Fellowship

References

- Torgerson PR, Mastroiacovo P. The global burden of congenital toxoplasmosis: a systematic review. Bull World Health Organ. 2013; 91(7):501-8. https://doi.org/10.2471/BLT.12.111732
- Rima McLeod, et al, Toxoplasmosis Study Group; Outcome of Treatment for Congenital Toxoplasmosis, 1981–2004: The National Collaborative Chicago-Based, Congenital Toxoplasmosis Study, Clinical Infectious Diseases, Volume 42, Issue 10, 15 May 2006, Pages 1383–1394, https://doi.org/10.1086/501360
- Begeman, I. et al. (2017). Point-of-care testing for Toxoplasma gondii IgG/IgM using the Toxoplasma ICT IgG-IgM test with sera from the United States and implications for developing countries. PLOS Neglected Tropical Diseases
- Wallon M, et al. Congenital toxoplasma infection: monthly prenatal screening decreases transmission rate and improves clinical outcome at age 3 years. Clin Infect Dis. 2013; 56(9):1223–31. https://doi.org/10.1093/cid/cit032 PMID: 23362291
- Sousa OE, et al. Toxoplasmosis in Panama: A 10-year study. Am J Trop Med Hyg. 1988;38(2):315–322
- McLeod, R., et al. (2009). Why prevent, diagnose and treat congenital toxoplasmosis? Memorias Do Instituto Oswaldo Cruz, 104(2), 320–344

Impact of gestational and congenital toxoplasmosis medical education: A pre and post- intervention study in Panama City, Panama

INTRODUCTION

Toxoplasmosis is a common disease and significant cause of morbidity and mortality globally [1]. The causative agent is, *Toxoplasma gondii,* an obligate apicomplexan intracellular parasite. Cats are the parasite's definitive host and human infection can result from direct or indirect consumption of oocysts produced and shed in the feces of infected cats. The majority of immunocompetent adults who acquire toxoplasmosis usually do not exhibit any recognizable symptoms. However, congenital toxoplasmosis (CT) can occur following primary maternal acquisition and vertical transmission and subsequently can cause severe ocular and neurological disease and death. Studies have shown that early diagnosis and treatment of mothers with acute toxoplasmosis can significantly reduce the risk of fetal transmission and severity of clinical symptoms [2]. Furthermore, improvements in clinical outcomes and reduced rates of morbidity and mortality in France, Austria, and Uruguay following implementation of mandatory screening for toxoplasmosis during gestation, provides further evidence of the critical role screening plays in preventing CT [3-7]. In addition, new advances in toxoplasmosis seroscreening have been made with the development of the *Toxoplasma* ICT IgG-IgM (LDBIO Diagnostic, Lyon, France; LDBIO), a low-cost point-of-care test has been found to be accurate for the detection of the infection in France and the U.S and is already being used in France [3]. This development may facilitate gestational toxoplasmosis screening and contribute to future improvements in maternal-fetal health care.

In 2012, the global annual incidence and burden of CT was estimated to be 1.5 cases per 1000 live births and 1.20 million disability-adjusted life years (DALYs) respectively [1]. Given the high global prevalence and burden of CT and evidence that demonstrates it is both preventable and treatable, it is important that healthcare providers and medical students are knowledgeable about toxoplasmosis and the benefits of gestational screening and early detection and treatment of acutely infected mothers. A prior review assessed the impact of toxoplasmosis health education on pregnant women in Belgium, Poland, Canada, and France and found preliminary evidence that suggests such education may help improve knowledge and prevention behavior and in turn, reduce the risk of CT [8]. However, studies that aim to provide and assess the impact of gestational and congenital toxoplasmosis education among medical students and/or healthcare providers have yet to be carried out.

The purpose of this study was to evaluate the impact of a gestational and congenital toxoplasmosis medical education intervention in Panama City, Panama. Panama was chosen as the study location because Latin America has one of the highest seroprevalence rates of *T. gondii* in the world [9]. A study done over a 10-year period in a rural population of 326 Panamanians showed that antibody prevalence increased from 25% in those 5 years of age, to 50% at 10 years of age, and increased gradually, reaching 90% by 60 years [10]. The estimated annual incidence of CT in Panama is 1.8 cases per 1000 live births and the estimated number of DALYS is 840 [1]. In light of this significant burden and high toxoplasma seroprevalence in Panama, we aim to assess the impact of an educational intervention on gestational and congenital toxoplasmosis knowledge, confidence in knowledge, attitudes and beliefs, and intent-to-recommend gestational screening among Panamanian medical students and healthcare providers.

We hypothesized that an educational intervention will improve the aforementioned endpoints compared to baseline as measured by pre- and post- intervention surveys. Furthermore, in 2014, Panama's Ministry of Health (MINSA) released an executive order mandating toxoplasmosis screening twice during gestation. However, there is no literature on implementation success or on barriers pregnant women may face in trying to access screening or healthcare professionals encounter in trying to provide screening in Panama. We also, therefore, aimed to characterize these barriers and gain an understanding of how participants believe routine gestational toxoplasmosis screening can be achieved in Panama.

METHODS

Educational intervention and survey development

The educational material and surveys were developed and translated into Spanish with the assistance of Dr. Rima McLeod, Director of the Toxoplasmosis Center at University of Chicago and several collaborators from Instituto de Investigaciones Científicas y Servicios de Alta Tecnología de Panamá (INDICASAT), Hospital Santo Tomás, and Hospital San Miguel Árcangel.

Recruitment

Various medical schools, hospitals, and clinics were contacted directly and asked to participate. Medical students enrolled in accredited Panamanian medical schools or healthcare providers in Panamanian hospitals or clinics that provide maternal-fetal healthcare were eligible to participate in the study.

Intervention

Participants were presented with an educational PowerPoint presentation approximately 25 minutes in length and written in Spanish that addressed:

i. Toxoplasmosis parasitology, prevalence, transmission, and prevention
ii. Congenital toxoplasmosis symptoms
iii. Gestational and congenital toxoplasmosis screening, diagnosis, and treatment
iv. Importance and benefits of gestational screening and early diagnosis and treatment of acutely infected mothers
v. Gestational toxoplasmosis screening and congenital toxoplasmosis reporting protocols set forth by the Ministry of Health (MINSA)
vi. *Toxoplasma* ICT IgG-IgM (LDBIO Diagnostic, Lyon, France; LDBIO)

Data Collection

Surveys written in Spanish were administered to the audience pre and post-intervention. Participants were asked to indicate whether they are a physician, resident, medical student, or nurse and indicate specialty and level of training if applicable. They were also prompted to add IDs in the format of (initials, birth mo, birth yr) to both pre-and post- surveys as unique and anonymous identifiers.

The surveys consisted of 25 true or false questions assessing knowledge related to gestational and congenital toxoplasmosis (Appendix). All tested items were addressed in the educational PowerPoint presentation. In addition, 12 questions eliciting responses on a 5 point Likert scale were included (1=Definitely Agree, 2=Agree; 3= Not Sure, 4= Disagree, 5=Definitely Disagree). These questions measured confidence in knowledge of gestational toxoplasmosis, beliefs and attitudes related to gestational and congenital toxoplasmosis, and intent-to recommend gestational screening.

At the conclusion of the post-survey, participants were prompted to choose which barriers they believe patients and physicians face in accessing and providing gestational toxoplasmosis screening respectively. They were also asked to provide feedback on the presentation and surveys as well as suggestions on how to implement routine gestational toxoplasmosis screening in Panama.

Data analysis

Raw pre- and post- test scores were coded and entered into an Excel spreadsheet. Coded data was analyzed in STATA to evaluate changes from baseline and intervention efficacy. Statistical tests utilized include McNemar's test to compare the set of 25 true or false questions assessing toxoplasmosis knowledge. Wilcoxon signed- rank test was used to analyze Likert data obtained from questions assessing toxoplasmosis knowledge, knowledge confidence, attitudes and beliefs, and intent-to recommend gestational screening. Kruskal-Wallis H test was

used to assess if results differed significantly based on level of training. A p-level of $p<0.05$ was used as significant. Descriptive statistics were used to characterize participant demographics and assess patient and physician screening barriers.

RESULTS

Participants

4 medical intervention sessions were carried out in 2 public hospitals, 1 healthcare clinic, and 1 medical school in Panama City, Panama between July 2017 and August 2017. The intervention reached 119 attendees, which included physicians, third-year OB/GYN residents, and 9th semester medical students. A 73% response rate was achieved with 87 participants completing both the pre-and post-intervention survey. Sample characteristics are described in Table 1a and 1b.

Table 1a: Study location summary (N=87)

Variable		N (%)
Medical School 1		
	Medical Student (9th Semester)	48
	Total	48 (55.17%)
Healthcare Clinic		
	MD (specialty not specified)	6
	MD (Internal Medicine)	5
	MD (Ob/Gyn)	1
	MD (Pediatrics)	1
	MD (Orthopedics)	1
	Total	14 (16.10%)
Public Hospital 1		
	Resident (Ob/Gym PGY-3)	12
	MD (Ob/Gyn)	1
	Total	13 (14.94%)
Public Hospital 2		
	MD (Pediatrics)	3
	MD (Neonatology)	2
	MD (specialty not specified)	2
	MD (Internal Medicine)	1
	MD (Ob/Gyn)	1
	Medical Student (9th Semester)	3
	Total	12 (13.79%)

Table 1b: Level of training summary (N=87)

Variable	N (%)
Medical Student (9th Semester)	51 (58.62%)
MD (All)	24 (27.59%)
Ob/Gyn Resident (PGY3)	12 (13.79%)

Knowledge

Analysis of the portion of the survey measuring knowledge of congenital and gestational toxoplasmosis revealed an increase in correct response rate compared to baseline. Cumulative distribution plots display a positive post-intervention shift in proportion of respondents with a given score or lower out of 25 (Figure 1) (Figure 2).

Figure 1: Proportion of respondents with a given score or lower out of 25

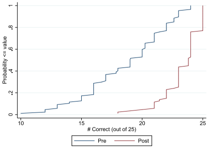

Figure 2: Proportion of respondents with a given score or lower out of 25 according to level of training

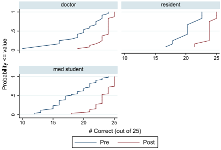

Furthermore, overall attainment knowledge was measured by assessing the pre- and post-intervention mean scores. An 18.32% increase from post-intervention mean score of 93.28% was seen when compared to the pre-survey mean score of 74.96%, (p<0.001) (Figure 3).

Figure 3: Mean Score: Pre- and Post- Intervention

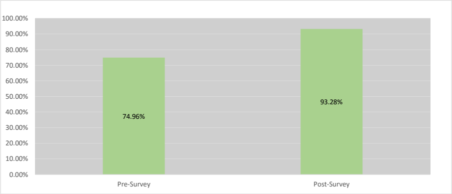

An increase in correct response rate was seen in 24 questions across all 6 categories measuring knowledge of gestational and congenital toxoplasmosis 1) symptoms 2) disease prevalence 3) transmission 4) fetal infection risk 5) diagnosis and 6) treatment. Statistically significant changes from baseline were observed in 18 survey questions (Table 2).

Table 2: Correct Response Rate

	Category	Pre-Survey	Post-Survey	P-Value
**Q1**	Congenital Symptoms	96.60%	100%	0.25
**Q2**	Congenital Symptoms	81.60%	100%	<0.0001*
**Q3**	Congenital Symptoms	70.10%	87.30%	0.0015*
**Q4**	Disease Prevalence	86.20%	94.30%	0.12
**Q5**	Congenital Symptoms	87.40%	96.60%	0.039*
**Q6**	Transmission	95.40%	97.80%	0.63
**Q7**	Transmission	82.80%	100%	0.0001*
**Q8**	Transmission	74.70%	98.90%	<0.0001*
**Q9**	Transmission	74.70%	80.50%	0.42
**Q10**	Transmission	97.70%	95.40%	0.63
**Q11**	Transmission	93.10%	100%	0.031*
**Q12**	Fetal Infection Risk	82.80%	92.00%	0.077
**Q13**	Diagnosis	93.10%	100%	0.031*
**Q14**	Diagnosis	60.90%	90.80%	<0.0001*
**Q15**	Diagnosis	63.20%	92%	<0.0001*
**Q16**	Fetal Infection Risk	56.30%	85.10%	<0.0001*
**Q17**	Fetal Infection Risk	51.70%	85.10%	<0.0001*
**Q18**	Treatment	54.00%	96.60%	<0.0001*
**Q19**	Treatment	92%	100%	0.016*
**Q20**	Treatment	60.90%	95.40%	<0.0001*
**Q21**	Treatment	59.80%	97.70%	<0.0001*
**Q22**	Diagnosis	68.50%	86.30%	0.0044*
**Q23**	Diagnosis	63%	89%	0.0003*
**Q24**	Treatment	23.30%	67%	<0.0001*
**Q25**	Treatment	91.80%	98.60%	0.063

The biggest improvements were observed in questions related to treatment of gestational and congenital toxoplasmosis. For example, the percentage of respondents correctly identifying spiramycin as a medication that can be administered to acutely infected mothers early in gestation to help block fetal transmission of *T. gondii* rose from 54% in the pre-survey to 96.6% in the post-survey. Furthermore, the percentage of participants who were able to correctly identify that treatment is not needed for immunocompetent pregnant women if they acquired toxoplasmosis 6 months or more prior to conception increased from 23.3% to 67.0% in the pre- and post-survey respectively. Questions assessing knowledge of fetal infection risk and diagnosis are also among the questions that showed the largest improvements in correct response rate compared to baseline. For example, 85.1% of post-survey respondents correctly identified the statement, “A low risk of fetal infection exists if a pregnant woman acquires toxoplasmosis in her second and third trimester”, as false compared to 51.7% of pre-survey respondents. In addition, 90.8% of post-survey respondents correctly identified “Amniocentesis cannot be used to diagnose CT in the fetus”, as a false statement compared to 60.9% of pre-survey respondents.

Confidence in knowledge

Analysis of the portion of the survey measuring confidence in knowledge on a 5 point Likert scale (1=Definitely Agree; 5=Definitely Disagree), revealed that a greater percentage of post-intervention respondents definitely agreed or agreed they are confident in their knowledge of gestational toxoplasmosis, diagnosis, and treatment as well screening and reporting protocols set forth by the Ministry of Health (MINSA) compared to baseline (Figure 4).

Figure 4:

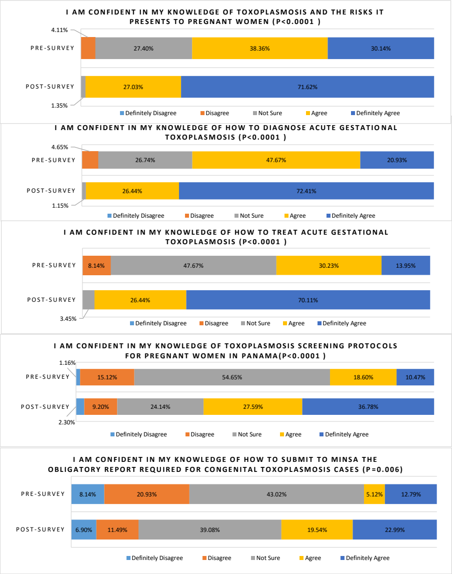
I AM CONFIDENT IN MY KNOWLEDGE OF TOXOPLASMOSIS AND THE RISKS IT PRESENTS TO PREGNANT WOMEN (P<0.0001)
PRE-SURVEY
POST-SURVEY
4.11%
27.40%
38.36%
30.14%
27.03%
71.62%
1.35%
Definitely Disagree
Disagree
Not Sure
Agree
Definitely Agree
I AM CONFIDENT IN MY KNOWLEDGE OF HOW TO DIAGNOSE ACUTE GESTATIONAL TOXOPLASMOSIS (P<0.0001)
4.65%
26.74%
47.67%
20.93%
26.44%
72.41%
1.15%
I AM CONFIDENT IN MY KNOWLEDGE OF HOW TO TREAT ACUTE GESTATIONAL TOXOPLASMOSIS (P<0.0001)
8.14%
47.67%
30.23%
13.95%
26.44%
70.11%
3.45%
I AM CONFIDENT IN MY KNOWLEDGE OF TOXOPLASMOSIS SCREENING PROTOCOLS FOR PREGNANT WOMEN IN PANAMA(P<0.0001)
1.16%
15.12%
54.65%
18.60%
10.47%
9.20%
24.14%
27.59%
36.78%
2.30%
I AM CONFIDENT IN MY KNOWLEDGE OF HOW TO SUBMIT TO MINSA THE OBLIGATORY REPORT REQUIRED FOR CONGENITAL TOXOPLASMOSIS CASES (P=0.006)
8.14%
20.93%
43.02%
5.12%
12.79%
6.90%
11.49%
39.08%
19.54%
22.99%

98.65% of post-survey respondents definitely agreed or agreed they were confident in their knowledge of toxoplasmosis and the risks it presents to pregnant women compared to 68.5% of pre-survey respondents (p <0.0001). 98.85% of post-survey respondents definitely agreed or agreed they were confident in their knowledge of toxoplasmosis and the risks it presents to pregnant women compared to 68.60% of pre-survey respondents (p <0.0001). 96.55% of post-survey respondents definitely agreed or agreed they were confident in their knowledge of how to treat congenital toxoplasmosis compared to 44.18% of pre-survey respondents (p <0.0001). 64.37% of post-survey respondents definitely agreed or agreed they were confident in their knowledge of toxoplasmosis screening protocols for pregnant women in Panama compared to 29.07% of pre-survey respondents (p <0.0001). 42.53% of post-survey respondents definitely agreed or agreed they were confident in their knowledge of how to report congenital toxoplasmosis cases to MINSA compared to 17.91% of pre-survey respondents (p=0.006).

Attitudes and beliefs
Significant changes from baseline were seen in the portion of the survey assessing attitudes and beliefs towards congenital and gestational toxoplasmosis (Figure 5).

Figure 5:

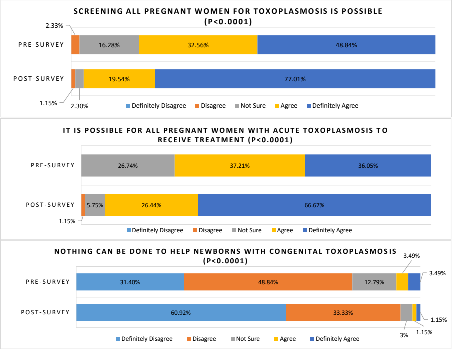

A greater percentage of post-survey respondents compared to baseline definitely agreed or agreed that screening all pregnant women for toxoplasmosis is possible. 81.40% of pre-survey respondents definitely agreed or agreed with this statement compared to 96.55% of post-survey respondents (p <0.0001).

A greater percentage of post-survey respondents compared to baseline also definitely agreed or agreed that it is possible for all pregnant women with acute toxoplasmosis to receive treatment. 73.26% of pre-survey respondents definitely agreed or agreed with this statement compared to 93.11% of post-respondents (p <0.0001).

Compared to baseline, a greater percentage of post-survey respondents definitely disagreed or disagreed with the statement that nothing can be done to help newborns with congenital toxoplasmosis. 80.24% of pre-survey respondents definitely disagreed or disagreed with this statement compared to 94.25% of post-survey respondents (p <0.0001).

Intent-to-recommend gestational toxoplasmosis screening
The majority of respondents agreed that they would recommend all pregnant women get screened for toxoplasmosis both at baseline (96.52%) and post-intervention (97.7%) (Figure 6). 60.47% of pre-respondents definitely agreed with this statement compared to 81.61% of post-respondents (p=0.002).

Figure 6:

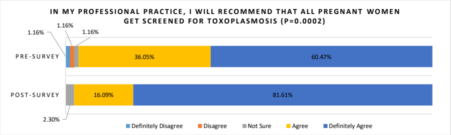

Barriers
Participants reported patient education, healthcare access, cost, test-availability, and disease awareness as barriers they believe pregnant women face when trying to access gestational toxoplasmosis screening and treatment. The most commonly selected barriers were patient education and cost with a selection frequency of 37.93% and 32.18% respectively (Table 3).

Table 3: Patient Barriers

Variable	(N, %)
Patient Education	33 (37.93%)
Cost	28 (32.18%)
Healthcare Access	21 (24.14%)
Disease Awareness	21 (24.14%)

Participants reportedlack of medical supplies, lack of medication, cost, test availability, physician knowledge, and patient volume, as barriers physicians face in screening for and treating toxoplasmosis. The most commonly selected barriers were lack of medical supplies and medications and cost with a selection frequency of 25.3%, 24.1%, and 24.1% respectively (Table 4).

Table 4: Physician Barriers

Variable	(N, %)
Lack of Medical Supplies	22 (25.29%)
Lack of Medication	21 (24.14%)
Cost	21 (24.14%)
Test Availability	18 (20.68%)
Physician Knowledge	5 (5.75%)
Patient Volume	5 (5.75%)

FEEDBACK

Table: 5

Theme	Representative Quote
Implementation of affordable and accessible test	"A good way to make toxoplasmosis screening possible would be with the reactive strips especially with the low cost" (Medical Student).
	"It would be great if the $4 test was approved and more so the case, if the test could provide individual IgG and IgM results" (MD).
Involvement of MINSA or the government	"The tests for toxoplasmosis in Panama is MINSA's responsibility. Inform the department of epidemiology with studies like yours to create awareness and concern" (Pediatrician).
	"After completing your study, it is very important to send to MINSA your results. Extend this study to other hospitals to have a better understanding of the situation here in Panama" (Neonatologist).
	"Campaigns should be brought to authorities' attention so medical supplies are available" (Neonatologist).
	"First and foremost, the appropriate authorities should be informed so that the issue of test availability is brought to a national level, so that there exists a budget designed for this disease (Medical Student).
Educating patients and the public	"Campaigns that makes the harm to exposed mothers and infected fetuses more visible so that the population can also ask the government for the tests" (Medical Student).
	"There should be educational and preventative public health campaigns in areas with difficult access" (Medical Student).
	"To make toxoplasmosis screening routine a source of education for healthcare providers and the public is needed being that this country doesn't have a preventative mindset. It is necessary to transform the non-medical culture that we live in…" (Medical Student).
Improvement of pre-natal care	"Improve pre-natal care" (MD).
	"There is a major advantage in early detection of this infection. The problem is that in Panama many women don't get pre-natal care making detection difficult (Medical Student).
	"There is a need to educate pregnant women about the importance of pre-natal care and place greater emphasis in regions that don't have easy access to health care centers (Medical Student).
Education for healthcare providers	"Keeping healthcare personnel up-to-date so they can provide patients adequate information" (Medical Student)

72 respondents provided feedback on the intervention in free-response form upon completion of the post-survey. The intervention was positively received. Participants also provided suggestions on how to make routine gestational screening for toxoplasmosis possible in Panama. 5 themes were identified and representative quotes are included below (Table 5). 12 participants stated that a more affordable and accessible test would make implementation of routine gestational screening feasible and more accessible to the general population. 9 participants indicated government or Ministry of Health (MINSA) involvement was needed. 7 respondents recommended providing education to patients and/or the public. 5 participants suggested improving pre-natal care and 5 participants suggested continuing medical education for healthcare professionals.

DISCUSSION

The purpose of this study was to evaluate the impact of a gestational and congenital toxoplasmosis educational intervention among medical students and healthcare providers in Panama, a country with a high prevalence of toxoplasmosis and burden of congenital toxoplasmosis. This was accomplished with the use of surveys administered pre- and post- intervention.

Statistically significantly increases in correct response rates post-intervention were observed in 18 out of 25 true or false questions measuring knowledge of toxoplasmosis 1) symptoms 2) disease prevalence 3) transmission 4) fetal infection risk 5) gestational and fetal diagnosis and 6) gestational and fetal treatment suggesting the medical education provided allowed participants to acquire new knowledge in each of these areas respectively. The biggest improvements were observed in questions related to knowledge of treatments for gestational and congenital toxoplasmosis. Questions measuring knowledge of toxoplasmosis fetal infection risk and diagnosis are also among the questions that showed the largest improvements in correct response rate compared to baseline. This demonstrates that the educational intervention may be valuable to study participants in their clinical practice.

A comparison between the mean pre-survey score of 75% and mean post-survey score of 93.3% revealed the educational intervention was also effective in improving overall disease knowledge. These findings appeared to be fairly consistent across physicians, residents, and medical students as demonstrated by cumulative distribution plots that illustrated a positive post- intervention shift in the proportion of participants with a given score or lower of correct answers out of 25. In sum, analysis of the portion of the surveys assessing knowledge revealed a significant increase in knowledge about gestational and congenital toxoplasmosis compared to baseline. This illustrates this educational intervention's potential to effectively enhance the knowledge medical students and healthcare providers possess about toxoplasmosis, which, may have positive implications for maternal-fetal healthcare.

Statistically significant differences pre- and post-intervention were also observed in participants' self-rated confidence in their toxoplasmosis-related knowledge. Post-intervention, a greater percentage of participants definitely agreed or agreed they were confident in their knowledge of gestational toxoplasmosis and the risks it presents to pregnant women, diagnosis, and treatment. This suggests that in addition to the intervention resulting in new knowledge acquisition, participants also gained more confidence in that knowledge.

Compared to baseline, participants also reported higher confidence in their knowledge of gestational toxoplasmosis screening and congenital toxoplasmosis reporting protocols set forth by Panama's Ministry of Health (MINSA). Despite greater confidence in this knowledge post-intervention, a significant proportion of participants still reported lack of confidence in their understanding of these protocols. 57.5% of post-survey respondent's participants reported they were not confident in their knowledge of reporting protocols for CT and 35.6% of post-survey respondents reported they were not confident in their knowledge of gestational toxoplasmosis screening protocols in Panama. Therefore, the educational intervention did not sufficiently

address this lack of confidence and future education from MINSA may be necessary to raise protocol awareness and confidence among medical students and healthcare providers.

The intervention also had a positive impact on participants' attitudes and beliefs towards toxoplasmosis and gestational screening. A statistically significant greater percentage of respondents compared to baseline definitely agreed or agreed that it is possible to screen all pregnant women for toxoplasmosis and treat all acutely infected mothers. Furthermore, compared to baseline, a greater percentage of respondents definitely disagreed or disagreed with the statement that nothing can be done to help newborns with congenital toxoplasmosis. This shift in attitudes may be attributed to data included in the educational intervention that highlighted the benefits of early screening, treatment, and advancements in point-of-care testing.

Finally, the majority of participants definitely agreed or agreed they would recommend gestational toxoplasmosis screening in their clinical practice pre-intervention (96.52%) and post-intervention (97.7%). However, 81.61% of post-respondents definitely agreed with this statement compared to 60.47% of pre-respondents. The educational intervention, therefore, helped to strengthen participants' intent-to-recommend gestational toxoplasmosis screening. A greater commitment to routine gestational toxoplasmosis screening can contribute to efforts to identify and treat acutely infected mothers and better fetal-health outcomes.

Despite, the fact that most participants agreed they would recommend gestational toxoplasmosis screening in their clinical practice, potential barriers to implementation of routine screening may exist. We aimed to assess these barriers and although screening is mandatory by law in Panama, participants cited many barriers that challenge healthcare providers' ability to offer gestational toxoplasmosis screening and patients' ability to seek it. Patient education and cost were the most commonly reported patient barriers while lack of medical supplies, lack of medications, and cost were the most commonly selected physician barriers. These findings were confirmed by feedback and suggestions participants voluntarily provided at the conclusion of the intervention. Both medical students and physicians placed a great emphasis on patient education and recommended that public health campaigns about toxoplasmosis be carried out in the rural interior of the country where there is less access to healthcare. Physicians also stressed that addressing lack of medical supplies and test availability is an important angle from which to attack the challenge of implementing routine gestational toxoplasmosis screening. In particular, they highlighted the crucial role they believe the Ministry of Health should play in ensuring access to screening and treatment. Improving pre-natal care in Panama was another theme commonly identified in the feedback. A more robust pre-natal care program in Panama may be the solution to ensuring that all pregnant women receive the appropriate care, tests, and needed treatments during gestation.

Study limitations include the small and limited sample size. Future research with a larger and more diverse sample size would be ideal to assess the intervention efficacy. A larger sample size and more representative sample of various backgrounds and levels of training would provide more power in study results. For example, future studies would ideally also look at the impact of this educational intervention on other healthcare providers such as nurses. Other study limitations include the fact that participants weren't randomly selected and that several participants were excluded from the study because they did not complete either the pre or post-survey. Also, because no prior studies assessing the impact of toxoplasmosis medical education on medical students and healthcare providers, the surveys used in our study were newly designed and edited rigorously but not officially validated. Future study directions include validating surveys, collaborating with MINSA to disseminate this information in hospitals and clinics across metropolitan and rural Panama in an effort to continue spreading disease awareness, implementation of toxoplasmosis point-of-care testing, and subsequently measuring screening compliance rates and its impact on clinical care in Panama.

CONCLUSION

Toxoplasmosis is a common infection globally that poses a significant risk to pregnant mothers and their babies. Primary acquisition and vertical transmission of *T. gondii* can cause severe, debilitating, and potentially fatal congenital disease. Given the critical role early diagnosis and treatment of acutely mothers plays in improving clinical outcomes, it is important that medical students and healthcare providers are knowledgeable about toxoplasmosis. This study demonstrated that gestational and congenital toxoplasmosis medical education effectively improved toxoplasmosis knowledge, knowledge confidence, attitudes and beliefs, and intent-to-recommend gestational screening compared to baseline in a study cohort of medical students, physicians, and OB/GYN residents. Furthermore, patient and physician gestational toxoplasmosis screening barriers were characterized and remain good targets for future interventions involving the Panamanian Ministry of Health (MINSA). More education related to toxoplasmosis and studies evaluating the efficacy and impact of educational interventions on screening compliance and the incidence of CT are needed in both the general population and medical community in Panama. Such research can contribute to the development of a more robust gestational toxoplasmosis screening programs and in turn, help reduce the incidence and burden of this devastating congenital disease.

ACKNOWLEDGMENTS

Dr. Rima McLeod, Director of Toxoplasmosis Center at the University of Chicago, for her assistance with study design, intervention and survey development, coordination of study logistics, and recruitment. Mariangela Soberón Felín JD, Coordinator of Toxoplasmosis Programs and Initiatives in Panama and Zuleima Caballero PhD from INDICASAT for their assistance with intervention and survey development, Spanish translations, coordination of study logistics and recruitment. Digna Wong PhD from INDICASAT, Dr. Osvaldo Reyes, Director of Hospital Santo Tomás Department of Obstetrics, and Dr. Natividad Caballero from Hospital San Miguel Arcángel for their assistance with recruitment. Kristen Wroblewski M.S. from the Department of Public Health Sciences at the University of Chicago for her statistical support and analysis of study results. Anabel Garcia, DVM and Mario Quijada, MPH, for their assistance with Spanish translations. The Pritzker School of Medicine, National Institute of Diabetes and Digestive and Kidney Diseases (NIDDK) Grant #T35DK062719-30, Thrasher Fund, ASTMH Benjamin H. Kean Fellowship in Tropical Medicine, and INDICASAT for their financial and/or logistical support.

Appendix:

	T/F Statement	Answer
**Q1**	Toxoplasmosis can cause severe brain disease to babies if transmitted across the placenta during gestation.	T
**Q2**	Toxoplasmosis can cause severe eye disease to babies if transmitted across the placenta during gestation.	T
**Q3**	In general, toxoplasmosis produces recognizable symptoms in healthy adults.	F
**Q4**	Toxoplasmosis is not a common infection in Panama	F
**Q5**	Toxoplasmosis cannot cause blindness in children.	T
**Q6**	*Toxoplasma gondii* can be transmitted to humans via direct contact with cat feces.	T
**Q7**	*Toxoplasma gondii* can be transmitted to humans via direct contact with surfaces or objects that have come in contact with cat feces.	T
**Q8**	*Toxoplasma gondii* can be transmitted via consumption of poorly washed foods.	T
**Q9**	*Toxoplasma gondii* cannot be transmitted via the consumption of poorly cooked foods.	F
**Q10**	Health pregnant women cannot acquire toxoplasmosis during gestation.	F
**Q11**	A woman can acquire toxoplasmosis at any point during gestation.	T
**Q12**	If a pregnant woman does not present with symptoms of toxoplasmosis, she cannot transmit the infection to her fetus.	F
**Q13**	Acute primary toxoplasmosis infection can be diagnosed with serologic tests during gestation.	T
**Q14**	Amniocentesis cannot be used to diagnose congenital toxoplasmosis in the fetus.	F
**Q15**	Ultrasounds can be used to help diagnoses fetal signs of congenital toxoplasmosis.	T
**Q16**	If a pregnant woman acquired toxoplasmosis before conception and she has a normal immune response, there is a low risk of fetal infection.	T
**Q17**	If a pregnant woman acquires toxoplasmosis in the second or third and she has a normal immune response, there is a low risk of fetal infection.	F
**Q18**	Spiramycin can be given to seropositive pregnant woman early in gestation to help block transmission of *Toxoplasma gondii* to the fetus.	T
**Q19**	Early diagnosis and treatment of acute toxoplasmosis in pregnant woman improves fetal health outcomes.	T
**Q20**	Severe neurological disease can be prevented in newborns if mothers with acute toxoplasmosis are treated with pyrimethamine, sulfadiazine, and folinic acid in the second and third trimester.	T
**Q21**	Future ocular damage in children can prevented if mothers with acute toxoplasmosis are treated with pyrimethamine, sulfadiazine, and folinic acid in the second and third trimester.	T
**Q22**	An IgG positive serological result indicates recent toxoplasmosis infection.	F
**Q23**	An IgM positive serological results can indicate recent toxoplasmosis infection.	T
**Q24**	A pregnant woman that acquired toxoplasmosis 6 months or more prior to conception should receive treatment to help prevent congenital toxoplasmosis.	F
**Q25**	A pregnant woman that acquires toxoplasmosis for the first time during gestation should receive treatment to help prevent congenital toxoplasmosis.	T

Appendix Con't:

	**Likert Data Questions**
**Q26**	Nothing can be done to help newborns with congenital Toxoplasmosis.
**Q27**	Congenital toxoplasmosis is preventable.
**Q28**	Patients are well-informed about toxoplasmosis and the risks it presents to pregnant women.
**Q29**	It is important to screen pregnant women for gestational toxoplasmosis.
**Q30**	Screening all pregnant women for toxoplasmosis is possible.
**Q31**	It is possible for all pregnant women with acute toxoplasmosis to receive treatment.
**Q32**	I am confident in my knowledge of toxoplasmosis and the risks it presents to pregnant women.
**Q33**	I am confident in my knowledge of how to diagnose acute toxoplasmosis during gestation.
**Q34**	I am confident in my knowledge of how to treat acute toxoplasmosis during gestation.
**Q35**	I am knowledgeable of the protocols used for screening pregnant women for toxoplasmosis in Panama.
**Q36**	I am knowledgeable of how to submit to MINSA the obligatory report required for congenital toxoplasmosis cases.
**Q37**	In my professional practice, I will recommend that all pregnant women get screened for toxoplasmosis.

REFERENCES

1. Torgerson PR, Mastroiacovo P. The global burden of congenital toxoplasmosis: a systematic review. Bull World Health Organ. 2013; 91(7):501–8. https://doi.org/10.2471/BLT.12.111732
2. Rima McLeod, Kenneth Boyer, Theodore Karrison, Kristen Kasza, Charles Swisher, Nancy Roizen, Jessica Jalbrzikowski, Jack Remington, Peter Heydemann, A. Gwendolyn Noble, Marilyn Mets, Ellen Holfels, Shawn Withers, Paul Latkany, Paul Meier, Toxoplasmosis Study Group; Outcome of Treatment for Congenital Toxoplasmosis, 1981–2004: The National Collaborative Chicago-Based, Congenital Toxoplasmosis Study, *Clinical Infectious Diseases*, Volume 42, Issue 10, 15 May 2006, Pages 1383–1394, https://doi.org/10.1086/501360
3. Begeman, I., Lykins, J., Zhou, Y., Lai, B.S., Levigne, P., El Bissati, K., Boyer, K., Withers, S., Clouser, F., Noble, A.G., Rabiah, P., Swisher, C.N., Heydemann, P.T., Contopoulos-Ioannidis, D.G., Montoya, J.G., Maldonado, Y., Ramirez, R., Press, C., Stillwaggon, E., Peyron, F., McLeod, R.. (2017). Point-of-care testing for *Toxoplasma gondii* IgG/IgM using the *Toxoplasma* ICT IgG-IgM test with sera from the United States and implications for developing countries. *PLOS Neglected Tropical Diseases*. Accepted.
4. Wallon M, Peyron F, Cornu C, Vinault S, Abrahamowicz M, Kopp CB, et al. Congenital toxoplasma infection: monthly prenatal screening decreases transmission rate and improves clinical outcome at age 3 years. Clin Infect Dis. 2013; 56(9):1223–31. https://doi.org/10.1093/cid/cit032 PMID: 23362291
5. Prusa AR, Kasper DC, Sawers L, Walter E, Hayde M, Stillwaggon E. Congenital toxoplasmosis in Aus- tria: prenatal screening for prevention is cost-saving. PLoS Negl Trop Dis. In press, 2017.
6. Hotop A, Hlobil H, Gross U. Efficacy of rapid treatment initiation following primary Toxoplasma gondii infection during pregnancy. Clin Infect Dis. 2012; 54(11):1545–52. https://doi.org/10.1093/cid/cis234 PMID: 22460980
7. Kieffer F, Wallon M, Garcia P, Thulliez P, Peyron F, Franck J. Risk factors for retinochoroiditis during the first 2 years of life in infants with treated congenital toxoplasmosis. Pediatr Infect Dis J. 2008; 27 (1):27–32. https://doi.org/10.1097/INF.0b013e318134286d PMID: 18162934
8. Mario S, Basevi V, Gagliotti C, Spettoli D, Gori G, D'Amico R, Magrini N. Prenatal education for congenital toxoplasmosis. Cochrane Database of Systematic Reviews2015, Issue 10. Art. No.: CD006171. DOI: 10.1002/14651858.CD006171.pub4.
9. G. Pappas, N. Roussos, M.E. Falagas. Toxoplasmosis snapshots: global status of Toxoplasma gondii seroprevalence and implications for pregnancy and congenital toxoplasmosis**.** Int. J. Parasitol., 39 (2009), pp. 1385-1394
10. Sousa OE, Saenz RE, Frenkel JK. Toxoplasmosis in Panama: A 10-year study. Am J Trop Med Hyg. 1988;38(2):315–332.
11. McLeod, R., Kieffer, F., Sautter, M., Hosten, T., & Pelloux, H. (2009). Why prevent, diagnose and treat congenital toxoplasmosis? *Memorias Do Instituto Oswaldo Cruz*, *104*(2), 320–344.

Impact of Gestational and Congenital Toxoplasmosis Medical Education: A Pre- and Post- Intervention Study in Panama City

By: Catherine Castro
Mentor: Rima McLeod, M.D.

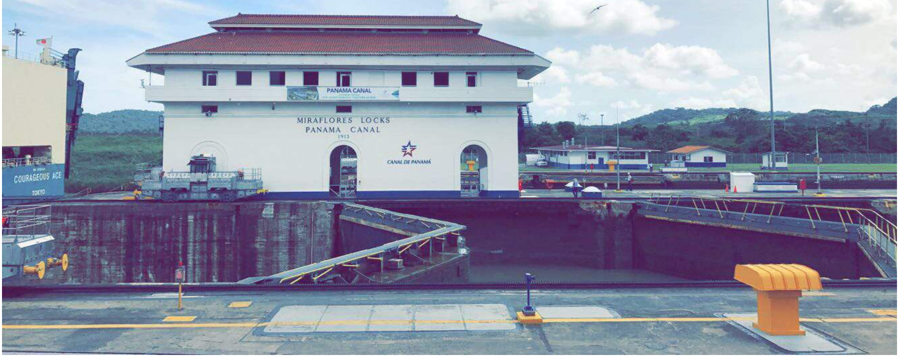

Introduction

- Toxoplasmosis is present in approximately 30–50% of the world population.

- **In Panama, a high seroprevalence of *T. gondii* exists**
  - A previous study revealed a seroprevalence rate ranging from 25% to 90% in those 5 and 60 years of age respectively.

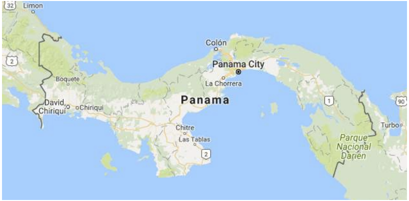

Introduction

- Primary acquisition of *T. gondii* during gestation can lead to **congenital infection.**

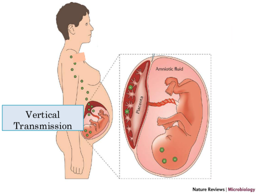

- Early diagnosis and treatment of acutely infected mothers significantly reduces fetal transmission risk and severity of clinical symptoms.

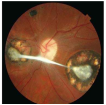

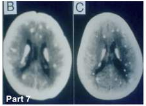

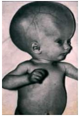

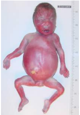

Aims

- To assess the impact of an educational intervention on gestational and congenital toxoplasmosis for medical students and healthcare providers

- To characterize screening barriers and how participants believe routine screening can be achieved in Panama

Hypothesis

- A gestational and congenital toxoplasmosis medical education intervention will improve toxoplasmosis:
  - Knowledge
  - Knowledge confidence
  - Attitudes and beliefs

- Survey responses will allow characterization of screening barriers.

Methods

- Participants recruited from several Panamanian medical schools, hospitals, and clinics
- Surveys were a combination of T/F questions and attitudinal statements eliciting responses on a 5-point Likert scale
- Intervention addressed various aspects of toxoplasmosis
- Statistical methods: descriptive statistics, McNemar's, Wilcoxon signed-rank, and Kruskal-Wallis H tests

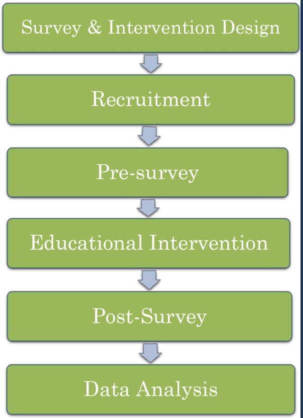

Results: Demographics

Sample Characteristics (N=87)	N (%)
Medical Student (9th Semester)	51 (58.62%)
MD (All)	24 (27.59%)
Ob/Gyn Resident (PGY3)	12 (13.79%)

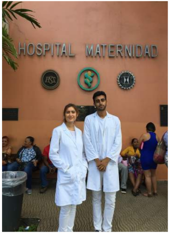

Results: Knowledge

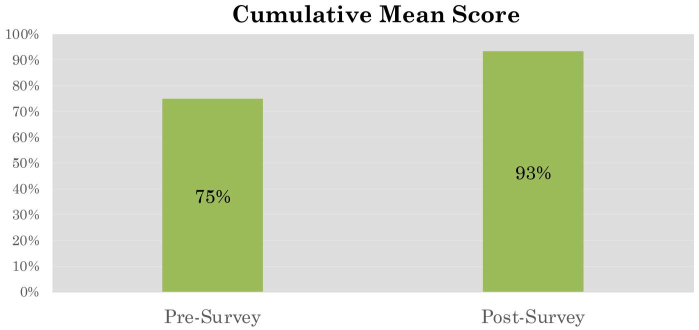

18% increase in cumulative mean score post-intervention when compared to baseline ($p<0.001$)

Results: Knowledge

- Statistically significant increases in correct response rates were observed in 18 items
- The biggest improvements were observed in questions related to **treatment**
- Large improvements in knowledge of **diagnosis and fetal infection risk**

Correct Response Rate

	Category	Pre-Survey	Post-Survey	P-Value
Q1	Congenital Symptoms	96.60%	100%	0.25
Q2	Congenital Symptoms	81.60%	100%	<0.0001*
Q3	Congenital Symptoms	70.10%	87.30%	0.0015*
Q4	Disease Prevalence	86.20%	94.30%	0.12
Q5	Congenital Symptoms	87.40%	96.60%	0.039*
Q6	Transmission	95.40%	97.80%	0.63
Q7	Transmission	82.80%	100%	0.0001*
Q8	Transmission	74.70%	98.90%	<0.0001*
Q9	Transmission	74.70%	80.50%	0.42
Q10	Transmission	97.70%	95.40%	0.63
Q11	Transmission	93.10%	100%	0.031*
Q12	Fetal Infection Risk	82.80%	92.00%	0.077
Q13	Diagnosis	93.10%	100%	0.031*
Q14	Diagnosis	60.90%	90.80%	<0.0001*
Q15	Diagnosis	63.20%	92%	<0.0001*
Q16	Fetal Infection Risk	56.30%	85.10%	<0.0001*
Q17	Fetal Infection Risk	51.70%	85.10%	<0.0001*
Q18	Treatment	54.00%	96.60%	<0.0001*
Q19	Treatment	92%	100%	0.016*
Q20	Treatment	60.90%	95.40%	<0.0001*
Q21	Treatment	59.80%	97.70%	<0.0001*
Q22	Diagnosis	68.50%	86.30%	0.0044*
Q23	Diagnosis	63%	89%	0.0003*
Q24	Treatment	23.30%	67%	<0.0001*
Q25	Treatment	91.80%	98.60%	0.063

Results: Knowledge

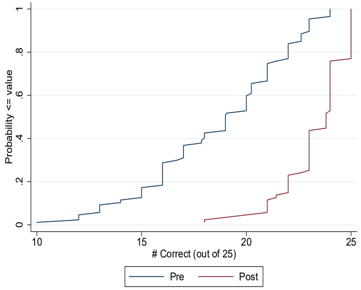

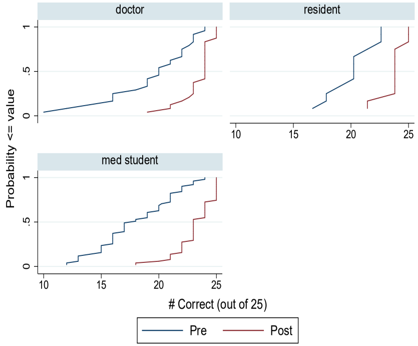

Positive post-intervention shift in proportion of respondents with a given score or lower out of 25

Results: Knowledge Confidence

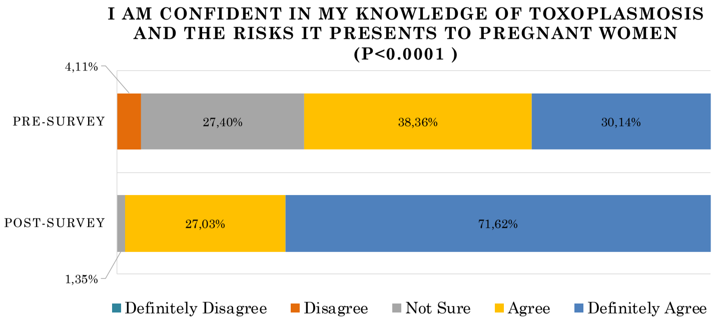

Results: Knowledge Confidence

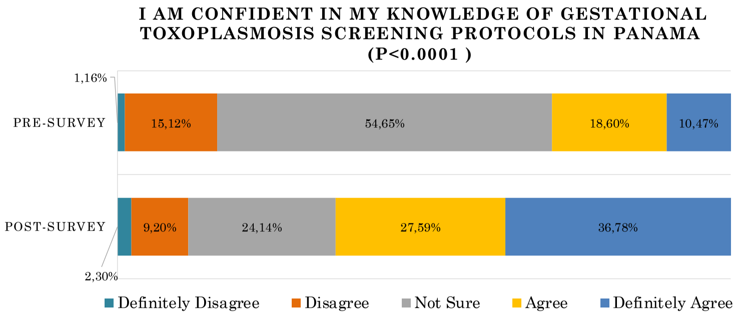

Results: Attitudes and Beliefs

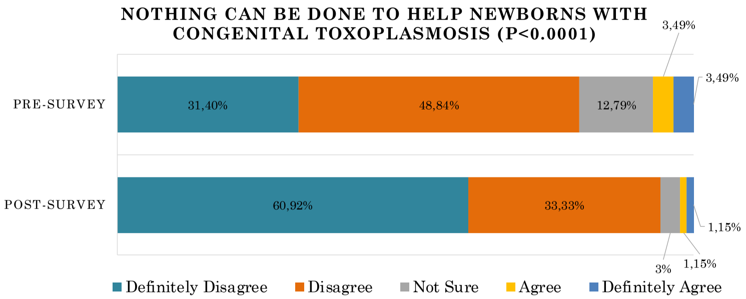

Results: Barriers

Patient Barriers:	(N, %)
Patient Education	33 (37.93%)
Cost	28 (32.18%)
Healthcare Access	21 (24.14%)
Disease Awareness	21 (24.14%)

Physician Barriers:	(N, %)
Lack of Medical Supplies	22 (25.29%)
Lack of Medication	21 (24.14%)
Cost	21 (24.14%)
Test Availability	18 (20.68%)
Physician Knowledge	5 (5.75%)
Patient Volume	5 (5.75%)

Conclusion

Toxoplasmosis medical education can improve toxoplasmosis knowledge, knowledge confidence, and attitudes and beliefs, with many spill-over benefits.

Limitations

- Small and limited sample size

- Intervention has not reached healthcare centers in Panama's rural interior

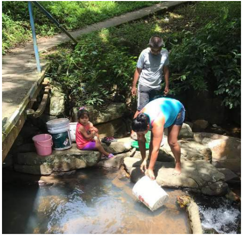

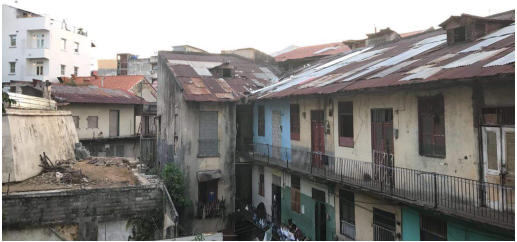

Future Directions

- Expand study to other schools and healthcare centers especially in areas with difficult access
- Collaborate with Ministry of Health (MINSA) to disseminate educational materials and coordinate public health campaigns
- Implementation of multiplexed test for preventable congenital infections and toxoplasmosis point-of-care testing

All these have implications for improving maternal-fetal and pediatric healthcare in Panama

Acknowledgments

Rima McLeod MD, FACP, FIDSA
Mariangela Soberón Felín JD Coordinator of Toxoplasmosis Programs and Initiatives in Panama
Zuleima Caballero PhD investigator at INDICASAT
Kristen Wroblewski, M.S. Department of Public Health Sciences
Digna Wong PhD Coordinator at INDICASAT
Osvaldo Reyes MD, Director of HST Department of Obstetrics
Natividad Caballero MD, Neonatologist at Hospital San Miguel Arcángel
Jeanne Farnan MD, MHPE at University of Chicago
Brian Callender, MD Professor at University of Chicago
John Schneider, MD, MPH Professor at University of Chicago
Anabel Garcia, DVM
Mario Quijada, MPH
Global Health Cluster Group
Study participants

Summer Research Program
The Pritzker School of Medicine
National Institute of Diabetes and Digestive and Kidney Diseases (NIDDK) Grant #T35DK062719-30
ASTMH Benjamin H. Kean Fellowship in Tropical Medicine
Thrasher Fund
INDICASAT

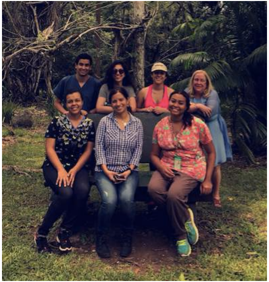

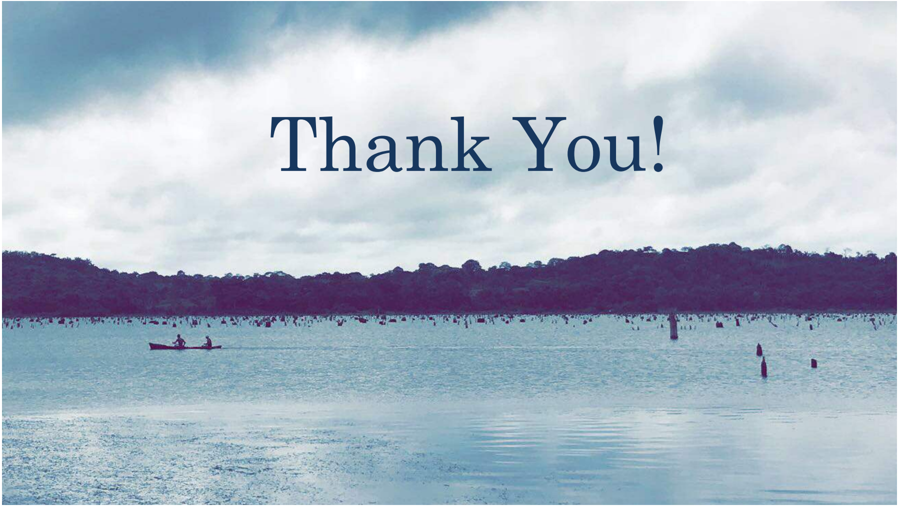
Thank You!

References

- Torgerson PR, Mastroiacovo P. The global burden of congenital toxoplasmosis: a systematic review. Bull World Health Organ. 2013; 91(7):501–8. https://doi.org/10.2471/BLT.12.111732
- Rima McLeod, Kenneth Boyer, Theodore Karrison, Kristen Kasza, Charles Swisher, Nancy Roizen, Jessica Jalbrzikowski, Jack Remington, Peter Heydemann, A. Gwendolyn Noble, Marilyn Mets, Ellen Holfels, Shawn Withers, Paul Latkany, Paul Meier, Toxoplasmosis Study Group; Outcome of Treatment for Congenital Toxoplasmosis, 1981–2004: The National Collaborative Chicago-Based, Congenital Toxoplasmosis Study, *Clinical Infectious Diseases*, Volume 42, Issue 10, 15 May 2006, Pages 1383–1394, https://doi.org/10.1086/501360
- Begeman, I., Lykins, J., Zhou, Y., Lai, B.S., Levigne, P., El Bissati, K., Boyer, K., Withers, S., Clouser, F., Noble, A.G., Rabiah, P., Swisher, C.N., Heydemann, P.T., Contopoulos-Ioannidis, D.G., Montoya, J.G., Maldonado, Y., Ramirez, R., Press, C., Stillwaggon, E., Peyron, F., McLeod, R.. (2017). Point-of-care testing for *Toxoplasma gondii* IgG/IgM using the *Toxoplasma* ICT IgG-IgM test with sera from the United States and implications for developing countries. *PLOS Neglected Tropical Diseases*. Accepted.
- Wallon M, Peyron F, Cornu C, Vinault S, Abrahamowicz M, Kopp CB, et al. Congenital toxoplasma infection: monthly prenatal screening decreases transmission rate and improves clinical outcome at age 3 years. Clin Infect Dis. 2013; 56(9):1223–31. https://doi.org/10.1093/cid/cit032 PMID: 23362291
- Prusa AR, Kasper DC, Sawers L, Walter E, Hayde M, Stillwaggon E. Congenital toxoplasmosis in Aus- tria: prenatal screening for prevention is cost-saving. PLoS Negl Trop Dis. In press, 2017.
- Hotop A, Hlobil H, Gross U. Efficacy of rapid treatment initiation following primary Toxoplasma gondii infection during pregnancy. Clin Infect Dis. 2012; 54(11):1545–52. https://doi.org/10.1093/cid/cis234 PMID: 22460980
- Kieffer F, Wallon M, Garcia P, Thulliez P, Peyron F, Franck J. Risk factors for retinochoroiditis during the first 2 years of life in infants with treated congenital toxoplasmosis. Pediatr Infect Dis J. 2008; 27 (1):27–32. https://doi.org/10.1097/INF.0b013e318134286d PMID: 18162934
- Mario S, Basevi V, Gagliotti C, Spettoli D, Gori G, D'Amico R, Magrini N. Prenatal education for congenital toxoplasmosis. Cochrane Database of Systematic Reviews2015, Issue 10. Art. No.: CD006171. DOI: 10.1002/14651858.CD006171.pub4.
- G. Pappas, N. Roussos, M.E. Falagas. Toxoplasmosis snapshots: global status of Toxoplasma gondii seroprevalence and implications for pregnancy and congenital toxoplasmosis. Int. J. Parasitol., 39 (2009), pp. 1385-1394
- Sousa OE, Saenz RE, Frenkel JK. Toxoplasmosis in Panama: A 10-year study. Am J Trop Med Hyg. 1988;38(2):315–332.
- McLeod, R., Kieffer, F., Sautter, M., Hosten, T., & Pelloux, H. (2009). Why prevent, diagnose and treat congenital toxoplasmosis? *Memorias Do Instituto Oswaldo Cruz*, *104*(2), 320–344.

Variable	N (%)
Medical School 1	
Medical Student (9th Semester)	48
Total	48 (55.17%)
Healthcare Clinic	
MD (specialty not specified)	6
MD (Internal Medicine)	5
MD (Ob/Gyn)	1
MD (Pediatrics)	1
MD (Orthopedics)	1
Total	14 (16.10%)
Public Hospital 1	
Resident (Ob/Gym PGY-3)	12
MD (Ob/Gyn)	1
Total	13 (14.94%)
Public Hospital 2	
MD (Pediatrics)	3
MD (Neonatology)	2
MD (specialty not specified)	2
MD (Internal Medicine)	1
MD (Ob/Gyn)	1
Medical Student (9th Semester)	3
Total	12 (13.79%)

Gracias por su participación. Introduzca iniciales + mes y año de nacimiento para ID único. Escoja la mejor respuesta de las siguientes afirmaciones.

Post-Survey ID
__ __ __ __ __ __

	Cierto	Falso	No Estoy Seguro
1. La toxoplasmosis puede causar enfermedad grave del cerebro en los bebés si se transmite a través de la placenta durante la gestación.	☐	☐	☐
2. La toxoplasmosis puede causar enfermedad grave en los ojos de los bebés si se transmite a través de la placenta durante la gestación.	☐	☐	☐
3. Por lo general, la toxoplasmosis produce síntomas reconocibles en adultos sanos.	☐	☐	☐
4. La toxoplasmosis no es una infección común en Panamá.	☐	☐	☐
5. La toxoplasmosis congénita no puede causar ceguera en los niños.	☐	☐	☐
6. El *Toxoplasma gondii* se puede transmitir a las personas por contacto directo con las heces de los gatos.	☐	☐	☐
7. El *Toxoplasma gondii* se puede transmitir a las personas por contacto directo con superficies u objetos que han estado en contacto con heces de gato.	☐	☐	☐
8. El *Toxoplasma gondii* se puede transmitir por el consumo de alimentos no bien lavados.	☐	☐	☐
9. El *Toxoplasma gondii* no se puede transmitir por el consumo de carnes mal cocidas.	☐	☐	☐
10. Las mujeres embarazadas y sanas no pueden contraer *Toxoplasma gondii* durante la gestación.	☐	☐	☐
11. Las mujeres pueden contraer toxoplasmosis en cualquier momento del periodo gestacional.	☐	☐	☐
12. Si una mujer embarazada no presenta los síntomas de toxoplasmosis, no puede transmitir la infección a su feto.	☐	☐	☐
13. La infección primaria aguda de toxoplasmosis puede ser diagnosticada con pruebas serológicas durante la gestación.	☐	☐	☐
14. No se puede utilizar la amniocentesis para diagnosticar toxoplasmosis congénita en el feto.	☐	☐	☐
15. Se puede utilizar el ultrasonido para ayudar a diagnosticar signos de toxoplasmosis congénita en el feto.	☐	☐	☐
16. Si una mujer embarazada contrae toxoplasmosis antes de la concepción y tiene una respuesta inmune normal, hay un bajo riesgo de infección fetal.	☐	☐	☐
17. Si una mujer embarazada contrae toxoplasmosis durante el segundo y tercer trimestre, hay un bajo riesgo de infección fetal.	☐	☐	☐
18. Espiramicina se puede administrar a mujeres embarazadas seropositivas, en la fase temprana de la gestación, para ayudar a bloquear la transmisión del *Toxoplasma gondii* al feto.	☐	☐	☐
19. La detección y el tratamiento precoz de toxoplasmosis aguda en mujeres embarazadas puede producir mejores resultados en la salud del feto.	☐	☐	☐
20. Si las madres con toxoplasmosis aguda se tratan con pirimetamina, sulfadiazina, y ácido folínico en el segundo y tercer trimestre, se puede prevenir enfermedades neurológicas graves en los recién nacidos.	☐	☐	☐
21. Si las madres con toxoplasmosis aguda se tratan con pirimetamina, sulfadiazina, y ácido folínico en el segundo y tercer trimestre, se puede prevenir el daño a los ojos en etapas futuras de la vida de los niños.	☐	☐	☐

	Muy de Acuerdo	De Acuerdo	No Estoy Seguro	No Estoy de Acuerdo	Muy en Desacuerdo
26. No se puede hacer nada para ayudar a los recién nacidos con toxoplasmosis congénita.	☐	☐	☐	☐	☐
27. La toxoplasmosis congénita es prevenible.	☐	☐	☐	☐	☐
28. Las pacientes están bien informadas sobre la toxoplasmosis y el riesgo que implica en las mujeres embarazadas.	☐	☐	☐	☐	☐
29. Es importante realizar exámenes para detectar la toxoplasmosis en las mujeres embarazadas.	☐	☐	☐	☐	☐
30. Las pruebas de toxoplasmosis para todas las mujeres embarazadas es algo posible.	☐	☐	☐	☐	☐
31. Es posible que todas las mujeres embarazadas con toxoplasmosis aguda reciban tratamiento.	☐	☐	☐	☐	☐
32. Tengo confianza en mí comprensión sobre la toxoplasmosis y el riesgo que implica en las mujeres embarazadas.	☐	☐	☐	☐	☐
33. Tengo confianza en mí comprensión acerca del diagnóstico de la toxoplasmosis aguda durante el embarazo..	☐	☐	☐	☐	☐
34. Tengo confianza en mí comprensión acerca del tratamiento para toxoplasmosis aguda durante el embarazo.	☐	☐	☐	☐	☐
35. Tengo conocimiento sobre los protocolos que son utilizados para realizar la prueba de toxoplasmosis en mujeres embarazadas en Panamá.	☐	☐	☐	☐	☐
36. Tengo conocimiento sobre cómo hacer el reporte obligatorio de los casos de toxoplasmosis congénita al MINSA.	☐	☐	☐	☐	☐
37. En mi práctica profesional, voy a sugerir a todas las pacientes embarazadas que se hagan la prueba de toxoplasmosis.	☐	☐	☐	☐	☐

Results: Feedback

THEME	REPRESENTATIVE QUOTE
Implementation of affordable and accessible test	"It would be great if the $4 test was approved and more so the case, if the test could provide individual IgG and IgM results" (MD).
Involvement of MINSA or the government	"The tests for toxoplasmosis in Panama is MINSA's responsibility. Inform the department of epidemiology with studies like yours to create awareness and concern" (Pediatrician).
	"After completing your study, it is very important to send to MINSA your results. Extend this study to other hospitals to have a better understanding of the situation here in Panama" (Neonatologist).
Patient education	"To make toxoplasmosis screening routine a source of education for healthcare providers and the public is needed being that this country doesn't have a preventative mindset. It is necessary to transform the non-medical culture that we live in…" (Medical Student).
	"There is a need to educate pregnant women about the importance of pre-natal care and place greater emphasis in regions that don't have easy access to health care centers (Medical Student).

THE UNIVERSITY OF CHICAGO

Gestational Toxoplasmosis

By: Catherine Castro
Pritzker School of Medicine

Mentor: Rima McLeod, M.D., F.A.C.P., F.I.D.S.A.

Supported by :
Pritzker School of Medicine

What is Toxoplasmosis?

- Toxoplasmosis is an infection caused by the microscopic parasite: ***Toxoplasma gondii***

Life Cycle

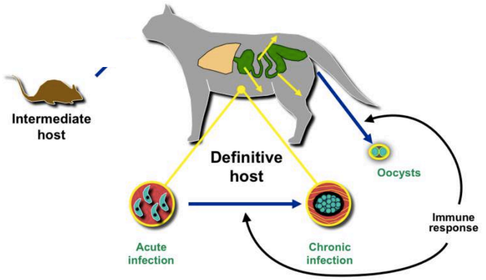

Seroprevalence of *Toxoplasma gondii*

- *T. gondii* is present in approximately **1/3** of the world population

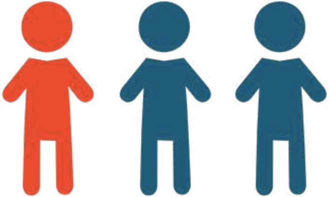

- Approximately **2 billion** people are seropositive for *T. gondii*

Seroprevalence of *Toxoplasma gondii*

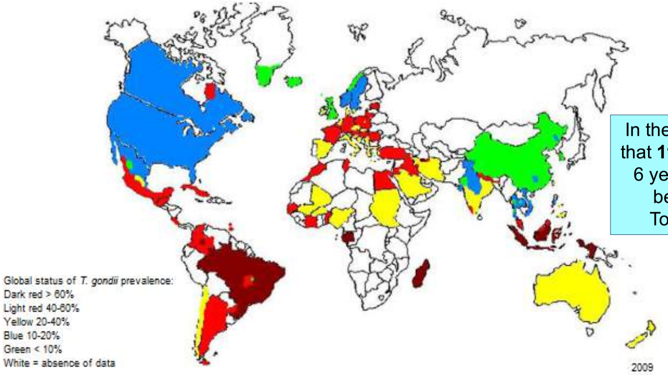

In the U.S. it is estimated that **11%** of the population 6 years and older have been infected with Toxoplasma (CDC)

Olindo Assis Martins Filho, Jordana Grazziela Alves Coelho dos Reis
UFMG Congenital Toxoplasmosis Brazilian Group *Centro de Pesquisas René Rachou - FIOCRUZ-MINAS*

Why is toxoplasmosis not commonly discussed despite its significant prevalence worldwide?

The majority of immunocompetent adults with toxoplasmosis **do not** present any recognizable symptoms.

How is *T. gondii* transmitted?

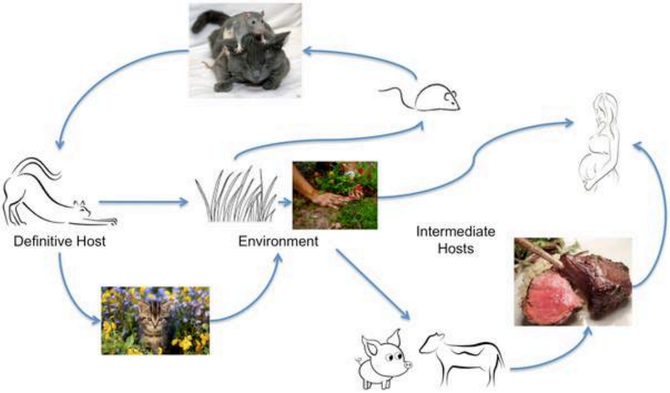

Why is screening pregnant women important?

- Healthy pregnant women can acquire toxoplasmosis at any point during gestation.
- The majority of immunocompetent adults do not have any recognizable symptoms.
- A pregnant woman can transmit the infection to her fetus even if she doesn’t present any toxo symptoms.

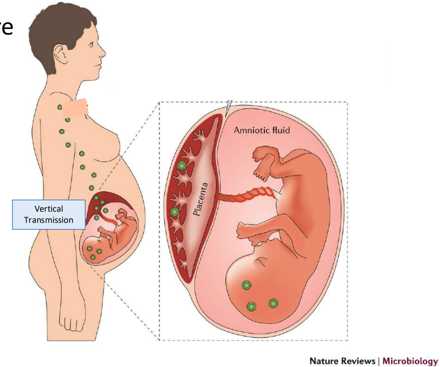

Why is screening pregnant women important?

- Toxoplasmosis can result in congenital disease when women are infected for the first time during pregnancy.

- Risk of fetal transmission and congenital disease also exists in women who acquire toxoplasmosis in the months immediately preceding conception (~6 months or less).

- Vertical transmission can result in severe or fatal symptoms in the fetus or post-partum.

- In the majority of cases, symptoms arise in the first years or future stages of life.

Congenital Toxoplasmosis Symptoms

Mild/Moderate

- Intrauterine growth restriction
- Low birth weight
- Fever
- Jaundice
- Rash
- Anemia
- Neutropenia
- Thrombocytopenia
- Pneumonia
- Hepatosplenomegaly
- Aseptic meningitis cells in CSF
- Peripheral retinal scars

Severe Congenital Toxoplasmosis Symptoms

- Cerebral calcifications
- Hydrocephalus
- Chorioretinitis
- Blindness

- Cognitive impairment
- Motor impairment
- Auditory loss
- Microcephaly
- Convulsions

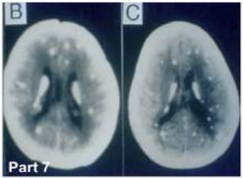

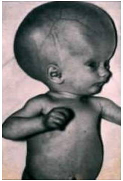

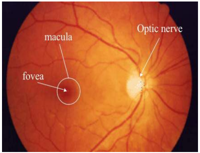

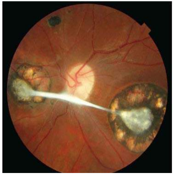

Probability of congenital infection (—) and severity of clinical signs (—) According to gestational age at maternal seroconversion

- If toxoplasmosis is acquired prior to conception, there is a low risk of fetal infection
- If toxoplasmosis is acquired in the second and third trimester, there is a high risk of fetal infection
- Many children are asymptomatic at birth but develop symptoms in the future

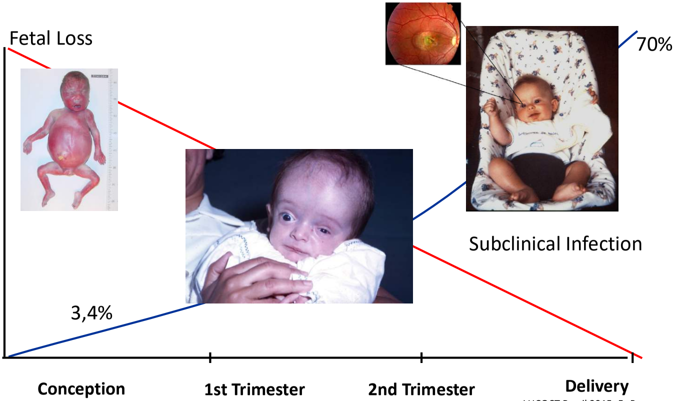

V ICOCT Brazil 2015- F. Peyron

Why is screening pregnant women important?

- Congenital toxoplasmosis is preventable and treatable.

Gestational Toxoplasmosis Screening and Diagnosis

- Serological tests

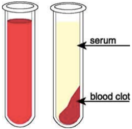

- Amniocentesis and PCR

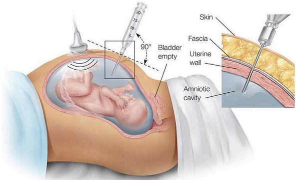

- Ultrasounds

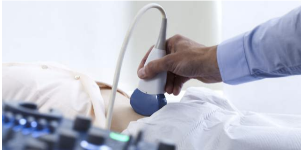

Serological tests: IgG and IgM

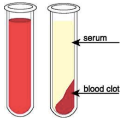

IgG-
- No prior infection with the parasite
- Treatment not needed

IgG +
- Indicates prior/an older infection (**usually 6 months or more prior**)
- Null probability of congenital infection
- **Treatment not needed**

IgM –
- Indicates the patient has not been recently infected
- Treatment not needed

IgM +
- Indicates it is possible to have a recent infection or that the patient was infected for the first time during her pregnancy: **acute toxoplasmosis**
- There is a major risk of fetal transmission and congenital disease is probable
- In this case, it is important to utilize a **toxo avidity test** to help confirm the diagnosis

It is important that pregnant women with acute toxopasmosis (IgM+) receive early treatment to reduce fetal transmission risk and the probability of congenital disease.

Treatment for Acute Toxoplasmosis (IgM+)

Diagnosis at < 14-18 weeks of gestation:

Normal Ultrasound

- Spiramycin

- Spiramycin administered to seropositive women early in gestation helps to block transmission of *T. gondii* to the fetus.

- Ultrasounds every 2 weeks

- Amniocentesis y PCR between 16-18 weeks to determine if the fetus is infected

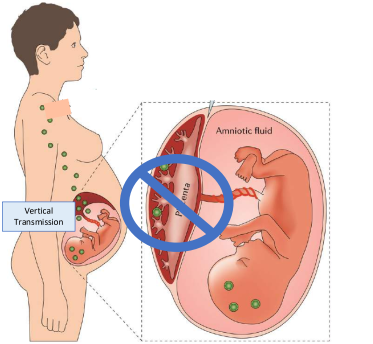

Treatment for Acute Toxoplasmosis (IgM+)

Diagnosis at < 14-18 weeks of gestation:

Abnormal Ultrasound

- Amniocentesis y PCR to determine if the fetus is infected
- For first semester -Sulfadiazine
- At ~14 weeks add: -Sulfadiazine

  -Pyrimethamine

  -Folinic acid(Leucovorin)

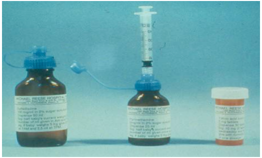

- Ultrasounds every 2 week

Treatment for Acute Toxoplasmosis (IgM+)

Diagnosis at >18 weeks of gestation:

- Amniocentesis y PCR to determine if the fetus is infected

- If the fetus is infected or fetal infection is probable (after 21 weeks), the patient should be treated with:

  -Sulfadiazine

  -Pyrimethamine

  -Folinic Acid (Leucovorin)

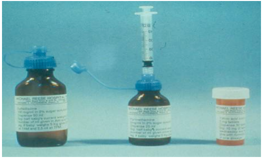

- Ultrasounds every 2 weeks

Treatment for Congenital Toxoplasmosis

<u>Diagnosis in utero or post-partum</u>:

- **For the first year of life-**
  - Sulfadiazine
  - Pyrimethamine
  - Folinic Acid (Leucovorin)

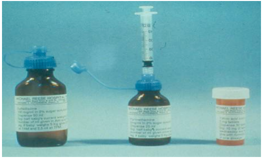

Improved Health Outcomes

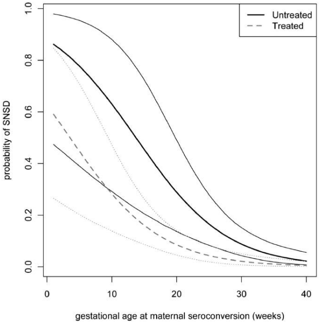

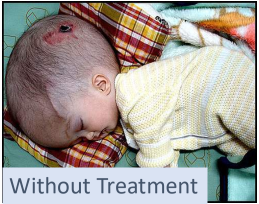

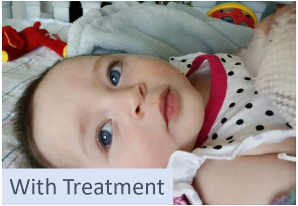

Early detection and treatment significantly reduces the risk of congenital abnormalities, severe neurological disease, and ocular damage at birth and in future life stages.

Toxoplasmosis Prevention

- Limit contact with cats and cat feces
- Limit contact with stray animals
- Use gloves and wash hands and nails well when handling materials that could potentially be contaminated with cat feces (sand, dirt, gardening tools)
- Avoid changing or handling litter boxes
- Do not drink water that could potentially be contaminated
- Do not eat undercooked or raw meats, eggs, or mussels
- Do not consume non-pateurized milk and cheeses
- Wash fruits and vegetables before consumption
- Wash surfaces, hands, and utensils after contact with meat or raw vegetables
- Obtain pre-natal health care

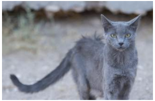
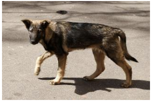
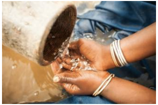
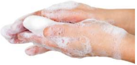
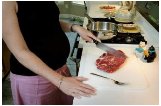
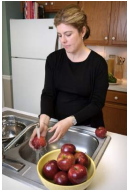

The Future

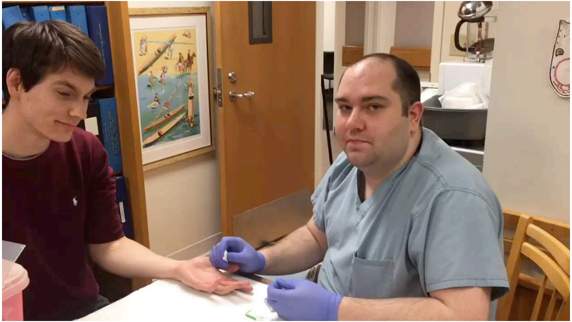

LDBIO Point-of-Care Testing

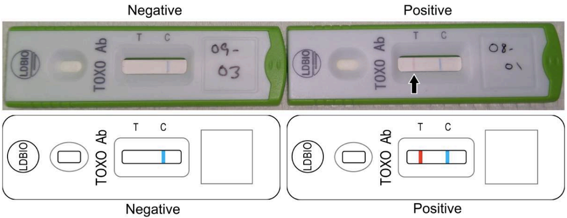

- Simple
- Precise
- Affordable ($4)
- Rapid results (20 minutes)

Screening Paradigm

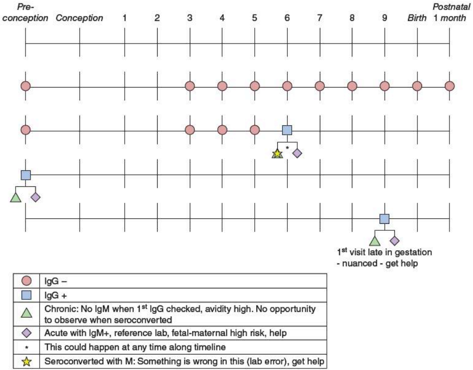

Conclusion

- Toxoplasmosis is a very common parasite and congenital infection presents a significant risk to pregnant women and their babies.
- Toxoplasmosis can cause severe nerological and ocular harm to fetuses, newborns, and children if transmitted vertically during gestation.
- Screening tests are simple and can help to prevent severe clinical outcomes.
- Early treatment of acutely infected mothers (IgM+) and of newborns with clinical manifestations can considerably improve health outcomes.
- Congenital toxoplasmosis is **preventable** and **treatable**.

Thank you!

Questions?

How can routine screening be accomplished?

Opinions and suggestions?

REFERENCES

- McLeod, R., Kieffer, F., Sautter, M., Hosten, T., & Pelloux, H. (2009). Why prevent, diagnose and treat congenital toxoplasmosis? *Memorias Do Instituto Oswaldo Cruz*, *104*(2), 320–344.
- Sousa OE, Saenz RE, Frenkel JK. Toxoplasmosis in Panama: A 10-year study. Am J Trop Med Hyg. 1988;38(2):315–332.
- Lykins, Joseph, Kanix Wang, Kelsey Wheeler, Fatima Clouser, Ashtyn Dixon, Kamal El Bissati, Ying Zhou, Christopher Lyttle, Andrey Rzewski, and Rima McLeod. "Understanding Toxoplasmosis in the United States Through "Large Data" Analyses." *Clinical Infectious Diseases* (2016): ciw356.
- Begeman, I., Lykins, J., Zhou, Y., Lai, B.S., Levigne, P., El Bissati, K., Boyer, K., Withers, S., Clouser, F., Noble, A.G., Rabiah, P., Swisher, C.N., Heydemann, P.T., Contopoulos-Ioannidis, D.G., Montoya, J.G., Maldonado, Y., Ramirez, R., Press, C., Stillwaggon, E., Peyron, F., McLeod, R.. (2017). Point-of-care testing for *Toxoplasma gondii* IgG/IgM using the *Toxoplasma* ICT IgG-IgM test with sera from the United States and implications for developing countries. *PLOS Neglected Tropical Diseases*. Accepted.
- Rengifo-Herrera, C., Pile, E., García, A., Pérez, A., Pérez, D., Nguyen, F. K., … Caballero, Z. (2017). Seroprevalence of *Toxoplasma gondii* in domestic pets from metropolitan regions of Panama. *Parasite*, *24*, 9. http://doi.org/10.1051/parasite/2017009
- Etheredge, Gina D., Michael, Girma, Muehlenbein, Michael P., & Frenkel, Jacob K. (2004). The roles of cats and dogs in the transmission of Toxoplasma infection in Kuna and Embera children in eastern Panama. *Revista Panamericana de Salud Pública*, *16*(3), 176-186. https://dx.doi.org/10.1590/S1020-49892004000900004
- Frenkel JK, Hassanein KM, Hassanein RS, Brown E, Thulliez P, Quintero-Nunez R. (1995). Transmission of *Toxoplasma gondii* in Panama City, Panama: a five-year prospective cohort study of children, cats, rodents, birds, and soil. American Journal of Tropical Medicine and Hygiene
- Mimica, Francisco, Muñoz-Zanzi, Claudia, Torres, Marisa, & Padilla, Oslando. (2015). Toxoplasmosis, zoonosis parasitaria prevalente en Chile: recuento y desafíos. *Revista chilena de infectología*, *32*(5), 541-549. https://dx.doi.org/10.4067/S0716-10182015000600008
- Ellie J. C. Goldstein, Jose G. Montoya, Jack S. Remington; Management of *Toxoplasma gondii* Infection during Pregnancy. *Clin Infect Dis* 2008; 47 (4): 554-566. doi: 10.1086/590149
- McAuley, J. B. (2014). Congenital Toxoplasmosis. *Journal of the Pediatric Infectious Diseases Society*, *3*(Suppl 1), S30–S35. http://doi.org/10.1093/jpids/piu077
- Maldonado, Y. A., & Read, J. S. (2017). Diagnosis, Treatment, and Prevention of Congenital Toxoplasmosis in the United States. *Pediatrics*. https://doi.org/10.1542/peds.2016-3860
- Cortina-Borja, M., Tan, H. K., Wallon, M., Paul, M., Prusa, A., Buffolano, W., … for The European Multicentre Study on Congenital Toxoplasmosis (EMSCOT). (2010). Prenatal Treatment for Serious Neurological Sequelae of Congenital Toxoplasmosis: An Observational Prospective Cohort Study. *PLoS Medicine*, *7*(10), e1000351. http://doi.org/10.1371/journal.pmed.1000351
- Paquet, CarolineYudin, Mark H.Yudin, Mark H.Allen, Victoria M.Bouchard, CélineBoucher, MarcCaddy, SheilaCastillo, ElianaMoney, Deborah M.Murphy, Kellie E.Ogilvie, GinaPaquet, Carolinevan Schalkwyk, JulieSenikas, Vyta et al. *Journal of Obstetrics and Gynaecology Canada*, Volume 35 , Issue 1 , 78 – 79
- Da Silva, L. B., de Oliveira, R. de V. C., da Silva, M. P., Bueno, W. F., Amendoeira, M. R. R., & Neves, E. de S. (2011). Knowledge of Toxoplasmosis among Doctors and Nurses Who Provide Prenatal Care in an Endemic Region. *Infectious Diseases in Obstetrics and Gynecology*, *2011*, 750484. http://doi.org/10.1155/2011/750484
- Davis, S. M., Anderson, B. L., Schulkin, J., Jones, K., Eng, J. V., & Jones, J. (2015). Survey of obstetrician-gynecologists in the United States about toxoplasmosis: 2012 Update. *Archives of Gynecology and Obstetrics*, *291*(3), 545–555. http://doi.org/10.1007/s00404-014-3450-y
- Jones J, Lopez A, Wilson M. Congenital toxoplasmosis. Am Fam Physician (2003) 67(10):2131–8

Toxoplasmosis Immunology

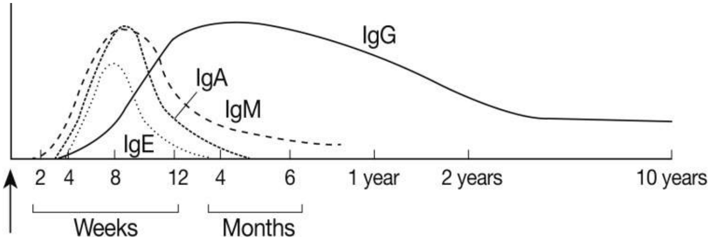

C. Test parameters of LDBIO combined *Toxoplasma* IgG/IgM POC test

Test parameter	Result	95% CI
Sensitivity*	100%	97.18-100%
Specificity**	100%	93.02-100.00%
Positive likelihood ratio	N/A (specificity 100%)	N/A (specificity 100%)
Negative likelihood ratio	0.00	0.00

* Sensitivity = TP/(TP + FN), where TP = number of true positives, FN = number of false negatives

** Specificity = TN/(TN + FP), where TN = number of true negatives, FP = number of false positives

Table 2. Economic considerations.

Test type	Cost per test (USD)	Cost per Pregnancy (10 tests) (USD)	Cost for 100 pregnancies (estimate of an obstetrical practice) (USD)	Cost savings on testing alone (USD)
Standard testing for *T. gondii* (at the University of Chicago Hospitals)	$650	$6,500[a]	$650,000	$0
Standard testing for *T. gondii* (estimate per Stillwaggon [15])	$12	$120	$12,000	$638,000
LDBIO POC test with "bookend" multiplexed testing	$4	$40	$4,000[b]	$646,000[c]
LDBIO POC test with Fingerprick on Whole Blood	$4.95[d]	$49.50	$4950	$645,050
LDBIO POC test with Saliva	$5.55[e]	$55.50	$5550	$644,450

[a] Well above standard capitation for pregnancy.

[b] Initial Investment for POC testing will also include BD Microtainer® Tubes: US$38.25 for 50 tubes, Sprout Centrifuge: US$228, Class I biosafety cabinet: US$6,546; this cost is still lower than conventional testing and substantial cost savings remains.

[c] Cost-savings could be many fold greater by multiplexing testing for HIV, syphilis, CMV, hepatitis B, herpes simplex, and potentially Zika virus or *Trypanosoma cruzi,* along with *T. gondii* IgG and IgM. This could reduce costs associated with individual testing. Additionally, monthly testing could enhance maternal-child healthcare by increasing interactions with physicians, promoting screening for pre-eclampsia and gestational diabetes.

[d] This cost includes the cost of the LDBIO test as well as the cost of an individual lancet (estimated at US$0.33) and a Sarstedt Minivette POCT collection pipette (estimated at US$0.62); collection of whole blood obviates need for initial investment for centrifuge, electricity.

[e] This cost includes the LDBIO test and the cost of a saliva swab (Beaver-Visitec- #58109, US$0.79 each) and a swab storage tube (Salimetrics- US$0.76 each)

Acute Toxoplasmosis Symptoms

- Flu-like symptoms
- Lymphadenopathy
- Muscle aches
- Fatigue
- Fever
- Headache
