# Supplementary material for: Building Programs to Eradicate Toxoplasmosis Part II: Education
Source: Curr Pediatr Rep. Author manuscript; Available in PMC 2023 Mar 23. (PMC10035399; doi:10.1007/s40124-022-00267-y)

THE UNIVERSITY OF CHICAGO
PRITZKER SCHOOL OF MEDICINE

Andrew Grose
with Catherine Castro, MD; Kevin Ashi; Rima McLeod. MD

Infectious Disease Education That Spans Borders: Bringing Panama's Toxoplasmosis Learning Tools to the United States

Andrew Grose

Advisor: Rima McLeod, MD, FACP, FIDSA

Pritzker School of Medicine Summer Research Program 2020

University of Chicago

AT THE FOREFRONT
UChicago Medicine

ABSTRACT

The value of educating both medical professionals and the public about congenital toxoplasmosis (CT) has been emphasized by researchers involved with implementing universal prenatal screening for this disease. The present study adapted an educational module that had been developed for use with medical students in a 2017 study in Panama and applied this module to a sample of U.S. students. A convenience sample of 37 students was administered a quiz on basic aspects of CT, shown a presentation that addressed this information, and given the quiz again. Average quiz scores were compared both within the U.S. sample and between the 2020 U.S. and 2017 Panama student groups. Qualitative feedback data on perceived barriers to toxoplasmosis screening were also gathered, coded, and compared to what the Panama students had said. On average, U.S. students scored significantly higher than had the Panama sample on the pre-presentation quiz (20.32 vs 18, $p = 0.000331$). Meanwhile, the students from the present study also showed a statistically significant score increase following the presentation (3.11 points, $p < 0.00001$), bringing them to a final score similar to that of the Panama group (23.43 vs 23.29, $p = 0.69$). Additionally, the educational module elicited very similar questions, comments, and ideas from both groups regarding barriers to implementing regular CT screening. Quantitative and qualitative data from this study reveal the cross-country relevance and overall effectiveness of the educational materials that were tested.

INTRODUCTION

Toxoplasma gondii is an obligate intracellular parasite with high worldwide frequency, and if acquired congenitally and not treated for promptly, the organism can have devastating and lifelong cognitive, motor, and visual sequelae – as well as severe economic and social costs – for those infected (4,10,12,13,15,16,17,19,20,25,34,36). While global estimates for incidence of congenital toxoplasmosis (CT) have been established at about 190,100 cases a year (33), the true rate in the United States is unknown (16). This disease is considered a "neglected parasitic infection" (6) in the U.S., in part because the few existing U.S. studies on CT have suggested an incidence as low as 0.5/10,000 live births/year (27). However, an issue in the United States is that there is no current systematic screening of pregnant women for *Toxoplasma*. This makes it difficult both to establish true incidence or prevalence rates and to minimize morbidity and mortality of CT. Nevertheless, arguments have been made in favor of implementing prenatal CT screening programs in the United States and worldwide (1,3,8,16,21). Such initiatives have proven very successful in reducing overall disease burden in countries such as France and Austria (28,32,35), but a similar program has still not been incorporated into U.S. healthcare protocols.

Reasons for a lack of screening in the U.S. – and in other countries without these protocols – have been widely analyzed and debated in literature and policy. They include cost-effectiveness considerations (1,2,12,13,17,22,24,28,31), lack of consistent patient access to public care facilities (24), and even a general lack of awareness regarding the disease (14,19,29). In addition, until recently there were no randomized studies of gestational treatment efficacy (18,26). Of all of these factors, previous health education research strongly suggests that improving the knowledge and awareness of pregnant women, obstetricians, and medical students is key to building high-quality,

universal CT screening programs from scratch. Recent studies in Panama and Colombia, which examine techniques for teaching medical providers and pregnant women about CT, indicate relationships between awareness of a disease and factors such as a physician's adherence to screening protocols, a patient's willingness to follow protocols, and broader societal attitudes toward addressing CT (5,7,20,23,29). More thoroughly informing both at-risk populations and medical communities thus may be a crucial component of comprehensive efforts to address CT in the United States. However, little research has been conducted regarding the background knowledge of either patients or medical professionals regarding this disease in the U.S. context.

This study focuses on the education of U.S. medical students regarding CT; it examines and seeks to improve background knowledge of students from a single institution in the United States. Methods are adapted from a similar 2017 study conducted by Dr. Catherine Castro, in association with multiple medical centers in Ciudad de Panamá, Panama (5). This project hypothesizes that knowledge of congenital toxoplasmosis among U.S. medical students, as characterized and measured by surveys adapted from Castro: (A) will initially be significantly lower than that of a sample of medical students from Panama, due to the lower CT disease burden in the U.S. (33); and (B) will significantly increase, following an academic presentation similar to that used by Castro.

METHODS

Educational intervention and survey development

The educational material and surveys used in this study were based on materials Dr. Catherine Castro had developed in collaboration with Dr. Rima McLeod, Director of the Toxoplasmosis Center at the University of Chicago, and several officials from Panama

in 2017. In-country researchers and participants came from: the Instituto de Investigaciones Científicas y Servicios de Alta Tecnología de Panamá (INDICASAT); two large urban hospitals, Hospital Santo Tomás and Hospital San Miguel Arcángel; and the Universidad Latina de Panamá.

Recruitment

While Castro's study was conducted in an in-person, large group setting, the present study was adapted to self-quarantine orders given by federal and state officials in light of SARS-CoV-2, which the World Health Organization declared a global pandemic in mid-March of 2020. From June to July 2020, rising second-year medical students at University of Chicago's Pritzker School of Medicine were asked to participate in this study via electronic mail and online communication platform Slack.

Intervention

Participants were presented with an educational video 14 minutes in length; this was an adapted version of the presentation Castro had used in her Panama study. The presentation addressed: (A) *Toxoplasma* biology and modes of transmission; (B) congenital toxoplasmosis symptoms; (C) CT screening, diagnosis, and treatment; (D) benefits of gestational screening and of early treatment of an infected fetus; (E) current U.S. policies regarding CT screening, reporting, and treatment; (F) CT reporting protocols outside of the U.S.; and (G) the *Toxoplasma* ICT IgG-IgM diagnostic point-of-care test, which is currently undergoing trials for regular use in the United States and worldwide (9,15).

Data Collection

A survey gauging knowledge of congenital toxoplasmosis was administered to participants both before and after the educational presentation, via online platform Google Forms (Appendix A). Each participant was asked to provide consent to participate and to provide a code in order to link both surveys.

Both surveys included the same twenty-five true or false questions that assessed knowledge related to CT. All tested items were addressed in the educational video, which was created by adding narrations and timed transitions to a PowerPoint presentation that had been developed for use in Panama.

At the conclusion of the post-intervention survey, participants were prompted to write about barriers they believed patients and physicians in the United States might face in accessing and providing toxoplasmosis screening.

Duration of Study

Students were given a three-week period to participate in this study.

Data Analysis

Pre- and post-intervention questionnaire scores were coded and entered into an Excel spreadsheet. Quantitative data were analyzed in STATA to evaluate changes from baseline and intervention efficacy. Statistical tests used to evaluate U.S. data and to compare with data from the previous study in Panama included McNemar's test, paired t-tests, and unpaired t-tests. Statistical significance was set at $p < 0.05$.

Qualitative data from the U.S. study were coded independently by the investigator and two peer students. Themes that each reader gleaned from the students' comments were compared, and categories the readers found in common were then used

to group data. The most common themes from the U.S. data were then compared with themes Castro had gathered from the Panama student group in 2017.

IRB Approval/Timeline

This study was conducted in accordance with an IRB exempt protocol approval, granted on June 2, 2020 (IRB20-0922). In order to satisfy Pritzker School of Medicine requirements, data were gathered with approval from the medical school's Educational Survey Request process, granted on June 15, 2020. Data collection began on June 16, 2020 and ended on July 7, 2020.

RESULTS

Participants (U.S.)

Educational materials were made available online to 93 second-year medical students between June and July 2020. A total of 37 students (40% participation rate) completed all components of the study.

Effectiveness of the teaching tool (U.S.)

Comparative analysis of the U.S. survey scores (N=37) showed a significant increase in quiz scores after the educational presentation, from an average score of 81.30% to 93.73%, or an average score increase of 3.11 points ($p < 0.00001$; see also Figure 1). The overall score distribution experienced an overall positive post-intervention shift in proportions of respondents with a given quiz score out of twenty-five (Figure 1). Meanwhile, correct response rate increased for 21 individual questions; nine showed statistically significant increases (Appendix B). The three largest increases (and lowest

initial scores) were observed in the following three questions, all related to diagnosis and treatment of congenital toxoplasmosis:

(18) Spiramycin can be given to seropositive pregnant woman early in gestation to help block transmission of *Toxoplasma gondii* to the fetus. (true)

(22) An IgG+ serological result indicates recent *Toxoplasma* infection. (false)

(24) A pregnant woman who acquired toxoplasmosis 6 months or more prior to conception should receive treatment to help prevent CT. (false)

Comparison with Panamanian 9th-semester medical students

As an attempt to compare the effectiveness of this educational intervention between the U.S. and Panama, data from this study were compared with similar data collected in a 2017 study that used very similar questionnaires and an identical presentation with 51 ninth-semester Panamanian medical students (Figure 1). In pre-presentation surveys, the Panamanian group had significantly lower mean scores (18 compared to 20.32, $p = 0.000331$). However, both groups showed statistically significant increases in average score following the presentation (U.S. = 3.11 points, $p < 0.00001$; Panama = 5.29 points, $p < 0.00001$). Additionally, average post-presentation scores between groups did not show a statistically significant difference (23.29 compared to 23.43, $p = 0.69$).

Figure 1. Distributions of Panama and U.S. questionnaire scores preceding and following educational intervention. While the Panamanian group had significantly lower average scores (as determined using an unpaired t-test), both groups of students showed statistically significant increases in average score following the presentation (as determined by paired t-tests), while average post-test scores between groups (unpaired t-test) were not significantly different.

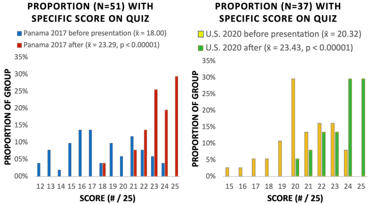

Qualitative analysis (U.S.)

Nineteen of the 37 U.S. respondents provided feedback on the free-response prompt: “What are some barriers you believe patients and physicians face in accessing/providing a toxo screening program in the United States? How might we go about implementing routine screening?” As the investigator’s initial qualitative analysis shows, some of the most commonly mentioned topics included: cost-effectiveness of a universal screening program, patient and physician awareness of toxoplasmosis and its effects, an unequally distributed disease burden (both internationally and in-country), restrictions associated with health systems and access to care, and the typical clinical protocols associated with pregnant women’s appointments (Table 1).

Table 1. Most commonly mentioned topics regarding barriers and possible solutions for implementing national screening programs for CT in the United States. Data here reflect a preliminary qualitative analysis done only by the investigator.

Theme	Selected quotes (edited for clarity)
**Cost-effectiveness of a national screening program**	“[I]n the USA [toxo screening] would probably be less cost effective, with the risks of doing invasive procedures like amniocentesis outweighing a reduction in morbidity/mortality due to congenital toxo” (Participant 4); “[H]ow expensive is the treatment? A cheap test with expensive treatment would leave us in the same predicament with more knowledge, [i.e.] we’d have a toxo diagnosis but still no funds to treat it” (P8); “Looking at base rate of congenital toxo in the U.S., as well as

	sensitivity and specificity of tests, routine screening simply may not be a cost effective program to implement in the U.S." (P29)
**Patient and physician awareness of toxoplasmosis and its effects**	"[P]atients may not be aware that they need screening for this (require more patient education and awareness about toxo)" (P13); "Educating women about what the disease is" (P15); "Assuring pregnant people that screening will improve health outcomes for their child" (P25)
**Unequal disease burden**	"[H]ow is the burden distributed globally (developed vs less developed countries)" (P3); "Neglected disease that affects underserved communities therefore less interest" (P37)
**Health systems and access to prenatal care**	"Community-based toxo screenings leveraging already-trusted community assets (churches, food banks) could be a good place to start" (P8); "Patients may have a difficult time getting to appointments, finding childcare, affording co-payments, etc." (P14); "In general, maternal care is underfunded and underemphasized in society" (P17); "Limitations in insurance coverage" (P27); "[L]ack of transportation to screening centers/opportunities; lack of healthcare insurance and/or prohibitive cost to patients" (P34)
**Protocols for clinical visits**	"Potential solution: Baking screening into protocols/EMR" (P3); "Time in visit" (P16); "Integrating routine screening into prenatal care visits" (P25)

Once analysis had been completed by all three readers and a final list of themes and representative quotes had been obtained, comparison between the free responses from this study and the 2017 research in Panama showed that both student groups had commented on similar themes. The three most common themes in the U.S. were nearly identical to the three most common themes in Panama, as determined by Castro's study: cost-effectiveness of screening and treatment protocols, health systems and access to screening care, and public awareness regarding congenital toxoplasmosis (Table 2).

Table 2. Comparison of most commonly mentioned themes between Panama and U.S. medical student groups. Data in the U.S. section reflect a shortened form of the final qualitative analysis done by all three readers. The results of Castro's qualitative analysis were compared with those of this study, and many similarities were found between both groups' feedback. The most common themes mentioned included cost, health systems and access, and education and overall awareness regarding congenital toxoplasmosis.

THEME	EXAMPLE (Panama 2017)	EXAMPLE (U.S. 2020)
COST	"It would be great if the *$4 [point of care] test* was approved..."	"[H]ow *expensive is the treatment* [for a single diagnosed congenital toxoplasmosis case]?"
HEALTH SYSTEMS & ACCESS	"...this country *doesn't have a preventative mindset*."	"In general, *maternal care is underfunded and underemphasized in society*."
AWARENESS	"There is a need to educate pregnant women about *the importance of prenatal care*..."	"*Assuring pregnant people* that screening will improve health outcomes for their child"

DISCUSSION

As measured by metrics already used in a study on Panamanian congenital toxoplasmosis education, background knowledge of the U.S. medical students was already relatively high. Scores on the pre-presentation survey were significantly greater

than those of a sample of students from Panama, a country with higher CT disease burden than the U.S. However, the learning materials from Panama still taught students in this study about key topics related to CT; this was demonstrated by the significant increase in mean survey score. Additionally, the presentation brought both groups of students to a similar final average score. Finally, with respect to the free responses gathered in this study, the presentation elicited similar questions from Panamanian and U.S. medical students regarding the challenges of implementing regular prenatal screening for CT.

When it comes to the effectiveness of Panama's teaching tools in the U.S., the second and third findings mentioned above support that the presentation used in this study is a versatile tool that can work equally well among students from both countries. With respect to the barriers to prenatal screening in these countries, the fourth finding supports that many issues underlying the lack of universal screening for CT are fundamentally the same between Panama and the United States.

As this study is the first of its kind, it is difficult to say whether these interpretations are consistent with the findings of other studies about international educational efforts regarding toxoplasmosis. However, with respect to Castro's study, my results validate the utility of her presentation and surveys outside of Panama, and they invite further use of these materials in more rigorous educational studies in the U.S. Furthermore, the investigation suggests that both countries could benefit each other by collaborating on educational campaigns regarding congenital toxoplasmosis. The similarity of feedback, concerns, and ideas between Panama and the U.S. underlines the importance of sharing teaching resources on health issues among countries with similar gaps in healthcare.

In order to further evaluate the utility of the educational materials used in this study, methods should be fit to a more rigorous study design. While this investigation was quickly adapted to a setting that prohibited in-person communication and reduced intended population size, there were nevertheless issues that could have been improved upon. In order to support or challenge my claims regarding "U.S. medical students," a study similar to this one would need to be replicated with larger samples of students, people at different levels of training, and different medical schools. Additionally, to ensure that scores more accurately reflect that of a general student population, it would be ideal if participants were randomly selected. While self-selection was needed to maximize participation rate, this may have skewed initial scores toward higher values, since students who took part likely had an earlier interest in and greater knowledge of CT.

With all of this said, results indicate the success of bringing teaching materials developed for use in Panama to a group of medical students in the United States.

CONCLUSION

Implementing regular screening to reduce congenital toxoplasmosis incidence within any country is an effort that depends on many factors besides the immediate threat that *Toxoplasma* presents to a fetus (23,27). While cost-effectiveness, access to care, and disparities in disease burden are all unique challenges related to such healthcare protocols, this study explored the knowledge and beliefs of medical students in the Panamanian and U.S. healthcare systems.

This study demonstrated that a medical education module on congenital toxoplasmosis effectively improved knowledge of a study cohort of U.S. medical students. Moreover, the improvement here was similar to that seen in a study that used

the same learning materials with medical students in Panama. This suggests that the educational interventions used in this study can – and should – be expanded to new student populations within both countries. As we imagine an informed pregnant woman would take the preventative steps she could to avoid acquiring *Toxoplasma*, so we hope an informed medical community would educate their patients about congenital toxoplasmosis and strongly support novel CT screening and treatment protocols.

Additionally, this study showed the value of an assumption that has guided CT screening initiatives in Panama: when it comes to building robust medical infrastructure to address a relatively rare disease, participants' ideas for the future are just as valuable as what researchers have to offer. As such, this study both taught participants about CT and gathered feedback that could help us identify key targets for future steps to implementing care strategies. As students in both Panama and the U.S. have indicated – and as we believe – one of these priority items is raising patient and physician awareness about congenital toxoplasmosis and its burden on society.

ACKNOWLEDGEMENTS

Special thanks to Dr. Rima McLeod, director of the Toxoplasmosis Center at the University of Chicago, for her guidance with study design, educational interventions, survey development, and all adaptations made during the SARS-CoV-2 pandemic. Thanks to Dr. Catherine Castro, Pritzker School of Medicine Class of 2020, for assisting with data analysis and for sharing her perspectives on the study she conducted in Panama in 2017. Thanks to two student small groups of which I was a part – the University of Chicago Center for Global Health's summer seminar group and the Summer Research Program Cancer Research small group – for providing important feedback throughout the research process. Special thanks to Kevin Ashi, Pritzker Class

of 2023, for introducing me to the field of toxoplasmosis research and for assisting me with my literature review. Finally, thanks to the Pritzker School of Medicine for their financial and logistical support of this research.

APPENDIX A: QUESTIONNAIRE

Question Number	T/F Statement	Answer
1	Toxoplasmosis can cause severe brain disease to babies if transmitted across the placenta during gestation.	T
2	Toxoplasmosis can cause severe eye disease to babies if transmitted across the placenta during gestation.	T
3	In general, toxoplasmosis produces recognizable symptoms in healthy adults.	F
4	Toxoplasmosis is not a common infection in Panama.	F
5	Toxoplasmosis cannot cause blindness in children.	T
6	*Toxoplasma gondii* can be transmitted to humans via direct contact with cat feces.	T
7	*Toxoplasma gondii* can be transmitted to humans via direct contact with surfaces or objects that have come in contact with cat feces.	T
8	*Toxoplasma gondii* can be transmitted via consumption of poorly washed foods.	T
9	*Toxoplasma gondii* cannot be transmitted via the consumption of poorly cooked foods.	F
10	Healthy pregnant women cannot acquire toxoplasmosis during gestation.	F
11	A woman can acquire toxoplasmosis at any point during gestation.	T
12	If a pregnant woman does not present with symptoms of toxoplasmosis, she cannot transmit the infection to her fetus.	F
13	Acute primary toxoplasmosis infection can be diagnosed with serologic tests during gestation.	T
14	Amniocentesis cannot be used to diagnose congenital toxoplasmosis in the fetus.	F
15	Ultrasounds can be used to help diagnose fetal signs of congenital toxoplasmosis.	T
16	If a pregnant woman acquired toxoplasmosis before conception and she has a normal immune response, there is a low risk of fetal infection.	T
17	If a pregnant woman acquires toxoplasmosis in the second or third and she has a normal immune response, there is a low risk of fetal infection.	F
18	Spiramycin can be given to seropositive pregnant woman early in gestation to help block transmission of *Toxoplasma gondii* to the fetus.	T
19	Early diagnosis and treatment of acute toxoplasmosis in pregnant woman improves fetal health outcomes.	T
20	Severe neurological disease can be prevented in newborns if mothers with acute toxoplasmosis are treated with pyrimethamine, sulfadiazine, and folinic acid in the second and third trimester.	T
21	Future ocular damage in children can prevented if mothers with acute toxoplasmosis are treated with pyrimethamine, sulfadiazine, and folinic acid in the second and third trimester.	T
22	An IgG positive serological result indicates recent toxoplasmosis infection.	F
23	An IgM positive serological results can indicate recent toxoplasmosis infection.	T
24	A pregnant woman that acquired toxoplasmosis 6 months or more prior to conception should receive treatment to help prevent congenital toxoplasmosis.	F
25	A pregnant woman who acquires toxoplasmosis for the first time during gestation should receive treatment to help prevent congenital toxoplasmosis.	T

APPENDIX B: INDIVIDUAL QUESTION SCORES

Individual question scores on congenital toxoplasmosis questionnaire before and after an educational presentation on the topic, compared using McNemar's test. Statistically significant increases (all $p < 0.05$) were seen in nine out of the 25 questions, while overall increases were seen in 21 of the items. The three largest increases (and lowest initial scores) were observed in the highlighted three questions, all related to diagnosis and treatment of congenital toxoplasmosis.

Question	Category	Pre-Survey [# out of 37 who responded correctly]	Post-Survey (change)	P-Value
**1**	Symptoms	36	37 (+1)	0.32
**2**	Symptoms	36	37 (+1)	0.32
**3**	Symptoms	31	36 (+5)	0.025*
**4**	Prevalence	35	36 (+1)	0.56
**5**	Symptoms	34	30 (-4)	0.16
**6**	Transmission	36	37 (+1)	0.32
**7**	Transmission	32	37 (+5)	0.025*
**8**	Transmission	28	36 (+8)	0.0047*

**9**	Transmission	19	18 (-1)	0.059
**10**	Transmission	37	34 (-3)	0.083
**11**	Transmission	34	37 (+3)	0.083
**12**	Infection risk	36	37 (+1)	0.32
**13**	Diagnosis	35	36 (+1)	0.56
**14**	Diagnosis	26	30 (+4)	0.25
**15**	Diagnosis	26	37 (+11)	0.0009*
**16**	Infection risk	26	29 (+3)	0.37
**17**	Infection risk	32	37 (+5)	0.025*
**18**	Treatment	21	37 (+16)	<0.0001*
**19**	Treatment	36	37 (+1)	0.32
**20**	Treatment	35	37 (+2)	0.16
**21**	Treatment	35	37 (+2)	0.16
**22**	Diagnosis	21	34 (+13)	0.0008*
**23**	Diagnosis	29	37 (+8)	0.0047*
**24**	Treatment	14	30 (+16)	<0.0001*
**25**	Treatment	37	37 (0)	N/A

BIBLIOGRAPHY

(1) Aguirre AA, Longcore T, Barbieri M, Dabritz H, Hill D, Klein PN, Lepczyk, Lilly EL, McLeod R, Milcarsky J, Murphy CE, Su C, VanWormer E, Yolken, Sizemore GC (2019). The One Health Approach to Toxoplasmosis: Epidemiology, Control, and Prevention Strategies. EcoHealth 16: 378-390. doi: 10.1007/s10393-019-01405-7.

(2) Begeman IJ, Lykins J, Zhou Y, Lai BS, Levigne P, El Bissati K, Boyer, K, Withers S, Clouser F, Noble AG, Rabiah P, Swisher CN, Heydemann PT, Contopoulous-Ioannidis DG, Montoya JG, Maldonado Y, Ramirez R, Press C, Stillwaggon E, Peyron F, McLeod R (2017). Point-of-care testing for Toxoplasma gondii IgG/IgM using Toxoplasma ICT IgG-IgM test with sera from the United States and implications for developing countries. PLoS Negl Trop Dis 11(6): e0005670. doi: 10.1371/journal.pntd.0005670.

(3) Boyer K, Holfels E, Roizen N, et al. (2005). Risk factors for Toxoplasma gondii infection in mothers of infants with congenital toxoplasmosis: implications for prenatal management and screening. Am. J. Obstetrics and Gynecology 192: 564-571.

(4) Boyer K, Hill D, Mui E, Wroblewski K, Karrison T, Dubey JP, Sautter M, Noble AG, Withers S, Swisher C, Heydemann P, Hosten T, Babiarz J, Lee D, Meier P, McLeod R (2011). Unrecognized Ingestion of *Toxoplasma gondii* Oocysts Leads to Congenital Toxoplasmosis and Causes Epidemics in North America. CID 53(11): 1081-1089. doi: 10.1093/cid/cir667.

(5) Castro C (2017). Impact of gestational and congenital toxoplasmosis medical education: A pre and post-intervention study in Panama City, Panama. Unpublished. From a study conducted in Panama in 2017 through the University of Chicago Pritzker School of Medicine Summer Research Program. Supplement to (30).

(6) Centers for Disease Control and Prevention. Toxoplasmosis. Available at: http://www.cdc.gov/parasites/toxoplasmosis/.

(7) Di Mario S, Basevi V, Gagliotti C, Spettoli D, Gori G, D'Amico R, Magrini N (2013). Prenatal education for congenital toxoplasmosis. Cochrane Database of Systematic Reviews 2: CD006171. doi: 10.1002/14651858.CD006171.pub3.

(8) El Bissati J, Levigne P, Lykins J, Adlaoui EB, Barkat A, Berraho A, Laboudi M, El Mansouri B, Ibrahimi A, Rhajaoui M, Quinn F, Murugesan M, Seghrouchni F, Gómez-Marín JE, Peyron F, McLeod R (2018). Global initiative for congenital toxoplasmosis: an observational and international comparative clinical analysis. Emerging Microbes & Infections 7(1): 1-14. doi: 10.1038/s41426-018-0164-4.

(9) Gomez CA, Budvytyte LN Press C, Zhou L, McLeod R, Maldonado Y, Montoya JG, Contopoulos-Ioannidis DG (2018). Evaluation of Three Point-of-Care Tests for Detection of *Toxoplasma* Immunoglobulin IgG and IgM in the United States: Proof of Concept and Challenges. Open Forum Infectious Disease 1: 1-8. doi: 10.1093/ofid/ofy215.

(10) Gómez-Marin JE, de la Torre A, Angel-Muller E, Rubio J, Arenas J, Osorio E, et al., 2011. First Colombian multicentric newborn screening for congenital toxoplasmosis. PLoS Negl. Trop. Dis. 5 (5): e1195. doi: 10.1371/journal. pntd.0001195.

(11) Hotop A, Hlobil H, Gross U (2012). Efficacy of rapid treatment initiation following primary Toxoplasma gondii infection during pregnancy. Clin. Infect. Dis. 54(11): 1545-1552.

(12) Jones JL, Dargelas V, Roberts J, Press C, Remington JS, Montoya JG (2009). Risk Factors for *Toxoplasma gondii* Infection in the United States. Clinical Infectious Diseases 49: 878-884. doi: 10.1086/605433.

(13) Kijlstra A & Petersen E (2013). Epidemiology, Pathophysiology, and the Future of Ocular Toxoplasmosis. Ocular Immunology and Inflammation 22(2): 138-147. doi: 10.3109/09273948.2013.823214.

(14) Li X, Soberón Felín M, Bodden L, Boyer KM, McLeod R, Reyes O (2016). Toxoplasmosis Education for Pregnant Women in Panama. Obstetrics and Gynecology 127(5): 15S-16S. Supplement to (30).

(15) Lykins J, Li X, Levigne P, Zhou Y, El Bissati K, Clouser F, et al. (2018) Rapid, inexpensive, fingerstick, whole-blood, sensitive, specific, point-of-care test for anti-Toxoplasma antibodies. PLoS Negl Trop Dis 12(8): e0006536. doi: 10.1371/journal.pntd.0006536.

(16) Lykins J, Wang K, Wheeler J, Clouser F, Dixon A, El Bissati K, Zhou Y, Lyttle C, Rzhetsky A, McLeod R (2016). Understanding Toxoplasmosis in the United States Through "Large Data" Analyses. Clinical Infectious Diseases 63(4): 468-475. doi: 10.1093/cid/ciw356.

(17) Maldonado YA, Read JS, AAP Committee on Infectious Diseases. Diagnosis, Treatment, and Prevention of Congenital Toxoplasmosis in the United States. *Pediatrics*. 2017;139(2):e20163860

(18) Mandelbrot L, Kieffer F, Sitta R, Laurichesse-Delmas H, Winer N, Mesnard L, Berrebi A, Le Bouar G, Bory JP, Cordier AG, Ville Y, Perrotin F, Jouannic JM, Biquard F, d'Ercole C, Houfflin-Debarge V, Villena I, Thiébaut R (2018). Prenatal therapy with pyrimethamine + sulfadiazie vs spiramycin to reduce placental transmission of toxoplasmosiss: a multicenter, randomized trial. Am J Obstet Gynecol 219: 386.e1-9. doi: 10.1016/j.ajog.2018.05.031.

(19) McLeod R & Boyer KM; ed. Kliegman RM & St. Geme J (2020). "Toxoplasmosis (*Toxoplasma gondii*)." *Nelson Textbook of Pediatrics, 21st edition*, Elsevier Inc.

(20) McLeod R, Cohen W, Dovgin S, Finkelstein L, Boyer K; ed. Weiss L (2020). "Human *Toxoplasma* infection." *Toxoplasma Gondii*, Elsevier Inc.

(21) McLeod R, Kieffer F, Sautter M, Hosten T, Pelloux H. (2009). Why prevent, diagnose and treat congenital toxoplasmosis? Mem. Inst. Oswaldo Cruz 104: 320-344.

(22) McLeod R, Lykins J, Noble AG, Rabiah P, Swisher CN, Heydemann PT, McLone D, Frim D, Withers S, Clouser F, Boyer K (2014). Management of Congenital Toxoplasmosis. Curr Pedatr Rep 2: 166-194. doi: 10.1007/s40124-014-0055-7.

(23) Montoya JG (2018). Systematic screening and treatment of toxoplasmosis during pregnancy: is the glass half full or half empty? Am J Obstet Gynecol 219: 315-319. doi: 10.1016/j.ajog.2018.08.001.

(24) Moreira AF & Pandey A (2016). Investigating Social and Infrastructural Parameters Concerning Toxoplasmosis in Panama. Unpublished. From a study conducted in Panama in 2016 under the University of Chicago Center for Global Health Summer Research Fellowship. Supplement to 30.

(25) Petersen E, Kijlstra A, Stanford M (2012). Epidemiology of Ocular Toxoplasmosis. Ocular Immunology and Inflammation 20(2): 68-75. doi: 10.3109/09273948.2012.661115.

(26) Peyron F, L'Ollivier C, Mandelbrot L, Wallon M, Piarroux R, Kieffer F, Hadjadj E, Paris L, Garcia-Meric P (2019). Maternal and Congenital Toxoplasmosis: Diagnosis and Treatment Recommendations of a French Multidisciplinary Working Group. Pathogens 8(24): 1-15.

(27) Peyron F, McLeod R, Ajzenberg D, Contopoulos-Ioannidis D, Kieffer F, Mandelbrot L, et al. (2017) Congenital Toxoplasmosis in France and the United States: One Parasite, Two Diverging Approaches. PLoS Negl Trop Dis 11(2): e0005222. doi:10.1371/journal.pntd.0005222

(28) Prusa AR, Kasper DC, Sawers L, Walter E, Hayde M, Stillwaggon E (2017). Congenital toxoplasmosis in Austria: Prenatal screening for prevention is cost-saving. PLoS Negl Trop Dis 11(7): e0005648. doi: 10.1371/journal.pntd.0005648.

(29) Sánchez J, Raggi C, Lorena López L, Orozco González RJ, Gómez Marín, JE, McLeod R (2019). Education as a Critical Tool to Aid in the Fight Against Toxoplasmosis. Unpublished. From a study conducted in Colombia in 2019 under the University of Chicago Center for Global Health Summer Research Fellowship. Supplement to (30).

(30) Soberón-Felín M, Wang K, Caballero Z, Norero X, Estripeaut D. Ellis D, Dovgin S, Raggi C, Castro C, Moosazadeh D, Ramirez M, Pandey A, Moreira A, Grose A, Ashi K, Dixon A, Li X, Clauser FA, Begeman I, Heichman S, Lykins J, Padrieu G, Villavos D, Fabrega L, Sanchez J, Mendivil C, Quijada ML, Fernandez-Pirla S, Wong D, Guevara M, Flores C, Borace J, Garcia A, Caballero N, Renfugio C, Wroblewski K, Dhamsania V, Graves N, Kirchberg M, Mathur K, Aue A, Rebellon A, Nicolai D, Rzhetsky A, Stillwaggon E, Sawer L, Peyron F, Boyer K, Heydemann P, Britton G, Villareal A, Perez A, Vargas M, Lopez LL, Gomez-Marin J, Rao J, Llorenz XS, Reyes O, McLeod R (2020). Building Programs to Eradicate Toxoplasmosis: Use of a Public Health System's Approach Paradigm to Save Mothers' and Children's Lives, Cognition, Motor Function, and Sight in Developing and Developed Countries. In preparation.

(31) Stillwaggon E, Carrier CS, Sautter M, McLeod R (2011). Maternal Serologic Screening to Prevent Congenital Toxoplasmosis: A Decision-Analytic Economic Model. PLoS Negl Trop Dis 5(9): e1333. doi:10.1371/journal.pntd.0001333.

(32) Thulliez P (1992). Screening Programme for Congenital Toxoplasmosis in France. Scandinavian Journal of Infectious Diseases Supplement 84: 43-45.

(33) Torgerson PR & Mastroiacovo P (2013). The global burden of congenital toxoplasmosis: a systematic review. Bull World Health Organ 91: 501-508. doi: 10.2471/BLT.12.111732.

(34) Vogel N, Kirisits M, Michael E, Bach H, Hostetter M, Boyer K, Simpson R, Holfels E, Hopkins J, Mack D, Mets MB, Swisher CN, Patel D, Roizen N, Stein L, Stein M, Withers S, Mui E, Egwuagu C, Remington J, Dorfman R, McLeod R (1996). Congenital Toxoplasmosis Transmitted from an Immunologically Competent Mother Infected Before Conception. Clinical Infectious Diseases 23: 1055-1060.

(35) Wallon M, Peyron F, Cornu C, VInault S, Abrahamowicz M, Kopp CB, Binquet C (2013). Congenital *Toxoplasma* Infection: Monthly Prenatal Screening Decreases Transmission Rate and Improves Clinical Outcome at Age 3 Years. Clinical Infectious Diseases 56(9): 1223-1231. doi: 10.1093/cid/cit032.

(36) Weiss LM & Dubey JP (2009). Toxoplasmosis: a history of clinical observations. International Journal of Parasitology 39(8): 895-900.

AT THE FOREFRONT UChicago Medicine

Infectious Disease Education That Spans Borders: Bringing Panama's Toxoplasmosis Learning Tools to the United States

Andrew Grose
Pritzker School of Medicine

Rima McLeod, MD, FACP, FIDSA

Supported by Pritzker School of Medicine

Background – The organism: *Toxoplasma gondii*

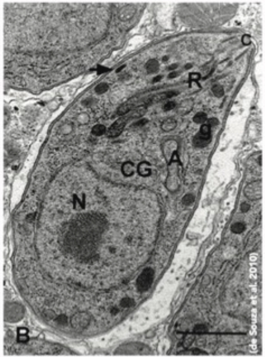

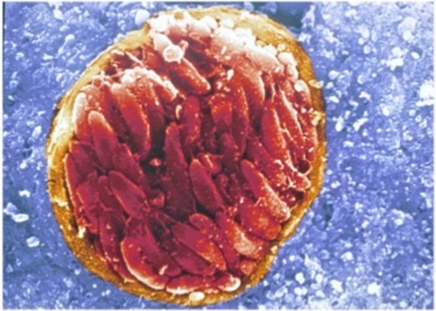

The protozoan Toxoplasma gondii, tissue cyst in brain (Photo: D. Ferguson, Oxford University)

Congenital toxoplasmosis (and the value of early detection)

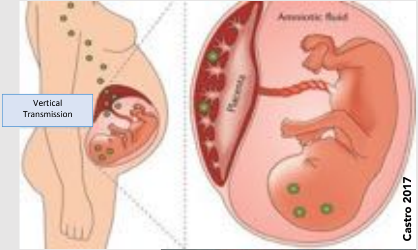

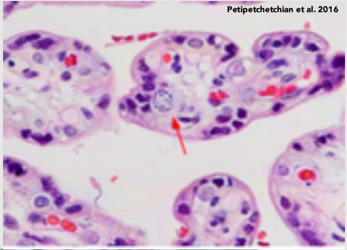

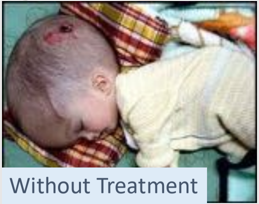

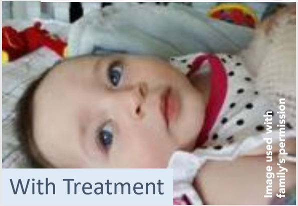

The value of education – Panama (2014-present)

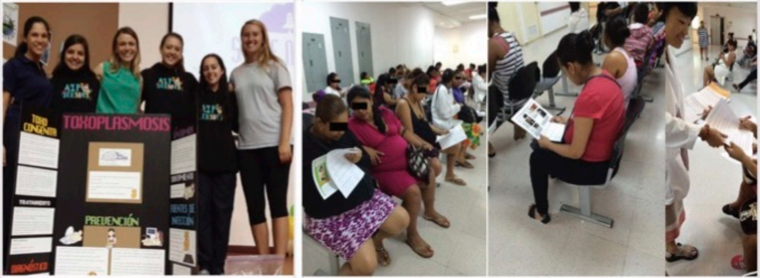

Research Question (as defined in Panama)

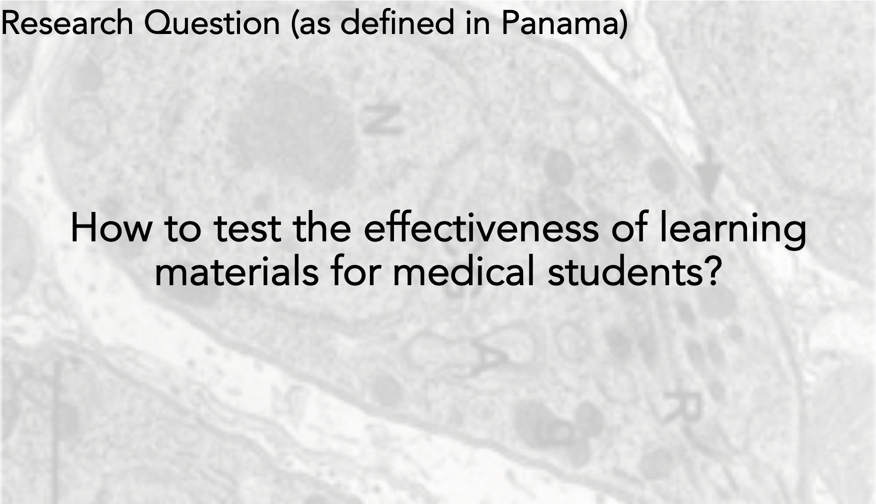

How to test the effectiveness of learning materials for medical students?

How to test the effectiveness of learning materials for medical students? (Castro 2017, Panama)

(1) Administer quiz

Does an IgM-positive result indicate recent infection?

Should a pregnant woman who acquired *Toxoplasma* 6 months or prior to conception receive treatment?

Should a pregnant woman who acquired *Toxoplasma* during gestation receive treatment?

(etc.)

How to test the effectiveness of learning materials for medical students? (Castro 2017, Panama)

(2) Give presentation

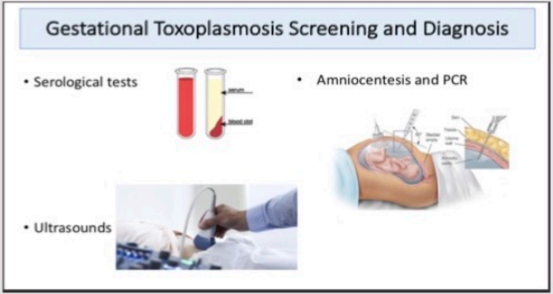

How to test the effectiveness of learning materials for medical students? (Castro 2017, Panama)

(3) Administer quiz again

Does an IgM-positive result indicate recent infection?

Should a pregnant woman who acquired *Toxoplasma* 6 months or prior to conception receive treatment?

Should a pregnant woman who acquired *Toxoplasma* during gestation receive treatment?

(etc.)

My Hypotheses – Panama (in-person) → U.S. (online)

Hypothesis A: Sample of U.S. medical students scores ***significantly lower*** than sample of students from Panama

Hypothesis B: Average U.S. score ***significantly increases*** following presentation from Panama

Methods (online)

- Convenience sample (2nd-year, one U.S. school)
- 37 respondents (40% response rate)
- T-tests compare average scores (within U.S. *and* U.S. vs Panama)

(1) Administer quiz

Does an IgM-positive result indicate recent infection?

Should a pregnant woman who acquired *Toxoplasma* 6 months or prior to conception receive treatment?

Should a pregnant woman who acquired *Toxoplasma* during gestation receive treatment?

(etc.)

Methods (online)

- Convenience sample (2nd-year, one U.S. school)
- 37 respondents (40% response rate)
- T-tests compare average scores (within U.S. *and* U.S. vs Panama)

(2) Give presentation

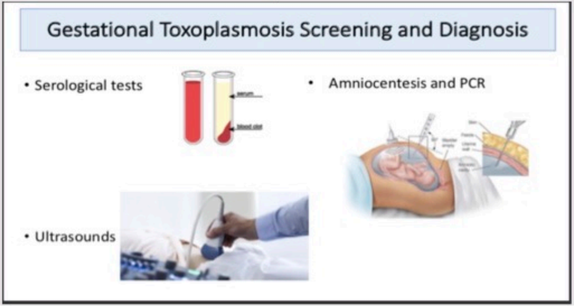

Methods (online)

- Convenience sample (2nd-year, one U.S. school)
- 37 respondents (40% response rate)
- T-tests compare average scores (within U.S. *and* U.S. vs Panama)

(3) Administer quiz again + free response

Should a pregnant woman who acquired *Toxoplasma* during gestation receive treatment?

(etc.)

What do you believe are barriers physicians and their patients would face in accessing or implementing universal prenatal toxoplasmosis screening?

Results: Scores (quantitative)

(a) U.S. score before presentation ***significantly higher*** than that of Panama ($p = 0.0003$)

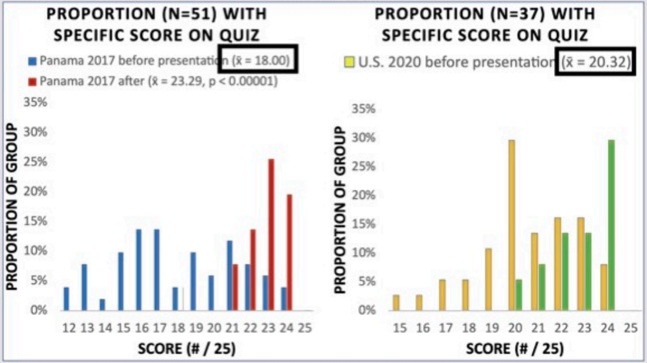

Results: Scores (quantitative)

(b) ***Significant increase*** in U.S. score following presentation

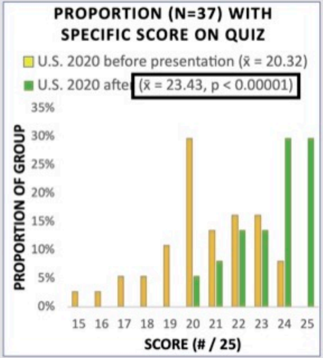

Results: Scores (quantitative)

(c) Post-presentation scores ***not significantly different*** between Panama and U.S. ($p = 0.69$)

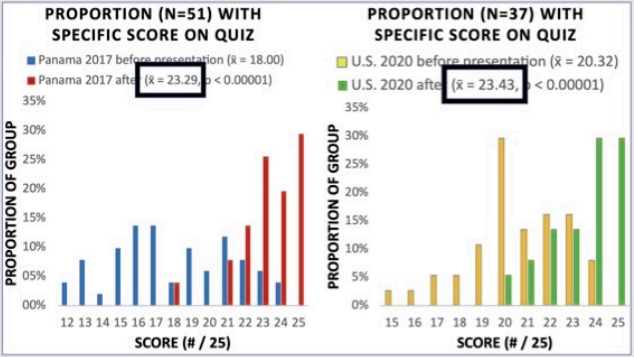

Results: Perceived barriers (qualitative)

THEME	EXAMPLE (Panama 2017)	EXAMPLE (U.S. 2020)
COST	"It would be great if the *$4 [point of care] test* was approved…"	"[H]ow *expensive is the treatment?*"
HEALTH SYSTEMS & ACCESS	"…this country *doesn't have a preventative mindset.*"	"In general, *maternal care is underfunded and underemphasized* in society…"
AWARENESS	"There is a need to educate pregnant women about the *importance of pre-natal care…*"	"*Assuring pregnant people* that screening will improve health outcomes for their child"

Conclusions and Limitations

Conclusions

- Learning materials for Panamanian medical students teach information on toxoplasmosis to U.S. medical students
- Many issues underlying lack of preventative protocols for toxo are reportedly similar between Panama & U.S.

Limitations

- Self-selection
- Small sample (low response rate)
- Single school/medical center

Results: Perceived barriers (qualitative)

THEME	EXAMPLE (Panama 2017)	EXAMPLE (U.S. 2020)
COST	"It would be great if the *$4 [point of care] test* was approved…"	"[H]ow *expensive is the treatment?"*
HEALTH SYSTEMS & ACCESS	"…this country *doesn't have a preventative mindset."*	"In general, *maternal care is underfunded and underemphasized* in society…"
AWARENESS	"There is a need to educate pregnant women about the *importance of pre-natal care…"*	"*Assuring pregnant people* that screening will improve health outcomes for their child"

Future Steps (U.S.)

(A) COST

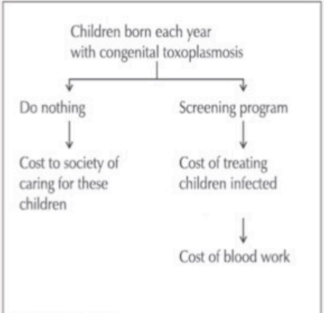

Sahai & Onyett 1996

Future Steps (U.S.)

(B) HEALTH SYSTEMS & ACCESS

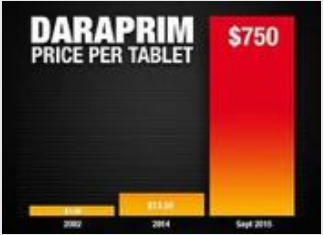

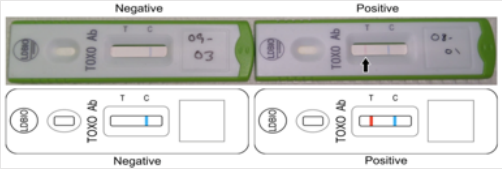

Future Steps (U.S.)

(C) AWARENESS

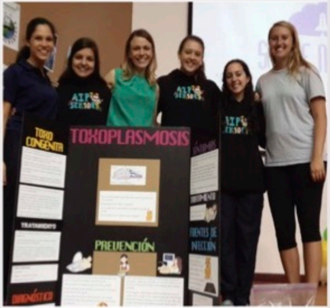

A Mother's Testimony in Support of The Prenatal and Neonatal Congenital Toxoplasmosis Prevention and Treatment Act

Posted on July 24, 2014 by John Easton

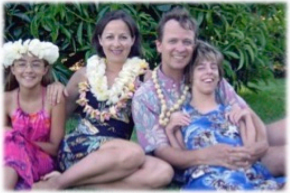

Rebecca, Janet, Rob, and Dana Morel, on vacation in Hawaii, 2005.

Acknowledgements

- Dr. Rima McLeod
  - Medical Director of UChicago Medicine's Toxoplasmosis Center
- Dr. Catherine Castro
  - Pritzker Class of 2020
- Pritzker School of Medicine Summer Research Program
- University of Chicago Center for Global Health
  - CGH summer seminar group
- Summer Research Program small group
- Students who participated in this study

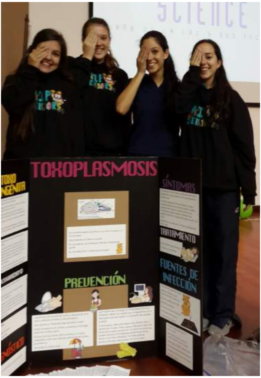

Silvia Fernández Pirla
with Najma Arjona; María Gabriela Bagatelas; Valeria García de Paredes

TOXOPLASMOSIS

Silvia Fernández Pirla
Najma Arjona
María Gabriela Bagatelas
Valeria García de Paredes

Toxoplasmosis en Panamá

- 4598 casos
- 418 casos por año
- 35 casos mensual durante el período de 1993-2003
- Factores como el clima tropical, la alta prevalencia de gatos y la exposición al suelo hacen que la condición hospitalaria de T. gondii permanezca en el ambiente durante un tiempo prolongado.

Toxoplasmosis en Panamá

- En mayo de 2014, Panamá aprobó una ley que obligaba a todos los médicos a examinar a las mujeres embarazadas durante el período de gestación e informar al Ministerio de Salud de todos los casos de toxoplasmosis.
- Un estudio reciente encontró que el 100% de las mujeres entre las edades de 11-14 años y el 60% de las mujeres entre las edades de 36-49 años tenían pruebas serológicas positivas para la infección por T. Gondii en el Hospital del Niño.

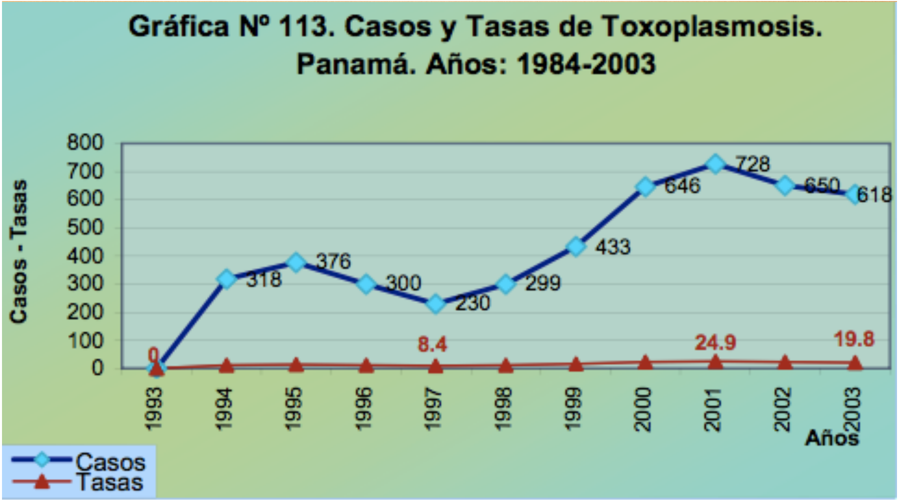
Gráfica Nº 113. Casos y Tasas de Toxoplasmosis.
Panamá. Años: 1984-2003
Casos - Tasas
800
700
600
500
400
300
200
100
0
318
376
300
230
299
433
646
728
650
618
0
8.4
24.9
19.8
1993
1994
1995
1996
1997
1998
1999
2000
2001
2002
2003
Años
Casos
Tasas

Cuadro Nº 157. Tasas Acumuladas De Toxoplasmosis Según Región De Salud República De Panamá. Periodos: 1981-2003

Región de Salud	1981-1985		1986-1990		1991-1995		1996-2000		2001-2003	
	C	T(2)	C	T(3)	C	T (4)	C	T (5)	C	T (6)
**República**	**1448**	**70.6**	**1073**	**44.7**	**1365**	**53.8**	**1908**	**69.0**	**1996**	**67.4**
Bocas del Toro	8	11.5	4	5	26	23.4	43	32.5	87	86.9
Coclé	23	14.9	30	17.9	84	45.4	168	85.8	65	31.2
Colón	7	3.8	6	3	3	1.5	17	8.7	13	6.1
Chiriquí	136	41.1	219	59.5	441	111.1	767	181.2	1089	287.5
Darién	4	11.7	-	-	-	-	0	0.0	0	0.0
Azuero	233	133.1	304	164.7	126	70.2	44	24.1		
Herrera							33	32.1	43	40.9
Kuna Yala	1	2.6	*	-	3	8.1	0	0.0	0	0.0
Los Santos							177	222.1	89	104.5
Panamá (1)	323	35.5	*	-	*	-	522	39.8	552	38.0
Panamá Este	*	-	1	2	18	50.3	14	15.3	15	18.6
Panamá Metro (2)	647	69.5	343	41.5	258	40.7	197	36.2	269	46.8
Panamá Oeste	-	-	67	35.1	182	79.3	189	76.5	155	43.8
San Miguelito	*	-	*	-	67	24.4	122	28.5	113	25.5
Veraguas	66	33.3	99	46.4	157	72.6	137	61.6	58	26.8
Ngöbe Buglé							0	0.0	0	0.0

TASA DE TOXOPLASMOSIS, EN LA REPÚBLICA DE PANAMÁ, SEGÚN REGIÓN DE SALUD. AÑO : 2003

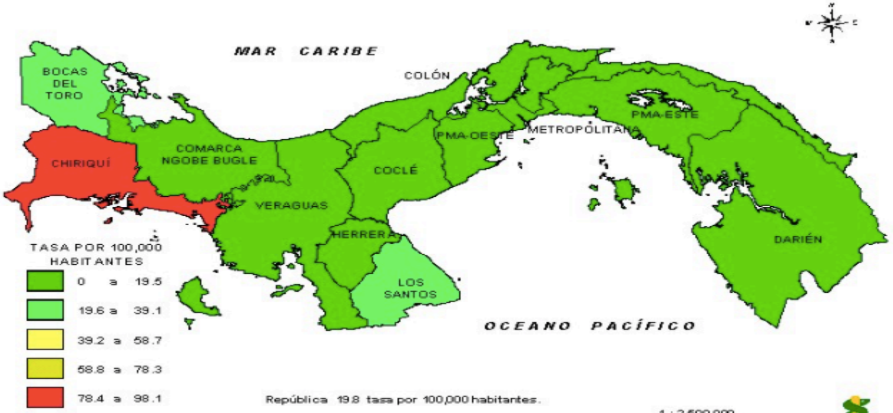

Fuente : Depto. de Vigilancia de F.P.R.S.E.- Minsa.
Elaborado por : D.A.S.T.S.- Sección de Seguimiento y Evaluación de Proyectos.

salud

Cuadro Nº 159. Casos de Toxoplasmosis Según Grupo de Edad. Panamá. Años: 1999-2003

Grupo de Edad	Años					
	Total	1999	2000	2001	2002	2003
Total	3075	433	646	728	650	618
< 1 año	36	3	6	13	5	9
1-4	178	24	21	40	60	33
5-9	423	59	96	114	89	65
10-14	518	81	119	117	110	91
15-19	352	58	89	76	61	68
20-24	273	37	49	81	53	53
25-49	965	141	198	220	184	222
50-54	251	21	54	51	67	58
55 y +	79	9	14	16	21	19

Fuente: Departamento de Vigilancia de Factores Protectores y de Riesgos a la Salud y Enfermedades. Año: 2004.

SENACYT
Secretaría Nacional de Ciencia, Tecnología e Innovación

INDICASAT-AIP
INSTITUTO DE INVESTIGACIONES CIENTÍFICAS AVANZADAS Y SERVICIOS DE ALTA TECNOLOGÍA

Congenital Toxoplasmosis in Panama: Current Situation and Recommendations

Mariangela Soberón, J.D.

Seroprevalence in Pregnant Women in Panama

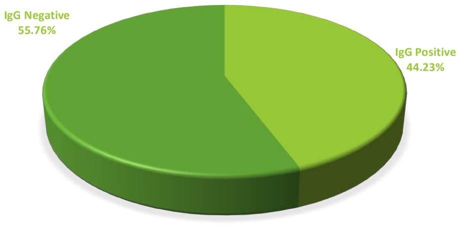

Mandatory Reporting

Officials can get information:

- in REAL TIME

- about events that put the population's health at risk

- that allows us to quickly identify outbreaks and epidemics

- that helps officials ground prices of medications and medical supplies in real statistics

- that allows for quick and efficient workflow

- that helps us identify areas of greatest prevalence and to prevent new cases from emerging

Mandatory Reporting:

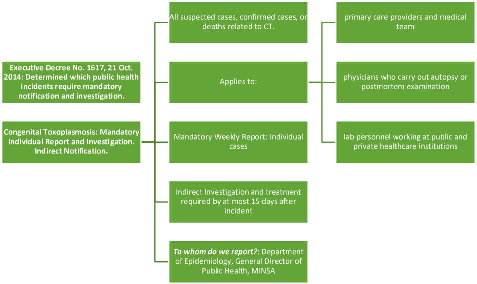

National Protocol for Prevention, Diagnosis, and Treatment of CT

Prevention:

- No direct prevention campaign
- Available literature does not consider current issues
- MINSA/CSS: under-reporting of cases

Diagnosis:

- MINSA/CSS protocol: mandatory serologic tests for IgG and IgM twice during pregnancy
- HCP: Report of IgM and IgG results

National Protocol for CT Treatment

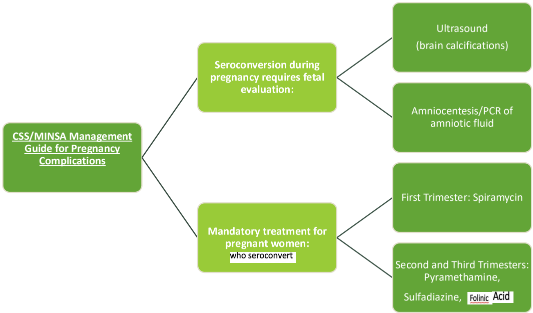

In Panama, the test pregnant women receive for *Toxoplasma gondii* is:

MANDATORY

FREE

If there is a seroconversion during the pregnancy:

- Treating the patient is required (following CSS/MINSA Requirements)

- Reporting the case is required

Seroprevalence and genetic makeup of *Toxoplasma gondii* in pregnant women, newborns, and domestic animals.

Updated data on incidence and prevalence

Determining risk factors

Strengthening the diagnosis and treatment protocols

Recommendations and Proposals

Participating patients: free diagnosis and treatment

Health sciences analysis helped us identify

- STRENGTHS
- WEAKNESSES
- RISKS
- OPPORTUNITIES

Strengths of the Healthcare System

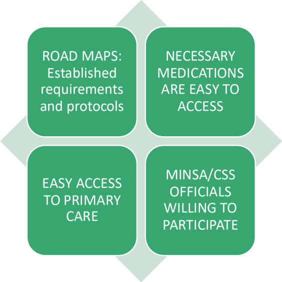

Weaknesses of the Healthcare System

Requirements and Protocols not enforced:

- No MANDATORY REPORTING/UNDER-REPORTING
- Not FREE OF CHARGE
- No FOLLOWING THROUGH with TEST PROTOCOLS/Prenatal controls
- Delays in filing e-applications

NO AVAILABILITY of REAGENTS for DIAGNOSTIC TESTS

NO AVAILABILITY of MEDICINES

NO NATIONAL PLAN for monitoring CT

Official statistics: CT cases from 2000-2016

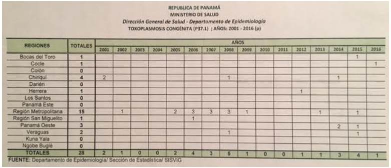

REPUBLICA DE PANAMÁ
MINISTERIO DE SALUD
Dirección General de Salud - Departamento de Epidemiología
TOXOPLASMOSIS CONGÉNITA (P37.1) ; AÑOS: 2001 - 2016 (p)

REGIONES	TOTALES	AÑOS															
		2001	2002	2003	2004	2005	2006	2007	2008	2009	2010	2011	2012	2013	2014	2015	2016
Bocas del Toro	1															1	
Cocle	1																1
Colón	0																
Chiriquí	4	2							1						1		
Darién	0																
Herrera	1												1				
Los Santos	0																
Panamá Este	0																
Región Metropolitana	15		1			2	3	3	3	1				1		1	
Región San Miguelito	1						1										
Panamá Oeste	3														2	1	
Veraguas	2								1							1	
Kuna Yala	0																
Ngobe Buglé	0																
TOTALES	28	2	1	0	0	2	4	3	5	1	0	0	1	1	3	4	1

FUENTE: Departamento de Epidemiología/ Sección de Estadística/ SISVIG

- 28 cases registered in 16 years of gathered data

- Serious public and private under-reporting

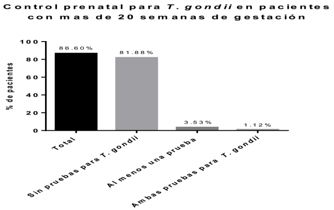
Control prenatal para T. gondii en pacientes con mas de 20 semanas de gestación
% de pacientes
100
80
60
40
20
0
86.60%
81.88%
3.53%
1.12%
Total
Sin pruebas para T. gondii
Al menos una prueba
Ambas pruebas para T. gondii

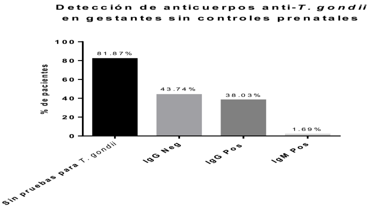
Detección de anticuerpos anti-T. gondii en gestantes sin controles prenatales
% de pacientes
100
80
60
40
20
0
81.87%
43.74%
38.03%
1.69%
Sin pruebas para T. gondii
IgG Neg
IgG Pos
IgM Pos

Risks Posed to the Healthcare System

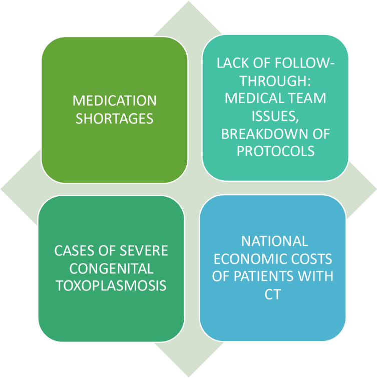

Opportunities for the Healthcare System

IMPROVE AND BROADEN THE LEGAL FRAMEWORK:

- Extend reporting requirements to more pregnant women
- Assess monthly testing in high-risk areas
- Create protocols for areas with low access to resources

CREATE AND IMPLEMENT PROTOCOLS THAT ARE EASY TO FOLLOW

- Standardized perinatal history
- Faster reference system for seroconversion cases
- Infectious disease consults

USE REAL-TIME DATA TO COORDINATE PURCHASES OF MEDICATIONS AND DIAGNOSTIC TESTS

ECONOMIC SAVINGS: PREVENTION vs TREATMENT

Strengthening our national healthcare system: How do we get there?

Healthcare system makes decisions based on scientific information.

Innovation:

- Mitigation measures to take if medications are not immediately available: development of new medications.
- Areas that have lower access to resources.
  - Telemedicine.

Technology:

- Implementation of the latest advances in diagnostic tests, especially in areas with lower access to resources.

Planning:

- Implementation of successful public health policies from other countries, e.g. France.
- With chronic toxoplasmosis:
  - Development of management protocols for reactivations.
  - Development of an economic model to determine relative costs for public health: prevention versus treatment.

Prevention:

- Establishment of prevention measures specific to each region's situation.

Acknowledgements:

Hospital del Niño "José Renán Esquivel"

- Dr. Maria Teresa Moreno, Dr. Xavier Saez Llorens, Dr. Ximena Norero

Hospital Santo Tomás:

- Dr. Carmen Charter, Dr. Migdalia Frías.
- Dr. Aris Mendieta, Dr. Jorge Espino
- Dr. Leisa Rosas, Dr. Geneva Gonzalez, Dra. Ana Bazo
- Dr. Osvaldo Reyes, Dr. Edwin Ortiz, Dr. Dario Beneddítto, Dr. Carlos Moreno
- Department of Infectious Diseases: Dr. Ana Belén Arauz.
- Laboratory: Jovana Borace PhD, Carlos Flores PhD, Evelyn Arauz PhD.
- Staff at the High-Risk Department, Maternity Wing of HST.
- Pharmacy: Moisés Márquez PharmD

Toxoplasmosis Center at the University of Chicago :

- Dr. Rima McLeod

COMMUNITY EDUCATION PRESENTATIONS IN PANAMA: 2014-2019

2017-2019: PREVALENCE AND RELATIONSHIP PATTERNS BETWEEN DIFFERENT RISK FACTORS ASSOCIATED WITH *T. GONDII* INFECTION IN PREGNANT WOMEN FROM PANAMA

- SPONSORED BY SENACYT, EXECUTED BY INDICASAT AIP
- MATERNITY WARD OF THE SANTO TOMAS HOSPITAL IN PANAMA CITY IN COLLABORATION WITH THE HOSPITAL DEL NIÑO JOSÉ RENAN ESQUIVEL, INDICASAT AIP.
- INTERNATIONAL COLLABORATION WITH THE TOXOPLASMOSIS CENTER, UNIVERSITY OF CHICAGO.
- SIZE OF THE SAMPLE: 3000 WOMEN

JANUARY 2019: LIVE PRESENTATION "SCIENTIFIC CAFÉ" HOSTED BY SENACYT. TOXOPLASMOSIS DURING PREGNANCY IN PANAMA, RECOMMENDATIONS FOR THE PUBLIC HEALTH SYSTEM.

OCTOBER 2018: APANAC INTERNATIONAL EVENT: SPECIAL PRESENTATION *TOXOPLASMOSIS, A ZOONOTIC DISEASE WITH CONGENITAL AND CHRONIC IMPLICATIONS.*

- SPONSORED BY SENACYT, EXECUTED BY INDICASAT AIP
- DIFFERENT SCIENTIFIC EDUCATIONAL ACTIVITIES.
- COLLABORATORS: THE TOXOPLASMOSIS CENTER UNIVERSITY OF CHICAGO, TOXOPLASMOSIS SEROLOGY LABORATORY PALO ALTO MEDICAL FOUNDATION-STANFORD UNIVERSITY, CENTRE HOSPITALIER UNIVERSITARIE DE LYON, UNIVERSITY OF TENNESSSEE, UNIVERSIDAD DE MINAS GERAIS.
- APANAC CONGRESS 2018, HOTEL SORTIS, PANAMA CITY.

OCTOBER 2016: SCIENTIFIC EDUCATIONAL ACTIVITIES RELATED TO CONGENITAL TOXOPLASMOSIS IN PANAMA.

- PRIVATE SPONSORS, EXECUTED BY INDICASAT AIP
- DIFFERENT SCIENTIFIC EDUCATIONAL ACTIVITIES UNIVERISTY OF PANAMA, APANAC
- COLLABORATORS: THE TOXOPLASMOSIS CENTER OF THE UNIVERSITY OF CHICAGO, TOXOPLASMOSIS SEROLOGY LABORATORY PALO ALTO MEDICAL FOUNDATION-STANFORD UNIVERSITY, CENTRE HOSPITALIER UNIVERSITARIE DE LYON, UNIVERSITY OF TENNESSSEE, GETTISBURG COLLEGE.

2015-2019 RESEARCH PROJECTS EXECUTED BY INTERNATIONAL STUDENTS IN COLLABORATION WITH THE TOXOPLASMOSIS CENTER OF THE UNIVERSITY OF CHICAGO, THE GLOBAL HEALTH CENTER OF THE UNIVERSITY OF CHICAGO, INDICASAT-AIP.

- OCTUBER 2018: STUDENT ASHELY AUE, MPP CANDIDATE CLASS OF 2019. UNIVERSITY OF CHICAGO, HARRIS SCHOOL OF PUBLIC POLICY.
- AUGUST-OCTOBER 2017:

- STUDENTS:
  - CATHERINE CASTRO, IMPACT OF GESTATIONAL AND CONGENITAL TOXOPLASMOSIS MEDICAL EDUCATION: A PRE AND POST INTERVENTIONAL STUDY IN PANAMA CITY.
  - DAVINA MOOSSAZADEH AND MARGARITA RAMIREZ: DATA ANALYSIS, FIELD PROYECT.
  - GUILLERMO PRADIEU. MPH CANDIDATE COLLEGE OF PUBLIC HEALTH, UNIVERSITY OF SOUTH FLORIDA: INTERNSHIP.

- JULY-AUGUST 2016
  - STUDENTS: ALIYA MOREIRA, ABHINAV PANDEY, SEROPREVALENCE OF TOXOPLASMA GONDII AT PREGNANTS WOMEN BASED ON MEDICAL RECORDS AT SAN MIGUEL ARCANGEL HOSPITAL AND THE MATERNITY WARD OF THE SANTO TOMAS HOSPITAL.
- JULY-AUGUST 2015
  - STUDENT: SHARON HEICHMAN, THE EFFECTIVENESS OF EDUCATIONAL MATERIALS ABOUT TOXOPLASMOSIS TO BENEFIT MATERNAL AND CHILD HEALTH IN PANAMA.
- AUGUST 2014
  - STUDENTS:
    - XUAN LI, TOXOPLASMOSIS EDUCATION FOR PREGNANT WOMEN IN PANAMA.
    - ASHTYN DIXON

Community initiatives: *APANAC.SYNYCYT, INDICASAT,University of Panama, Hospital Santo Tomas, Hospital San Miguel de Arcangel, Hospital del Nino*

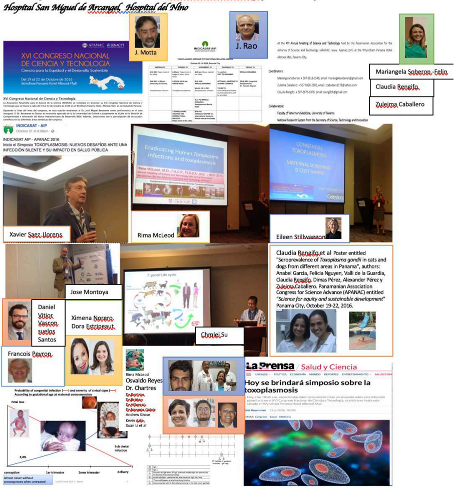

Screening for congenital toxoplasmosis: benefits and challenges abroad and at home

Eileen Stillwaggon, PhD

Outline

- Economics of prenatal screening to prevent Congenital Toxoplasmosis (CT) in Europe
- An economic model of costs and benefits of maternal screening in the United States
- Opportunity for a Panama model of screening and essential prenatal care

The European model of prenatal screening for toxoplasmosis

- Austria, since 1974
  - Bimonthly screening
- Slovenia, since 1995
  - Bimonthly screening
- France, since 1992
  - Monthly screening
- Only Austria’s program has been evaluated for economic costs and benefits

The Austrian Toxoplasmosis Register and the Austrian screening program

- Meticulous documentation of serology history and outcomes for all births
  - 1.4 million births
  - 1992 to 2008, with long-term follow-up to 2013
  - Screening at 8, 16, 24, 32 weeks, and at birth
- 93% of women had some screening
  - But very few had prescribed number of tests
- Treatment for newly infected mothers until birth
- Treatment for infected infants for first year

Austrian success

- Before the screening program
  - Mother-to-Child transmission = 50.8%
  - 78 cases of CT per 10,000 live births
- With screening and treatment
  - 70 women per year with new infections
    - **~ 9 per 10,000**
  - 8 babies per year with CT
    - **~ 1 per 10,000**
  - 81% of babies with CT had no clinical signs
  - All CT babies could go to school and enter workforce

What happened in Austria?

- Education reduced maternal infection through better food safety practices
- Early treatment reduced MTCT from 51% to 11%
- Education, testing, and early treatment reduced cases of CT from 78 per 10,000 to 1 per 10,000
- And no children have profound, lifelong injuries

The Paradox of Success

- Success is invisible
- Ministries of Health in France and Austria have threatened to shut down the toxoplasmosis screening programs
- *T. gondii* is still in the food supply
- Women are still at risk
- Babies are still at risk

PLOS | NEGLECTED TROPICAL DISEASES

RESEARCH ARTICLE

Congenital toxoplasmosis in Austria: Prenatal screening for prevention is cost-saving

Andrea-Romana Prusa[1], David C. Kasper[2], Larry Sawers[3], Evelyn Walter[4], Michael Hayde[1], Eileen Stillwaggon[5]*

Benefit-Cost Analysis (Doing the right thing is cheaper than neglect)

- Screening saved €450 million over 17 years
- The screening program costs €1.9 million per year
- The benefits are 14 times program costs

Prusa AR, Kasper DC, Sawers L, Walter E, Hayde M, and Stillwaggon E. Congenital Toxoplasmosis in Austria: Prenatal Screening for Prevention is Cost-saving, *PLoS Neglected Tropical Diseases*, 11(7), 2017.

How much is €1.9 million?

- Annual cost of toxoplasmosis screening and treatment = .003% of total Austrian budget
- Annual cost of the program that has prevented profound visual and cognitive injuries in infants and children is .0006% of Austrian GDP

United States

- Low prevalence ~ 11% (https://www.cdc.gov/parasites/toxoplasmosis/epi.html)
- High costs
  - Testing
  - Treatment
  - Special education
- No systematic screening at present

OPEN ACCESS Freely available online

PLoS NEGLECTED TROPICAL DISEASES

Maternal Serologic Screening to Prevent Congenital Toxoplasmosis: A Decision-Analytic Economic Model

Eileen Stillwaggon[1]*, Christopher S. Carrier[1], Mari Sautter[2], Rima McLeod[2]

1 Department of Economics, Gettysburg College, Gettysburg, Pennsylvania, United States of America, 2 Division of Ophthalmology and Visual Sciences, Department of Surgery, The University of Chicago, Chicago, Illinois, United States of America

USA: Screening is cost-saving

- Maternal screening and treatment for mothers and babies would save US$2.5 billion per year

Stillwaggon E, Carrier CS, Sautter M, McLeod R. Maternal Serologic Screening to Prevent Congenital Toxoplasmosis: A Decision-Analytic Economic Model, *PLoS Neglected Tropical Diseases* 5(9), 2011.

Point-of-Care Testing

- Reliable in variety of settings
- Inexpensive
- Adaptable to include tests for multiple diseases

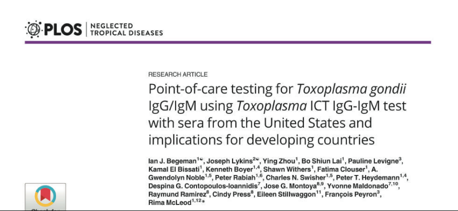

PLOS | NEGLECTED TROPICAL DISEASES

RESEARCH ARTICLE

Point-of-care testing for *Toxoplasma gondii* IgG/IgM using *Toxoplasma* ICT IgG-IgM test with sera from the United States and implications for developing countries

Ian J. Begeman[1☯], Joseph Lykins[2☯], Ying Zhou[1], Bo Shiun Lai[1], Pauline Levigne[3], Kamal El Bissati[1], Kenneth Boyer[1,4], Shawn Withers[1], Fatima Clouser[1], A. Gwendolyn Noble[1,5], Peter Rabiah[1,6], Charles N. Swisher[1,5], Peter T. Heydemann[1,4], Despina G. Contopoulos-Ioannidis[7], Jose G. Montoya[8,9], Yvonne Maldonado[7,10], Raymund Ramirez[8], Cindy Press[8], Eileen Stillwaggon[11], François Peyron[3], Rima McLeod[1,12]*

Begeman I, Lykins J, Zhou Y, Lai BS, Levigne P, El Bissati K, Boyer K, Withers S, Clouser F, Noble AG, Rabiah P, Swisher C, Heydemann P, Contopoulos-Ioannidis D, Montoya J, Maldonado Y, Ramirez R, Press C, Stillwaggon E, Peyron F, McLeod R. Point-of-care Testing for *Toxoplasma gondii* IgG/IgM Using *Toxoplasma* ICT IgG-IgM Test with Sera from the United States and Implications for Developing Countries, *PLoS Neglected Tropical Diseases*, 11(6), 2017.

Panama's Opportunity

- National screening for toxoplasmosis in pregnancy is mandatory by Panamanian law since 2014
- Inexpensive point-of-care testing makes this feasible
- Multiplex testing, with one stick:
  - Toxoplasmosis, HIV, syphilis, Chagas disease, etc.
- A new platform for prenatal care

Broad scope, lower costs

Along with mandated toxoplasmosis testing, a basic nurse visit can:

- Check blood pressure for pre-eclampsia
- Check urine for gestational diabetes
- Check for common vaginal infections
- Test for multiple diseases that threaten mothers and babies

Panama has the opportunity to create a new model of essential prenatal care on a national scale.

Thank you

¡Hasta pronto!

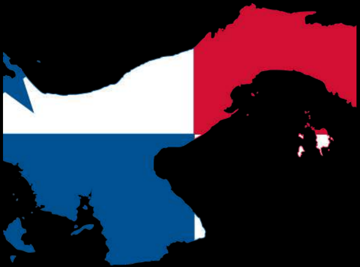

Supplement: 1832235_Sup_Material_3 [file NIHMS1832235-supplement-1832235_Sup_Material_3.pdf]
